# The 2025 British Society for Rheumatology guideline for the treatment of axial spondyloarthritis with biologic and targeted synthetic DMARDs

**DOI:** 10.1093/rheumatology/keaf089

**Published:** 2025-04-09

**Authors:** Sizheng Steven Zhao, Stephanie R Harrison, Ben Thompson, Max Yates, Joe Eddison, Antoni Chan, Nick Clarke, Nadia Corp, Charlotte Davis, Lambert Felix, Kalveer Flora, William J Gregory, Gareth T Jones, Christopher A Lamb, Helena Marzo-Ortega, Daniel J Murphy, Harry Petrushkin, Virinderjit Sandhu, Raj Sengupta, Stefan Siebert, Danielle A Van Der Windt, Dale Webb, Zenas Z N Yiu, Karl Gaffney

**Affiliations:** Centre for Musculoskeletal Research, Division of Musculoskeletal and Dermatological Science, School of Biological Sciences, Faculty of Biological Medicine and Health, The University of Manchester, Manchester Academic Health Science Centre, Manchester, UK; NIHR Manchester Biomedical Research Centre, Manchester University NHS Foundation Trust, Manchester, UK; Leeds Institute of Rheumatic and Musculoskeletal Medicine, University of Leeds, Leeds, UK; Leeds NIHR Biomedical Research Centre, Leeds Teaching Hospitals NHS Trust, Leeds, UK; Rheumatology Department, The Newcastle-upon-Tyne Hospitals NHS Foundation Trust, Newcastle upon Tyne, UK; Centre for Epidemiology, Norwich Medical School, University of East Anglia, Norwich, UK; Rheumatology Department, Norfolk & Norwich University Hospitals NHS Foundation Trust, Norwich, UK; Expert by Experience, Leeds, UK; University Department of Rheumatology, Royal Berkshire NHS Foundation Trust, Reading, UK; Expert by Experience, Norwich, UK; Primary Care Centre Versus Arthritis, School of Medicine, Keele University, Staffordshire, UK; Department of Rheumatology, The Leeds Teaching Hospitals NHS Trust, Leeds, UK; Primary Care Centre Versus Arthritis, School of Medicine, Keele University, Staffordshire, UK; Pharmacy Department, London North West University Healthcare NHS Trust, London, UK; Rheumatology Department, Salford Royal Hospital, Northern Care Alliance NHS Foundation Trust, Greater Manchester, UK; Faculty of Health and Education, Manchester Metropolitan University, Manchester, UK; Aberdeen Centre for Arthritis and Musculoskeletal Health (Epidemiology Group), University of Aberdeen, Aberdeen, UK; Translational & Clinical Research Institute, Faculty of Medical Sciences, Newcastle University, Newcastle upon Tyne, UK; Department of Gastroenterology, The Newcastle upon Tyne Hospitals NHS Foundation Trust, Newcastle upon Tyne, UK; Leeds Institute of Rheumatic and Musculoskeletal Medicine, University of Leeds, Leeds, UK; Leeds NIHR Biomedical Research Centre, Leeds Teaching Hospitals NHS Trust, Leeds, UK; Honiton Surgery, Department of Rheumatology, Royal Devon & Exeter Hospital, Exeter, UK; Uveitis and Scleritis Service, Moorfields Eye Hospital NHS Foundation Trust, London, UK; Department of Rheumatology, St George’s University Hospitals NHS Foundation Trust, London, UK; Royal National Hospital for Rheumatic Diseases, Royal United Hospitals, Bath, UK; School of Infection and Immunity, University of Glasgow, Glasgow, UK; Primary Care Centre Versus Arthritis, School of Medicine, Keele University, Staffordshire, UK; National Axial Spondyloarthritis Society (NASS), London, UK; Dermatology Centre, Salford Royal Hospital, Northern Care Alliance NHS Foundation Trust, Salford, UK; Rheumatology Department, Norfolk & Norwich University Hospitals NHS Foundation Trust, Norwich, UK

**Keywords:** axSpA, ankylosing spondylitis, biologics, targeted synthetic DMARDs, treatment, management, guideline, recommendations, extra-musculoskeletal manifestations, uveitis, psoriasis, inflammatory bowel disease

## Abstract

**Figure:**
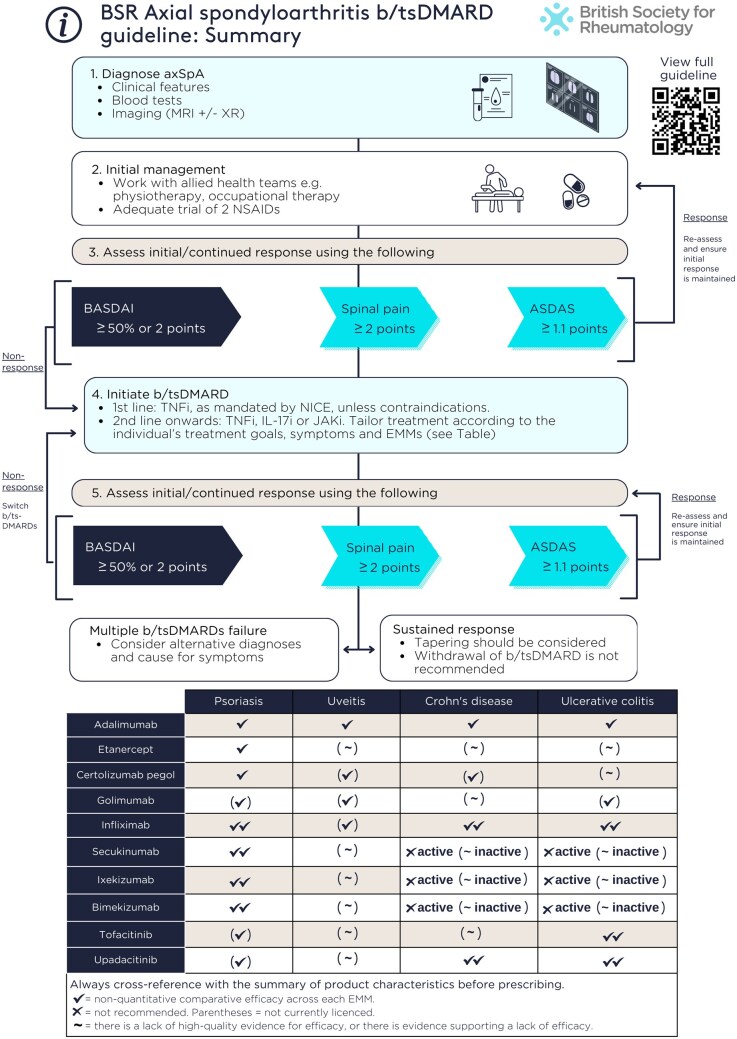


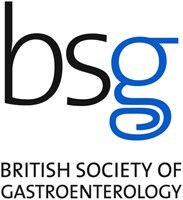



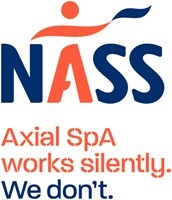



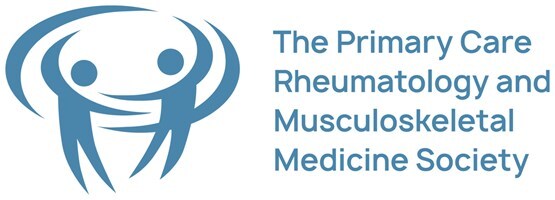



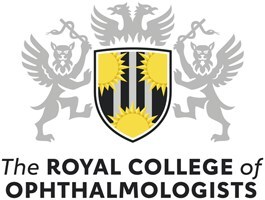



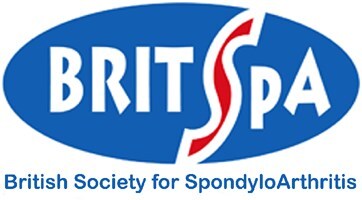


## Background

Axial spondyloarthritis (axSpA) is a chronic inflammatory condition that predominantly affects the spine and sacroiliac joints [1]. It can also involve peripheral joints and entheses, and extra-musculoskeletal manifestations (EMMs) such as acute anterior uveitis, psoriasis and IBD. Symptoms of axSpA typically begin in early adulthood but diagnosis can often take several years [2]. Chronic inflammatory pain and stiffness are well recognized as having adverse effects on quality of life, social participation and mental health [3–5].

### Need for guideline update 

Pharmacological management has advanced considerably since the previous BSR axSpA guideline [6] to incorporate new classes of biologic DMARDs (bDMARDs, including biosimilars), targeted synthetic DMARDs (tsDMARDs) and treatment strategies such as drug tapering. Therapeutic options for treating EMMs as index conditions have similarly evolved. The increasingly complex therapeutic landscape, with varying efficacy and safety of drugs for each disease manifestation, forms the context in which we aimed to update the BSR guideline for the treatment of axSpA with b/tsDMARDs. The key questions that the guideline sought to answer were published in the guideline scope [7], including the effectiveness and safety of targeted therapies; switching, combining, tapering or withdrawing targeted therapies; and treating to target. The guideline applies only to adults with axSpA. For brevity, we refer to b/tsDMARDs as “targeted therapies” throughout.

### Target audience

This guideline is for health professionals in the UK who directly care for adults with axSpA (including but not limited to rheumatologists, rheumatology specialist nurses, allied health professionals, rheumatology specialty trainees, pharmacists), people living with axSpA and other stakeholders.

### The areas the guideline does not cover

- NSAIDs, glucocorticoids and conventional synthetic DMARDs.
- Treatment of enthesitis/spondylitis-related juvenile idiopathic arthritis.
- Axial disease in psoriatic arthritis [8].
- Safety of targeted therapies [9] or their use in pregnancy [10].
- Health economic considerations.

### Stakeholder involvement

The guideline was developed by a multidisciplinary guideline working group (GWG), comprising and reflecting the views of individuals with lived experience of axSpA, rheumatologists, an ophthalmologist, a dermatologist, a gastroenterologist, a general practitioner, an epidemiologist, a specialist nurse, a consultant physiotherapist, a specialist pharmacist and the Chief Executive Office (CEO) of the patient-focused charity National Axial Spondyloarthritis Society (NASS). Drafting of the overarching principles was led by authors with lived experience of axSpA. Details of the GWG and their declared conflicts of interest are included at the end of this article and are available on the BSR website. The guideline was available for public consultation on the BSR website for a month prior to publication and was reviewed by the BSR Guideline Steering Group and external expert peer reviewers.

### Rigour of development

This guideline was developed in accordance with the BSR Creating Guidelines Protocol (v5.4). The guideline and recommendations were underpinned by a systematic literature review.

## Literature review

### Searches

The full methodology and evidence tables are provided in Supplementary Data S1, available at *Rheumatology* online. The literature search was informed by the guideline scope [7] and registered in advance (PROSPERO: CRD42023437846). A literature review specialist (NC) performed searches across two databases (MEDLINE, EMBASE) and The Cochrane Library without language restriction, covering the period between 30th June 2014 (review date for the previous version of the guideline) and 17th April 2023. Full search details are provided in Supplementary Data S2.

### Screening and selection

Eligibility criteria were agreed for randomized controlled trials (RCTs, for efficacy and safety) and observational designs (safety only). For observational evidence, only representative multi-site cohort studies or studies conducted using disease registries or electronic health record data were considered eligible. Other study designs, including cross-sectional studies, case-control designs, case series and other publication types (editorials, commentaries, trial protocols, letters, trials registry records and study protocols) were excluded, as well as full papers in any language other than English without an English translation. A detailed description of inclusion and exclusion criteria and a PRISMA flow diagram are provided in Supplementary Data S3 and S4, available at *Rheumatology* online, respectively. Two reviewers independently screened the first 10% of titles to ensure good agreement. For the remaining 90%, one reviewer screened titles, excluding studies that were clearly irrelevant. Abstracts, and then full texts were screened against eligibility criteria by one reviewer, with up to 20% double screened by a second reviewer to ensure accuracy. Any disagreements were resolved by a third reviewer.

### Data extraction and risk of bias assessment

A standardized data extraction form was developed, piloted and used to collect data for analysis. Data were collected on country, study design, characteristics of the study population (including radiographic or non-radiographic axSpA and the presence of comorbidities and EMMs), intervention characteristics and efficacy and safety outcomes. Cochrane risk of bias tool [11] was used to assess risk of bias for RCTs and controlled clinical trials. For cohort designs, relevant bias domains were used from the ROBINS-I tool for assessing risk of bias in non-randomized intervention studies [12]. Data extraction and risk of bias assessment were undertaken by one reviewer and independently checked by a second for correctness and consistency. Disagreements were resolved by consulting a third reviewer if necessary.

## Quality of evidence

Evidence tables were prepared for each guideline question (Supplementary Data S1, available at *Rheumatology* online). GRADE [13] was used to summarize certainty in the evidence for each outcome across studies for each guideline question, separately for RCTs and observational studies, and separately for each drug category, and efficacy or safety outcome. As this was an update of an existing guideline, GRADE was applied to evidence identified from the update searches only (2014–2023), so does not reflect all evidence available for each intervention.

Quality of evidence for each outcome was graded where A represents high, B moderate and C low/very low quality of evidence. “High quality” suggest that further research is *very unlikely* to change the confidence in the effect estimate (e.g. from well-performed RCTs or observational studies). Evidence was downgraded to moderate, low or very low based on concerns related to study design, risk of bias, inconsistency, indirectness (applicability) or imprecision. “Moderate quality” suggests that further research is *likely* to have an important impact on the confidence in the effect estimate and may change the estimate (e.g. from RCTs with important limitations, or from other study designs with special strength). “Low or very low quality” suggests that further research is *very likely* to have an important impact on confidence in the effect estimate and is likely to change the estimate (e.g. observational studies or RCTs with very serious limitations).

## Strength of agreement

Each recommendation was evaluated by all members of the GWG and subjected to a vote relating to strength of agreement (SoA) on a scale of 1 (total disagreement) to 100 (total agreement). The strength of agreement for each recommendation is presented as the mean of the GWG’s individual ratings, expressed as a percentage. Anonymized votes are shown in Supplementary Data S5, available at *Rheumatology* online.

## Strength of recommendation

A rating of 1 (strong) is given where the GWG feels that benefits clearly outweigh the risks; 2 (conditional) when risks and benefits are more closely balanced or more uncertain.

The recommendation statements are presented at the beginning of each section, accompanied by the strength of recommendation, quality of supporting evidence and strength of agreement in parentheses.

## Plan for review

This guideline is planned for update in 5 years.

## The guideline

### Overarching principles

1. The primary goal of treatment for people living with axSpA is to enable them to lead healthy and productive lives by optimizing health-related quality of life through comprehensive management of all disease manifestations, prevention of structural damage, preservation of physical function, work productivity and social participation (SoA 99%).
2. Management decisions should be developed in partnership with the individual living with axSpA based on their needs and priorities, within the available resources (SoA 99%).
3. Management should involve a multidisciplinary team coordinated by a rheumatologist, utilizing a holistic approach that incorporates both pharmacological and non-pharmacological interventions (SoA 98%).

Generic overarching recommendations reflect generally accepted best practices and consensus of expert opinion. The focus of treatment is to optimize health-related quality of life by placing the person living with axSpA at the centre of care provision. Providing information and education is essential to enable meaningful engagement in shared decision-making. The decision to start or change targeted therapy (for musculoskeletal manifestations) should be overseen by the responsible consultant rheumatologist and made in partnership with the person with axSpA, taking into account individual needs and priorities to support a healthy and productive life. Treatment goals should be reviewed regularly to ensure they remain realistic, achievable and acceptable to the person with axSpA.

Optimal management involves addressing axial and peripheral involvement, EMMs and comorbidities. In the presence of EMMs, holistic management should include cross-speciality collaboration. When selecting targeted therapies, consider that people with axSpA may prioritize controlling some disease manifestations over others. Care of EMMs should ideally be coordinated by the specialty managing the manifestation with the greatest impact, taking into account differential licencing and dosing for each indication. The number of therapeutic options for axSpA remains limited compared with other immune mediated inflammatory diseases [14]. In the context of well-controlled axSpA, consider managing mild EMMs without changing targeted therapy where possible and appropriate.

Management of comorbidities should adopt an multidisciplinary team (MDT) approach (e.g. nurse-led annual review of cardiovascular and fracture risk, clinical psychology for mental health) in close collaboration with primary care.

Escalation to targeted therapies should not diminish the focus on non-pharmacological management. Although it is beyond the scope of this guideline to make recommendations for non-pharmacological therapies, they are essential for the holistic approach to managing and living well with axSpA. Non-pharmacological and supported self-management strategies should remain at the forefront despite the increasing availability of pharmacological options. The GWG emphasizes the importance of the following:

- Physical activity, supervised exercise and physiotherapy are foundational for axSpA management. Supervised therapies have a stronger evidence base [15–17], but all forms of regular physical activity are likely to provide axSpA-specific as well as general health benefits.
- Aquatic physiotherapy and hydrotherapy are well established in axSpA management and are particularly beneficial for those who cannot tolerate land-based exercises [18, 19].
- Psychological therapies are important to address the high burden of mental health comorbidity among people with axSpA [5], and can range from remotely delivered cognitive-behavioural therapy to clinical psychology as part of the MDT.
- Supported self-management: people with axSpA should be empowered to manage their condition through need-based education, including being directed to appropriate resources for additional information and support.

### Recommendations

- TNF, IL-17 or JAK inhibitors are recommended for people with active axSpA who have not responded adequately despite non-pharmacological and conventional pharmacological management (1A, SoA 97%)
  The currently licenced targeted therapies for axSpA in the UK include TNF inhibitors (TNFi: adalimumab, certolizumab pegol, etanercept, golimumab, infliximab and relevant biosimilars), IL-17 inhibitors (IL-17i: secukinumab, ixekizumab, bimekizumab) and JAK inhibitors (JAKi: tofacitinib, upadacitinib). Infliximab and tofacitinib are not licenced for non-radiographic axSpA. All targeted therapies have demonstrated an acceptable balance of efficacy and safety in axSpA RCTs (Supplementary Data S1, available at *Rheumatology* online). There is no evidence to support recommending one class or drug over another in terms of efficacy for musculoskeletal manifestations. The decision to escalate to targeted therapies and the choice of therapy should be made with the person with axSpA, taking into account prognostic factors, comorbidities (not discussed further, see [9]) and EMMs (summarized in Table 1 and discussed in subsequent recommendations).
  Not all people with axSpA will undergo structural progression detectable on radiographs (radiographic progression) [20]. However, observational studies have shown that certain groups (e.g. males, smokers, those with high baseline damage or high CRP [21]) have greater risk and rate of radiographic progression. These adverse prognostic factors should be considered as part of shared decision-making when initiating targeted therapies. Although generating RCT evidence for the effect of therapies on radiographic progression is challenging, current observational literature suggests that targeted therapies (specifically TNFi) are more likely to reduce radiographic progression compared with NSAIDs [22].
- Active disease should be determined by the treating clinician in the context of verified diagnosis and inflammatory disease activity, supported by validated indices such as the Axial Spondyloarthritis Disease Activity Score (ASDAS), BASDAI and spinal pain (1B, SoA 97%).
  For the purpose of escalation to targeted therapies, active disease should be defined after (1) appropriate use of non-pharmacological and conventional pharmacological therapies, and (2) verifying the diagnosis and inflammatory disease activity. The diagnosis of axSpA should be verified by a consultant rheumatologist, and includes ankylosing spondylitis (radiographic axSpA) and non-radiographic axSpA with objective features of inflammation (elevated CRP and/or MRI findings). Recommendations on diagnosis are beyond the scope of this guideline. However, the GWG emphasizes that the Assessment of Spondyloarthritis International Society (ASAS) classification criteria should not be used for diagnosis [23]. In the general population, most individuals positive for HLA-B27 will not develop axSpA [24], and non-pathological inflammatory changes on MRI can be highly prevalent (e.g. postpartum, among the physically active [25–27]). The necessary push to reduce diagnostic delay must be cautiously balanced against the potential for misdiagnosis.
  The decision to initiate therapy should be agreed upon with the person with axSpA, rather than being based solely on disease indices. Nevertheless, validated measures of disease activity should be documented at the time of treatment initiation and at each follow-up. The GWG recommends using the ASDAS, BASDAI and spinal pain for assessing disease activity (numerical rating scale preferred by ASAS over visual analogue scale [28]). Although BASDAI and spinal pain are included in NICE recommendations due to their use in historical clinical trials, their subjective nature means that scores can potentially be influenced by non-disease-specific factors such as comorbidities [29, 30]. ASDAS is the only instrument shown to correlate with radiographic progression [31, 32] and is included in almost all contemporary clinical trials in axSpA. Definitions of high disease activity using ASDAS ≥ 2.1 or BASDAI ≥ 4 usually coincide but, when discordant, the ASDAS definition better predicts treatment response [33, 34]. For these reasons, the GWG recommends transitioning towards regular inclusion of ASDAS in clinical practice (Table 2).
  Verifying diagnosis and assessing inflammatory disease activity can be clinically challenging. When there is doubt about the extent of inflammation, imaging such as MRI for axial and ultrasound for peripheral manifestations may be helpful. Detailed MRI recommendations have been provided by the British Society for Spondyloarthritis [35, 36] and ASAS [37] and will not be discussed further.
- Response to targeted therapies should be assessed using validated indices (e.g. ASDAS, BASDAI, spinal pain) 3–4 months after initiation, and every 6–12 months if treatment is continued (1B, SoA 97%).
- The absence of response to targeted therapies should prompt reassessment of the diagnosis and the extent of inflammatory disease activity (1B, SoA 100%).
  Once started, it is important to assess the effectiveness, tolerability, compliance and the appropriateness of continuing therapy. Follow-up assessment of disease activity should be holistic and supported by, but not solely reliant on, disease indices. The decision to continue therapy should be made jointly between the person with axSpA and the treating clinician. Treatment response should be evaluated at a minimum of 12 weeks after initiation. NICE recommends assessing response to TNFi after 12 weeks [38]; secukinumab, bimekizumab and JAKis after 16 weeks [39–42] and ixekizumab after 16–20 weeks [43]. The 2013 axSpA guideline recommended 6-monthly reviews. For the current guideline, the nature and interval of follow-up were debated after feedback from the public consultation. Concerns were raised that specifying assessments “every 6–12 months” might conflict with patient-initiated follow-up pathways. The GWG acknowledged that high-quality care can be maintained with longer intervals. However, removing the interval recommendation entirely could be open to misinterpretation that regular follow-up is optional and make auditing difficult. The GWG added that the nature and interval of follow-up can be adjusted based on individual circumstances but should be reviewed at each visit to ensure it remains appropriate. Patient-initiated follow-up or extended follow-up intervals may be considered if the condition is well-controlled and the person with axSpA has adequate education and access to a local rheumatology advice line or equivalent to promptly re-establish contact with the clinical team if necessary. Follow-up interval should not typically exceed 24 months.
  NICE recommends a BASDAI 50% or 2-unit reduction and 2-unit reduction in spinal pain [44] which, until revised, will continue to be the cornerstone of assessment. However, treatment response can be influenced by the presence of comorbidities, particularly for more subjective indices such as BASDAI and spinal pain [30, 45]. Moreover, BASDAI is not associated with radiographic disease progression [21, 46]. ASDAS disease activity states and improvement criteria are superior to BASDAI in differentiating levels of, and change in, disease activity [47]. Routine use of ASDAS is endorsed by ASAS-EULAR and OMERACT [48, 49]. For these reasons, the GWG recommends that assessments incorporate ASDAS. A reduction of ≥1.1 represents a clinically important response [50].
  When using any index, assessments should consider whether residual symptoms are related to active inflammation; e.g. fibromyalgia may contribute to high tenderness and fatigue domains of BASDAI, while obesity may contribute to elevated CRP for ASDAS [51]. As discussed in recommendation (ii), repeat MRI of the whole spine and sacroiliac joints may help to assess inflammation and other causes of persistent symptoms. Diagnosing axSpA is challenging, and clinicians should remain open to re-evaluating the original diagnosis, particularly when there is (repeated) primary non-response to targeted therapies.
- An alternative targeted therapy is recommended for individuals with active disease who cannot tolerate, do not respond to, or lose response to the initial targeted therapy (1A, SoA 99%).
  As with overarching and treatment initiation recommendations, decisions for treatment switching should be shared and agreed upon with the person with axSpA. There is currently insufficient evidence to recommend a specific sequence of targeted therapies in the case of treatment failure in axSpA. TNFi are currently mandated as first-line by NICE, unless they are unsuitable [44]. In people with insufficient response to TNFi (TNFi-IR), a second TNFi has been shown in observational studies (but not RCTs) to be effective, albeit less so than the first [52]. The efficacy of IL-17i in those with TNFi-IR has been demonstrated in RCTs [53–58]. The efficacy of tofacitinib in TNFi-IR [59], and upadacitinib in TNFi-IR or IL-17i-IR [60], has also been shown in RCTs.
  Response to a second TNFi is lower in axSpA with primary non-response to the first TNFi, compared with those who switch because of secondary non-response [61, 62]. It is probable, as in other inflammatory arthritides, that people with axSpA who have primary non-response to one therapy are more likely to respond to a drug with a different mechanism of action.
  Switching from a bio-original drug to its equivalent biosimilar is not recommended when there is insufficient response to the former.
  In the context of verified diagnosis and inflammatory disease activity, the GWG suggests that there should not be a limit to the number of sequential therapies that any individual can have. This should include consideration of new therapies as they become available. Evidence for the safety and effectiveness of sequential therapy in axSpA is lacking and represents an important unmet research need. In axSpA, observational evidence is limited to cycling within the TNFi class, where the proportion achieving remission reduces across first-to third-line TNFi [62]. In PsA [63] and RA [64], responses reduce numerically across lines of therapy; however, an important proportion of participants were still able to achieve remission, up to the sixth-line in the case of RA.
- In the presence of moderate-to-severe or recurrent uveitis, a monoclonal TNFi is preferred over therapies with other mechanisms of action (1A, SoA 98%).
- A history of inactive uveitis is not an absolute contraindication to therapies with other mechanisms of action (2B, SoA 97%).
- If new uveitis develops in the context of well-controlled axSpA, decisions to change treatment should be made with an ophthalmologist where possible, taking into account the severity and/or frequency of uveitis flares and response to topical steroid (1B, SoA 97%).
  Acute anterior uveitis is the most common EMM that can occur in up to a quarter of people with axSpA [65]. In accordance with NICE, new presentations of uveitis should be assessed by an ophthalmologist within 24 hours [66].
  The severity of uveitis can range from infrequent mild episodes to recurrent or sight-threatening disease. For moderate–severe or recurrent uveitis, the decision to commence a targeted therapy should be jointly made between rheumatology and ophthalmology as part of an MDT. Mild cases can be effectively managed with topical therapy and monitoring of side effects (e.g. steroid-induced ocular hypertension and cataract).
  Monoclonal TNFi are effective for the treatment of anterior uveitis in axSpA [67], although no targeted therapies are specifically licenced for this condition (adalimumab is licenced for non-infectious intermediate, posterior or panuveitis). Observational evidence suggests that etanercept and IL-17A inhibitors may be less effective at controlling uveitis than monoclonal TNFi [68]. In contrast to IBD, trial data do not suggest that IL-17Ais are harmful for uveitis [69]. A *post hoc* analysis of pooled axSpA trial data published after the full literature search suggested that bimekizumab may be superior to placebo for preventing uveitis flares [70]. A network meta-analysis of axSpA RCTs suggested that all targeted therapies are likely superior to placebo [71]. If uveitis develops or flares in individuals with well-controlled axSpA on etanercept or IL-17Ai, the severity and/or frequency of uveitis should be considered in consultation with an ophthalmologist before automatically switching therapy.
- IL-17 and monoclonal TNFi are preferred in the presence of extensive psoriasis (e.g. >10% body surface area) or severe localized psoriasis at sites associated with high functional impairment or impact (e.g. face, scalp, palms, soles, flexures, genital or nails), ideally in conjunction with a dermatologist (1A, SoA 96%).
  Among people with axSpA, psoriasis has a prevalence of ∼10% [65, 72] and is typically reported as being mild [73]. For mild cutaneous psoriasis, concomitant topical therapy can be recommended to and managed by the GP according to NICE guidance [74].
  In people with well-controlled axSpA but inadequately controlled psoriasis, management should be discussed with a dermatologist and may not necessarily require a change in targeted therapy. For example, topical, photo, and systemic non-biologic therapies may provide sufficient control [74]. Where axSpA and psoriasis are both indications for targeted therapy, control of cutaneous psoriasis can be achieved by, in order of efficacy, IL-17i (bimekizumab was superior to secukinumab for skin control but increased incidence of candidiasis [75]), monoclonal TNFi or etanercept [76]. In dermatology settings, etanercept (though licenced) is used infrequently after monoclonal TNFi [77], while golimumab, tofacitinib and upadacitinib are not currently licenced for psoriasis.
  While detailed psoriasis assessment (e.g. Psoriasis Area and Severity Index) in rheumatology may be impractical outside academic settings, the GWG recommends using at least one objective measure for assessing and monitoring psoriasis. For example, body surface area can be estimated using the palm method, where the individual’s palm covers ∼1% of their body surface area.
  In people with psoriasis who subsequently develop axSpA, or in people with predominant skin involvement where targeted therapies have been led by dermatologists, decisions to switch therapy should consider that certain therapies used in psoriasis have no beneficial effect for axial symptoms (e.g. ustekinumab or IL-23p19 inhibitors), while other inhibitors of IL-17 signalling have (e.g. brodalumab [78]) or are likely to have therapeutic benefit despite not being licenced for axSpA.
- Individuals with unexplained lower gastrointestinal symptoms should be assessed by a gastroenterologist, ideally before commencing targeted therapies (1B, SoA 97%).
- In the presence of active IBD, monoclonal TNFi or JAKi are preferred; IL-17 inhibitors should not be commenced (1A, SoA 99%).
- A history of inactive IBD is not an absolute contraindication to IL-17 inhibitors or etanercept (2B, SoA 97%).
  People with unexplained lower gastrointestinal symptoms suggestive of IBD (including Crohn’s disease and ulcerative colitis) should be referred to a gastroenterologist or an appropriate diagnostic pathway, before commencing targeted therapies. Faecal calprotectin can be a useful screening tool for gastrointestinal inflammation with high negative predictive value for IBD [79]. However, levels can be elevated due to NSAID use [80], which should be taken into account when considering gastroenterology referral.
  The prevalence of IBD among people with axSpA is ∼7% [65]. Coexisting IBD can influence multiple aspects of axSpA management, including NSAID use and choice of targeted therapies. IBD severity, relapse frequency and prognosis can vary substantially, and treatment decisions should be made in collaboration with gastroenterology where possible.
  In well-controlled axSpA, mild IBD may be managed by gastroenterology without a change in targeted therapy. Monoclonal TNFi or JAKi are preferred in people with active axSpA and IBD where advanced therapies are indicated. IL-17i should not be commenced in people with active IBD, as it may exacerbate intestinal inflammation (as demonstrated in the clinical trial of secukinumab in Crohn’s disease [81]). Given the relatively limited number of drug classes for axSpA, IL-17i can still be considered in the context of well-controlled IBD when no other options are available, but the balance of risk and benefit in these circumstances should be carefully considered with input from gastroenterology. If IL-17i is used, individuals and their clinicians should regularly monitor for symptoms compatible with IBD.
  In cases where gastroenterology is leading the use of targeted therapies, treatment decisions should consider the fact that some therapies (e.g. ustekinumab, IL-23p19 inhibitors or vedolizumab) lack evidence-base for treatment of axial inflammation.
- Treatment should aim to achieve predefined targets agreed upon with the individual living with axSpA, using individualized therapy adjustments that consider comorbidities and inflammatory disease activity (1B, SoA 99%).
  ASDAS is consistently associated with radiographic progression [31, 32]. However, the TICOSPA trial of treating to target (ASDAS < 2.1) in axSpA did not achieve its primary outcome (≥30% improvement in ASAS Health Index) or the majority of secondary outcomes [82]. Although all outcomes were numerically better in the treat-to-target arm, these differences are difficult to interpret in the context of an open-label trial. While there may be credible explanations for not meeting the primary end point (e.g. usual care being better than expected in academic recruiting centres), there is insufficient evidence to recommend treating to an index-based target. Furthermore, treating to target in the TICOSPA trial involved cycling through a greater number of biologics, albeit without incurring excess healthcare/economic costs. This is an important consideration because, unlike RA and PsA, pharmacological options for axial disease comprise only three classes of targeted therapies and no csDMARDs.
  The GWG recommends that, as with the decisions to initiate or switch therapy, therapeutic targets should be agreed upon with the person with axSpA and not solely based on disease indices. Treatment escalation should consider (1) the overall number of available therapeutic options, (2) adverse prognostic factors for disease progression and treatment response and (3) extent of inflammatory disease activity. Specific comorbidities or complications (e.g. osteoarthritis, fractures and fibromyalgia) and general comorbidity burden are all associated with higher axSpA disease indices independent of inflammatory disease activity, particularly subjective indices such as BASDAI and spinal pain [29, 83].
- Tapering of targeted therapies should be considered for individuals who have achieved sustained remission (1A, SoA 98%).
- Withdrawal of targeted therapies in the context of sustained remission is not recommended (1A, SoA 99%).
  Multiple RCTs, all investigating TNFi, have compared tapering (i.e. dose reduction without complete discontinuation) vs continuing the standard dose among those in sustained remission [84–87]. Overall, TNFi dose reduction was not inferior to continuing standard dose for maintaining response in both ankylosing spondylitis (i.e. radiographic) and non-radiographic axSpA, with a comparable risk of relapse. There was no clear difference in the risk of adverse events, although these trials were not powered to formally compare safety. People with axSpA in sustained remission should be offered therapeutic tapering, with the decision agreed upon between the person with axSpA and the clinician. ‘Sustained remission’ lacks formal definition but can be operationalized as low disease activity or remission for at least 6 months. Tapering is typically implemented by increasing dosing intervals, which (with the exception of certolizumab pegol) is an off-licence use of therapy.
  Withdrawal of targeted therapies among those in sustained remission was investigated in several RCTs of TNFi [84, 85, 88, 89] and one RCT of ixekizumab [90]. Flare rates were significantly higher in withdrawal arms compared with continuing standard dose or tapering arms. For example, 80% of participants who discontinued certolizumab pegol flared during the trial period, and not all were able to regain control [84]. Abnormal inflammatory response to biomechanical strain is considered part of axSpA pathology [91] and, because such stresses and strains are inevitable, flares are likely without ongoing disease suppression. Although complete withdrawal of targeted therapies is not recommended, people with axSpA, informed with these trial data, may nevertheless choose to discontinue therapy and should be supported with access to timely clinical review when needed.

**Table 1.** Summary of the evidence for targeted therapies across extra-musculoskeletal manifestations

Biologic or targeted synthetic DMARD^a^	Review axSpA response (weeks)	Extra-musculoskeletal manifestations			
		Psoriasis	Uveitis	Crohn's disease	Ulcerative colitis
Adalimumab	12	✓	✓	✓✓	✓
Etanercept^b^	12	✓	(∼)	(∼)	(∼)
Certolizumab pegol^c^	12	✓	(✓)	(✓)	(∼)
Golimumab^d^	12	(✓)	(✓)	(∼)	✓✓
Infliximab^e^	12	✓✓	(✓)	✓✓	✓✓
Secukinumab^f^	16	✓✓	(∼)	✗active (∼inactive)	✗active (∼inactive)
Ixekizumab^f^	16-20	✓✓	(∼)	✗active (∼inactive)	✗active (∼inactive)
Bimekizumab^g^	16	✓✓	(∼)	✗active (∼inactive)	✗active (∼inactive)
Tofacitinib^h^	16	(✓)	(∼)	(∼)	✓✓
Upadacitinib^i^	16	(✓)	(∼)	✓✓	✓✓

This table is intended as a quick summary. Always cross-reference with the summary of product characteristics before prescribing. Uveitis data pertain to prevention of acute anterior uveitis incidence or flare. ‘Active/inactive’ refers to disease activity of each extra-musculoskeletal manifestation (EMM). The number of ticks provides a non-quantitative indication of comparative efficacy across each EMM. ✗: not recommended. Parentheses: not currently licenced. ∼: There is a lack of high-quality evidence for efficacy, or there is evidence supporting a lack of efficacy—see footnote for details.

a Information applies to both bio-originator and biosimilar where relevant.

b Etanercept has lower comparative efficacy for all EMMs compared with monoclonal TNFi. The risk of uveitis and IBD onset and flare is greater in etanercept than monoclonal TNFi in observational studies. Etanercept was not superior to placebo in a small RCT of Crohn’s but is unlikely to be directly detrimental to IBD; it could be considered for axSpA, following gastroenterology review.

c Certolizumab pegol is licenced for Crohn’s in the US and Europe but not in the UK. Phase III evidence is lacking for ulcerative colitis (UC), but there are single-arm studies suggesting some effectiveness.

d Golimumab has some evidence of efficacy for psoriasis (in PsA trials) but is not licenced. Phase III evidence is lacking for Crohn’s. Golimumab dosing differs for UC (requires loading dose).

e Infliximab is not licenced for non-radiographic axSpA. Subcutaneous infliximab is licenced for IBD but not for axSpA.

f According to network meta-analysis of RCTs published after the literature search cut-off date, IL-17A inhibitors are likely inferior to monoclonal TNFi, though likely superior to placebo, for uveitis. IL-17A inhibitors are not recommended in active IBD but could be considered for axSpA if IBD is inactive, following gastroenterology review. Dosing of secukinumab (higher dose) and ixekizumab (loading dose) differs in psoriasis. Higher (300 mg) dose of secukinumab is available for ankylosing spondylitis but not non-radiographic axSpA.

g Bimekizumab is superior to secukinumab for cutaneous psoriasis but increases incidence of candidiasis. Bimekizumab dosing differs in psoriasis (higher dose). In *post hoc* analyse of pooled axSpA trial data published after the literature search cut-off date, the bimekizumab arm had lower incidence of uveitis compared with placebo, but it is not currently licenced for uveitis. Evidence for the safety of bimekizumab in IBD is lacking.

h Tofacitinib is not licenced for non-radiographic axSpA. Tofacitinib has phase III evidence of efficacy for psoriasis but is not licenced. JAK inhibitors as a group are likely superior to placebo for uveitis according to network meta-analysis of RCTs. Tofacitinib was not superior to placebo in a phase II trial of Crohn’s.

i Upadacitinib has some evidence of efficacy for psoriasis (in PsA trials) but is not currently licenced. JAK inhibitors as a group are likely superior to placebo for uveitis according to network meta-analysis of RCTs.

**Table 2.** Components and thresholds for the Axial Spondyloarthritis Disease Activity Score (ASDAS)

Derivation	ASDAS	0.12 × back pain + 0.06 × duration of morning stiffness + 0.07 peripheral pain/swelling + 0.11 × patient global + 0.58 × Ln(CRP + 1)
	ASDAS-ESR	0.08 × back pain + 0.07 × duration of morning stiffness + 0.09 × peripheral pain/swelling + 0.11 × patient global + 0.29 × √(ESR)
ASDAS thresholds	High disease activity	≥2.1; >3.5 indicate very high disease activity
	Low disease activity	<2.1, ≥1.3
	Inactive disease	<1.3
	Clinically important improvement	Change of ≥1.1
	Major improvement	Change of ≥2.0

ASDAS based on CRP is preferred. CRP in mg/l and ESR in mm/h. BASDAI questions and patient global are assessed on a numerical rating scale of 0–10. Patient global: ‘How active was your spondylitis on average during the last week?’.

### Scope questions without recommendations

Among the clinical topics identified in the guideline scope, two have not resulted in a recommendation due to lack of any quality evidence and are automatically included as research topics.

The comparative safety of targeted therapies on comorbidities and risk factors has mostly come from non-axSpA (mainly RA and psoriasis) literature. There was insufficient evidence to make axSpA-specific recommendations. Warnings for JAKi extend across drugs in the class and across indications, but risks observed in RA may not be directly generalizable to axSpA populations due to differences in age, sex, the prevalence of cardiovascular risk factors and comorbidities.

There was also insufficient evidence on the clinical effectiveness and safety of combining targeted therapies, including those licenced for EMMs as the index disease. Two clinical trials of combination biologics in IBD have suggested superior efficacy for bowel outcomes [92, 93], but neither has yet reported musculoskeletal outcomes.

## Applicability and utility

This guideline aims to support clinical decision-making to improve quality of care for axSpA. It does not account for individual case complexities that may have greater influence on management decisions, nor does adherence to it constitute defence against claims of negligence. The recommendations are intended to be pragmatic and grounded in the best available evidence but not limited by absence of RCTs. Some recommendations diverge from existing NICE guidelines and drug licencing, which could pose barriers to implementation.

NICE does not currently mandate the use of ASDAS. NICE recommendation to use BASDAI and spinal pain are based on historical practice and data which, in the opinion of the GWG, are superseded by subsequent evidence in support of ASDAS. The overarching aim of treatment includes improving quality of life through (among others) prevention of structural damage—a goal for which BASDAI has lower predictive value. Implementation of ASDAS should be analogous to DAS28 (in terms of CRP timing and use of the patient global) thus familiar to care providers.

Questioning a diagnosis can be both practically and emotionally challenging. However, misdiagnosis is possible, particularly with the drive for earlier diagnosis. Access to specialist musculoskeletal radiologists can be helpful but is not universal. Diagnostic uncertainty, particularly in unexpected clinical trajectories, should be openly discussed within MDTs and with the person with axSpA.

Several recommendations on EMMs emphasize collaborative management with other specialties, which may not always be feasible. This emphasis hopes to provide support for combined services where there is clinical need.

Increasing the dosing interval when implementing drug tapering (except for certolizumab pegol) is outside of licencing authorization, with accompanying implications for the prescriber. However, evidence-based, shared decision-making may be more beneficial for people with axSpA than when they independently adjust dosing intervals without guidance.

The deliberate choice to prioritize non-pharmacological management at the start of the guideline for targeted therapies reflects the advocacy of individuals with lived experience and NASS. Non-pharmacological interventions are typically the first and, in many cases, the only treatment required. At a time when these resources (e.g. community physiotherapy, hydrotherapy, psychology) are strained or diminished, it is critical to highlight their importance in axSpA care. The GWG hopes this emphasis will support business cases for these provisions where clinical need exists.

## Research recommendations

The GWG members proposed research recommendations then voted to select the top 10 listed below.

1. Non-pharmacological management options.
2. Comparisons of targeted therapies in head-to-head clinical trials.
3. Strategies for managing fatigue.
4. Evidence on the sequential use of targeted therapies.
5. Criteria for initiating and predictors of successful therapeutic tapering.
6. Management of difficult-to-treat axSpA.
7. Role of imaging in assessing treatment response.
8. Effective use of patient-initiated follow-up.
9. Comparative safety of targeted therapies in axSpA populations.
10. Safety and efficacy of combining targeted therapies in axSpA with EMMs.

## Audit

A suggested audit tool is available via the BSR website and in Supplementary Data S6, available at *Rheumatology* online. The GWG encourages engagement with the BSR New Early Inflammatory Arthritis Audit.

## Supplementary Material

keaf089_Supplementary_Data

## Data Availability

All data are provided in online Supplementary Materials, available at *Rheumatology* online.
