# Supplementary material for: The 2025 British Society for Rheumatology guideline for the treatment of axial spondyloarthritis with biologic and targeted synthetic DMARDs
Source: Rheumatology (Oxford). 2025 Apr 9;64(6):3242–54. doi: 10.1093/rheumatology/keaf089 (PMC12107049; doi:10.1093/rheumatology/keaf089)
Supplement: keaf089_Supplementary_Data [file keaf089_supplementary_data.zip › keaf089_Supplementary_Data/rhe-24-2017-File002.docx]

**The 2024 BSR guideline for the treatment of axial spondyloarthritis with biologic and targeted synthetic DMARDs**

**Supplementary Data S1. Evidence tables**

**Table of Contents**

[Guideline question 1(Q1) 2](#_Toc178455055)

[Table 1 - Study characteristics of included studies (Q1) 2](#_Toc178455056)

[Table 2 - Efficacy outcomes – Dichotomous data (Q1) 12](#_Toc178455057)

[Table 3 - Efficacy outcomes - Continuous data (Q1) 35](#_Toc178455058)

[Table 4: Safety outcomes (Q1) 50](#_Toc178455059)

[Guideline question 4 (Q4) 78](#_Toc178455060)

[Table 5: Study characteristics of included studies (Q4) 78](#_Toc178455061)

[Table 6: Efficacy outcomes - Binary data (Q4) 80](#_Toc178455062)

[Table 7: Efficacy outcomes - Continuous data (Q4) 84](#_Toc178455063)

[Table 8: Safety outcomes (Q4) 85](#_Toc178455064)

[Guideline question 5 (Q5) 86](#_Toc178455065)

[Table 9: Study characteristics of included studies (Q5) 87](#_Toc178455066)

[Table 10: Efficacy outcomes - Binary data (Q5) 91](#_Toc178455067)

[Table 11: Efficacy outcomes - Continuous data (Q5) 100](#_Toc178455068)

[Table 12: Safety outcomes (Q5) 104](#_Toc178455069)

[Table 13: Observational studies – Study characteristics 111](#_Toc178455070)

[Table 14: Observational studies – Safety outcomes 114](#_Toc178455071)

[References 120](#_Toc178455072)

## Guideline question 1(Q1)

In adults with active axSpA, what is the clinical effectiveness and safety of targeted therapies, compared to each other or placebo?

### Table 1 - Study characteristics of included studies (Q1)

| Interventional drug  Study ID (axSpA type).  ^¥^*Primary outcome (time point in weeks)*  Randomised groups | N | Age, years Mean (SD) | Male  n (%) | HLA-B27-positive n (%) | Disease duration,  years  Mean /*median (SD/**IQR) | Symptom duration, years  Mean (SD) | Previous TNFi, n (%) | Active / History^/ Mixed^^ EMM n (%) | | | Overall Risk of bias  Low Unclear  High |
| --- | --- | --- | --- | --- | --- | --- | --- | --- | --- | --- | --- |
|  |  |  |  |  |  |  |  | IBD | Anterior Uveitis | Psoriasis |  |
| bDMARDs: TNF inhibitors vs Placebo | | | | | | | | | | | |
| Adalimumab (ADA) | | | | | | | | | | | |
| ASIM^1^ (mixed); *BASDAI50 (week 6)* | | | | | | | | | | | |
| Placebo | 24 | 35.1 (7.8) | 10  (42) | 19  (79) | 2.3  (3.5) | 10.4  (7.1) | n/a | 2^  (8) | 7^  (29) | 2^  (8) |  |
| ADA 40 mg, Q2W, SC | 25 | 39.9  (10.8) | 15  (60) | 17  (68) | 4.6  (10.2) | 13.8  (13.4) | n/a | 1^  (4) | 3^  (12) | 0^  (0) |  |
| COAST -V^2^ (r- axSpA); *ASAS40 (week 16)* | | | | | | | | | | | |
| Placebo | 87 | 42·7 (12·0) | 71  (83) | 76  (89) | 6·8  (7·6) | 16·6 (10·1) | n/a | 2^^  (2·3) | 14^^ (16·3) | 8^^  (9·3) |  |
| ADA, 40 mg Q2W, SC | 90 | 41·8 (11·4) | 73  (81) | 82  (91) | 7·5  (7·5) | 15·6  (9·3) | n/a | 1^^  (1·1) | 19 ^^ (21·1) | 6^^  (6·7) |  |
| DANISH^3^ (r-axSpA); *BASDAI50 (week 12)* | | | | | | | | | | | |
| Placebo | 27 | 37.5  (9.4) | 19 (76.0) | 19 (79.2) | NR | 8.2  (8.1) | NR | NR | NR | NR |  |
| ADA, 40 mg, Q2W, SC | 25 | 39.6  (12.4) | 21 (77.8) | 24 (96.0) | NR | 10.9  (10.8) | NR | NR | NR | NR |  |
| Certolizumab (CZP) | | | | | | | | | | | |
| C-axSpAnd^4^ (nr-axSpA); *ASDAS - MI (week 12)* | | | | | | | | | | | |
| Placebo | 158 | 37.4 (10.8) | 76 (48.1) | 132 (83.5) | 4.0  (5.4) | 8.0  (7.5) | 11  (7.0) | NR | 11  (7.0) | NR |  |
| CZP, 400 mg, 3 LD, Q2W  then 200 mg, Q2W | 159 | 37.3 (10.5) | 78 (49.1) | 128 (80.5) | 3.6  (4.8) | 7.8  (7.7) | 7  (4.4) | NR | 6  (3.8) | NR |  |
| Etanercept (ETN) | | | | | | | | | | | |
| EMBARK^5^ (nr-axSpA); *ASAS40 (week 12)* | | | | | | | | | | | |
| Placebo | 109 | 32.0 (7.8) | 62  (56.9) | 83 (76.2) | 2.5  (1.8) | NR | NR | 1^  (0.9) | 9^^  (8.3) | 10^^  (9.2) |  |
| ETN, 50 mg, OW, SC | 106 | 31.9 (7.8) | 68  (64.1) | 71 (67.0) | 2.4  (1.9) | NR | NR | 1^ (0.9) | 8^^  (7.6) | 14^^  (13.2) |  |
| NCT01934933 – Tu 2022^6^ (r- axSpA); *ASAS20 (week 52)* | | | | | | | | | | | |
| Celecoxib 200 mg, BID | 50 | 32.5 (8.2) | 38  (76) | 46  (92) | 9.4  (6.9) | NR | NR | NR | NR | NR |  |
| ETN, 50 mg, OW, SC | 50 | 33.1 (9.0) | 46  (92) | 48  (96) | 8.1  (5.8) | NR | NR | NR | NR | NR |  |
|  | 50 | 31.8 (7.9) | 45  (90) | 47  (94) | 9.2  (5.7) | NR | NR | NR | NR | NR |  |
| $PrevAS^7^ (nr-axSpA); *ASAS20 (week 16)* | | | | | | | | | | | |
| Placebo | 40 | 33  (9) | 16  (40) | 23  (58) | *3.5  (**2 - 8) | NR | n/a | 17^^ (43) | 5^^  (13) | 15^^  (38) |  |
| ETN, 25 mg, BIW, SC | 40 | 36  (10) | 13  (32) | 25  (63) | *5.0  (**2.5 - 14) | NR | n/a | 13^^ (33) | 10^^  (25) | 15^^  (38) |  |
| SPARSE^8^ (nr- axSpA); Change from baseline in *ASAS-NSAID score (week 8)* | | | | | | | | | | | |
| Placebo | 48 | 38.9 (11.4) | 32  (66.7) | 31  (64.6) | 5.5  (7.4) | NR | NR | NR | 3^  (6.3) | 9^  (18.8) |  |
| ETN, 50 mg, OW, SC | 42 | 38.8 (12.3) | 24  (57.1) | 28  (66.7) | 6.0  (9.0) | NR | NR | NR | 5^  (11.9) | 9^  (21.4) |  |
| Golimumab (GOL) | | | | | | | | | | | |
| GO-AHEAD^9^ (nr- axSpA); *ASAS20 (week 16)* | | | | | | | | | | | |
| Placebo | 100 | 31.7  (7.2) | 52 (52.0) | 82 (82.0) | NR | NR | n/a | NR | NR | NR |  |
| GOL, 50 mg, Q4W, SC | 97 | 30.7  (7.1) | 61 (62.2) | 81 (82.7) | NR | NR | n/a | NR | NR | NR |  |
| GO-ALIVE^10^ (r- axSpA); *ASAS20 (week 16)* | | | | | | | | | | | |
| Placebo | 103 | 39.2 (10.8) | 77 (74.8) | 93 (90.3) | 5.5  (5.9) | 11.6  (9.1) | 14  (13.6) | NR | NR | NR |  |
| GOL, 2 mg/kg, Q4W, IV | 105 | 38.4  (10.1) | 86 (81.9) | 94  (89.5) | 5.6  (6.6) | 10.2  (8.9) | 16  (15.2) | NR | NR | NR |  |
| $NCT01212653 – Tam 2014^11^ (r-axSpA); *ASAS20∞ (week 24)* | | | | | | | | | | | |
| Placebo | 21 | 34.2 (10.0) | 19  (90) | NR | *8.0  (**3.0 - 17.0) | NR | NR | NR | 5  (24) | NR |  |
| GOL, 50 mg, Q4W, SC | 20 | 35.6 (9.93) | 18  (90) | NR | *11.0  (**6.0-17.5) | NR | NR | NR | 3  (15) | NR |  |
| bDMARDs: TNF inhibitors – head-to-head | | | | | | | | | | | |
| NCT02489760 – Wei 2020^12^ ^(^r-axSpA)*; BASDAI and ASDAS change from baseline (CfB) (week 8)* | | | | | | | | | | | |
| ADA, 40 mg, Q2W, SC | 9 | 36 (7.6) | 7 (77.8) | (71.4) | NR | NR | NR | NR | NR | NR |  |
| ETN, 25 mg, BIW, SC | 10 | 39.4  (11.2) | 5 (55.6) | (88.9) | NR | NR | NR | NR | NR | NR |  |
| bDMARDs: TNF inhibitors – biosimilars vs originators | | | | | | | | | | | |
| Adalimumab (ADA) | | | | | | | | | | | |
| ChiCTR1900022520 – Su 2020^13^ (r-axSpA); *ASAS20* *(week 24)* | | | | | | | | | | | |
| HS016, 40 mg, Q2W, SC | 416 | 31.5  (7.8) | 359 (86.3) | 380 (91.8) | 6.4  (5.2) | NR | 7  (1.7) | 3  (5.8) | 12  (23.1) | 2  (3.8) |  |
| ADA, 40 mg, Q2W, SC | 232 | 32.1  (8.9) | 204 (87.9) | 212  (91.0) | 6.5  (5.7) | NR | 9  (3.9) | 0 | 6  (11.5) | 1  (1.9) |  |
| CTR20181863 – Li 2022^14^ (r-axSpA); *ASAS20* *(week 24)* | | | | | | | | | | | |
| TQ-Z2301, 40 mg, Q2W, SC | 188 | 31.4  (8.4) | 162 (86.2) | NR | *3.1  (3.7) | NR | NR | NR | NR | NR |  |
| ADA, 40 mg, Q2W, SC | 192 | 31.6  (7.8) | 167 (87) | NR | *3.8  (4.3) | NR | NR | NR | NR | NR |  |
| NCT02893254 – Xu 2019^15^ (r-axSpA); *ASAS20* *(week 24)* | | | | | | | | | | | |
| IBI303, 40 mg, Q2W, SC | 220 | 33·1 (9·2) | 175 (80) | 197  (90) | 4·9  (5·2) | 8·9  (6·2) | NR | NR | NR | NR |  |
| ADA, 40 mg, Q2W, SC | 218 | 31·4 (8·1) | 187 (86) | 202  (93) | 5·0  (4·8) | 8·5  (5·3) | NR | NR | NR | NR |  |
| Etanercept (ETN) Biosimilar – Yisaipu – Injection forms | | | | | | | | | | | |
| NCT04345458- Zhao 2021^16^ (r-axSpA); *ASAS20* *(week 24)* | | | | | | | | | | | |
| Prefilled liquid Yisaipu, 25mg, BIW | 360 | 32.1  (9.9) | 310 (86.1) | 313 (86.9) | *5  (1.6 – 9) | NR | NR | NR | NR | NR |  |
| Prefilled liquid Yisaipu, 50mg, OW | 140 | 34.1 (11) | 120 (85.7) | 118 (84.3) | *5.6  (2 – 9) | NR | NR | NR | NR | NR |  |
| Lyophilised (powered) Yisaipu, 25 mg, BIW | 140 | 32.9 (10.9) | 112 (80.6) | 112 (80.6) | *4.4  (1.9 – 9.4) | NR | NR | NR | NR | NR |  |
| IL-17 inhibitors | | | | | | | | | | | |
| Bimekizumab (BKZ) | | | | | | | | | | | |
| $BE AGILE^17^ (r-axSpA); *ASAS40 (week 12)* | | | | | | | | | | | |
| Placebo | 60 | 39.7  (10.3) | 49 (81.7) | 57  (95.0) | 6.6  (7.2) | 14.1  (8.4) | 7  (11.7) | n/a | n/a | NR |  |
| BKZ, 16 mg, Q4W, SC | 61 | 43.3  (12.6) | 53 (86.9) | 51  (83.6) | 8.0  (9.4) | 16.2 (10.6) | 8  (13.1) | n/a | n/a | NR |  |
| BKZ, 64 mg, Q4W, SC | 61 | 40.4  (10.9) | 52 (85.2) | 56  (91.8) | 7.3  (7.8) | 12.4  (8.3) | 7  (11.5) | n/a | n/a | NR |  |
| BKZ, 160 mg, Q4W, SC | 60 | 42.4  (13.1) | 52 (86.7) | 52  (86.7) | 8.8  (9.2) | 14.8 (10.3) | 7  (11.7) | n/a | n/a | NR |  |
| BKZ, 320 mg, Q4W, SC | 61 | 45.0  (11.4) | 50 (82.0) | 54  (88.5) | 8.8  (8.8) | 15.3 (10.6) | 5  (8.2) | n/a | n/a | NR |  |
| BE MOBILE 1^18^ (nr-axSpA); *ASAS40 (week 16)* | | | | | | | | | | | |
| Placebo | 126 | 39.4  (11.8) | 65 (51.6) | 94  (74.6) | 3.6  (5.4) | 9.0  (9.0) | 17  (13.5) | 1^  (0.8) | 21^ (16.7) | 7^  (5.6) |  |
| BKZ, 160 mg, Q4W, SC | 128 | 39.5  (11.1) | 73 (57.0) | 103  (80.5) | 3.7  (6.2) | 9.1  (8.7) | 10  (7.8) | 3^  (2.3) | 19^ (14.8) | 9^  (7.0) |  |
| BE MOBILE 2^18^ (r-axSpA); *ASAS40 (week 16)* | | | | | | | | | | | |
| Placebo | 111 | 39.2  (12.6) | 80 (72.1) | 93  (83.8) | 5.7  (6.9) | 11.9  (8.6) | 17  (15.3) | 1^  (0.9) | 24^  (21.6) | 10^  (9.0) |  |
| BKZ, 160 mg, Q4W, SC | 221 | 41.0  (12.1) | 160 (72.4) | 191  (86.4) | 6.7  (8.3) | 14.2 (11.0) | 37  (16.7) | 3^  (1.4) | 33^ (14.9) | 16^  (7.2) |  |
| Brodalumab (BRO) | | | | | | | | | | | |
| NCT02985983^19^ (mixed); *ASAS40* *(week 16)* | | | | | | | | | | | |
| Placebo | 79 | 38.3  (10.8) | 61 (77.2) | 65  (82.3) | 6.5  (6.5) | NR | 17  (21.5) | NR | 11 (13.9) | 5  (6.3) |  |
| BRO, 210 mg, 3 QW, Q2W, SC | 80 | 36.6  (11.4) | 66 (82.5) | 68  (85.0) | 7.1  (7.7) | NR | 16  (20.0) | NR | 16 (20.0) | 6  (7.5) |  |
| Ixekizumab (IXE) | | | | | | | | | | | |
| COAST -V^2^ (r- axSpA); bDMARD-naive; *ASAS40 (week 16)* | | | | | | | | | | | |
| Placebo | 87 | 42·7  (12·0) | 71  (83) | 76  (89) | 6·8  (7·6) | 16·6 (10·1) | n/a | 2^^  (2·3) | 14^^ (16·3) | 8^^  (9·3) |  |
| IXE, 80 mg, Q2W, SC | 83 | 41·3  (11·2) | 64  (77) | 75  (90) | 8·2  (9·0) | 15·8 (10·6) | n/a | 2^^  (2·4) | 21^^ (25·3) | 3^^  (3·6) |  |
| IXE, 80 mg, Q4W, SC | 81 | 41·0  (12·1) | 68  (84) | 75  (93) | 8·3  (9·6) | 15·8 (11·2) | n/a | 1^^  (1·2) | 17^^ (21·0) | 4^^  (4·9) |  |
| COAST -W^20 ,21^ (r- axSpA); TNFi-experienced; *ASAS40 (week 16)* | | | | | | | | | | | |
| Placebo | 104 | 46.6  (12.7) | 87 (83.7) | 86  (82.7) | 13.0  (10.5) | 19.9 (11.6) | 104  (100) | 3  (2.9) | 27 (26.0) | 15  (14.4) |  |
| IXE, 80 mg, Q2W, SC | 98 | 44.2  (10.8) | 75 (76.5) | 80  (81.6) | 11.7  (8.8) | 16.5  (9.6) | 97 | 3  (3.1) | 26 (26.5) | 9  (9.2) |  |
| IXE, 80 mg, Q4W, SC | 114 | 47.4  (13.4) | 91 (79.8) | 91  (79.8) | 10.1  (7.8) | 18.8 (11.6) | 114  (100) | 2  (1.8) | 21 (18.4) | 15  (13.2) |  |
| COAST - X^22,23^ (nr-axSpA); *ASAS40 (week 16)* | | | | | | | | | | | |
| Placebo | 105 | 39·9  (12·4) | 44  (42) | 77  (74) | 87  (83.7) | 10·1  (8·3) | n/a | n/a | n/a | n/a |  |
| IXE, 80 mg, Q2W, SC | 102 | 40·0  (12·0) | 49  (48) | 73  (72) | 75  (76.5) | 10·6 (10·1) | n/a | n/a | n/a | n/a |  |
| IXE, 80 mg, Q4W, SC | 96 | 40·9  (14·5) | 50  (52) | 71  (75) | 91  (79.8) | 11·3 (10·7) | n/a | n/a | n/a | n/a |  |
| Netakimab (NTK) | | | | | | | | | | | |
| $AILAS^24^ (r- axSpA ); *ASAS20* *(week 16)* | | | | | | | | | | | |
| Placebo | 22 | *41.0  (32.0-47.0) | 15 (68.2) | NR | *2.3  (0.8-4) | NR | 4  (18.2) | NR | NR | NR |  |
| NTK, 40 mg, 3 QW, Q2W, SC | 22 | *40.0  (33.0-44.0) | 5 (22.7) | NR | *2.3  (0.11-6.3) | NR | 4  (18.2) | NR | NR | NR |  |
| NTK, 80 mg, 3 QW, Q2W, SC | 23 | *34.0  (31.0-36.0) | 3 (13.6) | NR | *3.2  (1.8-4.8) | NR | 2  (9.1) | NR | NR | NR |  |
| NTK, 120 mg, 3 QW, Q2W, SC | 22 | *38.0  (35.0-44.0) | 0 | NR | *3.11  (1.1-8) | NR | 3  (13.6) | NR | NR | NR |  |
| Secukinumab (SEC) | | | | | | | | | | | |
| ACHILLES^25^ (mixed)^€^; *ASAS20 (week 24)* | | | | | | | | | | | |
| Placebo | 38 | NR**^€^** | NR**^€^** | 21  (55.3) | 4.8  (6.2) | NR**^€^** | NR**^€^** | NR | NR | NR |  |
| SEC, 150 mg, Q4W, SC | 38 | NR**^€^** | NR**^€^** | 27  (71.1) | 4.2  (5.7) | NR**^€^** | NR**^€^** | NR | NR | NR |  |
| MEASURE 1^26-30^ (r- axSpA); *ASAS20 (week 16)* | | | | | | | | | | | |
| Placebo | 122 | 43.1  (12.4) | 85  (70) | 90  (74) | 8.3  (8.9) | NR | 89  (73) | 2  (2) | 22  (18) | 7  (6) |  |
| SEC, 75 mg, Q4W, SC | 124 | 42.3  (13.2) | 88  (71) | 99  (80) | 7.9  (9.7) | NR | 90  (73) | 6  (5) | 25  (20) | 4  (3) |  |
| SEC, 150 mg, Q4W, SC | 125 | 40.1  (11.6) | 84  (67) | 86  (69) | 6.5  (6.9) | NR | 92  (74) | 2  (2) | 15  (12) | 8  (6) |  |
| MEASURE 2a*^31-33^ (r- axSpA); *ASAS20 (week 16) ^^^* | | | | | | | | | | | |
| Placebo | 45 | 43.5  (13.3) | 35 | 33  (75.0) | 3.9  (6.2) | NR | n/a | 2^  (3) | 3^  (18) | 8^  (11) |  |
| SEC, 75 mg, Q4W, SC | 45 | 43.9  (14.1) | 31 | 32  (71.1) | 3.7  (5.7) | NR | n/a | 0^ | 10^  (14) | 6^  (8) |  |
| SEC, 150 mg, Q4W, SC | 44 | 43.7  (12.9) | 26 | 33  (75.0) | 6.1  (8.6) | NR | n/a | 3^  (4) | 11^  (15) | 7^  (6) |  |
| MEASURE 2b*^31-33^ (r- axSpA); *ASAS20 (week 16) ^^^* | | | | | | | | | | | |
| Placebo | 29 | 43.8  (13.2) | 21 | 24  (85.7) | 10.2  (11.0) | NR | 29 | n/a*^^^* | n/a*^^^* | n/a*^^^* |  |
| SEC, 75 mg, Q4W, SC | 28 | 45.2  (11.3) | 20 | 21  (75.0) | 7.7  (9.0) | NR | 28 | n/a*^^^* | n/a*^^^* | n/a*^^^* |  |
| SEC, 150 mg, Q4W, SC | 28 | 39.3  (11.6) | 20 | 24  (85.7) | 8.5  (7.6) | NR | 28 | n/a*^^^* | n/a*^^^* | n/a*^^^* |  |
| MEASURE 3 ^34, 35^ (r- axSpA); *ASAS20 (week 16)* | | | | | | | | | | | |
| Placebo | 76 | 42.7  (11.4) | 40 (52.6) | 53  (69.7) | 5.2  (6.4) | NR | 17  (32.4) | NR | NR | NR |  |
| SEC, 150 mg, Q4W, IV | 74 | 42.9  (11.1) | 46 (62.2) | 52  (70.3) | 6.0  (7.2) | NR | 17  (33) | NR | NR | NR |  |
| SEC, 300 mg, Q4W, IV | 76 | 42.1  (11.8) | 50 (65.8) | 56  (73.7) | 5.3  (7.3) | NR | 19  (25) | NR | NR | NR |  |
| MEASURE 4^36^ (r- axSpA); (*combined data reported for 4a and 4b*); *ASAS20 (week 16)* | | | | | | | | | | | |
| Placebo | 117 | 43.4 (12.5) | 76  (65.0) | 93  (79.5) | 7.1  (9.2) | NR | 34  (20.5) | 0^ | 27^  (23.1) | NR |  |
| SEC, 150 mg, LD, 4 QW, Q4W, SC | 116 | 44.5 (11.6) | 81  (69.8) | 100  (86.2) | 8.4  (10.8) | NR | 31  (26.7) | 2^  (1.7) | 23^  (19.8) | NR |  |
| SEC, 150 mg, NL, 4 QW, Q4W, SC | 117 | 41.2 (11.1) | 83  (70.9) | 99  (84.6) | 6.5  (7.6) | NR | 32  (27.4) | 4^  (3.4) | 21^  (17.9) | NR |  |
| MEASURE 5^37^ (r- axSpA); *ASAS20 (week 16)* | | | | | | | | | | | |
| Placebo | 153 | 33.0 (10) | 132 (86.3) | 142  (92.8) | 5.3  (6) | NR | 31  (20.3) | NR | NR | NR |  |
| SEC, 150 mg, 4 QW, Q4W, SC | 305 | 35.1 (10.4) | 252 (82.6) | 276  (90.5) | 5.7  (6.4) | NR | 65  (21.3) | NR | NR | NR |  |
| PREVENT ^38^(nr-axSpA); *ASAS40* *(week 16)* | | | | | | | | | | | |
| Placebo | 186 | 39.30 (11.5) | 91 (48.9) | 129  (69.4) | 3  (5) | 8.4  (8.3) | 15  (8.1) | 5^  (2.7) | 18^  (9.7) | NR |  |
| SEC, 150 mg, LD, 4QW, Q4W, SC | 185 | 39.10 (11.5) | 80 (43.2) | 136  (73.5) | 2.8  (4.6) | 8.7  (9.3) | 21  (11.4) | 2^  (1.1) | 21^  (11.4) | NR |  |
| SEC,150 mg, NL, 4QW, Q4W, SC | 184 | 39.80 (11.7) | 84 (45.7) | 117  (63.6) | 2.12  (3.1) | 8.57  (8.6) | 18  (9.8) | 3^  (1.6) | 26^  (14.1) | NR |  |
| IL-6 inhibitors | | | | | | | | | | | |
| Sarilumab (SAR) | | | | | | | | | | | |
| $ALIGN^39^ (mixed); *ASAS20 (week 12)* | | | | | | | | | | | |
| Placebo | 50 | 40.3 (11.7) | 38 (76.0) | 37  (74) | 9.5  (8.3) | NR | NR | NR | NR | NR |  |
| Sarilumab, 100 mg, Q2W, SC | 49 | 42.4 (10.8) | 30 (61.2) | 39  (78.7) | 8.5  (10.3) | NR | NR | NR | NR | NR |  |
| Sarilumab, 150 mg, Q2W, SC | 50 | 43.0 (11.3) | 34 (68.0) | 38  (76) | 8.6  (10.6) | NR | NR | NR | NR | NR |  |
| Sarilumab, 100 mg, QW, SC | 52 | 40.4 (11.5) | 37 (71.2) | 41  (78.8) | 7.1  (8) | NR | NR | NR | NR | NR |  |
| Sarilumab, 200 mg, Q2W, SC | 50 | 37.2 (10.4) | 40 (80.0) | 39  (78) | 7.1  (7.1) | NR | NR | NR | NR | NR |  |
| Sarilumab, 150 mg, QW, SC | 50 | 41.1 (11.1) | 39 (78.0) | 41  (81.6) | 5.6  (5.3) | NR | NR | NR | NR | NR |  |
| IL-23 inhibitors | | | | | | | | | | | |
| $NCT02047110^40^ (r- axSpA); *ASAS40 (week 12) ^§1^*trial period – 24 weeks | | | | | | | | | | | |
| Placebo | 40 | 37.6 (11.0) | 25  (63) | 26  (65) | 8.1  (8.2) | NR | n/a | n/a | n/a | n/a |  |
| Risankizumab, 18 mg, SD, SC | 40 | 38.0 (11.1) | 28  (70) | 30  (75) | 7.4  (8.2) | NR | n/a | n/a | n/a | n/a |  |
| Risankizumab, 90 mg^ν^, SC | 39 | 39.5 (10.8) | 30  (77) | 30  (77) | 6.6  (8.8) | NR | n/a | n/a | n/a | n/a |  |
| ^Ə1^ Risankizumab,180 mg^ν^, SC | 40 | 40.6 (11.9) | 30  (75) | 34  (85) | 10.2  (9.5) | NR | n/a | n/a | n/a | n/a |  |
| JAK inhibitors | | | | | | | | | | | |
| Filgotinib | | | | | | | | | | | |
| ^$^TORTUGA^41,42^ (r-axSpA); *ASDAS change from baseline (CfB) (week 12)* | | | | | | | | | | | |
| Placebo | 58 | 42 (9·0) | 41  (71) | 51  (88) | 8  (7·6) | NR | 7  (12) | NR | NR | NR |  |
| Filgotinib 200mg, OD, oral | 58 | 41 (11·6) | 45  (78) | 51  (88) | 6  (5·5) | NR | 4  (7) | NR | NR | NR |  |
| Tofacitinib | | | | | | | | | | | |
| ^$^NCT01786668^43^ (r-axSpA); *ASAS20 (week 12)* | | | | | | | | | | | |
| Placebo | 51 | 41.9 (12.9) | 32 (62.7) | 44 (86.3) | *3.0 | NR | NR | 1  (2.0) | 7  (13.7) | 2  (3.9) |  |
| Tofacitinib 2 mg, BID, oral | 52 | 41.8 (12.3) | 34 (65.4) | 44 (84.6) | *4.1 | NR | NR | 0 | 13  (25.0) | 1  (1.9) |  |
| Tofacitinib 5mg, BID, oral | 52 | 41.2 (10.3) | 39  (75) | 44 (84.6) | *3.5 | NR | NR | 3  (5.8) | 12  (23.1) | 2  (3.8) |  |
| Tofacitinib 10 mg, BID, oral | 52 | 41.6 (12.2) | 38 (73.1) | 49 (94.2) | *1.5 | NR | NR | 0 | 6  (11.5) | 1  (1.9) |  |
| NCT03502616^44,45^ (r-axSpA); *ASAS20 (week 16)* | | | | | | | | | | | |
| Placebo | 136 | 40.0 (11.1) | 108 (79.4) | 118 (86.8) | 6.8  (6.9) | 12.9  (9.5) | 30 | 1  (0.7) | 5  (3.7) | 2  (1.5) |  |
| Tofacitinib 5mg, BID, oral | 133 | 42.2 (11.9) | 116 (87.2) | 117 (88.0) | 8.9  (9.1) | 14.2  (9.8) | 29 | 1  (0.8) | 6  (4.5) | 2  (1.5) |  |
| Upadacitinib | | | | | | | | | | | |
| ^$^SELECT-AXIS 1^46, 47^ (r-axSpA); *ASAS40 (week 14)* | | | | | | | | | | | |
| Placebo | 94 | 43·7 (12·1) | 69  (73) | 73  (78) | 6·0  (6·8) | 14·0  (9·9) | n/a | n/a | n/a | n/a |  |
| Upadacitinib 15mg OD, oral | 93 | 47·0 (12·8) | 63  (68) | 70  (75) | 7·8  (10·6) | 14·8  (11·6) | n/a | n/a | n/a | n/a |  |
| SELECT-AXIS 2 ^48,49^ (nr- axSpA); *ASAS40 (week 14)* | | | | | | | | | | | |
| Placebo | 157 | 42·5 (12·4) | 63  (40) | 93  (60) | 4·4  (5·8) | 9·2  (8·1) | 54  (34) | 6  (4) | 11  (7) | 4  (3) |  |
| Upadacitinib 15mg OD, oral | 156 | 41·6 (12·0) | 67  (43) | 90  (59) | 4·5  (5·5) | 9·0  (7·9) | 49  (31) | 3  (2) | 12  (8) | 4  (3) |  |
| PDE4i | | | | | | | | | | | |
| Apremilast | | | | | | | | | | | |
| $POSTURE^50^ (r-axSpA); *ASAS20 (week 16)* ^§2^trial period – 24 weeks | | | | | | | | | | | |
| Placebo | 164 | 44.0 (12.9) | 124  (75.6) | 140 (85.4) | 10.4  (10.4) | NR | n/a | n/a | n/a | n/a |  |
| Apremilast, 20 mg, BID, Oral | 163 | 45.2 (11.9) | 121  (74.2) | 136 (83.4) | 11.1 (11.3) | NR | n/a | n/a | n/a | n/a |  |
| ^Ə2^ Apremilast, 30 mg, BID, Oral | 163 | 44.8 (11.8) | 107  (65.6) | 131 (80.4) | 10.3  (9.9) | NR | n/a | n/a | n/a | n/a |  |

SD: Standard deviation; IQR: Inter quartile range; EMM: Extra musculoskeletal manifestations; ^ EMM – history of events; ^^ EMM includes both active and history events; *^^^*history of EMMs includes both a* and b* groups; IBD: Inflammatory bowel disease; $phase 2 trial; **^¥^**Primary outcome*; ∞* primary outcome is progression of subclinical atherosclerosis at 12 months, however, those failing to achieve ASAS20 at 6 months entered the open-label GOL treatment; ^40, 50^At Week 16, patients not achieving ASAS20 switched to **^Ə1^** and **^Ə2^** in ***^§1^*** and **^§2^** respectively; a*: TNFi – naïve group; b*: TNFi – Inadequate response group. Studies are grouped into a and b only if the outcome is reported by TNFi status; r: radiographic axSpA; nr: non-radiographic axSpA; **^€^**Study includes both PsA and axSpA patients and data not reported by condition; LD: loading dose; NL: No loading dose; BID – twice a day; BIW – twice weekly, OW – Once weekly; Q2W - Once every two weeks; Q4W - Once every four weeks; OD: once daily; SD: single dose; **^ν^**day 1, and at wks - 8, 16 and 24; SC: Subcutaneous; IV: Intravenous; NR: not reported; n/a: not applicable if the study reported the variable as part of the exclusion criteria; Risk of bias: Green – Low, Amber – Unclear, Red – High.

### Table 2 - Efficacy outcomes – Dichotomous data (Q1)

| **Study ID**  *Population*  *Time point* | | **Treatment arm** | | **Response, n (%)** | | | **RR**  **(95% CI)** | | **NNT** | | **Risk of bias** | **Quality of evidence** |
| --- | --- | --- | --- | --- | --- | --- | --- | --- | --- | --- | --- | --- |
|  |  |  |  | **Intervention** | **Control** | |  |  |  |  |  |  |
| **bDMARDs: TNF inhibitors vs Placebo** | | | | | | | | | | | | |
| **ASAS20** | | | | | | | | | | | | |
| ASIM^1^  mixed *-axSpA*  *week 6* | | ADA 40 mg, Q2W, SC | | 13  (52) | 4  (17) | | 3.1  (1.2, 8.2) | | 2.8 | |  | **Moderate confidence:** TNFi reduce disease activity in people with AxSPA (increase in proportion meeting ASAS20 responder criteria)  9 RCTs, 6 considered low risk of bias.  Findings are consistently in favour of TNFi compared to placebo, but effect sizes are consistently larger for ADA and GOL than for ETN. Effects appear larger for radiographic than for non-radiographic presentation of AxSpa, but these differences are unlikely to be statistically significant. There is some heterogeneity across studies in terms of %male and duration (Downgraded for inconsistency)  4 RCTs include at least 50 pts per arm (total sample size across all arms: n= 1,157). Confidence intervals are wide, but only in small studies (effects not statistically significant). |
| COAST -V^2^  *r-axSpA*  *week 16* | | ADA 40 mg, Q2W, SC | | 53  (59) | 35  (40) | | 1.5  (1.1, 2.0) | | 5.3 | |  |  |
| EMBARK^5^  *nr-axSpA*  *week 12* | | ETN, 50 mg, OW, SC | | 55  (52.4) | 39  (36.1) | | 1.45  (1.1, 2.0) | | 6.2 | |  |  |
| **^¥^**NCT01934933 ^6^  *r-axSpA*  *week 52* | | ETN, 50 mg, OW, SC | | 29  (58) | 22  (44) | | 1.32  (0.9, 2.0) | | 7.1 | |  |  |
| **^¥^**PrevAS^7^  *nr-axSpA*  *week 16* | | ETN, 25 mg, BIW, SC | | 6  (17) | 4  (11) | | 1.50  (0.5, 4.9) | | 18 | |  |  |
| SPARSE^8^  *r-axSpA*  *week 8* | | ETN, 50 mg, OW, SC | | 16  (44.4) | 10  (23.8) | | 1.8  (0.9, 3.6) | | 5.8 | |  |  |
| **^¥^**GO-AHEAD^9^  *nr-axSpA*  *week 16* | | GOL, 50 mg, Q4W, SC | | 69  (71.1) | 40  (40) | | 1.8  (1.4, 2.3) | | 3.2 | |  |  |
| **^¥^**GO-ALIVE^10^  *r-axSpA*  *week 16* | | GOL, 2 mg**/**kg, Q4W, IV | | 77  (73.3) | 27  (26.2) | | 2.8  (2.0, 4.0) | | 2.1 | |  |  |
| **^¥^**NCT01212653^11^  *r-axSpA*  *week 24* | | GOL, 50 mg, Q4W, SC | | 11  (55) | 3  (14) | | 3.9  (1.3, 11.8) | | 2.5 | |  |  |
| **ASAS40** | | | | | | | | | | | | |
| ASIM^1^  mixed *-axSpA*  *week 6* | | ADA 40 mg, Q2W, SC | | 12  (48) | 1  (4) | | 11.5  (1.6, 81.9) | | 2.3 | |  | **Moderate confidence:** TNFi reduce disease activity in people with AxSPA (increase in proportion meeting ASAS40 responder criteria)  9 RCTs, 7 considered low risk of bias.  Findings are in favour of TNFi compared to placebo in 7 out of 8 studies. Effect sizes are consistently larger for ADA, CZP and GOL than for ETN. Effects appear larger for radiographic than for non-radiographic presentation of AxSpa, but these differences are unlikely to be statistically significant. There is some heterogeneity across studies in terms of %male and duration. (Downgraded for inconsistency)  6 RCTs include at least 50 pts per arm (total sample size across all arms: n= 1,433). Confidence intervals are wide, but only in small studies (effects not statistically significant). |
| **^¥^**COAST -V^2^  *r-axSpA*  *week 16* | | ADA**,** 40 mg, Q2W, SC | | 32  (36) | 16  (18) | | 1.9  (1.2, 3.3) | | 5.8 | |  |  |
| C-axSpAnd^4^  *nr-axSpA*  *week 12* | | CZP, 400 mg, 3 LD**,** Q2W  then 200 mg**,** Q2W | | 76  (47.8) | 18  (11.4) | | 4.2  (2.6, 6.7) | | 2.7 | |  |  |
| **^¥^**EMBARK^5^  *nr-axSpA*  *week 12* | | ETN, 50 mg, OW, SC | | 35  (33.3) | 16  (14.8) | | 2.0  (1.2, 3.3) | | 6.0 | |  |  |
| NCT01934933^6^  *r-axSpA*  *week 52* | | ETN, 50 mg, OW, SC | | 28  (56) | 18  (36) | | 1.6  (1.0, 2.4) | | 5.0 | |  |  |
| PrevAS^7^  *nr-axSpA*  *week 16* | | ETN, 25 mg, BIW, SC | | 3  (8) | 3  (8) | | 1.0  (0.2, 4.6) | | n/a | |  |  |
| SPARSE^8^  *r-axSpA*  *week 8* | | ETN, 50 mg, OW, SC | | 16  (44.4) | 9  (21.4) | | 2.0  (1.0, 4.1) | | 5.2 | |  |  |
| GO-AHEAD^9^  *nr-axSpA*  *week 16* | | GOL, 50 mg, Q4W, SC | | 55  (56.7) | 23  (23) | | 2.5  (1.7, 3.7) | | 3.0 | |  |  |
| GO-ALIVE^10^  *r-axSpA*  *week 16* | | GOL, 2 mg/kg, Q4W, IV | | 50  (47.6) | 9  (8.7) | | 5.5  (2.8, 10.5) | | 2.6 | |  |  |
| **ASDAS – Clinically Important Improvement (CII): Δ ≥ - 1.1** | | | | | | | | | | | | |
| COAST -V^2^  *r-axSpA*  *week 16* | | ADA**,** 40 mg, Q2W, SC | | 48  (55.8) | 20  (20.3) | | 2.3  (1.5, 3.6) | | 3.3 | |  | **Moderate confidence:** TNFi reduce disease activity in people with AxSPA (increase in proportion showing clinically important improvement in ASDAS score)  3 RCTs, all considered low risk of bias.  Findings are in favour of TNFi compared to placebo in all studies and appear consistent in terms of relative risk.  2 RCTs include at least 50 pts per arm (total sample size across all arms: n= 465). Confidence interval is very wide for the small RCT. (Downgraded for precision). |
| PrevAS^7^  *nr-axSpA*  *week 16* | | ETN, 25 mg, BIW, SC | | 7  (22) | 2  (7) | | 3.3  (0.7, 14.6) | | 6.6 | |  |  |
| GO-ALIVE^10^  *r-axSpA*  *week 16* | | GOL, 2 mg/kg, Q4W, IV | | 87  (82.7) | 23  (22.5) | | 3.7  (2.6, 5.4) | | 1.7 | |  |  |
| **ASDAS – Major Improvement (MI): Δ ≥ -2.0** | | | | | | | | | | | | |
| COAST -V^2^  *r-axSpA*  *week 16* | | ADA**,** 40 mg, Q2W, SC | | 21  (24.4) | 4  (4.7) | | 5.3  (1.9, 14.7) | | 5.1 | |  | **Moderate confidence**: TNFi reduce disease activity in people with AxSPA (increase in proportion showing major important improvement in ASDAS score)  4 RCTs, three considered low risk of bias.  Findings are in favour of TNFi compared to placebo in three studies. Direction is reversed in fourth study, but likely related to very small event rate.  2 RCTs includes at least 50 pts per arm (total sample size across all arms: n= 626). Confidence intervals are very wide, due to small sample size and/or low event rate. (Downgraded for precision). |
| DANISH^3^  r-axSpA  *week 12* | | ADA 40 mg, Q2W, SC | | 11  (48.1) | 2  (7.4) | | 6.0  (1.5, 24.2) | | 2.7 | |  |  |
| **^¥^**C-axSpAnd^4^  *nr-axSpA*  *week 12* | | CZP, 400 mg, 3 LD**,** Q2W  then 200 mg**,** Q2W | | 56  (35.2) | 10  (6.3) | | 5.6  (3.0, 10.5) | | 3.5 | |  |  |
| PrevAS^7^  *nr-axSpA*  *week 16* | | ETN, 25 mg, BIW, SC | | 1  (3) | 2  (7) | | 0.5  (0.0, 4.9) | | -28.2 | |  |  |
| **ASDAS: Inactive disease (<1.3)** | | | | | | | | | | | | |
| COAST -V^2^  *r-axSpA*  *week 16* | | ADA**,** 40 mg, Q2W, SC | | 14  (16) | 2  (2) | | 6.8  (1.6, 28.9) | | 7.5 | |  | **Moderate confidence**: TNFi reduce disease activity in people with AxSPA (increase in proportion showing inactive disease based on ASDAS score)  6 RCTs, all considered low risk of bias.  Findings are in favour of TNFi compared to placebo in all studies, although the difference is small in two ETN studies. Effect sizes appear larger for ADA and GOL compared to ETN.  4 RCTs include at least 50 pts per arm (total sample size across all arms: n= 967). Confidence intervals are wide in small studies, possibly due to low event rates; studies not powered for this outcome. (Downgraded for precision). |
| EMBARK^5^  *nr-axSpA*  *week 12* | | ETN, 50 mg, OW, SC | | 42  (40) | 19  (17.4) | | 2.3  (1.4, 3.6) | | 4.5 | |  |  |
| PrevAS^7^  *nr-axSpA*  *week 16* | | ETN, 25 mg, BIW, SC | | 7  (21) | 6  (17) | | 1.2  (0.5, 3.3) | | 25.5 | |  |  |
| SPARSE^8^  *r-axSpA*  *week 8* | | ETN, 50 mg, OW, SC | | 8  (19.5) | 6  (13.3) | | 1.5  (0.6, 4.0) | | 15.3 | |  |  |
| GO-AHEAD^9^  *nr-axSpA*  *week 16* | | GOL, 50 mg, Q4W, SC | | 29  (29.9) | 12  (12) | | 2.5  (1.4, 4.6) | | 5.6 | |  |  |
| GO-ALIVE^10^  *r-axSpA*  *week 16* | | GOL, 2 mg/kg, Q4W, IV | | 21  (20) | 3  (2.9) | | 2.6  (1.2, 5.6) | | 8.2 | |  |  |
| **ASDAS: Low disease activity (<2.1)** | | | | | | | | | | | | |
| COAST -V^2^  *r-axSpA*  *week 16* | | ADA**,** 40 mg, Q2W, SC | | 34  (38) | 11  (13) | | 3.0  (1.6, 5.5) | | 4 | |  | **Moderate confidence**: TNFi reduce disease activity in people with AxSPA (increase in proportion showing low disease activity based on ASDAS score)  4 RCTs, all considered low risk of bias.  Findings are in favour of TNFi compared to placebo in three studies, with consistent relative risk. One (small) study does not find a benefit for ETN.  2 RCTs include at least 50 pts per arm (total sample size across all arms: n= 544). Confidence intervals are wide, due to small sample size. (Downgraded for precision). |
| PrevAS^7^  *nr-axSpA*  *week 16* | | ETN, 25 mg, BIW, SC | | 15  (44) | 15  (42) | | 1.1  (0.6, 1.8) | | 40.8 | |  |  |
| SPARSE^8^  *r-axSpA*  *week 8* | | ETN, 50 mg, OW, SC | | 15  (36.6) | 6  (13.3) | | 2.9  (1.2, 6.7) | | 4.3 | |  |  |
| GO-AHEAD^9^  *nr-axSpA*  *week 16* | | GOL, 50 mg, Q4W, SC | | 56  (57.5) | 24  (24) | | 2.4  (1.6, 3.6) | | 3.0 | |  |  |
| **BASDAI50** | | | | | | | | | | | | |
| **^¥^**ASIM^1^  mixed *-axSpA*  *week 6* | | ADA 40 mg, Q2W, SC | | 13  (52) | 3  (13) | | 4.2  (1.4, 12.8) | | 2.5 | |  | **High confidence**: TNFi reduce disease activity in people with AxSPA (increase in proportion meeting BASDAI responder criteria)  7 RCTs, 5 considered low risk of bias.  Findings are in favour of TNFi compared to placebo in all studies, with effects sizes fairly consistent across types of TNFi.  4 RCTs include at least 50 pts per arm (total sample size across all arms: n= 988). Confidence intervals is wide in one study, mainly related to small event rate. |
| COAST -V^2^  *r-axSpA*  *week 16* | | ADA**,** 40 mg, Q2W, SC | | 29  (32) | 15  (17) | | 1.9  (1.1, 3.2) | | 6.7 | |  |  |
| **^¥^**DANISH^3^  r-axSpA  *week 12* | | ADA 40 mg, Q2W, SC | | 13  (52) | 6  (22.2) | | 2.3  (1.1, 5.2) | | 3.4 | |  |  |
| EMBARK^5^  *nr-axSpA*  *week 12* | | ETN, 50 mg, OW, SC | | 46  (43.8) | 26  (23.9) | | 1.8  (1.2, 2.7) | | 5.1 | |  |  |
| SPARSE^8^  *r-axSpA*  *week 8* | | ETN, 50 mg, OW, SC | | 16  (39) | 8  (17.8) | | 2.3  (1.1, 4.8) | | 4.7 | |  |  |
| GO-AHEAD^9^  *nr-axSpA*  *week 16* | | GOL, 50 mg, Q4W, SC | | 57  (57.7) | 30 (30.0) | | 2.0  (1.4, 2.8) | | 3.5 | |  |  |
| GO-ALIVE^10^  *r-axSpA*  *week 16* | | GOL, 2 mg/kg, Q4W, IV | | 43  (41) | 15  (14.6) | | 2.8  (1.7, 4.7) | | 3.8 | |  |  |
| **bDMARDs: TNF inhibitors – head-to-head** | | | | | | | | | | | | |
| **ASAS20** | | | | | | | | | | | | |
| NCT02489760^12^  *r-axSpA*  *week 8* | | G1: ADA, 40 mg, Q2W, SC  G2: ETN, 25 mg, BIW, SC | | 4  (44) | 3  (33) | | 1.5  (0.5, 4.9) | | 9.1 | |  | **Very low confidence:**  One very small trial (n= 19, high risk of bias) shows no significant difference between ADA and ETN for disease activity (ASAS20 responder criteria). (Downgraded for study limitations, inconsistency, precision). |
| **ASAS40** | | | | | | | | | | | | |
| NCT02489760^12^  *r-axSpA*  *week 8* | | G1: ADA, 40 mg, Q2W, SC  G2: ETN, 25 mg, BIW, SC | | 2  (22) | 2  (22) | | 1.1  (0.2, 6.3) | | n/a | |  | **Very low confidence:**  One very small trial (n= 19, high risk of bias) shows no significant difference between ADA and ETN for disease activity (ASAS40 responder criteria). (Downgraded for study limitations, inconsistency, precision). |
| **ASDAS: Inactive disease (<1.3)** | | | | | | | | | | | | |
| NCT02489760^12^  *r-axSpA*  *week 8* | | G1: ADA, 40 mg, Q2W, SC  G2: ETN, 25 mg, BIW, SC | | 5  (56) | 6  (67) | | 0.9  (0.4, 2.0) | | -22.5 | |  | **Very low confidence:**  One very small trial (n= 19, high risk of bias) shows no significant difference between ADA and ETN for disease activity (ASDAS inactive disease). (Downgraded for study limitations, inconsistency, precision). |
| **ASDAS: Low disease activity (<2.1)** | | | | | | | | | | | | |
| NCT02489760^12^  *r-axSpA*  *week 8* | | G1: ADA, 40 mg, Q2W, SC  G2: ETN, 25 mg, BIW, SC | | 7  (78) | 9  (100) | | 0.8  (0.5, 1.2) | | -8.2 | |  | **Very low confidence**:  One very small trial (n= 19, high risk of bias) shows no significant difference between ADA and ETN for disease activity (ASDAS low disease activity).  (Downgraded for study limitations, inconsistency, precision). |
| **bDMARDs: TNF inhibitors – biosimilars vs originators** | | | | | | | | | | | | |
| **^¥^ASAS20** | | | | | | | | | | | | |
| ChiCTR1900022520^13^  r-axSpA  *week 24* | | HS016, 40 mg, Q2W, SC | | 364  (87.5) | 209  (90.1) | | 1.0  (0.9, 1.0) | | -38.7 | |  | **High confidence:** biosimilars of ADA have similar effect on disease activity in AxSPA (ASAS20 responder criteria)  3 RCTs, 2 with low risk of bias.  Findings consistently show a similar proportion of pts meeting ASAS20 responder criteria for biosimilars and ADA.  All 3 RCTs include more than 100 pts per arm (larger sample size needed for non-superiority or equivalence (total sample size across arms: n= 1,466). |
| CTR20181863^14^  r-axSpA  *week 24* | | TQ-Z2301, 40 mg, Q2W, SC | | 163  (86.7) | 155  (80.7) | | 1.1  (1.0, 1.2) | | 16.7 | |  |  |
| NCT02893254^15^  *r-axSpA*  *week 24* | | IBI303, 40 mg, Q2W, SC | | 165  (75) | 158  (72) | | 1.0  (0.9, 1.2) | | 39.6 | |  |  |
| NCT04345458 ^16^  *r-axSpA*  *week 24* | | G1: Prefilled liquid Yisaipu, 25mg, BIW  G2: Lyophilised Yisaipu, 25 mg, BIW | | 308  (85.6) | 117  (83.5) | | 1.0  (0.9, 1.1) | | 47.4 | |  | **Low confidence:**  One trial (n≥140 in each arm, high/unclear risk of bias) shows similar ASAS20 responder rates for lyophilised Yisaipu versus either pre-filled liquid 50 or 25mg Yisaipu.  (Downgraded for study limitations, inconsistency – one trial only). |
| NCT04345458^16^  *r-axSpA*  *week 24* | | G1: Prefilled liquid Yisaipu, 50mg, OW  G2: Lyophilised Yisaipu, 25 mg, BIW | | 120  (85.7) | 117  (83.5) | | 1.0  (0.9, 1.1) | | 44.3 | |  |  |
| **ASAS40** | | | | | | | | | | | | |
| ChiCTR1900022520^13^  r-axSpA  *week 24* | | HS016, 40 mg, Q2W, SC | | 296  (71.2) | 175  (75.4) | | 0.9  (0.9, 1.0) | | -23.4 | |  | **High confidence:** biosimilars of ADA have similar effect on disease activity in AxSPA (ASAS40 responder criteria)  3 RCTs, 2 with low risk of bias.  Findings consistently show a similar proportion of pts meeting ASAS40 responder criteria for biosimilars and ADA.  All 3 RCTs include more than 100 pts per arm (larger sample size needed for non-superiority or equivalence (total sample size across all arms: n= 1,466). |
| CTR20181863^14^  r-axSpA  *week 24* | | TQ-Z2301, 40 mg, Q2W, SC | | 135  (71.8) | 132  (68.9) | | 1.0  (0.9, 1.2) | | 34.6 | |  |  |
| NCT02893254^15^  *r-axSpA*  *week 24* | | IBI303, 40 mg, Q2W, SC | | 137  (62) | 144  (65) | | 0.9  (0.8, 1.1) | | -26.4 | |  |  |
| NCT04345458^16^  *r-axSpA*  *week 24* | | G1: Prefilled liquid Yisaipu, 25mg, BIW  G2: Lyophilised Yisaipu, 25 mg, BIW | | 261  (72.5) | 101  (71.9) | | 1.0  (0.9,1.1) | | 178.6 | |  | **Low confidence:**  One trial (n≥140 in each arm, high/unclear risk of bias) shows similar ASAS40 responder rates for lyophilised Yisaipu versus either pre-filled liquid 50 or 25mg Yisaipu.  (Downgraded for study limitations, inconsistency). |
| NCT04345458^16^  *r-axSpA*  *week 24* | | G1: Prefilled liquid Yisaipu, 50mg, OW  G2: Lyophilised Yisaipu, 25 mg, BIW | | 102  (72.9) | 101  (71.9) | | 1.0  (0.9, 1.2) | | 108.7 | |  |  |
| **ASDAS – Clinically Important Improvement (CII): Δ ≥ - 1.1** | | | | | | | | | | | | |
| CTR20181863^14^  r-axSpA  *week 24* | | TQ-Z2301, 40 mg, Q2W, SC | | 161  (85.9) | 160  (83.1) | | 1.0  (1.0, 1.1) | | 35.6 | |  | **Low confidence:** biosimilars of ADA may have similar effect on disease activity in AxSPA (ASDAS-important improvement)  1 RCTs, unclear risk of bias (downgraded for study limitations).  Findings show similar proportion of pts meeting ASDAS clinically important improvement for biosimilars and ADA. (One study, downgraded for consistency).  This RCT includes more than 100 per arm (total sample size across arms: n= 380). |
| NCT04345458^16^  *r-axSpA*  *week 24* | | G1: Prefilled liquid Yisaipu, 25mg, BIW  G2: Lyophilised Yisaipu, 25 mg, BIW | | 311  (86.4) | 120  (86.0) | | 1.0  (0.9, 1.1) | | 285.7 | |  | **Low confidence:**  One trial (n≥140 in each arm, high/unclear risk of bias) shows similar ASDAS clinically important improvement for lyophilised Yisaipu versus either pre-filled liquid 50 or 25mg Yisaipu.  (Downgraded for study limitations, inconsistency). |
| NCT04345458^16^  *r-axSpA*  *week 24* | | G1: Prefilled liquid Yisaipu, 50mg, OW  G2: Lyophilised (powdered) Yisaipu, 25 mg, BIW | | 122  (87) | 120  (86) | | 1.0  (0.9, 1.1) | | 107.5 | |  |  |
| **ASDAS – Major Improvement (MI): Δ ≥ -2.0** | | | | | | | | | | | | |
| CTR20181863^14^  r-axSpA  *week 24* | | TQ-Z2301, 40 mg, Q2W, SC | | 106  (56.5) | 105  (54.6) | | 1.0  (0.9, 1.2) | | 53.2 | |  | **Low confidence:** biosimilars of ADA may have similar effect on disease activity in AxSPA (ASDAS major improvement)  1 RCTs, unclear risk of bias (downgraded for study limitations).  Findings show similar proportion of pts meeting ASDAS major improvement for biosimilars and ADA (One study, downgraded for consistency).  This RCT includes more than 100 per arm (total sample size: n= 380). |
| NCT04345458^16^  *r-axSpA*  *week 24* | | G1: Prefilled liquid Yisaipu, 25mg, BIW  G2: Lyophilised Yisaipu, 25 mg, BIW | | 192  (53.3) | 75  (53.7) | | 1.0  (0.8, 1.2) | | -285.7 | |  | **Low confidence:**  One trial (n≥140 in each arm, high/unclear risk of bias) shows similar ASDAS major improvement for lyophilised Yisaipu versus either pre-filled liquid 50 or 25mg Yisaipu.  (Downgraded for study limitations, inconsistency). |
| NCT04345458^16^  *r-axSpA*  *week 24* | | G1: Prefilled liquid Yisaipu, 50mg, OW  G2: Lyophilised Yisaipu, 25 mg, BIW | | 72  (51.5) | 75  (53.7) | | 1.0  (0.8, 1.2) | | -44.8 | |  |  |
| **ASDAS: Inactive disease (<1.3)** | | | | | | | | | | | | |
| NCT04345458^16^  *r-axSpA*  *week 24* | | G1: Prefilled liquid Yisaipu, 25mg, BIW  G2: Lyophilised Yisaipu, 25 mg, BIW | | 150  (41.7) | 59  (41.9) | | 1.0  (0.8, 1.2) | | -588.2 | |  | **Low confidence:**  One trial (n≥140 in each arm, high/unclear risk of bias) shows similar ASDAS clinically important improvement for lyophilised Yisaipu versus either pre-filled liquid 50 or 25mg Yisaipu.  (Downgraded for study limitations, inconsistency). |
| NCT04345458^16^  *r-axSpA*  *week 24* | | G1: Prefilled liquid Yisaipu, 50mg, OW  G2: Lyophilised Yisaipu, 25 mg, BIW | | 58  (41.3) | 59  (41.9) | | 1.0  (0.8, 1.3) | | -163.9 | |  |  |
| **BASDAI50** | | | | | | | | | | | | |
| ChiCTR1900022520^13^  r-axSpA  *week 24* | | HS016, 40 mg, Q2W, SC | | 318  (76.4) | 182  (78.5) | | 1.0  (0.9, 1.1) | | -49.9 | |  | **High confidence:** biosimilars of ADA have similar effect on disease activity in AxSPA (BASDAI50 responder criteria)  2 RCTs, 1 low, 1 unclear risk of bias.  Both studies show similar proportion of pts meeting BASDAI criteria for improvement for biosimilars and ADA.  Both trials include more than 100 per arm (total sample size across arms: n= 1,028). |
| CTR20181863^14^  r-axSpA  *week 24* | | TQ-Z2301, 40 mg, Q2W, SC | | 134  (71.2) | 136  (71) | | 1.0  (0.9, 1.1) | | 625.0 | |  |  |
| NCT04345458^16^  *r-axSpA*  *week 24* | | G1: Prefilled liquid Yisaipu, 25mg, BIW  G2: Lyophilised Yisaipu, 25 mg, BIW | | 274  (76) | 97  (69.1) | | 1.1  (1.0, 1.2) | | 14.5 | |  | **Low confidence:**  One trial (n≥140 in each arm, high/unclear risk of bias) shows improvement in disease activity (BASDAI50) for lyophilised Yisaipu versus either pre-filled liquid 50 or 25mg Yisaipu.  (Downgraded for study limitations, inconsistency). |
| NCT04345458 (Zhao 2021b^15^)  *r-axSpA*  *week 24* | | G1: Prefilled liquid Yisaipu, 50mg, OW  G2: Lyophilised Yisaipu, 25 mg, BIW | | 104  (74.6) | 97  (69.1) | | 1.1  (0.9, 1.2) | | 18.1 | |  |  |
| **IL-17 inhibitors** | | | | | | | | | | | | |
| **ASAS20** | | | | | | | | | | | | |
| $BE AGILE^17^  *r-axSpA*  *week 12* | | **BKZ,** 16 mg, Q4W, SC | | 25  (41) | 17  (28.3) | | 1.5  (0.9, 2.4) | | 7.9 | |  | **High confidence**  13 RCTs, all but one judged low risk of bias, with fairly consistent findings and high precision – nearly all RCTs included more than 50 pts per arm (total sample size across all arms: n=4,872).  However, there is very low confidence for Netakimab, evaluated in one phase 2 trial, downgraded for risk of bias, inconsistency, and imprecision due to small sample size.  2 RCTs include pts with nr-axSpA, but findings are not substantially different.  **Conclusion**:  A significantly larger proportion of pts with AxSpA receiving IL-17 inhibitors meet ASAS-20 criteria compared to placebo. RR varies between 1.2 and 2.0 for most comparisons.  NNT varies between 3 and 9.  A dose-response relationship is found in most, but not all trials comparing different dosages to placebo. |
|  |  | **BKZ,** 64 mg, Q4W, SC | | 38  (62.3) | 17  (28.3) | | 2.2  (1.4, 3.4) | | 2.9 | |  |  |
|  |  | **BKZ,** 160 mg, Q4W, SC | | 35  (58.3) | 17  (28.3) | | 2.1  (1.3, 3.3) | | 3.3 | |  |  |
|  |  | **BKZ,** 320 mg, Q4W, SC | | 44  (72.1) | 17  (28.3) | | 2.6  (1.7, 3.9) | | 2.3 | |  |  |
| BE MOBILE 1^18^  *nr-axSpA*  *week 16* | | **BKZ,** 160 mg, Q4W, SC | | 88  (68.8) | 48  (38.1) | | 1.8  (1.4, 2.3) | | 3.3 | |  |  |
| BE MOBILE 2^18^  *r-axSpA*  *week 16* | | **BKZ,** 160 mg, Q4W, SC | | 146  (66.1) | 48  (43.2) | | 1.5  (1.2,1.9) | | 4.4 | |  |  |
| NCT02985983^19^  *mixed*  *week 16* | | **BRO**, 210 mg, 3 QW, then Q2W, SC | | 54  (67.5)  **^r^**29 (46)  **^nr^**6 (35.3) | 33  (41.8)  **^r^**16 (25.8)  **^nr^**3 (21.4) | | 1.6  (1.2, 2.2) | | 3.9 | |  |  |
| COAST -V^2^  *r-axSpA*  *week 16* | | **IXE,** 80 mg, Q2W, SC | | 57  (69) | 35  (40) | | 1.7  (1.3, 2.3) | | 3.5 | |  |  |
|  |  | **IXE,** 80 mg, Q4W, SC | | 52  (64) | 35  (40) | | 1.6  (1.2, 2.2) | | 4.2 | |  |  |
| COAST -W^20,21^  *r-axSpA*  *week 16* | | **IXE,** 80 mg, Q2W, SC | | 46  (46.9) | 31  (29.8) | | 1.6  (1.1, 2.3) | | 5.8 | |  |  |
|  |  | **IXE,** 80 mg, Q4W, SC | | 55  (48.2) | 31  (29.8) | | 1.6  (1.1, 2.3) | | 5.4 | |  |  |
| **^¥^**$AILAS^24^  *r-axSpA*  *week 16* | | **NTK**, 40 mg**,** 3 QW, Q2W, SC | | 16  (72.7) | 9  (42.9) | | 1.8  (1.0, 3.1) | | 3.4 | |  |  |
|  |  | **NTK**, 80 mg, 3 QW, Q2W, SC | | 18  (81.8) | 9  (42.9) | | 2.0  (1.2, 3.4) | | 2.6 | |  |  |
|  |  | **NTK**, 120 mg, 3 QW, Q2W, SC | | 20  (90.9) | 9  (42.9) | | 2.2  (1.3, 3.7) | | 2.1 | |  |  |
| **^¥^**MEASURE 1^26-30^  *r-axSpA*  *week 16* | | **SEC,** 150 mg, 4 QW, then Q4W, SC | | 76  (61) | 35  (29) | | 2.1  (1.6, 2.9) | | 3.1 | |  |  |
|  |  | **SEC,** 75 mg,4 QW, then Q4W, SC | | 74  (60) | 35  (29) | | 2.1  (1.5, 2.9) | | 3.2 | |  |  |
| **^¥^**MEASURE 2a*^31-33^  *r-axSpA*  *week 16* | | **SEC**, 75 mg, Q4W, SC | | 23  (51.1) | 14  (31.1) | | 1.6  (1.0, 2.8) | | 5 | |  |  |
|  |  | **SEC,** 150 mg, Q4W, SC | | 30  (68.2) | 14  (31.1) | | 2.2  (1.4, 3.6) | | 2.6 | |  |  |
| **^¥^**MEASURE 2b*^31-33^  *r-axSpA*  *week 16* | | **SEC**, 75 mg, Q4W, SC | | 7  (25) | 7  (24) | | 1.0  (0.4, 2.6) | | 116 | |  |  |
|  |  | **SEC,** 150 mg, Q4W, SC | | 14  (50) | 7  (24) | | 2.1  (1.0, 4.4) | | 3.9 | |  |  |
| **^¥^**MEASURE 3a^34, 35^  *r-axSpA*  *week 16* | | **SEC,** 150 mg, Q4W, IV | | 36  (63.2) | 23  (39.0) | | 1.6  (1.1, 2.4) | | 4.1 | |  |  |
|  |  | **SEC**, 300 mg, Q4W, IV | | 37  (64.9) | 23  (39.0) | | 1.7  (1.2, 2.4) | | 3.9 | |  |  |
| **^$^**MEASURE 3b^34, 35^  *r-axSpA*  *week 16* | | **SEC,** 150 mg, Q4W, IV | | 7  (41.2) | 5  (29.4) | | 1.4  (0.6, 3.6) | | 8.5 | |  |  |
|  |  | **SEC**, 300 mg, Q4W, IV | | 9  (47.4) | 5  (29.4) | | 1.6  (0.7, 3.9) | | 5.6 | |  |  |
| **^¥^**MEASURE 4a^36^  *r-axSpA*  *week 16* | | **SEC**, 150 mg, LD, 4 QW, then Q4W, SC | | 51  (60) | 41  (49.4) | | 1.2  (0.9, 1.6) | | 9.4 | |  |  |
|  |  | **SEC,** 150 mg, No LD, 4 QW, then Q4W, SC | | 53  (62.4) | 41  (49.4) | | 1.3  (1.0,1.7) | | 7.7 | |  |  |
| **^¥^**MEASURE 4b^36^  *r-axSpA*  *week 16* | | **SEC**, 150 mg, LD, 4 QW, then Q4W, SC | | 18  (58.1) | 14  (41.2) | | 1.4  (0.9, 2.3) | | 5.9 | |  |  |
|  |  | **SEC,** 150 mg, No LD, 4 QW, then Q4W, SC | | 19  (59.4) | 14  (41.2) | | 1.4  (0.9, 2.4) | | 5.5 | |  |  |
| **^¥^**MEASURE 5a^37^  *r-axSpA*  *week 16* | | **SEC,** 150 mg, Q4W, SC | | 140  (58.3) | 45  (36.9) | | 1.6  (1.2, 2.0) | | 4.7 | |  |  |
| **^¥^**MEASURE 5b^37^  *r-axSpA*  *week 16* | | **SEC,** 150 mg, Q4W, SC | | 38  (58.5) | 11  (35.5) | | 1.7  (1.0, 2.8) | | 4.4 | |  |  |
| PREVENT^38^  *nr-axSpA*  *week 16* | | **SEC,** 150 mg, LD, 4 QW, then Q4W, SC | | 105  (56.8) | 85  (45.7) | | 1.2  (1.0,1.5) | | 9.3 | |  |  |
|  |  | **SEC**,150 mg, NL, 4 QW, then Q4W, SC | | 107  (58.2) | 85  (45.7) | | 1.3  (1.0,1.6) | | 8.2 | |  |  |
| **ASAS40** | | | | | | | | | | | | |
| **^¥^**$BE AGILE^17^  *r-axSpA*  *week 12* | | **BKZ,** 16 mg, Q4W, SC | | 18  (29.5) | 8  (13.3) | | 2.2  (1.0, 4.7) | | 6.2 | |  | **High confidence**  13 RCTs, all but one judged low risk of bias, with consistent findings. Most trials include >50 pts per arm (total sample size across all arms: n=5,136).  Furthermore, there is very low confidence for Netakimab, evaluated in one phase 2 trial, downgraded for risk of bias, inconsistency, and imprecision.  **Conclusion**:  A significantly larger proportion of pts with AxSpA receiving IL-17 inhibitors meet ASAS-40 criteria compared to placebo. RR varies between 1.3 and 2.6 for most comparisons.  NNT varies between 3 and 8.  A dose-response relationship is found in most, but not all trials comparing different dosages to placebo. |
|  |  | **BKZ,** 64 mg, Q4W, SC | | 26  (42.6) | 8  (13.3 | | 3.2  (1.6, 6.5) | | 3.4 | |  |  |
|  |  | **BKZ,** 160 mg, Q4W, SC | | 28  (46.7) | 8  (13.3) | | 3.5  (1.7, 7.0) | | 3 | |  |  |
|  |  | **BKZ,** 320 mg, Q4W, SC | | 28  (45.9) | 8  (13.3) | | 3.4  (1.7, 6.9) | | 3.1 | |  |  |
| **^¥^**BE MOBILE 1^18^  *nr-axSpA*  *week 16* | | **BKZ,** 160 mg, Q4W, SC | | 61  (47.7) | 27 (21.4) | | 2.2  (1.5, 3.3) | | 3.8 | |  |  |
| **^¥^**BE MOBILE 2^18^  *r-axSpA*  *week 16* | | **BKZ,** 160 mg, Q4W, SC | | 99  (44.8) | 25 (22.5) | | 2.0  (1.4, 2.9) | | 4.5 | |  |  |
| **^¥^**NCT02985983^19^  *mixed*  *week 16* | | **BRO**, 210 mg, 3 QW, then Q2W, SC | | 35  (43.8)  **^r^**29 (46)  **^nr^**6 (35.3) | 19  (24.1)  **^r^**16 (25.8)  **^nr^**3 (21.4) | | 1.8  (1.1, 2.9) | | 5.1 | |  |  |
| **^¥^**COAST -V^2^  *r-axSpA*  *week 16* | | **IXE,** 80 mg, Q2W, SC | | 43  (52) | 16  (18) | | 2.8  (1.7, 4.6) | | 3 | |  |  |
|  |  | **IXE,** 80 mg, Q4W, SC | | 39  (48) | 16  (18) | | 2.6  (1.6, 4.3) | | 3.4 | |  |  |
| **^¥^**COAST -W ^20,21^  *r-axSpA*  *week 16* | | **IXE,** 80 mg, Q2W, SC | | 30  (30.6) | 13 (12.5) | | 2.4  (1.3, 4.4) | | 5.5 | |  |  |
|  |  | **IXE,** 80 mg, Q4W, SC | | 29  (25.4) | 13 (12.5) | | 2.5  (1.4, 4.5) | | 7.7 | |  |  |
| **^¥^**COAST - X^22,23^  *nr-axSpA*  *week 16* | | **IXE,** 80 mg, Q2W, SC | | 41  (40) | 20  (19) | | 2.1  (1.3, 3.3) | | 4.7 | |  |  |
|  |  | **IXE,** 80 mg, Q4W, SC | | 34  (35) | 20  (19) | | 1.9  (1.2, 3.0) | | 6.1 | |  |  |
| $AILAS^24^  *r-axSpA*  *week 16* | | **NTK**, 40 mg**,** 3 QW, Q2W, SC | | 9  (40.91) | 3  (14.29) | | 3.0  (0.9, 9.6) | | 3.8 | |  |  |
|  |  | **NTK**, 80 mg, 3 QW, Q2W, SC | | 14  (63.64) | 3  (14.29) | | 4.7  (1.6,14.0) | | 2.0 | |  |  |
|  |  | **NTK**, 120 mg, 3 QW, Q2W, SC | | 16  (72.73) | 3  (14.29) | | 5.3  (1.8,15.7) | | 1.7 | |  |  |
| MEASURE 1^26-30^  *r-axSpA*  *week 16* | | **SEC,** 150 mg, Q4W, SC | | 52  (42) | 16  (13) | | 3.2  (1.9, 5.2) | | 3.5 | |  |  |
|  |  | **SEC,** 75 mg, Q4W (starting W 8), SC | | 41  (33) | 16  (13) | | 2.5  (1.5, 4.2) | | 5 | |  |  |
| MEASURE 2a*^31-33^  *r-axSpA*  *week 16* | | **SEC**, 75 mg, Q4W, SC | | 14  (31.1) | 8  (17.8) | | 1.8  (0.8, 3.8) | | 7.5 | |  |  |
|  |  | **SEC,** 150 mg, Q4W, SC | | 19  (43.2) | 8  (17.8) | | 2.4  (1.2, 5.0) | | 3.9 | |  |  |
| MEASURE 2b*^31-33^  *r-axSpA*  *week 16* | | **SEC**, 75 mg, Q4W, SC | | 5  (17.9) | 0  (0) | | 11.4^cc^  (0.7, 196.7) | | 5.6 | |  |  |
|  |  | **SEC,** 150 mg, Q4W, SC | | 7  (25) | 0  (0) | | 15.5^cc^  (0.9, 259.5) | | 4 | |  |  |
| MEASURE 3a^14,35^  *r-axSpA*  *week 16* | | **SEC,** 150 mg, Q4W, IV | | 25  (43.9) | 14 (23.7) | | 1.9  (1.1, 3.2) | | 5 | |  |  |
|  |  | **SEC**, 300 mg, Q4W, IV | | 25  (43.9) | 14 (23.7) | | 1.9  (1.1, 3.2) | | 5 | |  |  |
| MEASURE 3b ^34,35^  *r-axSpA*  *week 16* | | **SEC,** 150 mg, Q4W, IV | | 5  (29.4) | 2  (11.8) | | 2.5  (0.6, 11.2) | | 5.7 | |  |  |
|  |  | **SEC**, 300 mg, Q4W, IV | | 7  (36.8) | 2  (11.8) | | 3.1  (0.8, 13.1) | | 4 | |  |  |
| MEASURE 4a^36^  *r-axSpA*  *week 16* | | **SEC**, 150 mg, LD, 4 QW, then Q4W, SC | | 34  (40) | 25 (30.1) | | 1.3  (0.9, 2.0) | | 10.1 | |  |  |
|  |  | **SEC,** 150 mg, No LD, 4 QW, then Q4W, SC | | 33  (38.8) | 25 (30.1) | | 1.3  (0.8, 2.0) | | 11.5 | |  |  |
| MEASURE 4b^36^  *r-axSpA*  *week 16* | | **SEC**, 150 mg, LD, 4 QW, then Q4W, SC | | 11  (35.5) | 8  (23.5) | | 1.5  (0.7, 3.3) | | 8.4 | |  |  |
|  |  | **SEC,** 150 mg, No LD, 4 QW, then Q4W, SC | | 9  (28.1) | 8  (23.5) | | 1.2  (0.5, 2.7) | | 21.76 | |  |  |
| MEASURE 5a^37^  *r-axSpA*  *week 16* | | **SEC,** 150 mg, Q4W, SC | | 102  (42.5) | 22  (18) | | 2.4  (1.6, 3.5) | | 4.1 | |  |  |
| MEASURE 5b^37^  *r-axSpA*  *week 16* | | **SEC,** 150 mg, Q4W, SC | | 26  (42.6) | 4  (12.9) | | 3.1  (1.2, 8.1) | | 3.7 | |  |  |
| **^¥^**PREVENT^38^  *nr-axSpA*  *week 16* | | **SEC,** 150 mg, LD, 4 QW, then Q4W, SC | | 77  (41.5) | 54  (29.2) | | 1.4  (1.1`, 1.9) | | 8.1 | |  |  |
|  |  | **SEC**,150 mg, NL, 4 QW, then Q4W, SC | | 78  (42.2) | 54  (29.2) | | 1.5  (1.1, 1.9) | | 7.7 | |  |  |
| **ASDAS Clinically important improvement (Δ≥1.1)** | | | | | | | | | | | | |
| NCT02985983^19^  *mixed*  *week 16* | | **BRO**, 210 mg, 3 QW, then Q2W, SC | | 12  (15) | 5  (6.3) | | 2.4  (0.9, 6.4) | | 11.5 | |  | **High confidence**  3 RCTs, all low risk of bias and with consistent findings. Precision is high, with >50 pts per arm (total sample size across arms: n= 944).  **Conclusion**:  A significantly larger proportion of pts with AxSpA receiving IL-17 inhibitors meet ASDAS criteria for clinically important improvement. RR varies from 2.0 to 2.8 compared to placebo.  NNT varies between 2.5 and 6.3. |
| COAST -V ^2^  *r-axSpA*  *week 16* | | **IXE,** 80 mg, Q2W, SC | | 50  (60.2) | 20  (20.3) | | 2.6  (1.7, 4.0) | | 2.7 | |  |  |
|  |  | **IXE,** 80 mg, Q4W, SC | | 50  (60.2) | 20  (20.3) | | 2.7  (1.8, 4.1) | | 2.6 | |  |  |
| COAST -W ^20,21^  *r-axSpA*  *week 16* | | **IXE,** 80 mg, Q2W, SC | | 48  (85) | 18  (19.4) | | 2.1  (1.3, 3.4) | | 3.2 | |  |  |
|  |  | **IXE,** 80 mg, Q4W, SC | | 51  (44.7) | 18  (19.4) | | 2.8  (1.8, 4.4) | | 3.6 | |  |  |
| **ASDAS – Major Improvement (Δ≥2.0)** | | | | | | | | | | | | |
| $BE AGILE^17^  *r-axSpA*  *week 12* | | **BKZ,** 16 mg, Q4W, SC | | 11  (18.97) | 0  (0) | | 23.8^cc^  (1.4, 394.5) | | 5.3 | |  | **Moderate confidence**  6 RCTs, all low risk of bias and consistent findings. Down-graded for precision: all trials include >50 pts per arm (total sample size across all arms: n= 1,974), but confidence intervals are wide or cannot be estimated due to small number of pts achieving major improvement.  **Conclusion**:  A significantly larger proportion of pts with AxSpA receiving IL-17 inhibitors meet ASDAS criteria for major improvement. RR is larger than 4 compared to placebo.  NNT between 3 and 9. |
|  |  | **BKZ,** 64 mg, Q4W, SC | | 21  (35.59) | 0  (0) | | 43.7^cc^  (2.7, 705.5) | | 2.8 | |  |  |
|  |  | **BKZ,** 160 mg, Q4W, SC | | 15  (25.9) | 0  (0) | | 32.1^cc^  (2.0, 523.6) | | 3.9 | |  |  |
|  |  | **BKZ,** 320 mg, Q4W, SC | | 19  (31.1) | 0  (0) | | 38.4 ^cc^  (2.4, 621.5) | | 3.2 | |  |  |
| BE MOBILE 1^18^  *nr-axSpA*  *week 16* | | **BKZ,** 160 mg, Q4W, SC | | 35  (27.3) | 9  (7.1) | | 3.8  (1.9, 7.6) | | 5 | |  |  |
| BE MOBILE 2^18^  *r-axSpA*  *week 16* | | **BKZ,** 160 mg, Q4W, SC | | 57  (25.8) | 6  (5.4) | | 4.8  (2.1, 10.7) | | 4.9 | |  |  |
| NCT02985983 ^19^  *mixed*  *week 16* | | **BRO**, 210 mg, 3 QW, then Q2W, SC | | 12  (15) | 5  (6.3) | | 2.4  (0.9, 6.4) | | 11.5 | |  |  |
| COAST -V^2^  *r-axSpA*  *week 16* | | **IXE,** 80 mg, Q2W, SC | | 19  (22.9) | 4  (4.7) | | 5.2  (1.9, 14.7) | | 5.5 | |  |  |
|  |  | **IXE,** 80 mg, Q4W, SC | | 24  (29.6) | 4  (4.7) | | 4.9  (1.8, 13.6) | | 4 | |  |  |
| COAST -W ^20,21^  *r-axSpA*  *week 16* | | **IXE,** 80 mg, Q2W, SC | | 21  (21.4) | 4  (4.3) | | 5.0  (1.8, 14.0) | | 5.8 | |  |  |
|  |  | **IXE,** 80 mg, Q4W, SC | | 18  (15.8) | 4  (4.3) | | 3.7  (1.3, 10.5) | | 8.7 | |  |  |
| **ASDAS: Inactive disease (<1.3)** | | | | | | | | | | | | |
| $BE AGILE^17^  *r-axSpA*  *week 12* | | **BKZ,** 16 mg, Q4W, SC | | 2  (3.45) | 0  (0) | | 5.2 ^cc^  (0.3, 105.4) | | 29 | |  | **Moderate confidence**  7 RCTs, all low risk of bias and consistent findings in terms of direction of effect. Down-graded for precision: all trials include >50 pts per arm (total sample size across all arms: n= 2,353), but confidence intervals are wide or cannot be estimated due to small number of pts achieving inactive disease status.  **Conclusion**:  A larger proportion of pts with AxSpA receiving IL-17 inhibitors meet ASDAS criteria for inactive disease. RR is larger than 3.7 compared to placebo for all comparisons but results not statistically significant in all trials.  NNT varies widely, mostly due to very low event rate |
|  |  | **BKZ,** 64 mg, Q4W, SC | | 6  (10.17) | 0  (0) | | 13.2 ^cc^  (0.8, 229.5) | | 9.8 | |  |  |
|  |  | **BKZ,** 160 mg, Q4W, SC | | 6  (10.3) | 0  (0) | | 13.4 ^cc^  (0.8, 233.3) | | 9.7 | |  |  |
|  |  | **BKZ,** 320 mg, Q4W, SC | | 6  (9.8) | 0  (0) | | 12.8 ^cc^  (0.7, 222.2) | | 10.2 | |  |  |
| BE MOBILE 1^18^  *nr-axSpA*  *week 16* | | **BKZ,** 160 mg, Q4W, SC | | 24  (18.8) | 8  (6) | | 3.0  (1.4, 6.3) | | 8.1 | |  |  |
| BE MOBILE 2^18^  *r-axSpA*  *week 16* | | **BKZ,** 160 mg, Q4W, SC | | 36  (16.5) | 5  (4.6) | | 3.7  (1.5, 9.2) | | 8.4 | |  |  |
| NCT02985983^19^  *mixed*  *week 16* | | **BRO**, 210 mg, 3 QW, then Q2W, SC | | 39  (48.8) | 23  (29.1) | | 1.7  (1.1, 2.5) | | 5.1 | |  |  |
| COAST -V ^2^  *r-axSpA*  *week 16* | | **IXE,** 80 mg, Q2W, SC | | 9  (11) | 2  (2) | | 4.7  (1.1, 21.2) | | 11.7 | |  |  |
|  |  | **IXE,** 80 mg, Q4W, SC | | 13  (16) | 2  (2) | | 7.0  (1.6, 30.0 | | 7.3 | |  |  |
| COAST -W ^20,21^  *r-axSpA*  *week 16* | | **IXE,** 80 mg, Q2W, SC | | 5  (5.1) | 1  (1.1) | | 5.2  (0.6, 43.4 | | 24.8 | |  |  |
|  |  | **IXE,** 80 mg, Q4W, SC | | 4  (3.5) | 1  (1.1) | | 6.3  (0.7, 55.1) | | 41.1 | |  |  |
| MEASURE 5 ^37^  *r-axSpA*  *week 16* | | **SEC,** 150 mg, Q4W, SC | | 45  (14.8) | 5  (3.3) | | 4.5  (1.8, 11.4) | | 8.7 | |  |  |
| **ASDAS: Low disease activity (<2.1)** | | | | | | | | | | | | |
| $BE AGILE^17^  *r-axSpA*  *week 12* | | **BKZ,** 16 mg, Q4W, SC | | 9  (15.5) | 8  (13.3) | | 1.2  (0.5, 2.8) | | 45.8 | |  | **High confidence**  7 RCTs, all low risk of bias.  Findings are fairly consistent, although one study reports no benefit of IL-17 inhibitors.  Precision is high, with >50 pts per arm (total sample size across arms: n= 2,392).  **Conclusion**:  A significantly larger proportion of pts with AxSpA receiving IL-17 inhibitors meet ASDAS criteria for low disease activity. RR varies between 1.2 and 3.6 compared to placebo.  NNT varies between 3 and 9 for most comparisons. |
|  |  | **BKZ,** 64 mg, Q4W, SC | | 19  (32.2) | 8  (13.3) | | 2.4  (1.2, 5.1) | | 5.3 | |  |  |
|  |  | **BKZ,** 160 mg, Q4W, SC | | 16  (27.6) | 8  (13.3) | | 2.1  (1.0, 4.5) | | 7 | |  |  |
|  |  | **BKZ,** 320 mg, Q4W, SC | | 20  (32.8) | 8  (13.3) | | 2.5  (1.2, 5.2) | | 5.1 | |  |  |
| BE MOBILE 1^18^  *nr-axSpA*  *week 16* | | **BKZ,** 160 mg, Q4W, SC | | 35  (27.4) | 18  (14.2) | | 2.0  (1.2, 3.3) | | 7.6 | |  |  |
| BE MOBILE 2^18^  *r-axSpA*  *week 16* | | **BKZ,** 160 mg, Q4W, SC | | 63  (28.4) | 14  (12.9) | | 2.3  (1.3, 3.9) | | 6.5 | |  |  |
| NCT02985983^19^  *mixed*  *week 16* | | **BRO**, 210 mg, 3 QW, then Q2W, SC | | 22  (27.5) | 22  (27.8) | | 1.0  (0.6, 1.6) | | 333.3 | |  |  |
| COAST -V^2^  *r-axSpA*  *week 16* | | **IXE,** 80 mg, Q2W, SC | | 35  (42.2) | 11  (13) | | 3.3  (1.8, 6.1) | | 3.4 | |  |  |
|  |  | **IXE,** 80 mg, Q4W, SC | | 35  (43.2) | 11  (13) | | 3.4  (1.9, 6.3) | | 3.3 | |  |  |
| COAST -W^20,21^  *r-axSpA*  *week 16* | | **IXE,** 80 mg, Q2W, SC | | 16  (16.3) | 5  (4.8) | | 3.4  (1.3, 8.9) | | 8.7 | |  |  |
|  |  | **IXE,** 80 mg, Q4W, SC | | 20  (17.5) | 5  (4.8) | | 3.7  (1.4, 9.4) | | 7.9 | |  |  |
| COAST - X^22,23^  *nr-axSpA*  *week 16* | | **IXE,** 80 mg, Q2W, SC | | 33  (32) | 13  (12) | | 2.6  (1.5, 4. 7) | | 5 | |  |  |
|  |  | **IXE,** 80 mg, Q4W, SC | | 36  (28) | 13  (12) | | 2.2  (1.2, 4.0) | | 6.8 | |  |  |
| **BASDAI50** | | | | | | | | | | | | |
| $BE AGILE^17^  *r-axSpA*  *week 12* | | **BKZ,** 16 mg, Q4W, SC | | 14  (24.1) | 7  (11.7) | | 2.1  (0.9, 4.8) | | 8 | |  | **High confidence**  4 RCTs, all low risk of bias and with consistent findings. Precision is high, with >50 pts per arm (total sample size across arms: n= 1,931).  **Conclusion**:  A significantly larger proportion of pts with AxSpA receiving IL-17 inhibitors meet ASDAS criteria for low disease activity. RR varies between 1.8 and 4 compared to placebo.  NNT varies between 3 and 9 for most comparisons |
|  |  | **BKZ,** 64 mg, Q4W, SC | | 26  (44.1) | 7  (11.7) | | 3.78  (1.8, 8.0) | | 3.1 | |  |  |
|  |  | **BKZ,** 160 mg, Q4W, SC | | 22  (37.9) | 7  (11. 7) | | 3.3  (1.5, 7.0) | | 3.8 | |  |  |
|  |  | **BKZ,** 320 mg, Q4W, SC | | 29  (47.5) | 7  (11.7) | | 4.1  (1.9, 8.6) | | 2.8 | |  |  |
| COAST -V^2^  *r-axSpA*  *week 16* | | **IXE,** 80 mg, Q2W, SC | | 36  (43) | 15  (17) | | 2.5  (1.5, 4.2) | | 3.8 | |  |  |
|  |  | **IXE,** 80 mg, Q4W, SC | | 34  (42) | 15  (17) | | 2.4  (1.4, 4.1) | | 4 | |  |  |
| COAST -W ^20,21^  *r-axSpA*  *week 16* | | **IXE,** 80 mg, Q2W, SC | | 23  (23.5) | 10  (10.8) | | 2.0  (1.0, 3.9) | | 7.9 | |  |  |
|  |  | **IXE,** 80 mg, Q4W, SC | | 25  (21.9) | 10  (10.8) | | 2.9  (1.5, 5.7) | | 9 | |  |  |
| PREVENT^38^  *nr-axSpA*  *week 16* | | **SEC,** 150 mg, LD, 4 QW, then Q4W, SC | | 69  (37.3) | 39  (21) | | 1.8  (1.3, 2.5) | | 6.1 | |  |  |
|  |  | **SEC**,150 mg, NL, 4 QW, then Q4W, SC | | 69  (37.5) | 39  (21) | | 1.8  (1.3, 2.5) | | 6.1 | |  |  |
| **IL-6 inhibitors** | | | | | | | | | | | | |
| **ASAS20** | | | | | | | | | | | | |
| **^¥^**$ALIGN^39^  mixed *-axSpA*  *week 12* | | Sarilumab**,** 100 mg, Q2W, SC | | 12  (24.5) | 12  (24.0) | | 1.0  (0.5, 2.1) | | 204.2 | |  | **Very low confidence**  1 RCT with a total sample size = 501, considered low risk of bias. Downgraded for inconsistency (one study only), and imprecision (wide confidence intervals). The study does not report significantly higher proportions of pts meeting ASAS20 criteria for Sarilumab (various dosages) compared to placebo.  **Conclusion:**  There is insufficient evidence to make any conclusion regarding the effectiveness of IL-6i on disease activity (ASAS20). |
|  |  | Sarilumab**,** 150 mg, Q2W, SC | | 15  (30.0) | 12  (24.0) | | 1.3  (0.7, 2.4) | | 16.7 | |  |  |
|  |  | Sarilumab**,** 100 mg, QW, SC | | 10  (19.2) | 12  (24.0) | | 0.8  (0.4, 1.7) | | -21 | |  |  |
|  |  | Sarilumab**,** 200 mg, Q2W, SC | | 15  (30.0) | 12  (24.0) | | 1.3  (0.7, 2.4) | | 16.7 | |  |  |
|  |  | Sarilumab**,** 150 mg, QW, SC | | 19  (38.0) | 12  (24.0) | | 1.6  (0.9, 2.9) | | 7.1 | |  |  |
| **ASAS40** | | | | | | | | | | | | |
| $ALIGN^39^  mixed *-axSpA*  *week 12* | | Sarilumab**,** 100 mg, Q2W, SC | | 7  (14.3) | 4  (8.0) | | 1.8  (0.6, 5.7) | | 15.9 | |  | **Very low confidence**  1 RCT with a total sample size = 501, considered low risk of bias. Downgraded for inconsistency (one study only), and imprecision (wide confidence intervals). The study does not report significantly higher proportions of pts meeting ASAS40 criteria for Sarilumab (various dosages) compared to placebo.  **Conclusion:**  There is insufficient evidence to make any conclusive decision about the effectiveness of IL-6i on disease activity (ASAS40). |
|  |  | Sarilumab**,** 150 mg, Q2W, SC | | 8  (16.0) | 4  (8.0) | | 2.0  (0.6, 6.2) | | 12.5 | |  |  |
|  |  | Sarilumab**,** 100 mg, QW, SC | | 3  (5.8) | 4  (8.0) | | 0.7  (0.2, 3.1) | | -44.8 | |  |  |
|  |  | Sarilumab**,** 200 mg, Q2W, SC | | 9  (18.0) | 4  (8.0) | | 2.3  (0.7, 6.8) | | 10 | |  |  |
|  |  | Sarilumab**,** 150 mg, QW, SC | | 10  (20.0) | 4  (8.0) | | 2.5  (0.8, 7.5) | | 8.3 | |  |  |
| **IL-23 inhibitors** | | | | | | | | | | | | |
| **ASAS20** | | | | | | | | | | | | |
| $NCT02047110 **^40^**  *r-axSpA*  *week 12* | | Risankizumab,18 mg, SD, SC | | 18  (45) | 8  (20) | | 2.3  (1.1, 4.6) | | 4 | |  | **Very low confidence**  1 RCT with a total sample size = 234, considered low risk of bias. Downgraded for inconsistency (one study only), and imprecision (wide confidence intervals, sample size <50 per arm). Reported differences between different doses of Risankizumab and placebo are not statistically significant).  **Conclusion:**  There is insufficient evidence to make any conclusion regarding the effectiveness of IL-23i on disease activity (ASAS20). |
|  |  | Risankizumab, 90 mg**^ν^,** SC | | 13  (33) | 8  (20) | | 1.7  (0.8, 3.6) | | 7.7 | |  |  |
|  |  | ^Ə1^ Risankizumab, 180 mg**^ν^**, SC | | 12  (30) | 8  (20) | | 1.5  (0.7, 3.3) | | 10 | |  |  |
| **ASAS40** | | | | | | | | | | | | |
| **^¥^**$NCT02047110 **^40^**  *r-axSpA*  *week 12* | | Risankizumab, 18 mg, SD, SC | | 10  (25) | 7  (18) | | 1.4  (0.6, 3.4) | | 14.3 | |  | **Very low confidence**  1 RCT with a total sample size = 234, considered low risk of bias. Downgraded for inconsistency (one trial only), and imprecision (wide confidence intervals, sample size <50 per arm). Reported differences between different doses of Risankizumab and placebo are not statistically significant).  **Conclusion:**  There is insufficient evidence to make any conclusive decision about the effectiveness of IL-23i on disease activity (ASAS40). |
|  |  | Risankizumab, 90 mg**^ν^,** SC | | 8  (21) | 7  (18) | | 1.2  (0.5, 2.9) | | 33.3 | |  |  |
|  |  | ^Ə1^ Risankizumab, 180 mg**^ν^**, SC | | 6  (15) | 7  (18) | | 0.9  (0.3, 2.3) | | -33.3 | |  |  |
| **JAK inhibitors** | | | | | | | | | | | | |
| **ASAS20** | | | | | | | | | | | | |
| TORTUGA ^41, 42^  r-axSpA  week 12 | Filgotinib 200mg, OD, oral | | 44  (76) | | | 23  (40) | | 1.9  (1.4, 2.7) | | 2.8 |  | **HIGH confidence**  5 RCTs, all low risk of bias.  Some heterogeneity in terms of type of JAKi, and one study including nr-AxSpA. However, findings seem fairly consistent, especially in the 4 studies with higher precision (larger no of pts per arm). All studies have >50 pts per arm (total sample size: n= 1,092). No strong reason to downgrade for risk of bias, inconsistency, or imprecision.  **Conclusion**:  A significantly larger proportion of pts with AxSpA receiving JAKi meet ASAS-20 criteria compared to placebo. RR: range 1.2 to 1.9  NNT: range 3 to 9 (for the 4 larger studies: 2.7 to 4.4). |
| **^¥^**NCT01786668 ^43^  *r-axSpA*  *week 12* | Tofacitinib 2 mg, BID; oral | | 27  (51.9) | | | 21  (41.2) | | 1.2,  (0.8, 1.9) | | 9.4 |  |  |
|  | Tofacitinib 5mg, BID, oral | | 42  (80.8) | | | 21  (41.2) | | 2.0  (1.4, 2.8) | | 2.5 |  |  |
|  | Tofacitinib 10 mg, BID | | 29  (55.8) | | | 21  (41.2) | | 1.4  (0.9, 2.0) | | 6.9 |  |  |
| **^¥^**NCT03502616 ^44,45^  *r-axSpA*  *week 16* | Tofacitinib 5mg, BID, oral | | 88  (68.8) | | | 48  (38.1) | | 1.9  (1.4, 2.6) | | 3.7 |  |  |
| SELECT-AXIS 1^46,47^  *r-axSpA*  *week 14* | Upadacitinib 15mg OD, oral | | 60  (65) | | | 38  (40) | | 1.6  (1.2, 2.1) | | 4.2 |  |  |
| SELECT-AXIS 2 ^48,49^  *nr-axSpA*  *week 14* | Upadacitinib 15mg OD, oral | | 104  (66.7) | | | 69  (43.8) | | 1.5  (1.3, 1.9) | | 4.4 |  |  |
| **ASAS40** | | | | | | | | | | | | |
| TORTUGA ^41, 42^  r-axSpA  week 12 | Filgotinib 200mg, OD, oral | | 22  (38) | | | 11  (19) | | 2.0  (1.1, 3.7) | | 5.3 |  | **HIGH confidence**  5 RCTs, all low risk of bias. Consistent findings and with sufficient precision: all >50 pts per arm, fairly narrow confidence intervals (total sample size: n= 1,092). No reason to downgrade for risk of bias, inconsistency, or imprecision.  **Conclusion**:  A significantly larger proportion of pts with AxSpA receiving JAKi meet ASAS-40 criteria compared to placebo. RR: range 2.0 to 3.3  NNT: range 3.6 to 5.3 |
| NCT01786668 ^43^  *r-axSpA*  *week 12* | Tofacitinib 2 mg, BID; oral | | 22  (42.3) | | | 10  (19.6) | | 2.3  (1.2, 4.5) | | 4.4 |  |  |
|  | Tofacitinib 5mg, BID, oral | | 24  (46.2) | | | 10  (19.6) | | 2.6  (1.4, 5.1) | | 3.8 |  |  |
|  | Tofacitinib 10 mg, BID | | 20  (38.5) | | | 10  (19.6) | | 2.2  (1.1, 4.3) | | 5.3 |  |  |
| NCT03502616 ^44,45^  *r-axSpA*  *week 16* | Tofacitinib 5mg, BID, oral | | 54  (40.6) | | | 17  (12.5) | | 3.3  (2.0, 5.3) | | 3.6 |  |  |
| **^¥^**SELECT-AXIS 1^46,47^  *r-axSpA*  *week 14* | Upadacitinib 15mg OD, oral | | 48  (52) | | | 24  (26) | | 2.0  (1.4, 3.0) | | 3.8 |  |  |
| **^¥^**SELECT-AXIS 2 ^48,49^  *nr-axSpA*  *week 14* | Upadacitinib 15mg OD, oral | | 70  (45) | | | 35  (23) | | 2.0  (1.4, 2.8) | | 4.4 |  |  |
| **ASDAS – Clinically Important Improvement (CII): Δ ≥ - 1.1** | | | | | | | | | | | | |
| TORTUGA ^41, 42^  r-axSpA  week 12 | Filgotinib 200mg, OD, oral | | 38  (66) | | | 15  (26) | | 2.5  (1.6, 4.1) | | 2.5 |  | **HIGH confidence**  5 RCTs, all low risk of bias. Consistent findings and with sufficient precision: all >50 pts per arm, fairly narrow confidence intervals (total sample size: n= 1,092). No reason to downgrade for risk of bias, inconsistency, or imprecision.  **Conclusion**:  A significantly larger proportion of pts with AxSpA receiving JAKi meet ASDAS criteria for clinically important improvement compared to placebo. RR: range 1.8 to 3.2  NNT: range 2.4 to 4.2 |
| NCT01786668 ^43^  *r-axSpA*  *week 12* | Tofacitinib 2 mg, BID; oral | | 27  (51.9) | | | 14  (27.5) | | 1.8  (1.1, 3.1) | | 4.1 |  |  |
|  | Tofacitinib 5mg, BID, oral | | 33  (63.5) | | | 14  (27.5) | | 2.3  (1.4, 3.8) | | 2.8 |  |  |
|  | Tofacitinib 10 mg, BID | | 29  (55.8) | | | 14  (27.5) | | 2.0  (1.2, 3.4) | | 3.5 |  |  |
| NCT03502616 ^44,45^  *r-axSpA*  *week 16* | Tofacitinib 5mg, BID, oral | | 81  (61.4) | | | 26  (19.1) | | 3.2  (2.2, 4.7) | | 2.4 |  |  |
| SELECT-AXIS 1^46,47^  *r-axSpA*  *week 14* | Upadacitinib 15mg OD, oral | | 49  (53) | | | 17  (18) | | 2.9  (1.8, 4.7) | | 2.9 |  |  |
| SELECT-AXIS 2 ^48,49^  *nr-axSpA*  *week 14* | Upadacitinib 15mg OD, oral | | 81  (52) | | | 44  (28) | | 1.9  (1.4, 2.6) | | 4.2 |  |  |
| **ASDAS – Major Improvement (Δ≥2.0)** | | | | | | | | | | | | |
| TORTUGA ^41, 42^  r-axSpA  week 12 | Filgotinib 200mg, OD, oral | | 19  (33) | | | 1  (2) | | 19.0  (2.6, 137.3) | | 3.2 |  | **Moderate confidence**  5 RCTs, all low risk of bias. All studies >50 pts per arm (total sample size: n= 1,092). Findings are less consistent in terms of magnitude, but likely mainly due to low event rates, leading to low precision (wide confidence intervals). Downgraded for imprecision.  **Conclusion**:  A larger proportion of pts with AxSpA receiving JAKi meet ASDAS criteria for major improvement compared to placebo. RR: range 1.5 to 19  NNT: range 3.2 to 13.5 |
| NCT01786668 ^43^  *r-axSpA*  *week 12* | Tofacitinib 2 mg, BID; oral | | 10  (19.2) | | | 6  (11.8) | | 1.5  (0.6, 3.8) | | 13.5 |  |  |
|  | Tofacitinib 5mg, BID, oral | | 12  (23.1) | | | 6  (11.8) | | 2.0  (0.8, 4.8) | | 8.8 |  |  |
|  | Tofacitinib 10 mg, BID | | 13  (25.0) | | | 6  (11.8) | | 2.1  (0.9, 5.1) | | 7.6 |  |  |
| NCT03502616 ^44,45^  *r-axSpA*  *week 16* | Tofacitinib 5mg, BID, oral | | 37  (30.1) | | | 6  (4.7) | | 6.5  (2.8, 14.8) | | 3.9 |  |  |
| SELECT-AXIS 1^46,47^  *r-axSpA*  *week 14* | Upadacitinib 15mg OD, oral | | 30  (32) | | | 5  (5) | | 6.1  (2.5, 15.0) | | 3.7 |  |  |
| SELECT-AXIS 2 ^48,49^  *nr-axSpA*  *week 14* | Upadacitinib 15mg OD, oral | | 37  (24) | | | 14  (9) | | 2.7  (1.5, 4.7) | | 6.7 |  |  |
| **ASDAS: Inactive disease (<1.3)** | | | | | | | | | | | | |
| TORTUGA ^41, 42^  r-axSpA  week 12 | Filgotinib 200mg, OD, oral | | 3  (5) | | | 0 | | 7.0 ^cc^  (0.4, 132.6) | | 19.3 |  | **Moderate confidence**  5 RCTs, all low risk of bias. All studies >50 pts per arm (total sample size: n= 1,092). Findings are less consistent in terms of magnitude and statistical significance, likely due to low event rates, leading to low precision (wide confidence intervals). Downgraded for imprecision  **Conclusion**:  A larger proportion of pts with AxSpA receiving JAKi meet ASDAS criteria for inactive disease compared to placebo. RR: range 2.2 to 31  NNT: range 6.2 to 19.3 |
| NCT01786668 ^43^  *r-axSpA*  *week 12* | Tofacitinib 2 mg, BID; oral | | 7  (13.5) | | | 4  (7.8) | | 2.3  (0.6, 8.4) | | 17.5 |  |  |
|  | Tofacitinib 5mg, BID, oral | | 7  (13.5) | | | 4  (7.8) | | 2.3  (0.6, 8.4) | | 17.5 |  |  |
|  | Tofacitinib 10 mg, BID | | 8  (15.4) | | | 4  (7.8) | | 2.6  (0.7, 9.3) | | 13.2 |  |  |
| NCT03502616 ^44,45^  *r-axSpA*  *week 16* | Tofacitinib 5mg, BID, oral | | 9  (6.8) | | | 0 | | 19.4 ^cc^  (1.1, 330.4) | | 14.8 |  |  |
| SELECT-AXIS 1^46,47^  *r-axSpA*  *week 14* | Upadacitinib 15mg OD, oral | | 15  (16) | | | 0  (0) | | 31.3 ^cc^  (1.9, 516.1) | | 6.2 |  |  |
| SELECT-AXIS 2 ^48,49^  *nr-axSpA*  *week 14* | Upadacitinib 15mg OD, oral | | 22  (14) | | | 8  (5) | | 3.0  (1.3, 6.9) | | 11.1 |  |  |
| **ASDAS: Low disease activity (<2.1)** | | | | | | | | | | | | |
| NCT01786668 ^43^  *r-axSpA*  *week 12* | Tofacitinib 2 mg, BID; oral | | 21  (40.4) | | | 10  (19.6) | | 2.3  (1.2, 4.5) | | 4.8 |  | **HIGH confidence**  4 RCTs, all low risk of bias. Consistent findings and with sufficient precision: all >50 pts per arm, most with fairly narrow confidence intervals (total sample size: n= 976). No strong reason to downgrade for risk of bias, inconsistency, or imprecision.  **Conclusion**:  A significantly larger proportion of pts with AxSpA receiving JAKi meet ASDAS criteria for low disease activity compared to placebo. RR: range 2.0 to 4.8  NNT: range 2.6 to 5.9 |
|  | Tofacitinib 5mg, BID, oral | | 28  (53.9) | | | 10  (19.6) | | 3.1  (1.6, 5.8) | | 2.9 |  |  |
|  | Tofacitinib 10 mg, BID | | 19  (36.5) | | | 10  (19.6) | | 2.0  (1.0, 3.9) | | 5.9 |  |  |
| NCT03502616 ^44,45^  *r-axSpA*  *week 16* | Tofacitinib 5mg, BID, oral | | 51  (38.9) | | | 11  (8.1) | | 4.8  (2.6, 8.8) | | 3.2 |  |  |
| SELECT-AXIS 1^46,47^  *r-axSpA*  *week 14* | Upadacitinib 15mg OD, oral | | 46  (49) | | | 10  (11) | | 4.7  (2.5, 8.7) | | 2.6 |  |  |
| SELECT-AXIS 2 ^48,49^  *nr-axSpA*  *week 14* | Upadacitinib 15mg OD, oral | | 66  (42) | | | 28  (18) | | 2.3  (1.6, 3.4) | | 4.2 |  |  |
| **BASDAI50** | | | | | | | | | | | | |
| NCT01786668 ^43^  *r-axSpA*  *week 12* | Tofacitinib 2 mg, BID; oral | | 24  (46.2) | | | 12  (23.5) | | 2.1  (1.2, 3.9) | | 4.4 |  | **HIGH confidence**  4 RCTs, all low risk of bias. Consistent findings and with sufficient precision: all >50 pts per arm, all with fairly narrow confidence intervals (total sample size: n= 976). No reason to downgrade for risk of bias, inconsistency, or imprecision.  **Conclusion**:  A significantly larger proportion of pts with AxSpA receiving JAKi meet BASDAI-50 criteria for disease activity compared to placebo.  RR: range 1.9 to 2.4  NNT: range 4.0 to 5.3 |
|  | Tofacitinib 5mg, BID, oral | | 26  (44.07) | | | 12  (23.5) | | 1.9  (1.0, 3.5) | | 5.3 |  |  |
|  | Tofacitinib 10 mg, BID | | 22  (37.9) | | | 12  (23.5) | | 1.9  (1.0, 3.5) | | 5.3 |  |  |
| NCT03502616 ^44,45^  *r-axSpA*  *week 16* | Tofacitinib 5mg, BID, oral | | 57  (42.9) | | | 24  (17.7) | | 2.4  (1.6, 3.7) | | 4 |  |  |
| SELECT-AXIS 1^46,47^  *r-axSpA*  *week 14* | Upadacitinib 15mg OD, oral | | 42  (45) | | | 22  (23) | | 1.9  (1.3, 3.0) | | 4.6 |  |  |
| SELECT-AXIS 2 ^48,49^  *nr-axSpA*  *week 14* | Upadacitinib 15mg OD, oral | | 66  (42) | | | 35  (22) | | 1.9  (1.4, 2.7) | | 5 |  |  |
| **PDE4 inhibitors** | | | | | | | | | | | | |
| **ASAS20** | | | | | | | | | | | | |
| **^¥^**$POSTURE**^50^**  *r-axSpA*  *week 16* | | Apremilast, 20 mg, BID**,** Oral | | 57  (35) | 60  (37) | | 1.0  (0.7, 1.3) | | -61.9 | |  | **Very low confidence**  1 RCT with a total sample size = 654, with some concerns of risk of bias. Downgraded for risk of bias, inconsistency one RCT only), and imprecision (wide confidence intervals). Effects of Apremilast vs placebo are not statistically significant.  **Conclusion:**  There is insufficient evidence to make any conclusion regarding the effectiveness of PDE4i on disease activity (ASAS20). |
|  |  | **^Ə2^** Apremilast, 30 mg, BID, Oral | | 53  (33) | 60  (37) | | 0.9  (0.7, 1.2) | | -24.6 | |  |  |
| **ASAS40** | | | | | | | | | | | | |
| $POSTURE**^50^**  *r-axSpA*  *week 24* | | Apremilast, 20 mg, BID**,** Oral | | 32  (19.6) | 30 (18.3) | | 1.1  (0.7, 1.7) | | 74.7 | |  | **Very low confidence**  1 RCT with a total sample size = 654, with some concerns of risk of bias. Downgraded for risk of bias, inconsistency (one stud only), and imprecision (wide confidence intervals). Effects of Apremilast vs placebo are not statistically significant.  **Conclusion:**  There is insufficient evidence to make any conclusive decision about the effectiveness of PDE4i on disease activity (ASAS40). |

RR: Relative risk; CI: confidence interval; NNT: Number needed to treat; **^¥^**Primary outcome*;* $phase 2 trial; a*: TNFi – naïve group; b*: TNFi – Inadequate response group. Studies are grouped into a and b only if the outcome is reported by TNFi status; r: radiographic axSpA; nr: non-radiographic axSpA; LD: loading dose; NL: No loading dose; BID – twice a day; BIW – twice weekly, OW – Once weekly; Q2W - Once every two weeks; Q4W - Once every four weeks; OD: once daily; SD: single dose; **^ν^**day 1, and at wks - 8, 16 and 24; SC: Subcutaneous; IV: Intravenous; Risk of bias: Green – Low, Amber – Unclear, Red – High.

Interventional drugs: ADA - Adalimumab; BKZ - Bimekizumab; BRO- Brodalumab; CZP- Certolizumab; ETN- Etanercept; GOL- Golimumab; IXE- Ixekizumab; NTK- Netakimab; RIS- Risankizumab; SAR- Sarilumab; Sec- Secukinumab.

### Table 3 - Efficacy outcomes - Continuous data (Q1)

| **Study ID**  *Population*  *Time point* | | **Treatment arm (n)** | **Mean Change from baseline**  **(SD/SE^95% CI^^)** | **Standardised Mean Difference (CfB)**  **(95% CI)** | **Risk of bias** | **Quality of evidence** |
| --- | --- | --- | --- | --- | --- | --- |
| **bDMARDs: TNF inhibitors vs Placebo** | | | | | | |
| **ASDAS – CRP mean score (change from baseline - CfB)** | | | | | | |
| COAST -V^2^  *r-axSpA*  *week 16* | | Placebo (87) | -0·5  (0·10^) | **Ref** |  | **Moderate confidence**: TNFi reduce disease activity in people with AxSPA (reduction in mean ASDAS-CRP score)  8 RCTs, 6 considered low risk of bias.  Findings are in favour of TNFi compared to placebo in 7 studies, with larger effect sizes for ADA and GOL than for ETN, but not consistently. Direction is reversed (but not significant) for ETN in one small study. (Downgraded for inconsistency).  4 RCTs include at least 50 pts per arm (total sample size across all arms: n= 1,060). |
|  |  | ADA**,** 40 mg, Q2W, SC (90) | -1·3  (0·1^) | -0.8  (-1.1, -0.5) |  |  |
| DANISH^3^  r-axSpA  *week 12* | | Placebo (27) | -0.6  (1.2) | **Ref** |  |  |
|  |  | ADA, 40 mg, Q2W, SC (25) | -1.9  (1.7) | -0.9  (-1.5, -0.3) |  |  |
| EMBARK^5^  *nr-axSpA*  *week 12* | | Placebo (109) | -0.5  (0.1^) | **Ref** |  |  |
|  |  | ETN, 50 mg, OW, SC (106) | -1.1  (0.1^) | -0.6  (-0.9, -0.3) |  |  |
| PrevAS^7^  *nr-axSpA*  *week 16* | | Placebo (40) | -0.6  (1.5) | **Ref** |  |  |
|  |  | ETN, 25 mg, BIW, SC (40) | -0.3  (0.9) | 0.2  (-0.2, 0.7) |  |  |
| **^¥^**SPARSE^8^  *r-axSpA*  *week 8* | | Placebo (45) | -0.5  (0.1^) | **Ref** |  |  |
|  |  | ETN, 50 mg, OW, SC (41) | -1.2  (0.1^) | -1.1  (-1.5, -0.6) |  |  |
| GO-AHEAD^9^  *nr-axSpA*  *week 16* | | Placebo (90) | −0.6  (1.1) | **Ref** |  |  |
|  |  | GOL, 50 mg, Q4W, SC (88) | −1.7  (1.3) | -0.9  (-1.2, -0.6) |  |  |
| GO-ALIVE^10^  *r-axSpA*  *week 16* | | Placebo (102) | -0.4  (0.8) | **Ref** |  |  |
|  |  | GOL, 2 mg/kg, Q4W, IV (104) | -2.0  (1.0) | -1.8  (-2.1, -1.4) |  |  |
| NCT01212653^11^  r-axSpA  week 24 | | Placebo (21) | -0.4  (0.8) | **Ref** |  |  |
|  |  | GOL, 50 mg, Q4W, SC (20) | -1.7  (1.3) | -1.2  (-1.9, -0.5) |  |  |
| **BASDAI mean score (change from baseline)** | | | | | | |
| COAST -V^2^  *r-axSpA*  *week 16* | | Placebo (87) | -1.5  (1.7) | **Ref** |  | **Low confidence**: TNFi may reduce disease activity in people with AxSPA (reduction in mean BASDAI score)  7 RCTs, 5 considered low risk of bias.  Findings are in favour of TNFi compared to placebo in 6 studies; effect sizes vary and are smaller for ETN. (Downgraded for inconsistency)  Only 3 RCTs include at least 50 pts per arm (total sample size across all arms: n=852). (Downgraded for precision.) |
|  |  | ADA**,** 40 mg, Q2W, SC (90) | -2.4  (2.3) | -0.4  (-0.7, -0.1) |  |  |
| DANISH^3^  r-axSpA  *week 12* | | Placebo (27) | -11  (19) | **Ref** |  |  |
|  |  | ADA, 40 mg, Q2W, SC (25) | -24  (24) | -0.6  (-1.2, 0.0) |  |  |
| EMBARK^5^  *nr-axSpA*  *week 12* | | Placebo (109) | -1.3  (0.3^) | **Ref** |  |  |
|  |  | ETN, 50 mg, OW, SC (106) | -2.0  (0.3^) | -0.2  (-0.5, 0.0) |  |  |
| PrevAS^7^  *nr-axSpA*  *week 16* | | Placebo (40) | -1.1  (2.7) | **Ref** |  |  |
|  |  | ETN, 25 mg, BIW, SC (40) | -0.8  (1.8) | 0.1  (-0.3, 0.6) |  |  |
| SPARSE^8^  *r-axSpA*  *week 8* | | Placebo (45) | -1.1  (0.3^) | **Ref** |  |  |
|  |  | ETN, 50 mg, OW, SC (41) | -2  (0.3^) | -0.5  (-0.9, 0.0) |  |  |
| GO-AHEAD^9^  *nr-axSpA*  *week 16* | | Placebo (96) | −1.6  (2.5) | **Ref** |  |  |
|  |  | GOL, 50 mg, Q4W, SC (93) | −3.7  (2.5) | -0.8  (-1.1, -0.5) |  |  |
| NCT01212653^11^  r-axSpA  week 24 | | Placebo (21) | -0.7  (1.2) | **Ref** |  |  |
|  |  | GOL, 50 mg, Q4W, SC (20) | -1.8  (1.6) | -0.8  (-1.4, -0.2) |  |  |
| **Spinal pain mean score (change from baseline)** | | | | | | |
| COAST -V^2^  *r-axSpA*  *week 16* | | Placebo (87) | -1.7 | Ref |  | **Moderate confidence**: TNFi may reduce spinal pain in people with AxSPA  5 RCTs, all considered low risk of bias.  Findings are in favour of TNFi compared to placebo in 4 studies, but effect sizes vary and are not significant in most studies. (Downgraded for inconsistency)  3 RCTs include at least 50 pts per arm (total sample size across all arms: n=759. |
|  |  | ADA**,** 40 mg, Q2W, SC (90) | -2.1 | -0.5  (-0.8, -0.2) |  |  |
| EMBARK^5^  *nr-axSpA*  *week 12* | | Placebo (109) | -1.1  (0.3^) | **Ref** |  |  |
|  |  | ETN, 50 mg, OW, SC (106) | -2.0  (0.3^) | -0.3  (-0.6, 0.0) |  |  |
| PrevAS^7^  *nr-axSpA*  *week 16* | | Placebo (40) | -1.4  (2.7) | **Ref** |  |  |
|  |  | ETN, 25 mg, BIW, SC (40) | -0.9  (2.7) | 0.2  (-0.2, 0.6) |  |  |
| SPARSE^8^  *r-axSpA*  *week 8* | | Placebo (45) | -1.0  (0.4^) | **Ref** |  |  |
|  |  | ETN, 50 mg, OW, SC (41) | -2.2  (0.4^) | -0.5  (-0.9, 0.0) |  |  |
| GO-AHEAD^9^  *nr-axSpA*  *week 16* | | Placebo (97) | −1.9  (3.0) | **Ref** |  |  |
|  |  | GOL, 50 mg, Q4W, SC (93) | −4.2  (2.9) | -0.8  (-1.1, -0.5) |  |  |
| **bDMARDs: TNF inhibitors – head-to-head** | | | | | | |
| **ASDAS – CRP mean score (change from baseline - CfB)** | | | | | | |
| **^¥^**NCT02489760 ^12^  *r-axSpA*  *week 8* | | ADA, 40 mg, Q2W, SC (9) | -1.2  (1.4) | **Ref** |  | **Very low confidence:**  One very small trial (n= 19, high risk of bias) shows no significant difference between ADA and ETN for ASDAS-CRP mean score.  (Downgraded for study limitations, inconsistency, precision). |
|  |  | ETN, 25 mg, BIW, SC (10) | -0.9  (1.4) | -0.2  (-1.1, 0.7) |  |  |
| **BASDAI** | | | | | | |
| **^¥^**NCT02489760 ^12^  *r-axSpA*  *week 8* | | ADA, 40 mg, Q2W, SC | -2.2  (2.4) | **Ref** |  | **Very low confidence:**  One very small trial (n= 19, high risk of bias) shows no significant difference between ADA and ETN for BASDAI mean score.  (Downgraded for study limitations, inconsistency, precision). |
|  |  | ETN, 25 mg, BIW, SC | -1.6  (3.1) | -0.2  (-0.1, 0.7) |  |  |
| **bDMARDs: TNF inhibitors – biosimilars vs originators** | | | | | | |
| **ASDAS – CRP mean score (change from baseline - CfB)** | | | | | | |
| ChiCTR1900022520^13^  r-axSpA  *week 24* | | ADA, 40 mg, Q2W, SC (232) | Fig 3, data only presented in the graph | NS |  | **Moderate confidence:** biosimilars of ADA are likely to have similar effect on disease activity in AxSPA (ASDAS-CRP mean score)  2 RCTs, 1 low, 1 unclear risk of bias.  Both studies report similar ASDAS scores for biosimilars compared to ADA.  Both trials include more than 100 per arm (total sample size across arms: 1,028 pts), but estimates including confidence intervals are not reported. (Downgraded for precision). |
|  |  | HS016, 40 mg, Q2W, SC (416) |  |  |  |  |
| CTR20181863^14^  r-axSpA  *week 24* | | ADA, 40 mg, Q2W, SC (192) | NR | NS^±^ |  |  |
|  |  | TQ-Z2301, 40 mg, Q2W, SC (188) |  |  |  |  |
| **BASDAI** | | | | | | |
| NCT02893254^12^  *r-axSpA*  *week 24* | | ADA, 40 mg, Q2W, SC (218) | -3·8  (2.0) | **Ref** |  | **Moderate confidence**: biosimilars of ADA are likely to have similar effect on disease activity in AxSPA (BASDAI mean score)  1 RCT, low risk of bias.  This trial reports only very small differences in mean BASDAI score for biosimilars compared to ADA. (One trial only, downgraded for consistency)  This trial includes more than 100 per arm (total sample size across arms: n= 438) |
|  |  | IBI303, 40 mg, Q2W, SC (220) | -3·8  (2·1) | **0·1  (-0·2, 0·5) |  |  |
| NCT04345458 ^16^  *r-axSpA*  *week 24* | | Prefilled liquid Yisaipu, 25mg, BIW | **^%^**72.7  (50.0, 86.0) | n/a |  | **Very low confidence:**  One trial (n≥140 in each arm, high/unclear risk of bias) shows similar BASDAI mean scores for lyophilised Yisaipu versus either pre-filled liquid 50 or 25mg Yisaipu. No effect estimates with 95% confidence interval presented.  (Downgraded for study limitations, inconsistency, precision). |
|  |  | Lyophilised Yisaipu, 25 mg, BIW | **^%^**68.3  (45.8, 86.6) |  |  |  |
| NCT04345458 ^16^  *r-axSpA*  *week 24* | | Prefilled liquid Yisaipu, 50mg, OW | **^%^**70.6  (49.3, 88.0) | n/a |  |  |
|  |  | Lyophilised Yisaipu, 25 mg, BIW | **^%^**68.3  (45.8, 86.6) |  |  |  |
| **Spinal pain** | | | | | | |
| CTR20181863^14^  r-axSpA  *week 24* | | ADA, 40 mg, Q2W, SC (192) | NR | NS^±^ |  | **Very low confidence:** biosimilars of ADA may have similar effect on spinal pain in AxSPA (mean score)  RCT, unclear risk of bias (downloaded for study limitations).  The study reports no significant difference in spinal pain for biosimilars compared to ADA. (One study only, downgraded for consistency)  The trial includes more than 100 per arm (total sample size across arms: n= 380), but estimates including confidence intervals are not reported. (Downgraded for precision). |
|  |  | TQ-Z2301, 40 mg, Q2W, SC (188) |  |  |  |  |
| NCT04345458 ^16^  *r-axSpA*  *week 24* | | Prefilled liquid Yisaipu, 25mg, BIW | **^%^**71.4  (50.0, 86.7) | n/a |  | **Very low confidence:**  One trial (n≥140 in each arm, high/unclear risk of bias) shows similar spinal pain mean scores for lyophilised Yisaipu versus either pre-filled liquid 50 or 25mg Yisaipu. No effect estimates with 95% confidence interval presented.  (Downgraded for study limitations, inconsistency, precision). |
|  |  | Lyophilised Yisaipu, 25 mg, BIW | **^%^**66.7  (42.2, 85.7) |  |  |  |
| NCT04345458 ^16^  *r-axSpA*  *week 24* | | Prefilled liquid Yisaipu, 50mg, OW | **^%^**66.7  (40.0, 87.5) | n/a |  |  |
|  |  | Lyophilised Yisaipu, 25 mg, BIW | **^%^**66.7  (42.2, 85.7) |  |  |  |
| **IL-17 inhibitors** | | | | | | |
| **ASDAS – CRP mean score (change from baseline - CfB)** | | | | | | |
| $BE AGILE ^17^  *r-axSpA*  *week 12* | | Placebo (60) | -0.4  (0.7) | **Ref** |  | **High confidence**  6 RCTs, all but one judged as low risk of bias and with consistent findings. Precision is high, with >50 pts per arm (total sample size across arms: n= 1,940).  However, there is very low confidence for Netakimab, evaluated in one phase 2 trial, downgraded for risk of bias, inconsistency, and imprecision due to small sample size.  **Conclusion**:  Pts with AxSpA receiving IL-17 inhibitors show a larger mean reduction in ASDAS score compared to placebo. The standardised mean difference (effect size) is estimated between 0.5 (moderate) and 1.4 (large) in favour of IL-17 inhibitors. |
|  |  | **BKZ,** 16 mg, Q4W, SC (61) | -0.9  (1.0) | -0.6  (-0.9, -0.2) |  |  |
|  |  | **BKZ,** 64 mg, Q4W, SC (61) | -1.7  (1.1) | -1.4  (-1.8, -1.0) |  |  |
|  |  | **BKZ,** 160 mg, Q4W, SC (61) | -1.4  (0.9) | -1.2  (-1.6, -0.8) |  |  |
|  |  | **BKZ,** 320 mg, Q4W, SC (61) | -1.5  (0.9) | -1.4  (-1.8, -1.0) |  |  |
| NCT02985983 ^19^  *mixed*  *week 16* | | Placebo (79) | -0.7  (-0.9, -0.5) ^^ | **Ref** |  |  |
|  |  | **BRO**, 210 mg, 3 QW, then Q2W, SC (80) | -1.1  (-1.3, -0.9) ^^ | -0.5  (-0.8, -0.2) |  |  |
| COAST -V ^2^  *r-axSpA*  *week 16* | | Placebo (87) | -0·5  (0·10^) | **Ref** |  |  |
|  |  | **IXE,** 80 mg, Q2W, SC (83) | -1·37  (0·10^) | -0.9  (-1.3, -0.6) |  |  |
|  |  | **IXE,** 80 mg, Q4W, SC (81) | -1·4  (0·10^) | -0.9  (-1.2, -0.6) |  |  |
| COAST -W ^20,21^  *r-axSpA*  *week 16* | | Placebo (104) | -0.1  (0.1^) | **Ref** |  |  |
|  |  | **IXE,** 80 mg, Q2W, SC (98) | -1.2  (0.1^) | -1.0  (-1.3, -0.7) |  |  |
|  |  | **IXE,** 80 mg, Q4W, SC (114) | -1.1  (0.1^) | -1.0  (-1.3, -0.7) |  |  |
| COAST - X^22,23^  *nr-axSpA*  *week 16* | | Placebo (105) | -0.6  (0.1^) | **Ref** |  |  |
|  |  | **IXE**, 80 mg, Q2W, SC (105) | -1.3  (0.1^) | -0.7  (-0.9, -0.4) |  |  |
|  |  | **IXE**, 80 mg, Q4W, SC (96) | -1.1  (0.1^) | -0.5  (-0.8, -0.3) |  |  |
| $AILAS^24^  *r-axSpA*  *week 16* | | Placebo (22) | -0.5  (0.9) | **Ref** |  |  |
|  |  | **NTK**, 40 mg, 3 QW, Q2W, SC (22) | -2.0  (0.9) | -1.8  (-2.5, -1.1) |  |  |
|  |  | **NTK**, 80 mg, 3 QW, Q2W, SC (22) | -1.8  (1.2) | -1.3  (-1.9, -0.6) |  |  |
|  |  | **NTK**, 120 mg, 3 QW, Q2W, SC (22) | -1.9  (0.9) | -1.6  (-2.3, -0.9) |  |  |
| **BASDAI** | | | | | | |
| $BE AGILE^17^  *r-axSpA*  *week 12* | | Placebo (60) | -1.0  (1.7) | **Ref** |  | **High confidence**  14 RCTs, all but one judged low risk of bias, with consistent findings and high precision – nearly all RCTs more than 50 pts per arm (total sample size across all arms: n= 5,251).  However, there is very low confidence for Netakimab, evaluated in one phase 2 trial, downgraded for risk of bias, inconsistency, and imprecision due to small sample size.  **Conclusion**:  Pts with AxSpA receiving IL-17 inhibitors show a larger mean reduction in BASDAI score compared to placebo. The standardised mean difference (effect size) is estimated between 0.3 (small) and 1.0 (large) and is statistically significant for most comparisons, in favour of IL-17 inhibitors. |
|  |  | **BKZ,** 16 mg, Q4W, SC (61) | -1.7  (2.3) | -0.3  (-0.7, 0.0) |  |  |
|  |  | **BKZ,** 64 mg, Q4W, SC (61) | -2.7  (2.2) | -0.9  (-1.2, -0.5) |  |  |
|  |  | **BKZ,** 160 mg, Q4W, SC (61) | -2.5  (1.8) | -0.9  (-1.2, -0.5) |  |  |
|  |  | **BKZ,** 320 mg, Q4W, SC (61) | -2.9  (2.2) | -1.0  (-1.3, -0.6) |  |  |
| BE MOBILE 1^18^  *nr-axSpA*  *week 16* | | Placebo (126) | -1.5  (0.2) | **Ref** |  |  |
|  |  | **BKZ,** 160 mg, Q4W, SC (128) | -3.1  (0.2) | -8.0  (-8.7, -7.3) |  |  |
| BE MOBILE 2^18^  *r-axSpA*  *week 16* | | Placebo (111) | -1.9  (0.2) | **Ref** |  |  |
|  |  | **BKZ,** 160 mg, Q4W (221) | -2.9  (0.1) | -7.1  (-7.7, -6.5) |  |  |
| NCT02985983^19^  *mixed*  *week 16* | | Placebo (79) | –2.4  (1.9) | **Ref** |  |  |
|  |  | **BRO**, 210 mg, 3 QW, then Q2W, SC (80) | –2.9  (2.1) | -0.3  (-0.6, 0.1) |  |  |
| COAST -V^2^  *r-axSpA*  *week 16* | | Placebo (87) | -1.5  (1.7) | **Ref** |  |  |
|  |  | **IXE,** 80 mg, Q2W, SC (83) | -2.7  (2.1) | -0.6  (-0.9, -0.3) |  |  |
|  |  | **IXE,** 80 mg, Q4W, SC (81) | -3.0  (2.4) | -0.7  (-1.0, -0.4) |  |  |
| COAST -W ^20,21^  *r-axSpA*  *week 16* | | Placebo (104) | -0.9  (0.2^) | **Ref** |  |  |
|  |  | **IXE,** 80 mg, Q2W, SC (98) | -2.1  (0.2^) | -0.6  (-0.9, -0.3) |  |  |
|  |  | **IXE,** 80 mg, Q4W, SC (114) | -2.2  (0.2^) | -0.6  (-0.9, -0.3) |  |  |
| COAST - X^22,23^  *nr-axSpA*  *week 16* | | Placebo (105) | -1.5  (0.2^) | **Ref** |  |  |
|  |  | **IXE**, 80 mg, Q2W, SC (105) | -2.5  (0.2^) | -0.5  (-0.7, -0.2) |  |  |
|  |  | **IXE**, 80 mg, Q4W, SC (96) | -2.2  (0.2^) | -0.3  (-0.6, 0.0) |  |  |
| $AILAS^24^  *r-axSpA*  *week 16* | | Placebo (22) | -1.4  (1.6) | **Ref** |  |  |
|  |  | **NTK**, 40 mg, 3 QW, Q2W, SC (22) | -2.6  (2.2) | -0.6  (-1.2, 0.0) |  |  |
|  |  | **NTK**, 80 mg, 3 QW, Q2W, SC (22) | -3.5  (2.5) | -1.0  (-1.6, -0.4) |  |  |
|  |  | **NTK**, 120 mg, 3 QW, Q2W, SC (22) | -3.7  (2.4) | -1.1  (-1.8, -0.5) |  |  |
| MEASURE 1^26-30^  *r-axSpA*  *week 16* | | Placebo (122) | -0.6  (0.2^) | **Ref** |  |  |
|  |  | **SEC,** 75 mg, Q4W (starting W 8), SC (124) | -2.3  (0.2^) | -0.9  (-1.1, -0.6) |  |  |
|  |  | **SEC,** 150 mg, Q4W (starting W 8), SC (125) | -2.32  (0.17^) | -0.9  (-1.2, -0.6) |  |  |
| MEASURE 2a^31-33^  *r-axSpA*  *week 16* | | Placebo (42) | -1.2  (0.3^) | **Ref** |  |  |
|  |  | **SEC,** 75 mg, Q4W, SC (45) | -2.3  (0.3^) | -0.6  (-1.0, -0.1) |  |  |
|  |  | **SEC,** 150 mg, Q4W, SC (45) | -2.6  (0.3^) | -0.7  (-1.1, -0.3) |  |  |
| MEASURE 2b^31-33^  *r-axSpA*  *week 16* | | Placebo (22) | -0.6  (0.4^) | **Ref** |  |  |
|  |  | **SEC,** 75 mg, Q4W, SC (22) | -1.4  (0.4^) | -0.4  (-1.0, 0.2) |  |  |
|  |  | **SEC,** 150 mg, Q4W, SC (24) | -1.6  (0.4^) | -0.2  (-0.8, 0.4) |  |  |
| MEASURE 3a^34, 35^  *r-axSpA*  *week 16* | | Placebo (59) | -1.9  (0.3^) | **Ref** |  |  |
|  |  | **SEC,** 150 mg, Q4W, IV (57) | -2.6  (0.3^) | -0.3  (-0.7, 0.1) |  |  |
|  |  | **SEC**, 300 mg, Q4W, IV (57) | -3.2  (0.3^) | -0.6  (-0.9, -0.2) |  |  |
| MEASURE 3b^34, 35^  *r-axSpA*  *week 16* | | Placebo (17) | -0.9  (0.6^) | **Ref** |  |  |
|  |  | **SEC,** 150 mg, Q4W, IV (17) | -2.2  (0.6^) | -0.5  (-1.2, 0.2) |  |  |
|  |  | **SEC**, 300 mg, Q4W, IV (19) | -1.8  (0.6^) | -0.4  (-1.0, 0.3) |  |  |
| MEASURE 4a^36^  *r-axSpA*  *week 16* | | Placebo (83) | -2.00  (0.23^) | **Ref** |  |  |
|  |  | **SEC,** 150 mg, Q4W (LD), SC (85) | -2.54  (0.23^) | -0.3  (-0.6, 0.0) |  |  |
|  |  | **SEC,** 150 mg, Q4W (NL), SC (85) | -2.65  (0.23^) | -0.3  (-0.6, 0.0) |  |  |
| MEASURE 4b^36^  *r-axSpA*  *week 16* | | Placebo (34) | -1.57 | **Ref** |  |  |
|  |  | **SEC,** 150 mg, Q4W (LD), SC (31) | -2.08  (0.42^) | -0.2  (-0.7, 0.3) |  |  |
|  |  | **SEC,** 150 mg, Q4W (NL), SC (32) | -2.42  (0.42^) | -0.4  (-0.8, 0.1) |  |  |
| MEASURE 5a^37^  *r-axSpA*  *week 16* | | Placebo (122) | -1.47  (0.21^) | **Ref** |  |  |
|  |  | **SEC,** 150 mg, Q4W, SC (240) | -2.69  (0.15^) | -0.5  (-0.7, -0.3) |  |  |
| MEASURE 5b^37^  *r-axSpA*  *week 16* | | Placebo (31) | -1.73  (0.41^) | **Ref** |  |  |
|  |  | **SEC,** 150 mg, Q4W, SC (65) | -3.26  (0.30^) | -0.6  (-1.1, -0.2) |  |  |
| PREVENT^38^  *nr-axSpA*  *week 16* | | Placebo (186) | -1.46  (0.21^) | **Ref** |  |  |
|  |  | **SEC,** 150 mg, LD, 4 QW, then Q4W, SC (185) | -2.35  (0.20^) | -0.3  (-0.5, -0.1) |  |  |
|  |  | **SEC**,150 mg, NL, 4 QW, then Q4W, SC (184) | -2.43  (0.20^) | -0.3  (-0.6, -0.1) |  |  |
| **Spinal pain** | | | | | | |
| $BE AGILE^17^  *r-axSpA*  *week 12* | | Placebo (60) | -1.1  (1.7) | **Ref** |  | **High confidence**  7 RCTs, all low risk of bias, with consistent findings and high precision – nearly all RCTs more than 50 pts per arm (total sample size across all arms: n= 2,394).  **Conclusion**:  Pts with AxSpA receiving IL-17 inhibitors show a significantly larger mean reduction in spinal pain score compared to placebo. The standardised mean difference is estimated between 0.3 (small) and 1.6 (large) compared to placebo. |
|  |  | **BKZ,** 16 mg, Q4W, SC (61) | -2.2  (2.4) | -0.5  (-0.9, -0.2) |  |  |
|  |  | **BKZ,** 64 mg, Q4W, SC (61) | -3.3  (2.5) | -1.1  (-1.4, -0.7) |  |  |
|  |  | **BKZ,** 160 mg, Q4W, SC (61) | -2.6  (2.2) | -0.8  (-1.1, -0.4) |  |  |
|  |  | **BKZ,** 320 mg, Q4W, SC (61) | -3.6  (2.4) | -1.2  (-1.6, -0.8) |  |  |
| BE MOBILE 1^18^  *nr-axSpA*  *week 16* | | Placebo (126) | -1.7  (0.2^) | Ref |  |  |
|  |  | **BKZ,** 160 mg, Q4W, SC (128) | -3.4  (0.2^) | -0.8  (-1.0, -0.5) |  |  |
| BE MOBILE 2^18^  *r-axSpA*  *week 16* | | Placebo (111) | -1.9  (0.2^) | Ref |  |  |
|  |  | **BKZ,** 160 mg, Q4W, SC (221) | -3.3  (0.2^) | -0.5  (-0.7, -0.3) |  |  |
| NCT02985983^19^  *mixed*  *week 16* | | Placebo (79) | –2.3  (2.5) | **Ref** |  |  |
|  |  | **BRO**, 210 mg, 3 QW, then Q2W, SC (80) | –2.9  (2.3) | -0.3  (-0.6, 0.1) |  |  |
| COAST -V^2^  *r-axSpA*  *week 16* | | Placebo (87) | -1.7 | **Ref** |  |  |
|  |  | **IXE,** 80 mg, Q2W, SC (83) | -2.4 | -0.7  (-1.0, -0.4) |  |  |
|  |  | **IXE,** 80 mg, Q4W, SC (81) | -2.4 | -0.7  (-1.0, -0.4) |  |  |
| COAST -W^20,21^  *r-axSpA*  *week 16* | | Placebo (104) | -0.7 | **Ref** |  |  |
|  |  | **IXE,** 80 mg, Q2W, SC (98) | -2.1 | -0.6  (-0.9, -0.3) |  |  |
|  |  | **IXE,** 80 mg, Q4W, SC (114) | -2.4 | -0.5  (-0.8, -0.3) |  |  |
| COAST - X^22,23^  *nr-axSpA*  *week 16* | | Placebo (105) |  | **Ref** |  |  |
|  |  | **IXE**, 80 mg, Q2W, SC (105) | -2.7  (0.3^) | -0.4  (-0.6, -0.1) |  |  |
|  |  | **IXE**, 80 mg, Q4W, SC (96) | -2.4  (0.3^) | -0.3  (-0.6, 0.0) |  |  |
| **IL-6 inhibitors** | | | | | | |
| **ASDAS – CRP mean score (change from baseline - CfB)** | | | | | | |
| $ALIGN^39^  mixed *-axSpA*  *week 12* | | Placebo (50) | -0.4  (0.7) | **Ref** |  | **Very low confidence**  1 RCT with a total sample size = 501, considered low risk of bias. Reduction in the disease activity is noted in all the arms compared to the respective arm’s baseline level. Downgraded for inconsistency (one study only), and imprecision (wide confidence intervals). the effects of Sarilumab on ASDAS mean score are not statistically significant).  **Conclusion:**  There is insufficient evidence to make any conclusion regarding the effectiveness of IL-6i on disease activity (change in ASDAS mean score). |
|  |  | Sarilumab**,** 100 mg, Q2W, SC (49) | -0.5  (0.9) | -0.1  (-0.5, 0.3) |  |  |
|  |  | Sarilumab**,** 150 mg, Q2W, SC (50) | -0.8  (1.2) | -0.4  (-0.8, 0.0) |  |  |
|  |  | Sarilumab**,** 100 mg, QW, SC (52) | -1.1  (0.8) | -0.9  (-1.3, -0.5) |  |  |
|  |  | Sarilumab**,** 200 mg, Q2W, SC (50) | -1.2  (0.9) | -1.0  (-1.4, -0.6) |  |  |
|  |  | Sarilumab**,** 150 mg, QW, SC (50) | -1.6  (0.9) | -1.5  (-1.9, -1.0) |  |  |
| **BASDAI** | | | | | | |
| $ALIGN^39^  mixed *-axSpA*  *week 12* | | Placebo (50) | -0.9  (1.7) | **Ref** |  | **Very low confidence**  1 RCT with a total sample size = 501, considered low risk of bias. Reduction in the disease activity is noted in all the arms compared to the respective arm’s baseline level. Downgraded for inconsistency (one study only), and imprecision (wide confidence intervals). The effects of Sarilumab on BASDAI mean score are not statistically significant.  **Conclusion:**  There is insufficient evidence to make any conclusive decision about the effectiveness of IL-6i on disease activity (change in BASDAI mean score). |
|  |  | Sarilumab**,** 100 mg, Q2W, SC (49) | -0.8  (1.9) | 0.1  (-0.3, 0.5) |  |  |
|  |  | Sarilumab**,** 150 mg, Q2W, SC (50) | -1.1  (2.0) | -0.1  (-0.5, 0.3) |  |  |
|  |  | Sarilumab**,** 100 mg, QW, SC (52) | -0.4  (1.4) | 0.3  (-0.1, 0.7) |  |  |
|  |  | Sarilumab**,** 200 mg, Q2W, SC (50) | -0.9  (1.8) | 0.0  (-0.4, 0.4) |  |  |
|  |  | Sarilumab**,** 150 mg, QW, SC (50) | -1.2  (1.8) | -0.2  (-0.6, 0.2) |  |  |
| **Spinal pain** | | | | | | |
| $ALIGN^39^  mixed *-axSpA*  *week 12* | | Placebo (50) | -0.8  (1.8) | **Ref** |  | **Very low confidence**  1 RCT with a total sample size = 501, considered low risk of bias. Downgraded for inconsistency (one study only), and imprecision (wide confidence intervals. The effects of Sarilumab on mean spinal pain score are not statistically significant.  **Conclusion:**  There is insufficient evidence to make any conclusion regarding the effectiveness of IL-6i on disease activity. |
|  |  | Sarilumab**,** 100 mg, Q2W, SC (49) | -1.3  (2.2) | -0.2  (-0.6, 0.1) |  |  |
|  |  | Sarilumab**,** 150 mg, Q2W, SC (50) | -1.2  (2.4) | -0.2  (-0.6, 0.2) |  |  |
|  |  | Sarilumab**,** 100 mg, QW, SC (52) | -0.5  (1.8) | 0.2  (-0.2, 0.6) |  |  |
|  |  | Sarilumab**,** 200 mg, Q2W, SC (50) | -0.9  (2.2) | 0.0  (-0.4, 0.3) |  |  |
|  |  | Sarilumab**,** 150 mg, QW, SC (50) | -1.6  (2.1) | -0.4  (-0.8, 0.0) |  |  |
| **IL-23 inhibitors** | | | | | | |
| **ASDAS – CRP mean score (change from baseline - CfB)** | | | | | | |
| $NCT02047110**^40^**  *r-axSpA*  *week 12* | | Placebo (40) | -0.3  (-1.0, 0.2) ^^ | n/a |  | **Very low confidence**  1 RCT with a total sample size = 234, considered low risk of bias. Median mean of change from baseline is statistically significant across all the 4 arms, although the reduction is larger in the intervention arms compared to the placebo arm. Downgraded for inconsistency (one study only) and imprecision (sample size <50 per arm and no effect estimates presented, only within-arm mean change from baseline).  **Conclusion:**  There is insufficient evidence to make any conclsion regarding the effectiveness of IL-6i, IL-23I, and PDE4i on disease activity (change in ASDAS mean score). |
|  |  | Risankizumab, 18 mg, SD, SC (40) | -0.7  (-1.3, -0.2) ^^ | n/a |  |  |
|  |  | Risankizumab, 90 mg**^ν^,** SC (39) | -0.6  (-1.2, 0.0) ^^ | n/a |  |  |
|  |  | ^Ə1^ Risankizumab, 180 mg**^ν^**, SC (40) | -0.7  (-1.1, -0.3) ^^ | n/a |  |  |
| **BASDAI** | | | | | | |
| $NCT02047110**^40^**  *r-axSpA*  *week 12* | | Placebo (40) | -0.7  (-3.0, -0.1) ^^ | n/a |  | **Very low confidence**  1 RCT with a total sample size = 234, considered low risk of bias. Median mean of change from baseline is statistically significant across all the 4 arms, although the reduction is larger in the intervention arms compared to the placebo arm. Downgraded for inconsistency (one study only) and imprecision (sample size <50 per arm and no effect estimates presented, only, within-arm mean change from baseline).  **Conclusion:**  There is insufficient evidence to make any conclusive decision about the effectiveness of IL-23i on disease activity (change in BASDAI mean score). |
|  |  | Risankizumab, 18 mg, SD, SC (40) | -1.3  (-2.8, -0.7) ^^ | n/a |  |  |
|  |  | Risankizumab, 90 mg**^ν^,** SC (39) | -1.0  (-2.2, 0.3) ^^ | n/a |  |  |
|  |  | ^Ə1^ Risankizumab, 180 mg**^ν^**, SC (40) | -1.0  (-2.0, -0.2) ^^ | n/a |  |  |
| **JAK inhibitors** | | | | | | |
| **ASDAS – CRP mean score (change from baseline - CfB)** | | | | | | |
| **^¥^**TORTUGA^41,42^  r-axSpA  week 12 | Placebo (58) | | -0.6  (0·8) | Ref |  | **HIGH confidence**  5 RCTs, all low risk of bias. Consistent findings and with sufficient precision: all >50 pts per arm, fairly narrow confidence intervals (total sample size: n= 1,092). No reason to downgrade for risk of bias, inconsistency, or imprecision.  **Conclusion**:  The ASDAS mean score shows a significantly larger improvement in pts with AxSpA receiving JAKi compared to placebo.  Mean difference in improvement: range 0.65 to 1.2 points (in favour of JAKi). |
|  | Filgotinib 200mg, OD, oral (58) | | -1.5  (1.0) | -1.0  (-1.4, -0.6) |  |  |
| NCT01786668^43^  *r-axSpA*  *week 12* | Placebo (51) | | -0.7  (0.1^) | **Ref** |  |  |
|  | Tofacitinib 2 mg, BID; oral (52) | | -1.2  (0.1^) | -0.7  (-1.1, -0.3) |  |  |
|  | Tofacitinib 5mg, BID, oral (52) | | -1.4  (0.1^) | -1.0  (-1.8, -0.6) |  |  |
|  | Tofacitinib 10 mg, BID, oral (52) | | -1.4  (0.1^) | -1.0  (-1.8, -0.6) |  |  |
| NCT03502616^44,45^  *r-axSpA*  *week 16* | Placebo (131) | | -0.4  (0.1^) | **Ref** |  |  |
|  | Tofacitinib 5mg, BID, oral (129) | | -1.3  (0.1^) | -1.2  (-1.5, -1.0) |  |  |
| SELECT-AXIS 1^46, 47^  *r-axSpA*  *week 14* | Placebo (94) | | -0.5  (-0.7, -0.4^^) | **Ref** |  |  |
|  | Upadacitinib 15mg OD, oral (93) | | -1.5  (-1.6, -1.3^^) | -0.9**  (-1.1, -0.7) |  |  |
| SELECT-AXIS 2^48,49^  *nr-axSpA*  *week 14* | Placebo (157) | | -0.7 | Ref |  |  |
|  | Upadacitinib 15mg OD, oral (156) | | -1.4 | -0.7**  (-0.9, -0.5) |  |  |
| **BASDAI** | | | | | | |
| TORTUGA^41,42^  r-axSpA  week 12 | Placebo (58) | | -1.4  (2.0) | **Ref** |  | **HIGH confidence**  3 RCTs, all low risk of bias.  Results are fairly consistent and with sufficient precision: all >50 pts per arm, fairly narrow confidence intervals (total sample size: n= 592). No strong reason to downgrade for risk of bias, inconsistency, or imprecision.  **Conclusion**:  The BASDAI mean score shows a significantly larger improvement in pts with AxSpA receiving JAKi compared to placebo.  Mean difference in improvement: range 0.37 to 0.72 points (in favour of JAKi). |
|  | Filgotinib 200mg, OD, oral (58) | | -2.4  (2.0) | -0.5  (-0.9, -0.1) |  |  |
| NCT01786668^43^  *r-axSpA*  *week 12* | Placebo (51) | | -1.9  (0.3^) | **Ref** |  |  |
|  | Tofacitinib 2 mg, BID; oral (52) | | -2.8  (0.3^) | -0.4  (-0.8, -0.0) |  |  |
|  | Tofacitinib 5mg, BID, oral (52) | | -2.9  (0.3^) | -0.5  (-0.9, -0.1) |  |  |
|  | Tofacitinib 10 mg, BID, oral (52) | | -2.7  (0.3^) | -0.4  (-0.8, 0.0) |  |  |
| NCT03502616^44,45^  *r-axSpA*  *week 16* | Placebo (131) | | -1.1  (0.2^) | **Ref** |  |  |
|  | Tofacitinib 5mg, BID, oral (129) | | -2.6  (0.2^) | -0.7  (1.0, -0.5) |  |  |
| **Spinal pain** | | | | | | |
| TORTUGA^9-11^  r-axSpA  week 12 | Placebo (58) | | -1.7  (2.2) | **Ref** |  | **Low confidence**  4 RCTs, all low risk of bias. All >50 pts per arm, but confidence intervals not provided or estimable for all (total sample size: n= 885).  Results are statistically significant in all studies but inconsistent in terms of magnitude. Downgraded for imprecision and inconsistency  **Conclusion**:  The mean spinal pain score shows a larger improvement in pts with AxSpA receiving JAKi compared to placebo.  Mean difference in improvement: range 0.41 to 1.03 points (in favour of JAKi). |
|  | Filgotinib 200mg, OD, oral (58) | | -2.7  (2.6) | -0.4  (-0.8, -0.1) |  |  |
| NCT03502616^44,45^  *r-axSpA*  *week 16* | Placebo (131) | | -1.3  (0.2^) | **Ref** |  |  |
|  | Tofacitinib 5mg, BID, oral (129) | | -2.9  (0.2^) | -0.7  (-0.9, -0.4) |  |  |
| SELECT-AXIS 1^46, 47^  *r-axSpA*  *week 14* | Placebo (94) | | -1.6 | **Ref** |  |  |
|  | Upadacitinib 15mg OD, oral (93) | | -3.3 | -1.0** |  |  |
| SELECT-AXIS 2^48,49^  *nr-axSpA*  *week 14* | Placebo (157) | | -1.8 | **Ref** |  |  |
|  | Upadacitinib 15mg OD, oral (156) | | -2.9 | -0.42**  (-0.8, -0.1) |  |  |
| **PDE4 inhibitors** | | | | | | |
| **ASDAS – CRP mean score (change from baseline - CfB)** | | | | | | |
| $POSTURE**^50^**  *r-axSpA*  *week 24* | | Placebo (164) | -0.4  (0.1^) | **Ref** |  | **Very low confidence**  1 RCT with a total sample size = 654, with some concerns of risk of bias. Reduction in the disease activity is noted in all the arms compared to the respective arm’s baseline level. Downgraded for risk of bias, inconsistency (one study only), and imprecision (wide confidence intervals). Effects of Apremilast compared to placebo are not statistically significant).  **Conclusion:**  There is insufficient evidence to make any conclusion regarding the effectiveness of PDE4i on disease activity (change in ASDAS mean score). |
|  |  | Apremilast, 20 mg, BID**,** Oral (163) | -0.5  (0.1^) | -0.1  (-0.3, 0.1) |  |  |
|  |  | Apremilast, 30 mg, BID, Oral (163) | -0.4  (0.1^) | 0.0  (-0.3, 0.2) |  |  |
| **BASDAI** | | | | | | |
| $POSTURE**^50^**  *r-axSpA*  *week 24* | | Placebo (164) | 1.2  (0.1^) | **Ref** |  | **Very low confidence**  1 RCT with a total sample size = 654, with some concerns of risk of bias. Mean change from baseline indicate reduction in the disease activity in both the intervention arms while there is an increase in the placebo arm. Downgraded for risk of bias, inconsistency (one study only), and imprecision (wide confidence intervals. The effects of Apremilast compared to placebo on BASDAI mean score are not statistically significant.  **Conclusion:**  There is insufficient evidence to make any conclusive decision about the effectiveness of PDE4i on disease activity (change in BASDAI mean score). |
|  |  | Apremilast, 20 mg, BID**,** Oral (163) | -1.3  (0.1^) | -0.1  (-0.3, 0.2) |  |  |
|  |  | Apremilast, 30 mg, BID, Oral (163) | -1.2  (0.1^) | 0.0  (-0.2, 0.2) |  |  |

SD – standard deviation; SE – Standard error; SE^ - SD is calculated using the formula – SE x square root (n); CI - Confidence interval. Standardised mean difference (SMD) is calculated using the ‘metan’ command in Stata; **^¥^**Primary outcome*;* $phase 2 trial; a*: TNFi – naïve group; b*: TNFi – Inadequate response group. Studies are grouped into a and b only if the outcome is reported by TNFi status; r: radiographic axSpA; nr: non-radiographic axSpA; LD: loading dose; NL: No loading dose; BID – twice a day; BIW – twice weekly, OW – Once weekly; Q2W - Once every two weeks; Q4W - Once every four weeks; OD: once daily; SD: single dose; **^ν^**day 1, and at wks - 8, 16 and 24; SC: Subcutaneous; IV: Intravenous; Risk of bias: Green – Low, Amber – Unclear, Red – High; NR- not reported; NS – nonsignificant; **as reported; ^±^ “Compared with baseline, overall back pain score, ASDAS-CRP, at weeks 12 and 24 were significantly improved (all P<0.0001) in both groups, and there was no statistical difference of these endpoints between two groups (P>0.05);” **^%^** improvement rate – median (range).

Interventional drugs: ADA - Adalimumab; BKZ - Bimekizumab; BRO- Brodalumab; CZP- Certolizumab; ETN- Etanercept; GOL- Golimumab; IXE- Ixekizumab; NTK- Netakimab; RIS- Risankizumab; SAR- Sarilumab; Sec- Secukinumab.

### Table 4: Safety outcomes (Q1)

| **Study** | **Drug** | **Response, n (%)** | | **RR (95% CI)** | **Risk of bias** | **Quality of evidence** |
| --- | --- | --- | --- | --- | --- | --- |
|  |  | **Intervention** | **Control** |  |  |  |
| **bDMARDs: TNF inhibitors vs Placebo** | | | | | | |
| **Patient with ≥1 AE** | | | | | | |
| COAST -V^2^  *r-axSpA*  *week 16* | ADA, 40 mg, Q2W, SC | 44  (49) | 34  (40) | 1.2  (0.9, 1.7) |  | **Moderate confidence:** TNFi are associated with an increased risk of adverse events (any) compared to placebo, although the risk varies.  8 RCTs, 6 considered low risk of bias.  Findings show higher proportion of people with at least 1 AE for TNFi compared to placebo in 7 studies, although statistically significant in only one study. One study shows similar rates compared to placebo; one study reports a reduced risk of AE.  (Downgraded for inconsistency).  6 RCTs include at least 50 pts per arm (total sample size across all arms: n=1,379). Studies possibly not powered to detect differences in AEs. |
| C-axSpAnd^4^  *nr-axSpA*  *week 12* | CZP, 400 mg, 3 LD**,** Q2W  then 200 mg**,** Q2W | 120  (75.5) | 101  (63.9) | 1.2  (1.0, 1.4) |  |  |
| EMBARK^5^  *nr-axSpA*  *week 12* | ETN, 50 mg, OW, SC | 63  (56.8) | 51  (45.1) | 1.3  (1.0, 1.6) |  |  |
| NCT01934933^6^  *r-axSpA*  *week 52* | ETN, 50 mg, OW, SC | 18  (36) | 13  (26) | 1.4  (0.8, 2.5) |  |  |
| PrevAS^7^  *nr-axSpA*  *week 16* | ETN, 25 mg, BIW, SC | 15  (39.5) | 15  (37.5) | 1.1  (0.6, 1.9) |  |  |
| SPARSE^8^  *r-axSpA*  *week 8* | ETN, 50 mg, OW, SC | 34  (81.0) | 26  (54.2) | 1.5  (1.1, 2.0) |  |  |
| GO-AHEAD^9^  *nr-axSpA*  *week 16* | GOL, 50 mg, Q4W, SC | 40  (41.2) | 47  (47.0) | 0.9  (0.6,1.2) |  |  |
| GO-ALIVE^10^  *r-axSpA*  *week 16* | GOL, 2 mg/kg, Q4W, IV | 34  (32.4) | 24  (23.3) | 1.4  (0.9, 2.2) |  |  |
| **Patient with ≥1 SAE** | | | | | | |
| COAST -V^2^  *r-axSpA*  *week 16* | ADA**,** 40 mg, Q2W, SC | 3  (3) | 0 | 6.7 ^cc^  (0.4, 127.7) |  | **Moderate confidence:** TNFi are unlikely to be associated with an increased risk of serious adverse events.  7 RCTs, 6 considered low risk of bias.  Findings show very low rates of SAE in all studies.  6 RCTs include at least 50 pts per arm (total sample size across all arms: n=1301). Studies are not powered to detect differences in SAEs resulting in very wide confidence intervals. (Downgraded for precision) |
| C-axSpAnd^4^  *nr-axSpA*  *week 12* | CZP, 400 mg, 3 LD**,** Q2W  then 200 mg**,** Q2W | 8  (5) | 3  (1.9) | 2.7  (0.7, 9.8) |  |  |
| EMBARK^5^  *nr-axSpA*  *week 12* | ETN, 50 mg, OW, SC | 2  (1.8) | 2  (1.8) | 1.0  (0.2, 7.2) |  |  |
| NCT01934933^6^  *r-axSpA*  *week 52* | ETN, 50 mg, OW, SC | 1  (0.7) | 0 | 3.0 ^cc^  (0.1, 71.9) |  |  |
| SPARSE^8^  *r-axSpA*  *week 8* | ETN, 50 mg, OW, SC | 1  (2.4) | 2  (4.2) | 0.6  (0.1, 6.1) |  |  |
| GO-AHEAD^9^  *nr-axSpA*  *week 16* | GOL, 50 mg, Q4W, SC | 1  (1.0) | 2  (2.0) | 0.5  (0.1, 5.6) |  |  |
| GO-ALIVE^10^  *r-axSpA*  *week 16* | GOL, 2 mg/kg, Q4W, IV | 2  (1.9) | 0 | 4.9 ^cc^  (0.2, 101) |  |  |
| **Patient with ≥1 infection** | | | | | | |
| COAST -V^2^  *r-axSpA*  *week 16* | ADA**,** 40 mg, Q2W, SC | 19  (21) | 13  (15) | 1.4  (0.8, 2.7) |  | **Moderate confidence**: TNFi are unlikely to be associated with an increased risk of infection compared to placebo.  6 RCTs, 5 considered low risk of bias.  Differences in infection rates appear to be small between TNFi and placebo in all studies.  5 RCTs include at least 50 pts per arm (total sample size across all arms: n=985). Studies are not powered to detect differences in infection rate. (Downgraded for precision) |
| EMBARK^5^  *nr-axSpA*  *week 12* | ETN, 50 mg, OW, SC | 11  (9.9) | 10  (8.8) | 1.1  (0.5, 2.6) |  |  |
| NCT01934933 ^6^  *r-axSpA*  *week 52* | ETN, 50 mg, OW, SC | - | - | Data by type of infections - |  |  |
| SPARSE^8^  *r-axSpA*  *week 8* | ETN, 50 mg, OW, SC | 11  (26.2) | 10  (20.8) | 1.3  (0.6, 2.7) |  |  |
| GO-AHEAD^9^  *nr-axSpA*  *week 16* | GOL, 50 mg, Q4W, SC | 0 | 0 | n/a |  |  |
| GO-ALIVE^10^  *r-axSpA*  *week 16* | GOL, 2 mg/kg, Q4W, IV | 12  (11.4) | 8  (7.8) | 1.5  (0.6, 3.5) |  |  |
| **EMM - Uveitis** | | | | | | |
| C-axSpAnd^4^  *nr-axSpA*  *week 12* | CZP, 400 mg, 3 LD**,** Q2W  then 200 mg**,** Q2W | 4  (2.5) | 8  (5.1) | 0.5  (0.2, 1.6) |  | **Low confidence:** There is insufficient evidence for the incidence of new onset uveitis in people treated with TNFi.  2 RCTs (n=525), considered at low risk of bias reported lower incidence of Uveitis in the TNFi arm but not statistically significant. (Downgraded for imprecision.)  Both the RCTs included 50 pts per arm, and was not powered to detect differences in EMMs. (Downgraded for precision) |
| GO-ALIVE^10^  *r-axSpA*  *week 16* | GOL, 2 mg/kg, Q4W, IV | 0 | 1  (0.7) | 0.3^cc^  (0.0, 7.9) |  |  |
| **EMM - IBD** | | | | | | |
| COAST -V^2^  *r-axSpA*  *week 16* | ADA**,** 40 mg, Q2W, SC | 0 | 0 | n/a |  | **Low confidence**: There is insufficient evidence for the incidence of IBD in people treated with TNFi.  1 RCT, considered at low risk of bias.  This trial reports there were no cases of IBD in either arm. (One study only, downgraded for inconsistency.)  This RCT included more than 50 pts per arm (n=176 in total), but was not powered to detect differences in EMMs. (Downgraded for precision) |
| **EMM - Psoriasis** | | | | | | |
| COAST -V^2^  *r-axSpA*  *week 16* | ADA, 40 mg, Q2W, SC | 1  (1) | 0 | n/a |  | Low confidence: There is insufficient evidence for the incidence of candida infection in people treated with TNFi.  1 RCT, considered at low risk of bias.  This trial reports only one case of psoriasis in the ADA arm. (One study only, downgraded for inconsistency.)  This RCT included more than 50 pts per arm (n=176 in total) but was not powered to detect differences in EMMs. (Downgraded for precision) |
| **Candida** | | | | | | |
| COAST -V^2^  *r-axSpA*  *week 16* | ADA, 40 mg, Q2W, SC | 1  (1) | 0 | n/a |  | **Low confidence**: There is insufficient evidence for the incidence of candida infection in people treated with TNFi.  1 RCT, considered at low risk of bias.  This trial reports only one case of candida in the ADA arm. (One study only, downgraded for inconsistency.)  This RCT included more than 50 pts per arm (n=176 in total) but was not powered to detect differences in EMMs. (Downgraded for precision) |
| **Tuberculosis** | | | | | | |
| C-axSpAnd^4^  *nr-axSpA*  *week 12* | CZP, 400 mg, 3 LD**,** Q2W  then 200 mg**,** Q2W | 0 | 0 | n/a |  | **Low confidence:** There is insufficient evidence for the incidence of new onset tuberculosis in people treated with TNFi.  3 RCT, considered at low risk of bias, reports no cases of new onset TB in either arm.  The RCT included more than 50 pts per arm (n= 604 in total but was not powered to detect differences in EMMs. (Downgraded for precision). |
| SPARSE^8^  *r-axSpA*  *week 8* | ETN, 50 mg, OW, SC | 0 | 0 | n/a |  |  |
| GO-AHEAD^9^  *nr-axSpA*  *week 16* | GOL, 50 mg, Q4W, SC | 0 | 0 | n/a |  |  |
| **CVD events** | | | | | | |
| COAST -V^2^  *r-axSpA*  *week 16* | ADA**,** 40 mg, Q2W, SC | 0 | 0 | n/a |  | **Low confidence**: There is insufficient evidence for the incidence of major cardiovascular events in people treated with TNFi.  2 RCTs, considered at low risk of bias, reports no cases of a major cardiovascular event in either arm.  The RCTs included more than 50 pts per arm (n=493 in total) but was not powered to detect differences in CVD events. (Downgraded for precision) |
| C-axSpAnd^4^  *nr-axSpA*  *week 12* | CZP, 400 mg, 3 LD**,** Q2W  then 200 mg**,** Q2W | 0 | 0 | n/a |  |  |
| **Malignancies** | | | | | | |
| C-axSpAnd^4^  *nr-axSpA*  *week 12* | CZP, 400 mg, 3 LD**,** Q2W  then 200 mg**,** Q2W | 2  (1.3) | 1  (0.6) | 2.0  (0.2, 21.7) |  | **Low confidence**: There is insufficient evidence for the incidence of major cardiovascular events in people treated with TNFi.  Of the 4 RCTs, considered at low risk of bias, with a total sample size of n=812, 3 RCTs reports no cases of a major cardiovascular event in either arm. One RCT reports higher incidence in the TNFi arm compared to the placebo arm, but the difference is not statistically significant.  The RCTs included more than 50 pts per arm but was not powered to detect differences in CVD events. (Downgraded for precision) |
| SPARSE^8^  *r-axSpA*  *week 8* | ETN, 50 mg, OW, SC | 0 | 0 | n/a |  |  |
| GO-AHEAD^9^  *nr-axSpA*  *week 16* | GOL, 50 mg, Q4W, SC | 0 | 0 | n/a |  |  |
| GO-ALIVE^10^  *r-axSpA*  *week 16* | GOL, 2 mg/kg, Q4W, IV | 0 | 0 | n/a |  |  |
| **bDMARDs: TNF inhibitors – biosimilars vs originators** | | | | | | |
| **Patient with ≥1 AE** | | | | | | |
| ChiCTR1900022520^13^  r-axSpA  *week 24* | HS016, 40 mg, Q2W, SC | 352  (84.6) | 200  (86.2) | 1.0  (0.9, 1.1) |  | **High confidence**: biosimilars have similar risk of adverse events as ADA in AxSPA (proportion with at least 1AE)  3 RCT, 2 with low risk of bias.  The trials all report a similar incidence of adverse effects (any) for both biosimilars and ADA.  All 3 RCTs include more than 100 pts per arm (larger sample size needed for non-superiority or equivalence (total sample size across all arms: n= 1,466). |
| CTR20181863^14^  r-axSpA  *week 24* | TQ-Z2301, 40 mg, Q2W, SC | 155  (82.5) | 161  (83.9) | 1.0  (0.9, 1.1) |  |  |
| NCT02893254^15^  *r-axSpA*  *week 24* | IBI303, 40 mg, Q2W, SC | 174  (79) | 178  (82) | 1.0  (0.9, 1.1) |  |  |
| NCT04345458^16^  *r-axSpA*  *week 24* | G1: Prefilled liquid Yisaipu, 25mg, BIW  G2: Lyophilised Yisaipu, 25 mg, BIW | 208  (57.8) | 74  (52.9) | 1.1  (0.9, 1.3) |  | **Low confidence:**  One trial (n≥140 in each arm, high/unclear risk of bias) shows similar risk of adverse events (proportion with at least 1 AE) for lyophilised Yisaipu versus either pre-filled liquid 50 or 25mg Yisaipu.  (Downgraded for study limitations, inconsistency). |
| NCT04345458^16^  *r-axSpA*  *week 24* | G1: Prefilled liquid Yisaipu, 50mg, OW  G2: Lyophilised Yisaipu, 25 mg, BIW | 77  (55) | 74  (52.9) | 1.0  (0.8, 1.3) |  |  |
| **Patient with ≥1 SAE** | | | | | | |
| ChiCTR1900022520^13^  r-axSpA  *week 24* | HS016, 40 mg, Q2W, SC | 18  (4.3) | 6  (2.6) | 1.7  (0.7, 4.2) |  | **Moderate confidence**: biosimilars are likely to have a similar low risk of serious adverse events as ADA in AxSPA  3 RCT, 2 with low risk of bias.  The trials all report a low risk of serious adverse effects for both biosimilars and ADA.  All 3 RCTs include more than 100 pts per arm (larger sample size needed for non-superiority or equivalence (total sample size across all arms: n= 1,466) but are unlikely to be powered for difference in SAEs. |
| CTR20181863^14^  r-axSpA  *week 24* | TQ-Z2301, 40 mg, Q2W, SC | 3  (1.6) | 7  (3.7) | 0.4  (0.1, 1.7) |  |  |
| NCT02893254^15^  *r-axSpA*  *week 24* | IBI303, 40 mg, Q2W, SC | 7  (3) | 8  (4) | 0.9  (0.3, 2.4) |  |  |
| NCT04345458^16^  *r-axSpA*  *week 24* | G1: Prefilled liquid Yisaipu, 25mg, BIW  G2: Lyophilised Yisaipu, 25 mg, BIW | 9  (2.5) | 2  (1.4) | 1.8  (0.4, 8.0) |  | **Very low confidence:**  One trial (n≥140 in each arm, high/unclear risk of bias) shows similar low risk of serious adverse events for lyophilised Yisaipu versus either pre-filled liquid 50 or 25mg Yisaipu.  Study probably not powered for SAEs.  (Downgraded for study limitations |
| NCT04345458^16^  *r-axSpA*  *week 24* | G1: Prefilled liquid Yisaipu, 50mg, OW  G2: Lyophilised Yisaipu, 25 mg, BIW | 4  (2.9) | 2  (1.4) | 2.0  (0.4, 10.7) |  |  |
| **Patient with ≥1 infection** | | | | | | |
| NCT02893254^15^  *r-axSpA*  *week 24* | IBI303, 40 mg, Q2W, SC | 87  (40) | 71  (33) | 1.2  (0.9, 1.6) |  | **Moderate confidence**: biosimilars are likely to have similar risk of infection as ADA in AxSPA  1 RCT, low risk of bias.  This trial reports a slightly higher incidence of infection for biosimilars compared to ADA, but the difference is not significant. (One trial only, downgraded for consistency)  This trial includes more than 100 per arm (total sample size across arms: n= 438). |
| **EMM - Psoriasis** | | | | | | |
| NCT02893254^15^  *r-axSpA*  *week 24* | IBI303, 40 mg, Q2W, SC | 0 | 1  (0.5) | n/a |  | **Low confidence**: biosimilars may have similar risk of incident psoriasis as ADA in AxSPA  1 RCT, low risk of bias.  This trial reports only one case of incident psoriasis in the ADA arm. (One trial only, downgraded for consistency)  This trial includes more than 100 per arm (total sample size across arms: n= 438), but unlikely to be powered for EMMs. (Downgraded for precision) |
| **Tuberculosis** | | | | | | |
| NCT02893254^15^  *r-axSpA*  *week 24* | IBI303, 40 mg, Q2W, SC | 2  (0.9) | 2  (0.9) | 1.0  (0.1, 7.0) |  | **Low confidence**: biosimilars may have similar risk of TB infection as ADA in AxSPA.  1 RCT, low risk of bias.  This trial reports a small rate of TB infection for both biosimilars and ADA. (One trial only, downgraded for consistency)  This trial includes more than 100 per arm (total sample size across arms: n= 438) but is unlikely to be powered for TB infection (downgraded for precision). |
| NCT04345458^16^  *r-axSpA*  *week 24* | G1: Prefilled liquid Yisaipu, 25mg, BIW  G2: Lyophilised Yisaipu, 25 mg, BIW | 1  (0.3) | 0 | n/a |  | **Very low confidence:**  One trial (n≥140 in each arm, high/unclear risk of bias) reports only one case of tuberculosis infection for lyophilised Yisaipu versus pre-filled liquid 25mg Yisaipu. Study probably not powered for detecting differences in TB infection.  (Downgraded for study limitations, inconsistency, precision). |
| **IL-17 inhibitors** | | | | | | |
| **Patient with ≥1 AE** | | | | | | |
| $BE AGILE^17^  *r-axSpA*  *week 12* | **BKZ,** 16 mg, Q4W, SC | 26  (42.6) | 26  (43.3) | 1.0  (0.7, 1.5) |  | **High confidence**  14 RCTs, all but one judged low risk of bias, with high precision – nearly all RCTs more than 50 pts per arm (total sample size across all arms: n= 5,275).  However, there is very low confidence for Netakimab, evaluated in one phase 2 trial, downgraded for risk of bias, inconsistency, and imprecision due to small sample size.  Findings are fairly consistent. In most RCTs a higher proportion of pts with AxSpA receiving IL-17 inhibitors report at least 1 adverse effect compared to placebo. A small number of trials report a higher proportion of pts with at least 1 AEs in the placebo arm (RR below 0), but these findings are not statistically significant.  **Conclusion**:  IL-17 inhibitors appear to be associated with a slightly increased risk of AEs.  RR vary between 1.0 and 1.4 in most trials. Differences are small in most trials and not statistically significant. |
|  | **BKZ,** 64 mg, Q4W, SC | 17  (29.3) | 26  (43.3) | 0.7  (0.4, 1.1) |  |  |
|  | **BKZ,** 160 mg, Q4W, SC | 20  (31.7) | 26  (43.3) | 0.7  (0.5, 1.2) |  |  |
|  | **BKZ,** 320 mg, Q4W, SC | 29  (47.5) | 26  (43.3) | 1.1  (0.7, 1.6) |  |  |
| BE MOBILE 1^18^  nr-axSpA  week 16 | **BKZ,** 160 mg, Q4W, SC | 80  (62.5) | 71  (56.3) | 1.1  (0.9, 1.4) |  |  |
| BE MOBILE 2^18^  *r-axSpA*  *week 16* | **BKZ,** 160 mg, Q4W, SC | 120  (54.3) | 48  (43.2) | 1.3  (1.0, 1.6) |  |  |
| NCT02985983^19^  *mixed*  *week 16* | **BRO**, 210 mg, 3 QW, then Q2W, SC | 44  (55.0) | 45  (57.0) | 1.0  (0.7, 1.3) |  |  |
| COAST -V^2^  *r-axSpA*  *week 16* | **IXE,** 80 mg, Q2W, SC | 36  (43) | 34  (40) | 1.1  (0.8, 1.6) |  |  |
|  | **IXE,** 80 mg, Q4W, SC | 34  (42) | 34  (40) | 1.1  (0.7, 1.5) |  |  |
| COAST -W^20,21^  *r-axSpA*  *week 16* | **IXE,** 80 mg, Q2W, SC | 79  (77) | 60  (57) | 1.4  (1.1, 1.7) |  |  |
|  | **IXE,** 80 mg, Q4W, SC | 63  (66) | 60  (57) | 1.2  (0.9, 1.4) |  |  |
| COAST - X^22,23^  *nr-axSpA*  *week 16* | **IXE,** 80 mg, Q2W, SC | 59  (60.2) | 51  (49) | 1.2  (1.0, 1.6) |  |  |
|  | **IXE,** 80 mg, Q4W, SC | 73  (64) | 51  (49) | 1.3  (1.0, 1.7) |  |  |
| $AILAS^24^  *r-axSpA*  *week 16* | **NTK**, 40 mg**,** 3 QW, Q2W, SC | 11  (50) | 7  (31.8) | 1.6  (0.8, 3.3) |  |  |
|  | **NTK**, 80 mg, 3 QW, Q2W, SC | 6  (27.3) | 7  (31.8) | 0.9  (0.3, 2.1) |  |  |
|  | **NTK**, 120 mg, 3 QW, Q2W, SC | 4  (18.2) | 7  (31.8) | 0.6  (0.2, 1.7) |  |  |
| MEASURE 1^26-30^  *r-axSpA*  *week 16* | **SEC,** 150 mg, 4 QW, then Q4W, SC | 87  (70) | 68  (56) | 1.2  (1.0, 1.5) |  |  |
|  | **SEC,** 75 mg,4 QW, then Q4W, SC | 83  (67) | 68  (56) | 1.3  (1.0, 1.5) |  |  |
| MEASURE 2 ^31-33^  *r-axSpA*  *week 16* | **SEC**, 75 mg, Q4W, SC | 42  (58) | 47  (64) | 0.9  (0.7, 1.2) |  |  |
|  | **SEC,** 150 mg, Q4W, SC | 47  (65) | 47  (64) | 1.0  (0.8, 1.3) |  |  |
| MEASURE 3^34,35^  *r-axSpA*  *week 16* | **SEC,** 150 mg, Q4W, IV | 34  (45.6) | 33  (43) | 1.0  (0.7, 1.5) |  |  |
|  | **SEC**, 300 mg, Q4W, IV | 34  (44.7) | 33  (43) | 1.0  (0.7, 1.5) |  |  |
| MEASURE 4^36^  *r-axSpA*  *week 16* | **SEC**, 150 mg, LD, 4 QW, then Q4W, SC | 72  (62.1) | 64  (54.7) | 1.1  (0.9, 1.4) |  |  |
|  | **SEC,** 150 mg, No LD, 4 QW, then Q4W, SC | 59  (50.4) | 64  (54.7) | 0.9  (0.7, 1.2) |  |  |
| MEASURE 5^37^  *r-axSpA*  *week 16* | **SEC,** 150 mg, Q4W, SC | 206  (67.8) | 91  (59.5) | 1.1  (1.0, 1.3) |  |  |
| PREVENT^38^  nr-axSpA  week 16 | **SEC,** 150 mg, LD, 4 QW, then Q4W, SC | 119  (64.3) | 101  (54.3) | 1.2  (1.0, 1.4) |  |  |
|  | **SEC**,150 mg, NL, 4 QW, then Q4W, SC | 107  (58.2) | 101  (54.3) | 1.1  (0.9, 1.3) |  |  |
| **Patient with ≥1 SAE** | | | | | | |
| $BE AGILE^17^  *r-axSpA*  *week 12* | **BKZ,** 16 mg, Q4W, SC | 0 | 2  (3.3) | 0.2 ^cc^  (0.0, 4.0) |  | **Low confidence**  14 RCTs, all but one judged low risk of bias. Downgraded for (i) precision: nearly all RCTs have more than 50 pts per arm (total sample size across all arms: n=5141), but event rate is very low, and trials not powered to detect differences in SAE; (ii) consistency: proportion with SAEs higher for IL-17 in some trials, higher for placebo in other trials.  There is very low confidence for Netakimab, evaluated in one phase 2 trial, downgraded for risk of bias, inconsistency, and imprecision due to small sample size.  **Conclusion**:  The risk of SAEs is low in all trials.  There is no strong evidence for an increased incidence of SAEs in pts with AxSpA treated with IL-17 inhibitors based on these trials. Sample size of individual trials is too small for reliable estimates of the incidence of SAEs. |
|  | **BKZ,** 64 mg, Q4W, SC | 2  (3.4) | 2  (3.3) | 1.0  (0.2, 7.1) |  |  |
|  | **BKZ,** 160 mg, Q4W, SC | 1  (1.6) | 2  (3.3) | 0.5  (0.0, 5.1) |  |  |
|  | **BKZ,** 320 mg, Q4W, SC | 0 | 2  (3.3) | 0.2 ^cc^  (0.0, 4.0) |  |  |
| BE MOBILE 1^18^  nr-axSpA  week 16 | **BKZ,** 160 mg, Q4W, SC | 0 | 1  (0.8) | 0.3 ^cc^  (0.0, 8.0) |  |  |
| BE MOBILE 2^18^  *r-axSpA*  *week 16* | **BKZ,** 160 mg, Q4W, SC | 3  (1.4) | 0 | 3.5 ^cc^  (0.2, 67.8) |  |  |
| NCT02985983^19^  *mixed*  *week 16* | **BRO**, 210 mg, 3 QW, then Q2W, SC | 4  (5) | 1  (1.3) | 4.0  (0.5, 34.6) |  |  |
| COAST -V^2^  *r-axSpA*  *week 16* | **IXE,** 80 mg, Q2W, SC | 1  (1) | 0 | 3.1 ^cc^  (0.1, 75.2) |  |  |
|  | **IXE,** 80 mg, Q4W, SC | 1  (1) | 0 | 3.2 ^cc^  (0.1, 77.0) |  |  |
| COAST -W^20,21^  *r-axSpA*  *week 16* | **IXE,** 80 mg, Q2W, SC | 1  (1) | 1  (1) | 1.0  (0.1, 16.1) |  |  |
|  | **IXE,** 80 mg, Q4W, SC | 2  (2) | 1  (1) | 2.2  (0.2, 23.5) |  |  |
| COAST - X^22,23^  *nr-axSpA*  *week 16* | **IXE,** 80 mg, Q2W, SC | 3  (3.1) | 5  (4.8) | 0.6  (0.2, 2.6) |  |  |
|  | **IXE,** 80 mg, Q4W, SC | 4  (3.5) | 5  (4.8) | 0.7  (0.2, 2.7) |  |  |
| $AILAS^24^  *r-axSpA*  *week 16* | **NTK**, 40 mg**,** 3 QW, Q2W, SC | 0 | 0 | n/a |  |  |
|  | **NTK**, 80 mg, 3 QW, Q2W, SC | 0 | 0 | n/a |  |  |
|  | **NTK**, 120 mg, 3 QW, Q2W, SC | 0 | 0 | n/a |  |  |
| MEASURE 1^26-30^  *r-axSpA*  *week 16* | **SEC,** 150 mg, Q4W, SC | 3  (2) | 5  (4) | 0.6  (0.1, 2.4) |  |  |
|  | **SEC,** 75 mg, Q4W (starting W 8), SC | 2  (2) | 5  (4) | 0.4  (0.1, 2.0) |  |  |
| MEASURE 2^31-33^  *r-axSpA*  *week 16* | **SEC**, 75 mg, Q4W, SC | 4  (5) | 3  (4) | 1.4  (0.3, 5.8) |  |  |
|  | **SEC,** 150 mg, Q4W, SC | 4  (6) | 3  (4) | 1.4  (0.3, 5.9) |  |  |
| MEASURE 3^34,35^  *r-axSpA*  *week 16* | **SEC,** 150 mg, Q4W, IV | 0 | 1  (1.3) | 0.3 ^cc^  (0.0, 8.2) |  |  |
|  | **SEC**, 300 mg, Q4W, IV | 1  (1.3) | 1  (1.3) | 1.0  (0.1, 15.5) |  |  |
| MEASURE 4^36^  *r-axSpA*  *week 16* | **SEC**, 150 mg, LD, 4 QW, then Q4W, SC | 2  (1.7) | 4  (3.4) | 0.5  (0.1, 2.7) |  |  |
|  | **SEC,** 150 mg, No LD, 4 QW, then Q4W, SC | 2  (1.7) | 4  (3.4) | 0.5  (0.1, 2.7) |  |  |
| MEASURE 5^37^  *r-axSpA*  *week 16* | **SEC,** 150 mg, Q4W, SC | 10  (3.3) | 3  (2) | 1.7  (0.5, 6.0) |  |  |
| PREVENT^38^  nr-axSpA  week 16 | **SEC,** 150 mg, LD, 4 QW, then Q4W, SC | 2  (1) | 5  (2.7) | 0.4  (0.1, 2.1) |  |  |
|  | **SEC**,150 mg, NL, 4 QW, then Q4W, SC | 4  (2) | 5  (2.7) | 0.8  (0.2, 3.0) |  |  |
| **Patient with ≥1 infection** | | | | | | |
| $BE AGILE^17^  *r-axSpA*  *week 12* | **BKZ,** 16 mg, Q4W, SC | 1  (1.6) | 0 | 3.0 ^cc^  (0.1, 71.1) |  | **Moderate confidence**  10 RCTs, all low risk of bias. Downgraded for precision: nearly all RCTs have more than 50 pts per arm (total sample size across all arms: n=4384), but event rate is low, and trials not powered to detect differences in infection rate.  **Conclusion**:  The proportion of pts with at least 1 infection is higher in pts with AxSpA receiving IL-19 inhibitors compared to placebo.  RR ranges from 1.3 to 3.1. Differences between arms are not statistically significant in all trials, but this may be due to a lack of precision: event rates are low in some trials, and trials may not have been powered to detect differences in infection rate. |
|  | **BKZ,** 64 mg, Q4W, SC | 0 | 0 | n/a |  |  |
|  | **BKZ,** 160 mg, Q4W, SC | 0 | 0 | n/a |  |  |
|  | **BKZ,** 320 mg, Q4W, SC | 0 | 0 | n/a |  |  |
| BE MOBILE 1^18^  nr-axSpA  week 16 | **BKZ,** 160 mg, Q4W, SC | 0 | 0 | n/a |  |  |
| BE MOBILE 2^18^  *r-axSpA*  *week 16* | **BKZ,** 160 mg, Q4W, SC | 1  (0.5) | 1  (0.9) | 0.5  (0.0, 8.0) |  |  |
| NCT02985983^19^  *mixed*  *week 16* | **BRO**, 210 mg, 3 QW, then Q2W, SC | 18  (22.5) | 15  (19.0) | 1.2  (0.6, 2.2) |  |  |
| COAST -V^2^  *r-axSpA*  *week 16* | **IXE,** 80 mg, Q2W, SC | 17  (20) | 13  (15) | 1.4  (0.7, 2.6) |  |  |
|  | **IXE,** 80 mg, Q4W, SC | 16  (20) | 13  (15) | 1.3  (0.7, 2.6) |  |  |
| COAST -W ^20,21^  *r-axSpA*  *week 16* | **IXE,** 80 mg, Q2W, SC | 43  (42) | 30  (29) | 1.5  (1.0, 2.1) |  |  |
|  | **IXE,** 80 mg, Q4W, SC | 38  (40) | 30  (29) | 1.4  (0.9, 2.0) |  |  |
| COAST - X^22,23^  *nr-axSpA*  *week 16* | **IXE,** 80 mg, Q2W, SC | 23  (23.5) | 10  (9.6) | 2.4  (1.2, 4.9) |  |  |
|  | **IXE,** 80 mg, Q4W, SC | 34  (29.8) | 10  (9.6) | 3.1  (1.6, 6.0) |  |  |
| $AILAS^24^  *r-axSpA*  *week 16* | **SEC,** 150 mg, Q4W, SC | 43  (34) | 15  (12) | 2.8  (1.6, 4.8) |  |  |
|  | **SEC,** 75 mg, Q4W (starting W 8), SC | 32  (26) | 15  (12) | 2.1  (1.2, 3.7) |  |  |
| MEASURE 2 ^31-33^  *r-axSpA*  *week 16* | **SEC**, 75 mg, Q4W, SC | 22  (30) | 20  (27) | 1.1  (0.7, 1.9) |  |  |
|  | **SEC,** 150 mg, Q4W, SC | 24  (33) | 20  (27) | 1.2  (0.8, 2.0) |  |  |
| MEASURE 4^36^  *r-axSpA*  *week 16* | **SEC**, 150 mg, LD, 4 QW, then Q4W, SC | 1  (0.9) | 0 | 3.0 ^cc^  (0.1, 73.5) |  |  |
|  | **SEC,** 150 mg, No LD, 4 QW, then Q4W, SC | 0 | 0 | n/a |  |  |
| PREVENT^38^  nr-axSpA  week 16 | **SEC,** 150 mg, LD, 4 QW, then Q4W, SC | 1  (0.5) | 0 | 3.0 ^cc^  (0.1, 73.6) |  |  |
|  | **SEC**,150 mg, NL, 4 QW, then Q4W, SC | 1  (0.5) | 0 | 3.0 ^cc^  (0.1, 74.0) |  |  |
| **EMM - Uveitis** | | | | | | |
| BE MOBILE 1^18^  nr-axSpA  week 16 | **BKZ,** 160 mg, Q4W, SC | 2 | 6 | 0.3  (0.1, 1.6) |  | **Low confidence**  8 RCTs, all low risk of bias. Downgraded for (i) precision: nearly all RCTs have more than 50 pts per arm (total sample size across all arms: n= 3353), but event rate is very low, and trials not powered to detect differences in uveitis; (ii) consistency: proportion with uveitis is higher for IL-17 in some trials, higher for placebo in other trials.  **Conclusion**:  The risk of uveitis is low in all trials.  There is no strong evidence for an increased incidence of uveitis in pts with AxSpA treated with IL-17 inhibitors based on these trials, but sample size of individual trials is too small for reliable estimates of the incidence of EMMs. |
| BE MOBILE 2^18^  *r-axSpA*  *week 16* | **BKZ,** 160 mg, Q4W, SC | 0 | 5 | 0.1 ^cc^  (0.0, 0.8) |  |  |
| NCT02985983^19^  *mixed*  *week 16* | **BRO**, 210 mg, 3 QW, then Q2W, SC | 0 | 0 | n/a |  |  |
| COAST -W ^20,21^  *r-axSpA*  *week 16* | **IXE,** 80 mg, Q2W | 3 | 0 | 7.4 ^cc^  (0.4, 142.0) |  |  |
|  | **IXE,** 80 mg, Q4W | 2 | 0 | 4.6 ^cc^  (0.2, 94.0) |  |  |
| COAST - X^22,23^  *nr-axSpA*  *week 16* | **IXE,** 80 mg, Q2W | 2 | 2 | 1.0  (0.2, 7.2) |  |  |
|  | **IXE,** 80 mg, Q4W | 1 | 2 | 0.6  (0.1, 6.0) |  |  |
| MEASURE 4^36^  *r-axSpA*  *week 16* | **SEC**, 150 mg, LD, 4 QW, then Q4W, SC | 0 | 0 | n/a |  |  |
|  | **SEC,** 150 mg, No LD, 4 QW, then Q4W, SC | 0 | 0 | n/a |  |  |
| MEASURE 5 ^37^  *r-axSpA*  *week 16* | **SEC,** 150 mg, Q4W | 3 | 1 | 1.5  (0.2, 14.4) |  |  |
| PREVENT ^38^  nr-axSpA  week 16 | **SEC,** 150 mg, LD, 4 QW, then Q4W, SC | 2 | 1 | 2.0  (0.2, 22.0) |  |  |
|  | **SEC**,150 mg, NL, 4 QW, then Q4W, SC | 0 | 1 | 0.3 ^cc^  (0.0, 8.2) |  |  |
| **EMM - IBD** | | | | | | |
| $BE AGILE^17^  *r-axSpA*  *week 12* | **BKZ,** 16 mg, Q4W, SC | 1  (1.6) | 0 | 3.0 ^cc^  (0.1, 71.1) |  | **Low confidence**  12 RCTs, all low risk of bias. Downgraded for (i) precision: nearly all RCTs have more than 50 pts per arm (total sample size across all arms: n= 4841), but event rate is very low, and trials not powered to detect differences in IBD; (ii) consistency: proportion with IBD higher for IL-17 arm in some trials, higher for placebo in other trials.  **Conclusion**:  The proportion of pts with IBD is very small in all trials.  There is no strong evidence for an increased occurrence of IBD in pts with AxSpA treated with IL-17 inhibitors based on these trials, but sample size of individual trials is too small for reliable estimates of the incidence of EMMs. |
|  | **BKZ,** 64 mg, Q4W, SC | 0 | 0 | n/a |  |  |
|  | **BKZ,** 160 mg, Q4W, SC | 0 | 0 | n/a |  |  |
|  | **BKZ,** 320 mg, Q4W, SC | 0 | 0 | n/a |  |  |
| BE MOBILE 1^18^  nr-axSpA  week 16 | **BKZ,** 160 mg, Q4W, SC | 0 | 1  (0.8) | 0.3  (0.0, 8.0) |  |  |
| BE MOBILE 2^18^  *r-axSpA*  *week 16* | **BKZ,** 160 mg, Q4W, SC | 1  (0.5) | 0 | 1.5 ^cc^  (0.1, 36.9) |  |  |
| NCT02985983^19^  *mixed*  *week 16* | **BRO**, 210 mg, 3 QW, then Q2W, SC | 4 | 2 | 2.0  (0.4, 10.5) |  |  |
| COAST -V^2^  *r-axSpA*  *week 16* | **IXE,** 80 mg, Q2W, SC | 1  (1) | 0 | 3.1 ^cc^  (0.1, 75.2) |  |  |
|  | **IXE,** 80 mg, Q4W, SC | 0 | 0 | n/a |  |  |
| COAST -W^20,21^  *r-axSpA*  *week 16* | **IXE,** 80 mg, Q2W, SC | 0 | 1  (1) | 0.3 ^cc^  (0.0, 8.3) |  |  |
|  | **IXE,** 80 mg, Q4W, SC | 3  (2.6) | 1  (1) | 1.1  (0.1, 17.1) |  |  |
| COAST – X^22,23^  *nr-axSpA*  *week 16* | **IXE,** 80 mg, Q2W, SC | 0 | 1  (1) | 0.4 ^cc^  (0.0, 8.6) |  |  |
|  | **IXE,** 80 mg, Q4W, SC | 1  (1) | 1  (1) | 2.7  (0.3, 25.9) |  |  |
| MEASURE 1^26-30^  *r-axSpA*  *week 16* | **SEC,** 150 mg, Q4W, SC | 0 | 0 | n/a |  |  |
|  | **SEC,** 75 mg, Q4W (starting W 8), SC | 1 | 0 | 3.0 ^cc^  (0.1, 71.8) |  |  |
| MEASURE 2^31-33^  *r-axSpA*  *week 16* | **SEC**, 75 mg, Q4W, SC | 1 | 0 | 3.0 ^cc^  (0.1, 73.4) |  |  |
|  | **SEC,** 150 mg, Q4W, SC | 0 | 0 | n/a |  |  |
| MEASURE 4^36^  *r-axSpA*  *week 16* | **SEC**, 150 mg, LD, 4 QW, then Q4W, SC | 0 | 0 | n/a |  |  |
|  | **SEC,** 150 mg, No LD, 4 QW, then Q4W, SC | 1  (0.9) | 0 | 3.0 ^cc^  (0.1, 73.0) |  |  |
| MEASURE 5^37^  *r-axSpA*  *week 16* | **SEC,** 150 mg, Q4W, SC | 0 | 0 | n/a |  |  |
| PREVENT^38^  nr-axSpA  week 16 | **SEC,** 150 mg, LD, 4 QW, then Q4W, SC | 0` | 0 | n/a |  |  |
|  | **SEC**,150 mg, NL, 4 QW, then Q4W, SC | 1 | 0 | 3.0 ^cc^  (0.1, 74.0) |  |  |
| **Candida** | | | | | | |
| $BE AGILE^17^  *r-axSpA*  *week 12* | **BKZ,** 16 mg, Q4W, SC | 0 | 0 | n/a |  | **Moderate confidence**  12 RCTs, all low risk of bias. Downgraded for (i) precision: nearly all RCTs have more than 50 pts per arm (total sample size across all arms: n= 3,882), but event rate is very low, and trials not powered to detect differences in candida.  **Conclusion**:  In nearly all trials, cases of candida only occur in pts with AxSpA treated with IL-17 inhibitors, but risk of candida is low in most trials. |
|  | **BKZ,** 64 mg, Q4W, SC | 0 | 0 | n/a |  |  |
|  | **BKZ,** 160 mg, Q4W, SC | 0 | 0 | n/a |  |  |
|  | **BKZ,** 320 mg, Q4W, SC | 3  (4.9) | 0 | 6.9 ^cc^  (0.4, 130.5) |  |  |
| BE MOBILE 1^18^  nr-axSpA  week 16 | **BKZ,** 160 mg, Q4W, SC | 5  (3.9) | 0 | 10.8 ^cc^  (0.6, 193.8) |  |  |
| BE MOBILE 2^18^  *r-axSpA*  *week 16* | **BKZ,** 160 mg, Q4W, SC | 10  (4.5) | 0 | 10.6 ^cc^  (0.6, 179.2) |  |  |
| NCT02985983^19^  *mixed*  *week 16* | **BRO**, 210 mg, 3 QW, then Q2W, SC | 0 | 0 | n/a |  |  |
| COAST -V^2^  *r-axSpA*  *week 16* | **IXE,** 80 mg, Q2W, SC | 0 | 0 | n/a |  |  |
|  | **IXE,** 80 mg, Q4W, SC | 0 | 0 | n/a |  |  |
| COAST -W ^20,21^  *r-axSpA*  *week 16* | **IXE,** 80 mg, Q2W, SC | 0 | 1 | 0.3 ^cc^  (0.0, 8.3) |  |  |
|  | **IXE,** 80 mg, Q4W, SC | 0 | 1 | 0.4 ^cc^  (0.0, 8.8) |  |  |
| COAST - X^22,23^  *nr-axSpA*  *week 16* | **IXE,** 80 mg, Q2W, SC | 1 | 0 | 3.2 ^cc^  (0.1, 77.2) |  |  |
|  | **IXE,** 80 mg, Q4W, SC | 0 | 0 | n/a |  |  |
| MEASURE 1^26-30^  *r-axSpA*  *week 16* | **SEC,** 150 mg, 4 QW, then Q4W, SC | 0 | 0 | n/a |  |  |
|  | **SEC,** 75 mg,4 QW, then Q4W, SC | 1 | 0 | 3.0 ^cc^  (0.1, 71.8) |  |  |
| MEASURE 2 ^31-33^  *r-axSpA*  *week 16* | **SEC**, 75 mg, Q4W, SC | 1  (1) | 0 | 3.0 ^cc^  (0.1, 73.4) |  |  |
|  | **SEC,** 150 mg, Q4W, SC | 0 | 0 | n/a |  |  |
| MEASURE 3 ^34,35^  *r-axSpA*  *week 16* | **SEC,** 150 mg, Q4W, IV | 1  (1.3) | 0 | 3.0 ^cc^  (0.1, 73.4) |  |  |
|  | **SEC**, 300 mg, Q4W, IV | 1  (1.4) | 0 | 3.0 ^cc^  (0.1, 71.6) |  |  |
| MEASURE 4^36^  *r-axSpA*  *week 16* | **SEC**, 150 mg, LD, 4 QW, then Q4W, SC | 3  (2.6) | 1  (0.9) | 3.0  (0.3, 28.7) |  |  |
|  | **SEC,** 150 mg, No LD, 4 QW, then Q4W, SC | 0 | 1  (0.9) | 0.3 ^cc^  (0.0, 8.1) |  |  |
| MEASURE 5 ^37^  *r-axSpA*  *week 16* | **SEC,** 150 mg, Q4W, SC | 3 | 0 | 3.5 ^cc^  (0.2, 68.0) |  |  |
| **Herpes zoster** | | | | | | |
| NCT02985983^19^  *mixed*  *week 16* | **BRO**, 210 mg, 3 QW, then Q2W, SC | 1  (1.3) | 0 | 3.0 ^cc^  (0.1, 71.7) |  | **Low confidence**  3 RCTs, low risk of bias reporting very few cases of herpes zoster. Downgraded for precision).  The RCTs included more than 50 pts per arm (n=995 in total) but was not powered to detect differences in infection rates.  **Conclusion:**  There is insufficient evidence for the incidence of herpes zoster in people treated with IL17 inhibitors. |
| COAST -W ^20,21^  *r-axSpA*  *week 16* | **IXE,** 80 mg, Q2W, SC | 0 | 1 | 0.3 ^cc^  (0.0, 8.3) |  |  |
|  | **IXE,** 80 mg, Q4W, SC | 2 | 1 | 2.2  (0.2, 23.5) |  |  |
| COAST – X ^22,23^  *nr-axSpA*  *week 16* | **IXE,** 80 mg, Q2W, SC | 0 | 0 | n/a |  |  |
|  | **IXE,** 80 mg, Q4W, SC | 1 | 0 | 2.7 ^cc^  (0.1, 66.5) |  |  |
| **Tuberculosis** | | | | | | |
| COAST -V^2^  *r-axSpA*  *week 16* | **IXE,** 80 mg, Q2W, SC | 0 | 0 | n/a |  | 2 RCTs (total sample size across all arms, n = 742), both low risk of bias, did not report any new or reactive cases of tuberculosis. (Downgraded for precision).  **Conclusion:**  There is insufficient evidence for the incidence of tuberculosis in people treated with IL17 inhibitors. |
|  | **IXE,** 80 mg, Q4W, SC | 0 | 0 | n/a |  |  |
| COAST -W ^20,21^  *r-axSpA*  *week 16* | **IXE,** 80 mg, Q2W, SC | 0 | 0 | n/a |  |  |
|  | **IXE,** 80 mg, Q4W, SC | 0 | 0 | n/a |  |  |
| **CVD events** | | | | | | |
| $BE AGILE^17^  *r-axSpA*  *week 12* | **BKZ,** 16 mg, Q4W, SC | 0 | 0 | n/a |  | 7 RCTs (total sample size across all arms, n = 3341), all low risk of bias, reporting 3 cases of CVD. (Downgraded for precision).  **Conclusion:**  There is insufficient evidence for the incidence of CVD in people treated with IL17 inhibitors. |
|  | **BKZ,** 64 mg, Q4W, SC | 0 | 0 | n/a |  |  |
|  | **BKZ,** 160 mg, Q4W, SC | 1 | 0 | 2.9 ^cc^  (0.1, 68.9) |  |  |
|  | **BKZ,** 320 mg, Q4W, SC | 0 | 0 | n/a |  |  |
| COAST -W ^20,21^  *r-axSpA*  *week 16* | **IXE,** 80 mg, Q2W, SC | 1 | 0 | 3.1 ^cc^  (0.1, 74.2) |  |  |
|  | **IXE,** 80 mg, Q4W, SC | 0 | 0 | n/a |  |  |
| MEASURE 1^26-30^  *r-axSpA*  *week 16* | **SEC,** 150 mg, 4 QW, then Q4W, SC | 0 | 0 | n/a |  |  |
|  | **SEC,** 75 mg,4 QW, then Q4W, SC | 0 | 0 | n/a |  |  |
| MEASURE 2 ^31-33^  *r-axSpA*  *week 16* | **SEC**, 75 mg, Q4W, SC | 1 | 0 | 3.0 ^cc^  (0.1, 73.4) |  |  |
|  | **SEC,** 150 mg, Q4W, SC | 0 | 0 | n/a |  |  |
| MEASURE 4^36^  *r-axSpA*  *week 16* | **SEC**, 150 mg, LD, 4 QW, then Q4W, SC | 0 | 0 | n/a |  |  |
|  | **SEC,** 150 mg, No LD, 4 QW, then Q4W, SC | 0 | 0 | n/a |  |  |
| MEASURE 5 ^37^  *r-axSpA*  *week 16* | **SEC,** 150 mg, Q4W, SC | 0 | 0 | n/a |  |  |
| PREVENT ^38^  nr-axSpA  week 16 | **SEC,** 150 mg, LD, 4 QW, then Q4W, SC | 0 | 1 | 0.3 ^cc^  (0.0, 8.2) |  |  |
|  | **SEC**,150 mg, NL, 4 QW, then Q4W, SC | 0 | 1 | 0.3 ^cc^  (0.0, 8.2) |  |  |
| **Malignancies** | | | | | | |
| $BE AGILE^17^  *r-axSpA*  *week 12* | **BKZ,** 16 mg, Q4W, SC | 0 | 0 | n/a |  | 7 RCTs (total sample size across all arms, n = 2425), all low risk of bias, did not report any new cases of malignancy. (Downgraded for precision).  **Conclusion:**  There is insufficient evidence |
|  | **BKZ,** 64 mg, Q4W, SC | 0 | 0 | n/a |  |  |
|  | **BKZ,** 160 mg, Q4W, SC | 0 | 0 | n/a |  |  |
|  | **BKZ,** 320 mg, Q4W, SC | 0 | 0 | n/a |  |  |
| NCT02985983^19^  *mixed*  *week 16* | **BRO**, 210 mg, 3 QW, then Q2W, SC | 0 | 0 | n/a |  |  |
| COAST -V^2^  *r-axSpA*  *week 16* | **IXE,** 80 mg, Q2W, SC | 0 | 0 | n/a |  |  |
|  | **IXE,** 80 mg, Q4W, SC | 0 | 0 | n/a |  |  |
| COAST -W ^20,21^  *r-axSpA*  *week 16* | **IXE,** 80 mg, Q2W, SC | 0 | 0 | n/a |  |  |
|  | **IXE,** 80 mg, Q4W, SC | 0 | 0 | n/a |  |  |
| MEASURE 3 ^34, 35^  *r-axSpA*  *week 16* | **SEC,** 150 mg, Q4W, IV | 0 | 0 | n/a |  |  |
|  | **SEC**, 300 mg, Q4W, IV | 0 | 0 | n/a |  |  |
| PREVENT ^38^  nr-axSpA  week 16 | **SEC,** 150 mg, LD, 4 QW, then Q4W, SC | 0 | 0 | n/a |  |  |
|  | **SEC**,150 mg, NL, 4 QW, then Q4W, SC | 0 | 0 | n/a |  |  |
| **IL-6 inhibitors** | | | | | | |
| **Patient with ≥1 AE** | | | | | | |
| $ALIGN^39^  mixed *-axSpA*  *week 12* | Sarilumab**,** 100 mg, Q2W, SC | 34  (69.4) | 18  (36) | 1.9  (1.3, 2.9) |  | **Low confidence**  1 RCT with a total sample size = 501, considered low risk of bias. Patients across all the Sarilumab arms are at higher risk of experiencing at least one adverse event compared to the placebo group (associations are statistically significant). Downgraded for inconsistency (one trial only).  **Conclusion:**  There is limited evidence for an increased incidence of AEs in pts treated with IL-6i. |
|  | Sarilumab**,** 150 mg, Q2W, SC | 40  (78.4) | 18  (36) | 2.2  (1.5, 3.2) |  |  |
|  | Sarilumab**,** 100 mg, QW, SC | 33  (63.5) | 18  (36) | 1.8  (1.2, 2.7) |  |  |
|  | Sarilumab**,** 200 mg, Q2W, SC | 35  (71.4) | 18  (36) | 2.0  (1.3, 3.0) |  |  |
|  | Sarilumab**,** 150 mg, QW, SC | 36  (73.5) | 18  (36) | 2.0  (1.4, 3.1) |  |  |
| **Patient with ≥1 SAE** | | | | | | |
| $ALIGN^39^  mixed *-axSpA*  *week 12* | Sarilumab**,** 100 mg, Q2W, SC | 1  (2) | 0 | 3.1 ^cc^  (0.1, 73.3) |  | **Very low confidence**  **IL-6i:** 1 RCT with a total sample size = 501, considered low risk of bias. The incidence of SAEs was low all Sarilumab arms. Downgraded for inconsistency (one trial only), and imprecision (wide confidence intervals).  **Conclusion:**  There is insufficient evidence regarding the incidence of SAEs in patients treated with IL-6i. |
|  | Sarilumab**,** 150 mg, Q2W, SC | 4  (7.8) | 0 | 8.8 ^cc^  (0.5, 159.8) |  |  |
|  | Sarilumab**,** 100 mg, QW, SC | 1  (1.9) | 0 | 2.9 ^cc^  (0.1, 69.2) |  |  |
|  | Sarilumab**,** 200 mg, Q2W, SC | 0 | 0 | n/a |  |  |
|  | Sarilumab**,** 150 mg, QW, SC | 1  (2) | 0 | 3.1 ^cc^  (0.1, 73.3) |  |  |
| **Patient with ≥1 infection** | | | | | | |
| $ALIGN^39^  mixed *-axSpA*  *week 12* | Sarilumab**,** 100 mg, Q2W, SC | 13  (26.5) | 9  (18) | 1.5  (0.7, 3.1) |  | **Very low confidence**  1 RCT with a total sample size = 501, considered low risk of bias. Risk of experiencing any type of infections is higher in the Sarilumab arms compared to the placebo arm but differences are not statistically significant. Downgraded for inconsistency (one study only), and imprecision (wide confidence intervals).  **Conclusion:**  There is insufficient evidence regarding the risk of infections in patients treated with IL-6i. |
|  | Sarilumab**,** 150 mg, Q2W, SC | 11  (21.6) | 9  (18) | 1.2  (0.5, 2.6) |  |  |
|  | Sarilumab**,** 100 mg, QW, SC | 13  (25) | 9  (18) | 1.4  (0.7, 3.0) |  |  |
|  | Sarilumab**,** 200 mg, Q2W, SC | 9  (18.4) | 9  (18) | 1.0  (0.4, 2.4) |  |  |
|  | Sarilumab**,** 150 mg, QW, SC | 14  (28.5) | 9  (18) | 1.6  (0.8, 3.3) |  |  |
| **EMM - IBD** | | | | | | |
| $ALIGN^39^  mixed *-axSpA*  *week 12* | Sarilumab**,** 100 mg, Q2W, SC | 0 | 0 | n/a |  | **Very ow confidence**  1 RCT with a total sample size = 501, considered low risk of bias. IBD was reported only in one patient in one of the Sarilumab arms. Downgraded for inconsistency and imprecision.  **Conclusion:**  There is insufficient evidence regarding the incidence of IBD in patients treated with IL-6i. |
|  | Sarilumab**,** 150 mg, Q2W, SC | 0 | 0 | n/a |  |  |
|  | Sarilumab**,** 100 mg, QW, SC | 0 | 0 | n/a |  |  |
|  | Sarilumab**,** 200 mg, Q2W, SC | 0 | 0 | n/a |  |  |
|  | Sarilumab**,** 150 mg, QW, SC | 1 | 0 | 3.1 ^cc^  (0.1, 73.3) |  |  |
| **CVD events** | | | | | | |
| $ALIGN^39^  mixed *-axSpA*  *week 12* | Sarilumab**,** 100 mg, Q2W, SC | 0 | 0 | n/a |  | **Very low confidence**  1 RCT with a total sample size = 501, considered low risk of bias. CVD events were reported only in one patient in one of the Sarilumab arms. Downgraded for inconsistency, and imprecision.  **Conclusion:**  There is insufficient evidence regarding the risk of CVD events in patients treated with IL-6i. |
|  | Sarilumab**,** 150 mg, Q2W, SC | 1  (2) | 0 | 2.9 ^cc^  (0.1, 70.6) |  |  |
|  | Sarilumab**,** 100 mg, QW, SC | 0 | 0 | n/a |  |  |
|  | Sarilumab**,** 200 mg, Q2W, SC | 0 | 0 | n/a |  |  |
|  | Sarilumab**,** 150 mg, QW, SC | 0 | 0 | n/a |  |  |
| **IL-23 inhibitors** | | | | | | |
| **Patient with ≥1 AE** | | | | | | |
| $NCT02047110**^40^**  *r-axSpA*  *week 12* | Risankizumab, 18 mg, SD, SC | 28  (70) | 26  (65) | 1.1  (0.8, 1.5) |  | **Very low confidence**  1 RCT with a total sample size = 234, considered low risk of bias. Risk of experiencing at least one adverse event from Risankizumab seems to be similar across all the arms. Downgraded for imprecision (wide confidence interval, <50 pts per arm), and inconsistency (one study only). Differences in the proportion pts experiencing at least 1 AE are not statistically significant).  **Conclusion:**  There is insufficient evidence regarding the incidence of AEs in pts treated with IL-23i. |
|  | Risankizumab, 90 mg**^ν^,** SC | 22  (56.4) | 26  (65) | 0.9  (0.6, 1.2) |  |  |
|  | ^Ə1^ Risankizumab, 180 mg**^ν^**, SC | 26  (65) | 26  (65) | 1.0  (0.7, 1.9) |  |  |
| **Patient with ≥1 SAE** | | | | | | |
| $NCT02047110**^40^**  *r-axSpA*  *week 12* | Risankizumab, 18 mg, SD, SC | 0 | 2  (5) | 0.2 ^cc^  (0.0, 4.0) |  | **Very low confidence**  1 RCT with a total sample size = 234, considered low risk of bias. Very few SAE’s reported across all arms. Downgraded for inconsistency (one RCT only), and imprecision (wide confidence intervals and sample size <50 per arm).  **Conclusion:**  There is insufficient evidence regarding the incidence of SAEs in patients treated with IL-23i. |
|  | Risankizumab, 90 mg**^ν^,** SC | 2  (5.1) | 2  (5) | 1.0  (0.2, 6.9) |  |  |
|  | ^Ə1^ Risankizumab, 180 mg**^ν^**, SC | 1  (2.5) | 2  (5) | 0.5  (0.1, 5.3) |  |  |
| **Patient with ≥1 infection** | | | | | | |
| $NCT02047110**^40^**  *r-axSpA*  *week 12* | Risankizumab, 18 mg, SD, SC | 16  (40) | 13  (32.5) | 0.9  (0.5, 1.6) |  | **Very low confidence**  1 RCT with a total sample size = 234, considered low risk of bias. Risk of experiencing any kind of infections is slightly lower in the Risankizumab arms compared to the placebo arm. Downgraded for imprecision (wide confidence interval) and inconsistency (one trial only).  **Conclusion:**  There is insufficient evidence regarding the risk of infections in patients treated with IL-23i. |
|  | Risankizumab, 90 mg**^ν^,** SC | 11  (28.2) | 13  (32.5) | 0.7  (0.3, 1.4) |  |  |
|  | ^Ə1^ Risankizumab, 180 mg**^ν^**, SC | 10  (25) | 13  (32.5) | 0.6  (0.3, 1.3) |  |  |
| **JAK inhibitors** | | | | | | |
| **Patient with ≥1 AE** | | | | | | |
| TORTUGA^41, 42^  r-axSpA  week 12 | Filgotinib 200mg, OD, oral | 18  (31) | 18  (31) | 1.0  (0.6, 1.7) |  | **High confidence**  5 RCTs, all low risk of bias. All studies >50 pts per arm (total sample size: n= 1,092). Findings are fairly consistent in terms of direction and magnitude. No strong reason to downgrade.  **Conclusion**:  A larger proportion of pts with AxSpA receiving JAKi experience at least 1 adverse event compared to placebo, but the difference is small and not statistically significant.  RR: range 1.0 to 1.25 |
| NCT01786668^43^  *r-axSpA*  *week 12* | Tofacitinib 2 mg, BID; oral | 23  (44) | 22  (43.1) | 1.1  (0.7, 1.7) |  |  |
|  | Tofacitinib 5mg, BID, oral | 28  (53.8) | 22  (43.1) | 1.3  (0.8, 1.9) |  |  |
|  | Tofacitinib 10 mg, BID | 27  (51.9) | 22  (43.1) | 1.2  (0.8, 1.8) |  |  |
| NCT03502616^44,45^  *r-axSpA*  *week 16* | Tofacitinib 5mg, BID, oral | 73  (54.9) | 70  (51.5) | 1.1  (0.9, 1.3) |  |  |
| SELECT-AXIS 1^46,47^  *r-axSpA*  *week 14* | Upadacitinib 15mg OD, oral | 58  (62) | 52  (55) | 1.1  (0.9, 1.4) |  |  |
| SELECT-AXIS 2^48,49^  *nr-axSpA*  *week 14* | Upadacitinib 15mg OD, oral | 75  (48) | 72  (46) | 1.1  (0.8, 1.3) |  |  |
| **Patient with ≥1 SAE** | | | | | | |
| TORTUGA ^41, 42^  r-axSpA  week 12 | Filgotinib 200mg, OD, oral | 1  (2) | 0 | 3.0 ^cc^  (0.1, 72.2) |  | **Moderate confidence**  5 RCTs, all low risk of bias. All studies >50 pts per arm (total sample size: n= 1,092). Findings are less consistent in terms of magnitude and statistical significance, due to very low event rates, leading to low precision (wide confidence intervals). Downgraded for imprecision.  **Conclusion**:  A very low proportion of pts receiving JAKi report serious adverse events.  RR: range 0.1 to 3.0. |
| NCT01786668 ^43^  *r-axSpA*  *week 12* | Tofacitinib 2 mg, BID; oral | 0 | 10  (19.6) | 0.2 ^cc^  (0.0, 4.0) |  |  |
|  | Tofacitinib 5mg, BID, oral | 1  (1.9) | 10  (19.6) | 0.1  (0.0, 0.8) |  |  |
|  | Tofacitinib 10 mg, BID | 1  (1.9) | 10  (19.6) | 0.1  (0.0, 0.8) |  |  |
| NCT03502616 ^44,45^  *r-axSpA*  *week 16* | Tofacitinib 5mg, BID, oral | 2  (1.5) | 1  (0.7) | 2.1  (0.2, 22.3) |  |  |
| SELECT-AXIS 1^46,47^  *r-axSpA*  *week 14* | Upadacitinib 15mg OD, oral | 1  (1) | 1  (1) | 1.0  (0.1, 15.9) |  |  |
| SELECT-AXIS 2 ^48,49^  *nr-axSpA*  *week 14* | Upadacitinib 15mg OD, oral | 4  (3) | 2  (1) | 2.0  (0.4, 10.8) |  |  |
| **Patient with ≥1 infection** | | | | | | |
| TORTUGA^41, 42^  r-axSpA  *week 12* | Filgotinib 200mg, OD, oral | 7  (12) | 7  (12) | 1.0  (0.6, 1.8) |  | **Moderate confidence**  4 RCTs, all low risk of bias. All studies >50 pts per arm (total sample size: n=823). Lower event rate affects precision and likely also inconsistency of findings. Downgraded for imprecision.  **Conclusion**:  A smaller proportion of pts with AxSpA receiving JAKi appear to experience at least 1 infection compared to placebo; estimates vary however, and are not statistically significant.  RR: range 0.7 to 1.0 |
| NCT01786668^43^  *r-axSpA*  *week 12* | Tofacitinib 2 mg, BID; oral | 12  (23.1) | 12  (23.5) | 1.0  (0.5, 2.0) |  |  |
|  | Tofacitinib 5mg, BID, oral | 11  (21.2) | 12  (23.5) | 0.9  (0.4, 1.9) |  |  |
|  | Tofacitinib 10 mg, BID | 9  (17.3) | 12  (23.5) | 0.7  (0.3, 1.6) |  |  |
| SELECT-AXIS 1^46,47^  *r-axSpA*  *week 14* | Upadacitinib 15mg OD, oral | 19  (20) | 26  (28) | 0.7  (0.4, 1.2) |  |  |
| SELECT-AXIS 2 ^48,49^  *nr-axSpA*  *week 14* | Upadacitinib 15mg OD, oral | 36  (23) | 36  (23) | 1.0  (0.7, 1.5) |  |  |
| **EMM - Uveitis** | | | | | | |
| TORTUGA^41, 42^  r-axSpA  *week 12* | Filgotinib 200mg, OD, oral | 0 | 0 | n/a |  | **Low confidence**  Two RCTs (low RoB), very low event rate leading to low precision.  **Conclusion**: risk of uveitis was low in this sample of pts with Ax-SpA |
| SELECT-AXIS 2 ^48,49^  *nr-axSpA*  *week 14* | Upadacitinib 15mg OD, oral | 1  (1) | 0 | n/a |  |  |
| **EMM - IBD** | | | | | | |
| TORTUGA^41, 42^  r-axSpA  *week 12* | Filgotinib 200mg, OD, oral | 0 | 0 | n/a |  | **Low confidence**  Two RCTs (low RoB), very low event rate leading to low precision.  **Conclusion**: risk of IBD is very low in this sample of pts with Ax-SpA |
| SELECT-AXIS 2 ^48,49^  *nr-axSpA*  *week 14* | Upadacitinib 15mg OD, oral | 0 | 0 | n/a |  |  |
| **EMM - Psoriasis** | | | | | | |
| TORTUGA^41, 42^  r-axSpA  *week 12* | Filgotinib 200mg, OD, oral | 0 | 0 | n/a |  | **Low confidence**  Only one RCT (low RoB), very low event rate leading to low precision.  **Conclusion**: risk of psoriasis is very low in this sample of pts with Ax-SpA |
| NCT03502616 ^44,45^  *r-axSpA*  *week 16* | Tofacitinib 5mg, BID, oral | 0 | 0 | n/a |  |  |
| SELECT-AXIS 2 ^48,49^  *nr-axSpA*  *week 14* | Upadacitinib 15mg OD, oral | 0 | 0 | n/a |  |  |
| **Candida** | | | | | | |
| NCT01786668 ^43^  *r-axSpA*  *week 12* | Tofacitinib 2 mg, BID; oral | 0 | 0 | n/a |  | **Moderate confidence**  4RCTs (n=976), very low event rate leading to low precision.  **Conclusion**: risk of herpes zoster is very low in this sample of pts with Ax-SpA. |
|  | Tofacitinib 5mg, BID, oral | 0 | 0 | n/a |  |  |
|  | Tofacitinib 10 mg, BID | 1  (1.9) | 0 | n/a |  |  |
| NCT03502616^44,45^  *r-axSpA*  *week 16* | Tofacitinib 5mg, BID, oral | 0 | 0 | n/a |  |  |
| SELECT-AXIS 1^46,47^  *r-axSpA*  *week 14* | Upadacitinib 15mg OD, oral | 0 | 0 | n/a |  |  |
| SELECT-AXIS 2^48,49^  *nr-axSpA*  *week 14* | Upadacitinib 15mg OD, oral | 2  (1) | 1  (1) | 2.0  (0.2, 22.0) |  |  |
| **Tuberculosis** | | | | | | |
| TORTUGA^41, 42^  r-axSpA  *week 12* | Filgotinib 200mg, OD, oral | 0 | 0 | n/a |  | **Moderate confidence**  3 RCTs (n=416), very low event rate leading to low precision.  **Conclusion**: risk of tuberculosis is very low in this sample of pts with Ax-SpA. |
| SELECT-AXIS 1^46,47^  *r-axSpA*  *week 14* | Upadacitinib 15mg OD, oral | 0 | 0 | n/a |  |  |
| SELECT-AXIS 2 ^48,49^  *nr-axSpA*  *week 14* | Upadacitinib 15mg OD, oral | 0 | 0 | n/a |  |  |
| **Thromboembolic events** | | | | | | |
| TORTUGA^41, 42^  r-axSpA  *week 12* | Filgotinib 200mg, OD, oral | 1  (2) | 0 | n/a |  | **Moderate confidence**  4 RCTs (n=885), very low event rate leading to low precision.  **Conclusion**: risk of thromboembolic events is very low in this sample of pts with Ax-SpA. |
| NCT03502616^44,45^  *r-axSpA*  *week 16* | Tofacitinib 5mg, BID, oral | 0 | 0 | n/a |  |  |
| SELECT-AXIS 1^46,47^  *r-axSpA*  *week 14* | Upadacitinib 15mg OD, oral | 0 | 0 | n/a |  |  |
| SELECT-AXIS 2^48,49^  *nr-axSpA*  *week 14* | Upadacitinib 15mg OD, oral | 0 | 0 | n/a |  |  |
| **GI - Diarrhoea** | | | | | | |
| TORTUGA^41, 42^  r-axSpA  *week 12* | Filgotinib 200mg, BID, oral |  |  |  |  | **Moderate confidence**  4 RCTs (n=976), low event rate leading to low precision.  **Conclusion**: risk of diarrhoea is low in this sample of pts with Ax-SpA. Differences between JAKi and placebo are small. |
| NCT03502616^44,45^  *r-axSpA*  *week 16* | Tofacitinib 5mg, BID, oral | 6  (4.5) | 5  (3.7) | n/a |  |  |
| SELECT-AXIS 1^46,47^  *r-axSpA*  *week 14* | Upadacitinib 15mg OD, oral |  |  |  |  |  |
| SELECT-AXIS 2^48,49^  *nr-axSpA*  *week 14* | Upadacitinib 15mg OD, oral |  |  |  |  |  |
| **CVD events** | | | | | | |
| NCT03502616^44,45^  *r-axSpA*  *week 16* | Tofacitinib 5mg, BID, oral | 0 | 0 | n/a |  | **Moderate confidence**  3 RCTs (n=769), very low event rate leading to low precision.  **Conclusion**: risk of CVD events is very low in this sample of pts with Ax-SpA. |
| SELECT-AXIS 1^46,47^  *r-axSpA*  *week 14* | Upadacitinib 15mg OD, oral | 0 | 0 | n/a |  |  |
| SELECT-AXIS 2 ^48,49^  *nr-axSpA*  *week 14* | Upadacitinib 15mg OD, oral | 0 | 0 | n/a |  |  |
| **PDE4 inhibitors** | | | | | | |
| **Patient with ≥1 AE** | | | | | | |
| $POSTURE**^50^**  *r-axSpA*  *week 24* | Apremilast, 20 mg, BID**,** Oral | 89  (55) | 82  (50) | 1.1  (0.9,1.3) |  | **Low confidence**  1 RCT with a total sample size = 654, with some concerns of risk of bias. Risk of experiencing at least one adverse event from Apremilast seems to be similar across all the arms. Downgraded for risk of bias and imprecision (wide confidence intervals). Differences in the proportion of pts experiencing at least 1 AE are not statistically significant).  **Conclusion:**  There is insufficient evidence regarding the incidence of AEs in pts treated with PDE4i. |
|  | **^Ə2^** Apremilast, 30 mg, BID, Oral | 85  (52) | 82  (50) | 1.0  (0.8,1.3) |  |  |
| **Patient with ≥1 SAE** | | | | | | |
| $POSTURE**^50^**  *r-axSpA*  *week 24* | Apremilast, 20 mg, BID**,** Oral | 3  (2) | 1  (1) | 3.0  (0.3, 28.7) |  | **Very low confidence**  1 RCT with a total sample size = 654, with some concerns of risk of bias. Risk of experiencing SAE’s is higher in the intervention arms compared to the placebo arm. Downgraded for risk of bias, inconsistency (one study only), and imprecision (wide confidence intervals).  **Conclusion:**  There is insufficient evidence regarding the incidence of SAEs in patients treated with PDE4i. |
|  | **^Ə2^** Apremilast, 30 mg, BID, Oral | 6  (4) | 1  (1) | 6.0  (0.7, 49.6) |  |  |
| **EMM - Uveitis** | | | | | | |
| $POSTURE**^50^**  *r-axSpA*  *week 24* | Apremilast, 20 mg, BID**,** Oral | 1 | 0 | n/a |  | **Very low evidence**  1 RCT with a total sample size = 654, with some concerns of risk of bias. Uveitis was reported only in one patient in one of the Apremilast arms. Downgraded for risk of bias, inconsistency, and imprecision.  **Conclusion:**  There is insufficient evidence regarding the incidence of new onset Uveitis in patients treated with PDE4i. |
|  | **^Ə2^** Apremilast, 30 mg, BID, Oral | 0 | 0 | n/a |  |  |
| **Malignancies** | | | | | | |
| $POSTURE**^50^**  *r-axSpA*  *week 24* | Apremilast, 20 mg, BID**,** Oral | 0 | 1 | n/a |  | **Very low Confidence**  1 RCT with a total sample size = 654, with some concerns of risk of bias. Only one patient in the placebo arm was recorded with a malignancy. Downgraded for risk of bias, inconsistency, and imprecision.  **Conclusion:**  There is insufficient evidence regarding the risk of malignancies in patients treated with PDE4i. |
|  | **^Ə2^** Apremilast, 30 mg, BID, Oral | 0 | 1 | n/a |  |  |

a*: TNFi – naïve group; b*: TNFi – Inadequate response group. Studies are grouped into a and b only if the outcome is reported by TNFi status.

RR: Relative risk; CI: Confidence interval; ^cc^ Continuity correction: in case of zero events in one of the arms, ‘metan’ command in Stata automatically inputs 0.5 to each cell in order to calculate RR; **^¥^**Primary outcome*;* $phase 2 trial; a*: TNFi – naïve group; b*: TNFi – Inadequate response group. Studies are grouped into a and b only if the outcome is reported by TNFi status; r: radiographic axSpA; nr: non-radiographic axSpA; LD: loading dose; NL: No loading dose; AE: Adverse events; SAE: Serious adverse events; EMM: Extra musculoskeletal manifestations; IBD: Inflammatory bowel disease; BID – twice a day; BIW – twice weekly, OW – Once weekly; Q2W - Once every two weeks; Q4W - Once every four weeks; OD: once daily; SD: single dose; **^ν^**day 1, and at wks - 8, 16 and 24; SC: Subcutaneous; IV: Intravenous; Risk of bias: Green – Low, Amber – Unclear, Red – High.

Interventional drugs: ADA - Adalimumab; BKZ - Bimekizumab; BRO- Brodalumab; CZP- Certolizumab; ETN- Etanercept; GOL- Golimumab; IXE- Ixekizumab; NTK- Netakimab; RIS- Risankizumab; SAR- Sarilumab; Sec- Secukinumab.

## Guideline question 4 (Q4)

In adults with active axSpA, what is the evidence for a treat-to-target strategy compared to usual care?

### Table 5: Study characteristics of included studies (Q4)

| Study ID (axSpA type); *Primary outcome (time point in weeks)*  Randomised groups | Induction therapy  Duration  N_i_ | | Eligibility criteria  N_r_ | N | Age, years Mean (SD) / *median (IQR) | Male  n (%) | HLA-B27-positive n (%) | Disease duration,  years  Mean /*median (SD/**IQR) | Symptom duration, years  Mean (SD) / *median (IQR) | Previous TNFi, n (%) | EMM (Active / History^/ Mixed^^)  n (%) | Overall Risk of bias |
| --- | --- | --- | --- | --- | --- | --- | --- | --- | --- | --- | --- | --- |
|  |  |  |  |  |  |  |  |  |  |  | 1. Anterior Uveitis 2. IBD 3. Psoriasis |  |
| Treat-to-target (T2T) | | | | | | | | | | | | |
| NL6771^51^ (mixed axSpA); *ASDAS -LDA (week 52)* | | | | | | | | | | | | |
| T2T with tapering | | n/a | ASDAS<2.1 for at least 6 months | 39 | 46  (13) | 25  (64) | 34  (87) | *13  (**5-24) | NR | 11  (26) | 1. NR 2. 4^ (10) 3. 4^ (10) |  |
| T2T without tapering | |  |  | 19 | 47  (17) | 12  (63) | 18  (95) | *12  (**2-26) | NR | 7  (37) | 1. NR 2. 1^ (5) 3. 0^ |  |
| NOR-DRUM^52^ (r- axSpA); ASDAS: Inactive disease (<1.3) *(week 30)* | | | | | | | | | | | | |
| Therapeutic drug monitoring (TDM)^±^ | Maintenance therapy with INX  30 weeks and 3 years | | ASDAS <1.3 | 59 | 44.8  (13.9) | 30  (51) | 41^Ͷt^  (77) | 3.3  (**0.5-14.7) | NR | 18  (30) | NR |  |
| Standard, INX: bs-CT-P13, 5mg/kg, IV |  |  |  | 58 | 42.6  (14.1) | 37  (64) | 42 ^Ͷc^  (76) | 3.1  (**0.4-14.2) | NR | 21  (37) | NR |  |
| ^±^dose adjusted according to an algorithm designed to maintain infliximab levels within the therapeutic range | | | | | | | | | | | | |
| TIPCOSA^53^ (mixed axSpA); *ASAS30 (week 48)* | | | | | | | | | | | | |
| Tight control (TC) / T2T (target – ASDAS -LDA) | n/a | | ASDAS ≥2.1 | 80 | 37.6  (10.8) | 45  (56.2) | 62 (77.5) | 4.2  (6.6) | NR | n/a | 1. 16^ (20) 2. NR 3. NR |  |
| Usual care |  |  |  | 80 | 38.1  (11.4) | 37  (46.2) | 58 (72.5) | 3.3  (5.8) | NR | n/a | 1. 6^ (7.5) 2. NR 3. NR |  |

EMM: Extra musculoskeletal manifestations; ^ EMM – history of events; ^^ EMM includes both active and history events; IBD: Inflammatory bowel disease; NR: Not reported; n/a: Not applicable; INX: Infliximab; bs: Bio similar; Risk of bias: Green – Low, Amber – Unclear.

### Table 6: Efficacy outcomes - Binary data (Q4)

| **Study ID**  *Population*  *Time point* | **Treatment arm** | **Response, n (%)** | | **RR**  **(95% CI)** | **NNT** | **Risk of bias** | **Quality of evidence**  **Given the small evidence base confidence has been downgraded for all outcomes (confidence cannot be higher than moderate)** |
| --- | --- | --- | --- | --- | --- | --- | --- |
|  |  | **Intervention** | **Control** |  |  |  |  |
| **ASAS20** | | | | | | | |
| TIPCOSA**^53^**  *Mixed axSpA*  *week 48* | TC/T2T | 76  (94.9) | 69  (85.9) | 1.1  (1.0, 1.2) | 11.1 |  | **Low evidence**: Proportion of participants meeting the ASAS 20 criteria is higher when the disease activity is closely monitored based on pre-specified target compared to usual care.  1 RCT with a sample size of 160, judged as low risk of bias contributed to the evidence. Downgraded for indirectness and imprecision. |
| **ASAS40** | | | | | | | |
| TIPCOSA**^53^**  *Mixed axSpA*  *week 48* | TC/T2T | 42  (52.3) | 28  (34.7) | 1.5  (1.0, 2.2) | 5.7 |  | **Low evidence**: Proportion of participants meeting the ASAS 20 criteria is higher when the disease activity is closely monitored based on pre-specified target compared to usual care.  1 RCT with a sample size of 160, judged as low risk of bias contributed to the evidence. Downgraded for indirectness and imprecision. |
| **ASDAS – Clinically Important Improvement (CII): Δ ≥ - 1.1** | | | | | | | |
| TIPCOSA**^53^**  *Mixed axSpA*  *week 48* | TC/T2T | 49  (61.2) | 37  (46) | 1.3  (1.0,1.8) | 6.6 |  | **Low confidence**: There is insufficient evidence to conclude that tight control of the disease activity leads to clinically important improvement in the ASDAS SCORE. Despite 1 RCT with a sample size of 160 participants and judged as low risk of bias indicating greater proportion of participants in the TC group with clinically important improvement in the ASDAS score, there is uncertainty in the evidence. Downgraded for inconsistency, indirectness, and imprecision. |
| **ASDAS – Major Improvement (MI): Δ ≥ -2.0** | | | | | | | |
| TIPCOSA**^53^**  *Mixed axSpA*  *week 48* | TC/T2T | 13  (16.5) | 12  (14.9) | 1.1  (0.5, 2.2) | 62.5 |  | **Low confidence**: There is insufficient evidence to conclude that tight control of the disease activity leads to major improvement in the ASDAS SCORE. 1 RCT with a sample size of 160 participants and judged as low risk of bias did not indicate any clear difference in the major improvement rates between the 2 groups. Downgraded for inconsistency, indirectness, and imprecision. |
| **ASDAS: Inactive disease (<1.3)** | | | | | | | |
| ^¥^NOR-DRUM**^52^**  *r-axSpA*  *week 30* | TDM - INX | 23  (39) | 21  (36.2) | 1.1  (0.7, 1.7) | 36 |  | **Low confidence:** There is insufficient evidence to conclude with certainty the effect of therapeutic monitoring of the disease activity on the proportion of participants achieving inactive disease status.  2 RCTs judged as low risk of bias with a total sample size of 277 contributed to the evidence. (Downgraded for inconsistency and imprecision). |
| TIPCOSA**^53^**  *Mixed axSpA*  *week 48* | TC/T2T | 21  (25.9) | 15  (18.7) | 1.4  (0.8, 2.5) | 13.9 |  |  |
| **ASDAS: Low disease activity (<2.1)** | | | | | | | |
| NL-6771**^51^**  *Mixed axSpA*  *week 52* | Tapering | 26  (67) | 14  (74) | 0.9  (0.6, 1.3) | -14.3 |  | **Low confidence:** There is insufficient evidence to conclude with certainty the effect of tapering or the continuation of the standard maintenance dose on the proportion of participants achieving low disease activity status.  2 RCTs judged as low risk of bias with a total sample size of 218 contributed to the evidence. (Downgraded for inconsistency, and imprecision). |
| TIPCOSA**^53^**  *Mixed axSpA*  *week 48* | TC/T2T | 61  (76.5) | 48  (59.5) | 1.3  (1.0, 1.6) | 5.9 |  |  |
| **BASDAI50** | | | | | | | |
| TIPCOSA**^53^**  *Mixed axSpA*  *week 48* | TC/T2T | 63  (79) | 35  (43.8) | 1.8  (1.4, 2.4) | 2.8 |  | **Low confidence:** Monitoring of axSpA patients using a tight control regimen may be more likely to experience 50% improvement in the BASDAI score compared to those receiving usual care.  1 RCT with a sample size of 160 participants and judged as low risk of bias contributed to the evidence. Downgraded for indirectness, and imprecision. |

RR: Relative risk; CI: confidence interval; NNT: Number needed to treat; **^¥^**Primary outcome; INX: Infliximab; TC: Tight control; T2T: treat-to-target; TDM: Therapeutic drug monitoring; Risk of bias: Green – Low, Amber – Unclear.

### Table 7: Efficacy outcomes - Continuous data (Q4)

| **Study ID**  *Population*  *Time point* | **Treatment arm (n)** | **Mean CfB**  **(SD/SE^)**  **Median (IQR)^^** | **Standardised Mean Difference (CfB)**  **(95% CI)** | **Risk of bias** | **Quality of evidence** |
| --- | --- | --- | --- | --- | --- |
| **ASDAS – CRP mean score (change from baseline – CfB)** | | | | | |
| NL-6771**^51^**  *Mixed axSpA*  *week 52* | T2T without tapering (19) | 0.3  (0.7) | **Ref** |  | **Low confidence**: Monitoring of axSpA patients using a tight control regimen may have a positive effect on the disease activity. However, tapering may not result to desirable effect.  3 RCTs with a sample size of 335 participants and judged as low risk of bias contributed to the evidence.  Downgraded for inconsistency, and imprecision. |
|  | T2T with tapering (39) | 0.5  (0.9) | 0.2  (-0.4, 0.8) |  |  |
| NOR-DRUM**^52^**  *r-axSpA*  *week 30* | Standard dose – INX (58) | -1.3  (1.1) | **Ref** |  |  |
|  | TDM – INX (59) | -1.4  (1.4) | -0.1  (-0.4, 0.3) |  |  |
| TIPCOSA**^53^**  *Mixed axSpA*  *week 48* | Usual Care (80) | -0.8  (0.8) | **Ref** |  |  |
|  | TC/T2T (80) | -1.1  (0.8) | -0.5  (-0.8, -0.1) |  |  |
| **BASDAI mean score (change from baseline)** | | | | | |
| NOR-DRUM**^52^**  *r-axSpA*  *week 30* | Standard dose – INX (58) | -2.2  (2.0) | **Ref** |  | **Low confidence:** Tight control using a targeted approach may have a positive effect on disease activity.  2 RCTs, judged as low risk of bias, with a total sample size of 277 contributed to the evidence.  (Downgraded for inconsistency, and imprecision).  277 |
|  | TDM – INX (59) | -2.0  (2.3) | 0.1  (-0.3, 0.5) |  |  |
| TIPCOSA **^53^**  *Mixed axSpA*  *week 48* | Usual Care (80) | -1.5  (2.0) | **Ref** |  |  |
|  | TC/T2T (80) | -2.5  (1.7) | -0.6  (-0.9, -0.3) |  |  |

CI: confidence interval; INX: Infliximab; TC: Tight control; T2T: treat-to-target; TDM: Therapeutic drug monitoring; ^c^ : Risk of bias: Green – Low, Amber – Unclear.

### Table 8: Safety outcomes (Q4)

| **Study** | **Drug** | **Response, n (%)** | | **RR (95% CI)** | **Risk of bias** | **Quality of evidence** |
| --- | --- | --- | --- | --- | --- | --- |
|  |  | **Intervention** | **Control** |  |  |  |
| **Patient with ≥1 AE** | | | | | | |
| TIPCOSA**^53^**  *Mixed axSpA*  *week 48* | TC/T2T | 33  (41.3) | 22  (27.5) | 1.5  (1.0, 2.3) |  | **Low confidence**: There is insufficient evidence that patients monitored using tight control regimen may be at high risk of experiencing an adverse event compared to those receiving standard dose.  1 RCT judged as low risk of bias contributed to the evidence.  Downgraded for indirectness and imprecision. |
| **Patient with ≥1 infection** | | | | | | |
| TIPCOSA**^53^**  *Mixed axSpA*  *week 48* | TC/T2T | 15  (18.8) | 16  (20) | 0.9  (0.5, 1.8) |  | **Low confidence:** There is insufficient evidence that tight control regimen may increase the incidence of infections compared to the standard dose group.  1 RCT, judged as low risk of bias, with a sample size of 160 contributed to the evidence.  Downgraded for indirectness and imprecision. |
| **CVD events** | | | | | | |
| TIPCOSA**^53^**  *Mixed axSpA*  *week 48* | TC/T2T | 0 | 0 | n/a |  |  |

RR: Relative risk; CI: confidence interval; AE: Adverse events; TC: Tight control; T2T: treat-to-target; Risk of bias: Amber – Unclear.

## Guideline question 5 (Q5)

In adults with axSpA who have achieved clinical remission or low disease activity, what is the evidence, compared to usual care, for tapering or dose reduction of targeted therapies; withdrawing targeted therapies; and switching to biosimilars?

### Table 9: Study characteristics of included studies (Q5)

| Study ID (axSpA type); *Primary outcome (time point in weeks)*  Randomised groups | Induction therapy  Duration  N_i_ | | Eligibility criteria  N_r_ | N | Age, years Mean (SD) / *median (IQR) | Male  n (%) | HLA-B27-positive n (%) | Disease duration,  years  Mean /*median (SD/**IQR) | Symptom duration, years  Mean (SD) / *median (IQR) | Previous TNFi, n (%) | EMM (Active / History^/ Mixed^^)  n (%) | | Overall Risk of bias |
| --- | --- | --- | --- | --- | --- | --- | --- | --- | --- | --- | --- | --- | --- |
|  |  |  |  |  |  |  |  |  |  |  | i.Anterior Uveitis  ii.IBD  iii.Psoriasis | |  |
| Tapering of targeted therapies | | | | | | | | | | | | | |
| $ANSWERS^54^ (r-axSpA); *BASDAI50 / ↓ ≥ 2-unit in BASDAI / ↓ ≥ 2-unit in BASDAI spinal pain (week 24)* | | | | | | | | | | | | | |
| ETN, 25 mg, OW | ETN, 50 mg, OW  6 months  N_i_ - 59 | | Responders: BASDAI50 or fall ≥2 units and a ≥2-unit reduction in BASDAI spinal pain | 23 | 46.7  (14.1) | 41  (87.2) | NR | NR | NR | n/a | 1. n/a 2. n/a   iii.NR |  | |
| ETN, 50 mg, OW |  |  |  | 24 |  |  | NR | NR | NR | n/a | 1. n/a 2. n/a 3. NR |  |  |
| C-OPTIMISE^55^ (mixed- axSpA); *Flare^♨^-free (week 48)* | | | | | | | | | | | | | |
| CZP, 200 mg, Q4W, SC | CZP, 200 mg, Q2W, SC  48 weeks  N_i_ - 736 | | Sustained remission: ASDAS <1.3  N_r_ - 323 | 105 | 32.4  (6.9) | 83  (79.0) | 97 (92.4) | 2.0  (1.7) | 3.4  (1.8) | 6  (5.7) | 1. 20(19.0) 2. 3 (2.9) 3. 2 (1.9) |  | |
| CZP, 200 mg, Q2W, SC |  |  |  | 104 | 32.6  (7.2) | 79  (76.0) | 91 (87.5) | 2.5  (1.7) | 3.8  (2.8) | 4  (3.8) | 1. 16 (15.4) 2. 2 (1.9) 3. 8 (7.7) |  |  |
| COAST-Y^56^ (mixed- axSpA); *Flare^♨^-free (week 52)* | | | | | | | | | | | | | |
| IXE, 80 mg, Q4W | IXE 80 mg, Q2W (N_i_ -350) /Q4W (Ni - 423)  24 weeks | | ASDAS <1.3 at weeks 16 or 20 and<2.1 at both visits.  N_r_ - 155 | 48 | 36.5  (9.7) | 38  (79) | 43  (90) | 7.1  (7.0) | 12.6  (7.5) | 9  (19) | NR |  | |
| IXE, 80 mg, Q2W |  |  |  | 54 | 38.4  (10.8) | 40  (74) | 49  (91) | 7.6  (8.4) | 12.9  (8.6) | 8  (15) | NR |  |  |
| GO-BACK^57^ (nr-axSpA); *ASDAS -LDA (week 52)* | | | | | | | | | | | | | |
| GLM, 50 mg, Q2M, SC | GLM 50mg, SC, QMT  10 months  N_i_ - 323 | | ASDAS <1.3 at both months 7 and 10 | 63 | *31.0  (20–44) | 42  (66.7) | 47 (74.6) | *0.51  (0-4.7) | *1.7  (0.2–8.2) | NR | NR |  | |
| GLM, 50 mg, QM, SC |  |  |  | 63 | *31.0  (18–45) | 44  (69.8) | 47 (74.6) | *0.82  (0-5) | *2.1  (0.1–8.5) | NR | NR |  |  |
| NTR3903^58^ (r-axSpA); *ASDAS -LDA (week 24)* | | | | | | | | | | | | | |
| ETN, 50 mg, Q2W/25mg OW, SC | ETN, 50 mg, Q2W/25mg OW, SC  6 months | | ASDAS<2.1 | 20 | 47  (12) | 16  (80) | 17  (85) | *15  (**10–28) | NR | NR | 1. 8^ (40) 2. NR 3. NR |  | |
| ETN, 50mg OW/ 25mg BIW, SC |  |  |  | 20 | 49  (10) | 17  (85) | 16  (80) | *17  (**7–22) | NR | NR | 1. 9^ (45) 2. NR 3. NR |  |  |
| REDES-TNF^59^ (mixed); *ASDAS -LDA (week 52)* | | | | | | | | | | | | | |
| 1. ADA, 40 mg Q3W, SC 2. ETN, 50mg every 10 days, SC 3. GLM, 50 mg, Q6W, SC 4. INF, 3mg/kg Q8W, IV | Current therapy – standard doses of one of the TNFi – INX, ADA, ETN, or GLM.  N_i_ - 157 | | Sustained clinical remission: (BASDAI) ≤ 2, no clinically active arthritis or enthesitis and C-reactive protein equal to or higher than the upper limit of  normality for ≥ 6 months. | 60   1. 23 2. 20 3. 5 4. 12 | 43.7  (12.4) | 49  (81.7) | NR | *9.3 (**5.2-17.6) | NR | 16  (26.6) | NR |  | |
| 1. ADA, 40 mg Q2W, SC 2. ETN, 25mg – BIW/50mg -QW, SC 3. GLM, 50 mg, Q6W, SC 4. INF, 5mg/kg, Q6W/ Q8W, IV |  |  |  | 60   1. 24 2. 21 3. 4 4. 11 | 46.2  (13.7) | 53  (88.3) | NR | *10.4  (**7.1, 20.8) | NR | 10  (16.7) | NR |  |  |
| T2TEASb^60^ (r- axSpA); Flare rate *(week 36)* | | | | | | | | | | | | | |
| Stepwise  25mg, OW- 12wks, 25mg, Q2W- 12wks, 25mg, OM- 12wks | rhTNFR:Fc (ETN – bs) 50 mg, SC, OW  12 weeks  N_i_ - 311 | | Low disease activity: ASDAS ≥ 1.3 < 2.1  N_r_ - 93 | 53 | 33.5  (12.2) | 42  (79.3) | NR | *5  (**2.3, 7.3) | NR | NR | NR |  | |
| Delayed  50 mg, OW- 12wks, 25 mg, OW- 12wks, 25mg, Q2W - 12wks |  |  |  | 19 | 33.1  (8.9) | 10  (52.6) | NR | *6  (**4.5, 10.9) | NR | NR | NR |  |  |
| Withdrawing of targeted therapies | | | | | | | | | | | | | |
| ABILITY-3^61^ (nr-axSpA); *Flare-^♨♨^* *free (week 40)* | | | | | | | | | | | | | |
| ADA, 40 mg, Q2W, SC | | ADA, 40 mg, Q2W, SC  N_i_ - 673 | ASDAS inactive disease: <1.3 | 152 | 34·7  (10·3) | 96  (63) | 132  (87) | 1·9  (2·9) | 6·4  (6·9) | NR | NR |  | |
| Placebo | |  |  | 153 | 35·3  (10·2) | 93  (61) | 134  (88) | 1·8  (2·9) | 7·1  (6·8) | NR | NR |  |  |
| C-OPTIMISE^55^ (mixed- axSpA); *Flare^♨^-free (week 48)* | | | | | | | | | | | | | |
| CZP, 200 mg, Q2W, SC | CZP, 200 mg, Q2W, SC  48 weeks  N_i_ - 736 | | Sustained remission: ASDAS <1.3 | 104 | 32.6  (7.2) | 79  (76.0) | 91 (87.5) | 2.5  (1.7) | 3.8  (2.8) | 4  (3.8) | 1. 16 (15.4) 2. 2 (1.9) 3. 8 (7.7) |  | |
| Placebo |  |  |  | 104 | 31.2  (6.6) | 85  (81.7) | 94 (90.4) | 2.1  (1.7) | 3.1  (1.6) | 7  (6.7) | 1. 17 (16.3) 2. 1 (1.0) 3. 7 (6.7) |  |  |
| COAST-Y^56^ (mixed- axSpA); *Flare^♨^-free (week 52)* | | | | | | | | | | | | | |
| IXE, 80 mg, Q2W | IXE 80 mg, Q2W (N_i_-350) /Q4W (Ni - 423)  24 weeks | | ASDAS <1.3 at weeks 16 20 and <2.1 at both visits. | 54 | 38.4  (10.8) | 40  (74) | 49  (91) | 7.6  (8.4) | 12.9  (8.6) | 8  (15) | NR |  | |
| Placebo |  |  |  | 53 | 38.5  (12.7) | 38  (72) | 45  (85) | 6.6  (7.5) | 12.6  (9.6) | 9  (17) | NR |  |  |
| GO-BACK^57^ (nr-axSpA); *Flare^♨^-free* (*week 52)* | | | | | | | | | | | | | |
| GLM, 50 mg, QM, SC | GLM 50mg, SC, QM  10 months  N_i_ - 323 | | ASDAS <1.3 at both months 7 and 10 | 63 | *31.0  (18–45) | 44  (69.8) | 47 (74.6) | *0.82  (0-5) | *2.1  (0.1–8.5) | NR | n/a |  | |
| Placebo |  |  |  | 62 | *33.0  (18–45) | 46  (74.2) | 44 (71.0) | *0.84  (0-5) | *1.9  (0.1–22.8) | NR | n/a |  |  |
| T2TEASa^60^ (*r- axSpA); Flare rates (week 48/36)* | | | | | | | | | | | | | |
| Stepwise tapering | rhTNFR: Fc (ETN – bs) 50 mg, SC, OW  12 weeks  N_i_ - 311 | | Clinical remission: ASDAS <1.3  N_r_ - 142 | 106 | 30.1  (10.4) | 68  (64.2) | NR | *3.3  (**1.9, 6.4) | NR | NR | NR |  | |
| Placebo |  |  |  | 36 | 29.7  (10.7) | 26  (72.2) | NR | *2  (**1, 6.1) | NR | NR | NR |  |  |
| T2TEASb^60^ (*r- axSpA); Flare rates (week 48/36)* | | | | | | | | | | | | | |
| Stepwise tapering | rhTNFR: Fc (ETN – bs) 50 mg, SC, OW  12 weeks  N_i_ - 311 | | Low disease activity: ASDAS ≥ 1.3 < 2.1  N_r_ - 93 | 53 | 33.5  (12.2) | 42  (79.3) | NR | *5  (**2.3, 7.3) | NR | NR | NR |  | |
| Placebo |  |  |  | 21 | 32.5  (10.2) | 13  (61.9) | NR | *3.17  (**1.3, 5.1) | NR | NR | NR |  |  |
| Switching to biosimilars | | | | | | | | | | | | | |
| NOR-SWITCH^62^ (r- axSpA); *Disease worsening^♨^ (week 52)* | | | | | | | | | | | | | |
| INX bs: CT-P13, 5mg/kg | INX: RP, 5mg/kg; 6 months; | | Stable for atleast 6 months | 46 | 52·8  (10·8) | 38  (83) | 33 | 21·9 (10·2) | NR | 12 (26) | NR |  | |
| INX: RP, 5mg/kg |  |  |  | 45 | 50·0  (10·3) | 38  (84) | 31 | 20·5 (11·2) | NR | 9 (20) | NR |  |  |
| REMSIMA^63^ (r- axSpA); *ASDAS, BASDAI* *(week 52)* | | | | | | | | | | | | | |
| INX bs: CT-P13, 5mg/kg | INX: RP, 5mg/kg. | | Clinical remission/Low disease activity | 38 | 34.89 (6.20) | 33  (86.8) | NR | 10.95  (2.7) | NR | NR | NR |  | |
| INX: RP, 5mg/kg |  |  |  | 40 | 33.37 (8.15) | 37  (92.5) | NR | 11.50  (2.6) | NR | NR | NR |  |  |

EMM: Extra musculoskeletal manifestations; ^ EMM – history of events; ^^ EMM includes both active and history events; IBD: Inflammatory bowel disease; $phase 2 trial; **^¥^**Primary outcome; Q4W: Once every four weeks; Q2W: Once every two weeks; BIW: twice weekly; OW: Once weekly; QM: Once monthly; Q2M: Once every two months; SC: Subcutaneous; IV: Intravenous; SD: Single dose; NR: not reported; n/a: not applicable if the study reported the variable as part of the exclusion criteria. ***^♨^***🡩≥1.1 in the ASDAS score and ASDAS ≥2.1 at any point; ***^♨♨^***flare: ASDAS ≥2.1 at two consecutive visits or ASDAS >3.5 at any time point. Interventional drugs: ADA - Adalimumab; CZP- Certolizumab; ETN- Etanercept; GOL- Golimumab; IXE- Ixekizumab; INX: Infliximab; bs: Bio similar; N_i :_ number of patients included in the induction therapy; N_r :_ number of patients who responded to induction therapy and enrolled in the subsequent trial. Risk of bias: Green – Low, Amber – Unclear, Red – High.

### Table 10: Efficacy outcomes - Binary data (Q5)

| **Study ID**  *Population*  *Time point* | **Treatment arm** | **Response, n (%)** | | **RR**  **(95% CI)** | **NNT** | **Risk of bias** | **Quality of evidence**  **Given the small evidence base confidence has been downgraded for all outcomes (confidence cannot be higher than moderate)** |
| --- | --- | --- | --- | --- | --- | --- | --- |
|  |  | **Intervention** | **Control** |  |  |  |  |
| **Tapering** | | | | | | | |
| **FLARE FREE** | | | | | | | |
| C-OPTIMISE**^55^**  *Mixed axSpA*  *week 48* | **CZP**, 200 mg, Q4W, SC | 83  (79) | 87  (83.7) | 0.9  (0.8, 1.1) | -21.7 |  | **Low confidence:** While tapering may slightly reduce flare free rates when compared to sustaining the maintenance dose there is some benefit of stepwise tapering over delayed tapering.  4 RCTS with a total sample size of 509 and mostly judged as low risk of bias contributed to the evidence (Downgraded for inconsistency, and imprecision). |
| COAST-Y**^56^**  *Mixed axSpA*  *week 40* | **IXE**, 80 mg, Q4W | 40  (83) | 45  (83) | 1.0  (0.8, 1.2) | n/a |  |  |
| GO-BACK**^57^**  *nr-axSpA*  *week 52* | **GLM,** 50 mg, Q2M, SC | 43  (68.3) | 53  (84.1) | 0.8  (0.7, 1.0) | -6.3 |  |  |
| T2TEASb**^60^**  *r- axSpA*  *week 48/36* | Stepwise tapering | 35  (66) | 11  (57. 9) | 1.1  (0.7, 1.8) | 12.3 |  |  |
| **ASAS20** | | | | | | | |
| $ANSWERS**^54^**  *r-axSpA*  *week 24* | Reduced dose:  **ETN,** 25 mg, QW, SC | 14  (63.6) | 20  (90.9) | 0.7  (0.5,1.1) | -4.5 |  | **Low confidence**: On average, the proportion of participants meeting the ASAS20 criteria tapering was consistently lower in the tapering group.  3 RCTs with a total sample size of 382, and all judged as low risk of bias contributed to the evidence.  Downgraded for inconsistency and imprecision. |
| C-OPTIMISE**^55^**  *Mixed axSpA*  *week 96* | **CZP**, 200 mg, Q4W, SC | 82  (78.1) | 89 (85.6) | 0.9  (0.8, 1.0) | -13.4 |  |  |
| GO-BACK**^57^**  *nr-axSpA*  *week 52* | **GLM,** 50 mg, Q2M, SC | 2  (3.2) | 6  (9.5) | 0.3  (0.1, 1.6) | -15.8 |  |  |
| **ASAS40** | | | | | | | |
| $ANSWERS**^54^**  *r-axSpA*  *week 24* | Reduced dose:  **ETN,** 25 mg, QW, SC | 10  (45.5) | 18  (81.8) | 0.6  (0.3, 1.0) | -3.2 |  | **Low confidence**: On average, the proportion of participants meeting the ASAS40 criteria tapering was consistently lower in the tapering group.  In one RCT none of the participants of the total 126 in both the groups met the ASAS40 criteria. Total sample size in the remaining 2 RCTs was 256, one of which was a pilot study with a total of less than 50 participants. All the 3  3 RCTs were judged as low risk of bias.  Downgraded for inconsistency and imprecision. |
| C-OPTIMISE**^55^**  *Mixed axSpA*  *week 96* | **CZP**, 200 mg, Q4W, SC | 77  (73.3) | 88 (84.6) | 0.9  (0.8, 1.0) | -8.9 |  |  |
| GO-BACK**^57^**  *nr-axSpA*  *week 52* | **GLM,** 50 mg, Q2M, SC | 0 | 0 | n/a | n/a |  |  |
| **ASDAS – Clinically Important Improvement (CII): Δ ≥ - 1.1** | | | | | | | |
| C-OPTIMISE**^55^**  *Mixed axSpA*  *week 96* | **CZP**, 200 mg, Q4W, SC | 79  (75.2) | 86 (82.7) | 0.9  (0.8, 1.1) | -13.4 |  | **Low confidence**: One RCT with a sample size of 209 participants and judged as low risk of bias indicate greater proportion of participants receiving the standard maintenance dose achieved clinically important improvement in the ASDAS score compared to those assigned in the tapering group. However, there is uncertainty in the evidence due to the presence of inconsistency, indirectness, and imprecision. |
| **ASDAS – Major Improvement (MI): Δ ≥ -2.0** | | | | | | | |
| C-OPTIMISE**^55^**  *Mixed axSpA*  *week 96* | **CZP**, 200 mg, Q4W, SC | 61  (58.1) | 70 (67.3) | 0.9  (0.7, 1.1) | -10.9 |  | **Low confidence**: One RCT with a sample size of 209 participants and judged as low risk of bias indicate greater proportion of participants receiving the standard maintenance dose achieved major improvement in the ASDAS score compared to those assigned in the tapering group. However, there is uncertainty in the evidence due to the presence of inconsistency, indirectness, and imprecision. |
| **ASDAS: Inactive disease (<1.3)** | | | | | | | |
| C-OPTIMISE**^55^**  *Mixed axSpA*  *week 96* | **CZP**, 200 mg, Q4W, SC | 58/83  (69.9) | 75/87 (86.2) | 0.8  (0.7, 1.0) | -6.1 |  | **Low confidence:** There is insufficient evidence to conclude with certainty the effect of tapering or the continuation of the standard maintenance dose on the proportion of participants achieving inactive disease status.  4 RCTs all but one judged as low risk of bias with a total sample size of 511 contributed to the evidence. (Downgraded for risk of bias, inconsistency, and imprecision). |
| COAST-Y**^56^**  *Mixed axSpA*  *week 40* | **IXE**, 80 mg, Q4W | 29  (60.4) | 29  (53.7) | 1.1  (0.8, 1.6) | 14.9 |  |  |
| GO-BACK**^57^**  *nr-axSpA*  *week 52* | **GLM,** 50 mg, Q2M, SC | 53  (84.1) | 54  (85.7) | 1.0  (0.9, 1.1) | -63 |  |  |
| REDES-TNF**^59^**  *Mixed axSpA*  *week 52* | Reduced dose | 31  (53.5) | 34  (61.4) | 1.0  (0.7, 1.3) | -44.3 |  |  |
| **ASDAS: Low disease activity (<2.1)** | | | | | | | |
| C-OPTIMISE**^55^**  *Mixed axSpA*  *week 96* | **CZP**, 200 mg, Q4W, SC | 19/83  (22.9) | 12/87 (13.8) | 1.7  (0.9, 3.2) | 11 |  | **Low confidence:** There is insufficient evidence to conclude with certainty the effect of tapering or the continuation of the standard maintenance dose on the proportion of participants achieving low disease activity status.  4 RCTs all but one judged as low risk of bias with a total sample size of 432 contributed to the evidence. (Downgraded for risk of bias, inconsistency, and imprecision). |
| COAST-Y**^56^**  *Mixed axSpA*  *week 40* | **IXE**, 80 mg, Q4W | 40  (83.3) | 44  (81.5) | 1.0  (0.9, 1.2) | 54 |  |  |
| **^¥^**NTR3903**^58^**  *r-axSpA*  *week 24* | Reduced dose | 11  (55) | 13  (65) | 0.9  (0.5, 1.4) | -10 |  |  |
| **^¥^**REDES-TNF**^59^**  *Mixed axSpA*  *week 52* | Reduced dose | 48  (80.1) | 51  (84.8) | 0.9  (0.8, 1.1) | -21.3 |  |  |
| **BASDAI50** | | | | | | | |
| $ANSWERS**^54^**  *r-axSpA*  *week 24* | **ETN,** 25 mg, QW, SC | 8  (34.8) | 16  (66.7) | 0.5  (0.3, 1.0) | -3.1 |  | **Moderate confidence**: Proportion of participants experiencing 50% improvement in the BASDAI score seems to be less in the tapering group compared to those continuing to receive the standard maintenance dose.  3 RCTs with a total sample size of 382 and judged as low risk of bias contributed to the evidence.,  Downgraded for imprecision. |
| C-OPTIMISE**^55^**  *Mixed axSpA*  *week 96* | **CZP**, 200 mg, Q4W, SC | 81  (77.1) | 87 (83.7) | 0.9  (0.8, 1.1) | -15.4 |  |  |
| GO-BACK**^57^**  *nr-axSpA*  *week 52* | **GLM,** 50 mg, Q2M, SC | 19  (30.2) | 31  (49.2) | 0.6  (0.4, 1.0) | -5.3 |  |  |
| **Withdrawal** | | | | | | | |
| **FLARE FREE** | | | | | | | |
| ABILITY-3**^61^**  *nr- axSpA*  *week 40* | **ADA,** 40 mg, Q2W, SC | 107  (70) | 72  (47) | 1.5  (1.2, 1.8) | 4.3 |  | **Moderate confidence**: Flare free rates seem to be consistently higher in the group that continued to receive the maintenance dose compared to the withdrawal group. Furthermore, stepwise tapering was found to be more beneficial compared to outright withdrawal of the treatment.    7 RCTs with a total sample size of 961, and all but 2 judged as low risk of bias contributed to the evidence. Downgraded for inconsistency, imprecision, and risk of bias. |
| C-OPTIMISE**^55^**  *Mixed axSpA*  *week 48* | **CZP**, 200 mg, Q2W, SC | 87  (83.7) | 21  (20.2) | 4.1  (2.8, 6.1) | 1.6 |  |  |
| COAST-Y**^56^**  *Mixed axSpA*  *week 40* | **IXE**, 80 mg, Q2W | 45  (83) | 29  (55) | 1.5  (1.2, 2.0) | 3.6 |  |  |
| GO-BACK**^57^**  *nr-axSpA*  *week 52* | **GLM,** 50 mg, QM, SC | 53  (84.1) | 21  (33.9) | 2.5  (1.7, 3.6) | 2 |  |  |
| T2TEASa**^60^**  *r- axSpA*  *week 48/36* | Stepwise tapering | 81  (76.4) | 15  (41.7) | 1.8  (1.2, 2.7) | 2.9 |  |  |
| T2TEASb**^60^**  *r- axSpA*  *week 48/36* | Stepwise tapering | 35  (66) | 8  (38.1) | 1.7  (1.0, 3.1) | 3.6 |  |  |
| **ASAS20** | | | | | | | |
| ABILITY-3**^61^**  *nr- axSpA*  *week 40* | **ADA,** 40 mg, Q2W, SC | 107  (70) | 72  (47) | 1.5  (1.2, 1.8) | 4.3 |  | **Low confidence**: Proportion of participants meeting the ASAS 20 criteria seems to be consistently higher in the group that continued to receive the maintenance dose compared to the withdrawal group.    3 RCTs with a total sample size of 638, and all judged as low risk of bias contributed to the evidence. Downgraded for inconsistency, and imprecision. |
| C-OPTIMISE**^55^**  *Mixed axSpA*  *week 96* | **CZP**, 200 mg, Q2W, SC | 89  (85.6) | 24 (23.1) | 3.7  (2.6, 5.3) | 1.6 |  |  |
| GO-BACK**^57^**  *nr-axSpA*  *week 52* | **GLM,** 50 mg, QM, SC | 6  (9.5) | 0 | 12.8  (0.7, 222.4) | 10.5 |  |  |
| **ASAS40** | | | | | | | |
| ABILITY-3**^61^**  *nr- axSpA*  *week 40* | **ADA,** 40 mg, Q2W, SC | 100  (66) | 70  (46) | 1.5  (1.2, 1.8) | 4.9 |  | **Low confidence**: Proportion of participants meeting the ASAS 40 criteria seems to be higher in the group that continued to receive the maintenance dose compared to the withdrawal group. However, in one RCT none of the participants in either of the groups achieved ASAS40.    3 RCTs with a total sample size of 512, and all judged as low risk of bias contributed to the evidence. Downgraded for inconsistency, and imprecision. |
| C-OPTIMISE**^55^**  *Mixed axSpA*  *week 96* | **CZP**, 200 mg, Q2W, SC | 88  (84.6) | 22 (21.2) | 4.0  (2.7, 5.9) | 1.6 |  |  |
| GO-BACK**^57^**  *nr-axSpA*  *week 52* | **GLM,** 50 mg, QM, SC | 0 | 0 | n/a | n/a |  |  |
| **ASDAS – Clinically Important Improvement (CII): Δ ≥ - 1.1** | | | | | | | |
| ABILITY-3**^61^**  *nr- axSpA*  *week 40* | **ADA,** 40 mg, Q2W, SC | 102  (67) | 69  (45) | 1.5  (1.2, 1.8) | 3.8 |  | **Moderate confidence**: Greater proportion of participants that continued to receive the maintenance dose seem to achieve clinically important improvement in the ASDAS score compared to the withdrawal group.  2 RCTs with a total sample size of 513, and to the evidence. Downgraded for imprecision. |
| C-OPTIMISE**^55^**  *Mixed axSpA*  *week 96* | **CZP**, 200 mg, Q2W, SC | 86  (82.7) | 22 (21.2) | 3.9  (2.7, 5.7) | 1.8 |  |  |
| **ASDAS – Major Improvement (MI): Δ ≥ -2.0** | | | | | | | |
| ABILITY-3**^61^**  *nr- axSpA*  *week 40* | **ADA,** 40 mg, Q2W, SC | 89  (59) | 49  (32) | 1.8  (1.4, 2.4) | 3.8 |  | **Moderate confidence**: Greater proportion of participants that continued to receive the maintenance dose seem to achieve major improvement in the ASDAS score compared to the withdrawal group.  2 RCTs with a total sample size of 513, and to the evidence. Downgraded for imprecision. |
| C-OPTIMISE**^55^**  *Mixed axSpA*  *week 96* | **CZP**, 200 mg, Q2W, SC | 70  (67.3) | 11 (10.6) | 6.4  (3.6, 11.3) | 1.8 |  |  |
| **ASDAS: Inactive disease (<1.3)** | | | | | | | |
| ABILITY-3**^61^**  *nr- axSpA*  *week 40* | **ADA,** 40 mg, Q2W, SC | 87  (57) | 51  (33) | 1.7  (1.3, 2.2) | 4.2 |  | **Moderate confidence**: Greater proportion of participants that continued to receive the maintenance dose seem to achieve inactive disease status compared to the withdrawal group.  4 RCTs with a total sample size of 648 contributed to the evidence. Downgraded for imprecision. |
| C-OPTIMISE**^55^**  *Mixed axSpA*  *week 96* | **CZP**, 200 mg, Q2W, SC | 75/87  (86.2) | 14/24 (58.3) | 1.5  (1.0, 2.1) | 3.6 |  |  |
| COAST-Y**^56^**  *Mixed axSpA*  *week 40* | **IXE**, 80 mg, Q2W | 29  (53.7) | 13  (24.5) | 2.2  (1.3, 3.7) | 3.4 |  |  |
| GO-BACK**^57^**  *nr-axSpA*  *week 52* | **GLM,** 50 mg, QM, SC | 54  (85.7) | 38  (61.3) | 1.4  (1.1, 1.8) | 4.1 |  |  |
| **ASDAS: Low disease activity (<2.1)** | | | | | | | |
| C-OPTIMISE**^55^**  *Mixed axSpA*  *week 96* | **CZP**, 200 mg, Q2W, SC | 12/87  (13.8) | 6/24 (25.0) | 0.6  (0.2, 1.3) | -8.9 |  | **Low confidence:** Proportion of participants achieving low disease activity may be greater among those continuing the maintenance dose compared to the withdrawal group.  2 RCTs with a total sample size of 218 and judged as low risk of bias contributed to the evidence. (Downgraded for inconsistency, and imprecision). |
| COAST-Y**^56^**  *Mixed axSpA*  *week 40* | **IXE**, 80 mg, Q2W | 44  (81.5) | 24  (45.3) | 1.8  (1.3, 2.5) | 2.8 |  |  |
| **BASDAI50** | | | | | | | |
| ABILITY-3**^61^**  *nr- axSpA*  *week 40* | **ADA,** 40 mg, Q2W, SC | 103  (68) | 72  (47) | 1.4  (1.2, 1.8) | 4.8 |  | **Moderate confidence:** Proportion of participants experience 50% improvement in the BASDAI score may be greater among those continuing the maintenance dose compared to the withdrawal group.  3 RCTs with a total sample size of 638 and judged as low risk of bias contributed to the evidence. (Downgraded for imprecision). |
| C-OPTIMISE**^55^**  *Mixed axSpA*  *week 96* | **CZP**, 200 mg, Q2W, SC | 87  (83.7) | 23 (22.1) | 3.8  (2.6, 5.5) | 1.6 |  |  |
| GO-BACK**^57^**  *nr-axSpA*  *week 52* | **GLM,** 50 mg, QM, SC | 31  (49.2) | 15  (24.2) | 2.0  (1.2, 3.4) | 4 |  |  |
| **Switching** | | | | | | | |
| **FLARE FREE** | | | | | | | |
| NOR-SWITCH**^62^**  *Mixed axSpA*  *week 52* | INX bs: CT-P13, 5mg/kg | 32  (69.6) | 28  (62.2) | 1.1  (0.8, 1.5) | 13.6 |  | **Low confidence:** Flare free rate seems to be similar in both the groups that switched to biosimilars and those that continued with the bioorginators.  1 RCT judged as low risk of bias with a sample size of 91 contributed to the evidence. (Downgraded for indirectness and imprecision). |
| **ASDAS: Inactive disease (<1.3)** | | | | | | | |
| NOR-SWITCH**^62^**  *Mixed axSpA*  *week 52* | INX bs: CT-P13, 5mg/kg | 8  (17.4) | 12  (26.7) | 0.7  (0.3, 1.4) | -10.8 |  | **Low confidence:** There is insufficient evidence to conclude with certainty that both biosimilars and bioorginators have similar effect on the proportion of participants achieving inactive disease status.  1 RCT judged as low risk of bias with a sample size of 91 contributed to the evidence. (Downgraded for indirectness and imprecision). |

RR: Relative risk; CI: confidence interval; NNT: Number needed to treat; **^¥^**Primary outcome*;* $phase 2 trial; Q4W: Once every four weeks; Q2W: Once every two weeks; BIW: twice weekly; OW: Once weekly; QM: Once monthly; Q2M: Once every two months; SC: Subcutaneous; IV: Intravenous; SD: Single dose; NR: not reported; n/a: not applicable if the study reported the variable as part of the exclusion criteria. ***^♨^***🡩≥1.1 in the ASDAS score and ASDAS ≥2.1 at any point; ***^♨♨^***flare: ASDAS ≥2.1 at two consecutive visits or ASDAS >3.5 at any time point. Interventional drugs: ADA - Adalimumab; CZP- Certolizumab; ETN- Etanercept; GOL- Golimumab; IXE- Ixekizumab; INX: Infliximab; bs: Bio similar; N_i :_ number of patients included in the induction therapy; N_r :_ number of patients who responded to induction therapy and enrolled in the subsequent trial. Risk of bias: Green – Low, Amber – Unclear, Red – High.

### Table 11: Efficacy outcomes - Continuous data (Q5)

| **Study ID**  *Population*  *Time point* | **Treatment arm (n)** | **Mean CfB**  **(SD/SE^)**  **Median (IQR)^^** | **Standardised Mean Difference (CfB)**  **(95% CI)** | **Risk of bias** | **Quality of evidence** |
| --- | --- | --- | --- | --- | --- |
| **Tapering** | | | | | |
| **ASDAS – CRP mean score (change from baseline – CfB)** | | | | | |
| $ANSWERS**^54^**  *r-axSpA*  *week 24* | **ETN**, 50 mg, OW (24) | -0.1  (0.8) | **Ref** |  | **Low confidence:** Tapering may not result in an improvement of the disease activity compared to the standard dose.  4 RCTS, all but one judged as low risk of bias, with a total sample size of 447 contributed to the evidence.  (Downgraded for risk of bias, and imprecision). |
|  | **ETN,** 25 mg, OW (23) | 0.0  (1.0) | 0.2  (-0.4, 0.7) |  |  |
| C-OPTIMISE**^55^**  *Mixed axSpA*  *week 48* | **CZP**, 200 mg, Q2W, SC (104) | 0.2  (0.1^) | **Ref** |  |  |
|  | **CZP**, 200 mg, Q4W, SC (105) | 0.5  (0.1^) | 0.3  (0.0, 0.6) |  |  |
| NTR3903**^58^**  *r-axSpA*  *week 24* | Standard dose (38) | -0.1  (0.6) | **Ref** |  |  |
|  | Reduced dose (40) | 0.6  (0.7) | 1.2  (0.7, 1.6) |  |  |
| REDES-TNF**^59^**  *Mixed axSpA*  *week 52* | Full dose (55) | 1.1  (0.1^) | **Ref** |  |  |
|  | Reduced dose (58) | 1.1  (0.1^) | 0.0  (-0.3, 0.3) |  |  |
| **BASDAI mean score (change from baseline)** | | | | | |
| C-OPTIMISE**^55^**  *Mixed axSpA*  *week 48* | **CZP**, 200 mg, Q2W, SC (104) | 0.6  (0.2^) | **Ref** |  | **Low confidence:** There is insufficient evidence to conclude with certainty the effect of tapering on disease activity.  3 RCTs, all but one judged as low risk of bias, with a total sample size of 287 contributed to the evidence.  (Downgraded for risk of bias, and imprecision). |
|  | **CZP**, 200 mg, Q4W, SC (105) | 0.8  (0.2^) | 0.1  (-0.2, 0.4) |  |  |
| NTR3903**^58^**  *r-axSpA*  *week 24* | Standard dose (38) | -0.1  (1.5) | **Ref** |  |  |
|  | Reduced dose (40) | 1.3  (1.7) | 0.9  (0.4, 1.4) |  |  |
| REDES-TNF**^55^**  *Mixed axSpA*  *week 52* | Full dose (55) | 1.4  (0.2^) | **Ref** |  |  |
|  | Reduced dose (58) | 1.4  (0.2^) | -0.0  (-0.5, 0.4) |  |  |
| **Spinal pain mean score (change from baseline)** | | | | | |
| ^!^ ANSWERS**^54^**  *r-axSpA*  *week 24* | **ETN,** 50 mg, OW (24) | 0.1  (1.9) | **Ref** |  | **Low confidence:** There is insufficient evidence to conclude with certainty the effect of tapering on spinal pain.  2 RCTs, one judged as low risk of bias while another judged having some concerns, with a total sample size of 160 contributed to the evidence. Both the RCTs specifically measured night pain.  (Downgraded for risk of bias, and imprecision). |
|  | **ETN**, 25 mg, OW (23) | 0.5  (2.4) | 0.2  (-0.4, 0.8) |  |  |
| ^!^ REDES-TNF**^59^**  *Mixed axSpA*  *week 52* | Full dose (55) | 1.4  (0.2^) | **Ref** |  |  |
|  | Reduced dose (58) | 1.4  (0.2^) | 0.4  (-0.3, 0.9) |  |  |
| **Withdrawal** | | | | | |
| **ASDAS – CRP mean score (change from baseline – CfB)** | | | | | |
| C-OPTIMISE**^55^**  *Mixed axSpA*  *week 48* | Placebo (104) | 1.7  (0.1^) | **Ref** |  | **Moderate confidence**: Continuation of standard maintenance dose may reduce disease activity.  1 RCT, with a sample size of 169 and judged as low risk of bias contributed to the evidence.  Downgraded for indirectness. |
|  | **CZP**, 200 mg, Q2W, SC (104) | 0.2  (0.1^) | -1.5  (-1.8, -1.2) |  |  |
| **BASDAI mean score (change from baseline)** | | | | | |
| C-OPTIMISE**^55^**  *Mixed axSpA*  *week 48* | Placebo (104) | 3.0  (0.2^) | **Ref** |  | **Moderate confidence**: Continuation of standard maintenance dose may reduce disease activity.  1 RCT, with a sample size of 208 and judged as low risk of bias contributed to the evidence.  Downgraded for indirectness. |
|  | **CZP**, 200 mg, Q2W, SC (104) | 0.6  (0.2^) | -1.2  (-1.5, -0.9) |  |  |
| **Switching** | | | | | |
| **ASDAS – CRP mean score (change from baseline – CfB)** | | | | | |
| NOR-SWITCH^6^**^2^**  *Mixed axSpA*  *week 52* | **INX: RP**, 5mg/kg (45) | 0.0  (0.6) | **Ref** |  | **Low confidence:** There is insufficient evidence to conclude with certainty the effect of switching to biosimilars on the disease activity, as measured by the change in the absolute values of the ASDAS score.  2 RCTs, one judged while another as high risk of bias, with a total sample size of 169 contributed to the evidence. (Downgraded for risk of bias, inconsistency, and imprecision). |
|  | **INX bs**: CT-P13, 5mg/kg (46) | -0.2  (0.6) | -0.3  (-0.8, 0.1) |  |  |
| REMIMSA^6^**^3^**  *r- axSpA*  *week 52* | **INX: RP**, 5mg/kg (40) | 0.1  (0.4) | **Ref** |  |  |
|  | **INX bs**: CT-P13, 5mg/kg (38) | 0.1  (0.5) | 0.0  (-0.4, 0.5) |  |  |
| **BASDAI mean score (change from baseline)** | | | | | |
| NOR-SWITCH**^62^**  *Mixed axSpA*  *week 52* | **INX: RP**, 5mg/kg (45) | 0.2  (1.0) | **Ref** |  | **Low confidence:** There is insufficient evidence to conclude with certainty the effect of switching to biosimilars on the disease activity.  2 RCTs, one judged while another as high risk of bias, with a total sample size of 169 contributed to the evidence. (Downgraded for risk of bias, and imprecision). |
|  | **INX bs**: CT-P13, 5mg/kg (46) | -0.2  (1.3) | -0.3  (-0.8, 0.1) |  |  |
| REMIMSA**^63^**  *r- axSpA*  *week 52* | **INX: RP**, 5mg/kg (40) | 0.1  (0.4) | **Ref** |  |  |
|  | **INX bs**: CT-P13, 5mg/kg (38) | 0.1  (0.4) | -0.0  (-0.5, 0.4) |  |  |

CI: confidence interval; **^¥^**Primary outcome*;* $phase 2 trial; Q4W: Once every four weeks; Q2W: Once every two weeks; BIW: twice weekly; OW: Once weekly; QM: Once monthly; Q2M: Once every two months; SC: Subcutaneous; IV: Intravenous; SD: Single dose; NR: not reported; n/a: not applicable if the study reported the variable as part of the exclusion criteria. Interventional drugs: ADA - Adalimumab; CZP- Certolizumab; ETN- Etanercept; GOL- Golimumab; IXE- Ixekizumab; INX: Infliximab; bs: Bio similar; N_i :_ number of patients included in the induction therapy; N_r :_ number of patients who responded to induction therapy and enrolled in the subsequent trial. Risk of bias: Green – Low, Amber – Unclear, Red – High; ^µ^adjusted least square means (as reported); ^md^ mean difference between the groups (as reported).

### Table 12: Safety outcomes (Q5)

| **Study** | **Drug** | **Response, n (%)** | | **RR (95% CI)** | **Risk of bias** | **Quality of evidence** |
| --- | --- | --- | --- | --- | --- | --- |
|  |  | **Intervention** | **Control** |  |  |  |
| **Tapering** | | | | | | |
| **Patient with ≥1 AE** | | | | | | |
| C-OPTIMISE**^55^**  *Mixed axSpA*  *week 48* | **CZP**, 200 mg, Q4W, SC | 64  (61.0) | 60  (57.7) | 1.1  (0.8, 1.3) |  | **Moderate confidence**: Proportion of patient experiencing atleast one adverse event seems to be similar between both the tapering and standard dose group.  4 RCTs, all but one judged as low risk of bias, with a sample size of 560 contributed to the evidence.  Downgraded for risk of bias. |
| COAST-Y**^56^**  *Mixed axSpA*  *week 40* | **IXE**, 80 mg, Q4W | 20  (42.6) | 24  (44.4) | 1.0  (0.6, 1.5) |  |  |
| GO-BACK**^57^**  *nr-axSpA*  *week 52* | Full dose**: GLM,** 50 mg, QM, SC | 29  (46) | 30  (46.9) | 1.0  (0.7, 1.4) |  |  |
| REDES-TNF**^59^**  *Mixed axSpA*  *week 52* | Reduced dose | 17  (27.9) | 22  (35.5) | 1.3  (0.8, 2.2) |  |  |
| **Patient with ≥1 SAE** | | | | | | |
| C-OPTIMISE**^55^**  *Mixed axSpA*  *week 48* | **CZP**, 200 mg, Q4W, SC | 0 | 5  (4.8) | 0.1  (0.0,1.6) |  | **Low confidence**: Proportion of patients experiencing any serious adverse events seems to be similar in both the withdrawal and the group receiving the standard maintenance dose.  4 RCTs, all but one judged as low risk of bias with a total sample size of 560 contributed to the evidence.  Downgraded for risk of bias and imprecision. |
| COAST-Y**^56^**  *Mixed axSpA*  *week 40* | **IXE**, 80 mg, Q4W | 2  (4.3) | 2  (3.7) | 1.2  (0.2, 7.8) |  |  |
| GO-BACK**^57^**  *nr-axSpA*  *week 52* | Full dose**: GLM,** 50 mg, QM, SC | 1  (1.6) | 1  (1.6) | 1.0  (0.1, 15.9) |  |  |
| REDES-TNF**^59^**  *Mixed axSpA*  *week 52* | Reduced dose | 11  (18) | 14  (22.6) | 1.3  (0.6, 2.5) |  |  |
| **Patient with ≥1 infection** | | | | | | |
| C-OPTIMISE**^55^**  *Mixed axSpA*  *week 48* | **CZP**, 200 mg, Q4W, SC | 3  (2.9) | 1  (1.0) | 3.0  (0.3, 28.1) |  | **Low confidence:** There is insufficient evidence that tapering may increase the incidence of infections compared to the standard dose group.  3 RCTs, all but one judged as low risk of bias, with a total sample size of 433 contributed to the evidence.  Downgraded for risk of bias and imprecision. |
| COAST-Y**^56^**  *Mixed axSpA*  *week 40* | **IXE**, 80 mg, Q4W | 8  (17) | 13  (24.1) | 0.7  (0.3, 1.6) |  |  |
| REDES-TNF**^59^**  *Mixed axSpA*  *week 52* | Full dose | 15  (7) | 11  (3) | 1.34  (0.67, 2.68) |  |  |
| **EMM - Uveitis** | | | | | | |
| COAST-Y**^56^**  *Mixed axSpA*  *week 40* | **IXE**, 80 mg, Q4W | 2  (4.2) | 3  (5.6) | 0.9  (0.3, 2.6) |  | **Low confidence:** There is insufficient evidence that tapering may increase the incidence of uveitis compared to the standard dose group.  1 RCT, judged as low risk of bias, with a total sample size of 102 contributed to the evidence.  Downgraded for inconsistency and imprecision. |
| **EMM - IBD** | | | | | | |
| COAST-Y**^56^**  *Mixed axSpA*  *week 40* | **IXE**, 80 mg, Q4W | 0 | 0 | n/a |  | **Low confidence:** There is insufficient evidence that tapering may increase the incidence of IBD compared to the standard dose group.  1 RCT, judged as low risk of bias, with a total sample size of 102, did not report any events in either of the groups.  Downgraded for inconsistency and imprecision. |
| **Candida** | | | | | | |
| C-OPTIMISE**^55^**  *Mixed axSpA*  *week 48* | **CZP**, 200 mg, Q4W, SC | 1 (1.0) | 0 |  |  | **Low confidence:** There is insufficient evidence that tapering may increase the incidence of Candidiasis compared to the standard dose group.  2 RCTs, judged as low risk of bias, with a total sample size of 311, contributed to the evidence. 1 RCT did not report any events in either of the groups.  Downgraded for inconsistency and imprecision. |
| COAST-Y**^56^**  *Mixed axSpA*  *week 40* | **IXE**, 80 mg, Q4W | 0 | 0 | n/a |  |  |
| **CVD events** | | | | | | |
| C-OPTIMISE**^55^**  *Mixed axSpA*  *week 48* | **CZP**, 200 mg, Q4W, SC | 0 | 0 | n/a |  | **Low confidence:** There is insufficient evidence that tapering may increase the incidence of CVD events compared to the standard dose group.  2 RCTs, judged as low risk of bias, with a total sample size of 311, did not report any events in either of the groups.  Downgraded for inconsistency and imprecision. |
| COAST-Y**^56^**  *Mixed axSpA*  *week 40* | **IXE**, 80 mg, Q4W | 0 | 0 | n/a |  |  |
| **Malignancies** | | | | | | |
| C-OPTIMISE**^55^**  *Mixed axSpA*  *week 48* | **CZP**, 200 mg, Q4W, SC | 0 | 0 | n/a |  | **Low confidence:** There is insufficient evidence that tapering may increase the incidence of malignancies compared to the standard dose group.  2 RCTs, judged as low risk of bias, with a total sample size of 311, did not report any events in either of the groups.  Downgraded for inconsistency and imprecision. |
| COAST-Y**^56^**  *Mixed axSpA*  *week 40* | **IXE**, 80 mg, Q4W | 0 | 0 | n/a |  |  |
| **Withdrawal** | | | | | | |
| **Patient with ≥1 AE** | | | | | | |
| ABILITY-3**^61^**  *nr- axSpA*  *week 40* | **ADA,** 40 mg, Q2W, SC | 99  (65) | 105  (69) | 1.0  (0.8, 1.1) |  | **Moderate confidence**: Proportion of patients experiencing any adverse events seems to be similar in both the withdrawal and the group receiving the standard maintenance dose.  3 RCTs, judged low risk of bias, with a total sample size of 745 contributed to the evidence.  Downgraded for imprecision. |
| C-OPTIMISE**^55^**  *Mixed axSpA*  *week 48* | **CZP**, 200 mg, Q2W, SC | 60  (57.7) | 56  (54.4) | 1.1  (0.8, 1.4) |  |  |
| COAST-Y**^56^**  *Mixed axSpA*  *week 40* | **IXE**, 80 mg, Q2W | 24  (44.4) | 28  (52.8) | 0.8  (0.6, 1.2) |  |  |
| **Patient with ≥1 SAE** | | | | | | |
| ABILITY-3**^61^**  *nr- axSpA*  *week 40* | **ADA,** 40 mg, Q2W, SC | 1  (1) | 10  (7) | 0.1  (0.0, 0.8) |  | **Low confidence**: There is insufficient evidence that patients monitored using tight control regimen may be at high risk of experiencing an adverse event compared to those receiving standard dose.  3 RCTs judged as low risk of bias with a total sample size of 457 contributed to the evidence.  Downgraded for inconsistency and imprecision. |
| C-OPTIMISE**^55^**  *Mixed axSpA*  *week 48* | **CZP**, 200 mg, Q2W, SC | 5  (4.8) | 0 | 11.0  (0.6, 196.4) |  |  |
| COAST-Y**^56^**  *Mixed axSpA*  *week 40* | **IXE**, 80 mg, Q2W | 2  (3.7) | 1  (1.9) | 2.0  (0.2, 21.0) |  |  |
| **Patient with ≥1 infection** | | | | | | |
| ABILITY-3**^61^**  *nr- axSpA*  *week 40* | **ADA,** 40 mg, Q2W, SC | 80  (53) | 70  (46) | 1.2  (0.9,1.5) |  | **Low confidence:** There is insufficient evidence that withdrawal of treatment may increase the incidence of infections compared to the standard dose group.  3 RCTs, judged as low risk of bias, with a sample size of 620 contributed to the evidence.  Downgraded for inconsistency and imprecision. |
| C-OPTIMISE**^55^**  *Mixed axSpA*  *week 48* | **CZP**, 200 mg, Q2W, SC | 1  (1.0) | 2  (1.9) | 0.5  (0.1, 5.4) |  |  |
| COAST-Y**^56^**  *Mixed axSpA*  *week 40* | **IXE**, 80 mg, Q2W | 13  (24.1) | 18  (34) | 0.7  (0.4, 1.3) |  |  |
| **EMM - Uveitis** | | | | | | |
| COAST-Y**^56^**  *Mixed axSpA*  *week 40* | **IXE**, 80 mg, Q2W | 3  (5.6) | 3  (5.7) | 1.0  (0.4, 2.6) |  | **Low confidence:** There is insufficient evidence that tapering may increase the incidence of uveitis compared to the standard dose group.  1 RCT, judged as low risk of bias, with a total sample size of 107 contributed to the evidence.  Downgraded for inconsistency and imprecision. |
| **EMM - IBD** | | | | | | |
| COAST-Y**^56^**  *Mixed axSpA*  *week 40* | **IXE**, 80 mg, Q2W | 0 | 0 | n/a |  | **Low confidence:** There is insufficient evidence that tapering may increase the incidence of IBD compared to the standard dose group.  1 RCT, judged as low risk of bias, with a total sample size of 107, did not report any events in either of the groups.  Downgraded for inconsistency and imprecision. |
| **Candida** | | | | | | |
| C-OPTIMISE**^55^**  *Mixed axSpA*  *week 48* | **CZP**, 200 mg, Q2W, SC | 0 | 0 | n/a |  | **Low confidence:** There is insufficient evidence that tapering may increase the incidence of Candidiasis compared to the standard dose group.  2 RCTs, judged as low risk of bias, with a total sample size of 315, did not report any events in either of the groups.  Downgraded for inconsistency and imprecision. |
| COAST-Y**^56^**  *Mixed axSpA*  *week 40* | **IXE**, 80 mg, Q2W | 0 | 0 | n/a |  |  |
| **CVD events** | | | | | | |
| C-OPTIMISE**^55^**  *Mixed axSpA*  *week 48* | **CZP**, 200 mg, Q2W, SC | 0 | 0 | n/a |  | **Low confidence:** There is insufficient evidence that tapering may increase the incidence of CVD events compared to the standard dose group.  2 RCTs, judged as low risk of bias, with a total sample size of 315, did not report any events in either of the groups.  Downgraded for inconsistency and imprecision. |
| COAST-Y**^56^**  *Mixed axSpA*  *week 40* | **IXE**, 80 mg, Q2W | 0 | 0 | n/a |  |  |
| **Malignancies** | | | | | | |
| ABILITY-3**^61^**  *nr- axSpA*  *week 40* | **ADA,** 40 mg, Q2W, SC | 0 | 1  (1) | n/a |  | **Low confidence:** There is insufficient evidence that tapering may increase the incidence of malignancies compared to the standard dose group.  3 RCTs, judged as low risk of bias, with a total sample size of 620, contributed to the evidence. 2 RCTs did not report any events in either of the groups.  Downgraded for inconsistency and imprecision. |
| C-OPTIMISE**^55^**  *Mixed axSpA*  *week 48* | **CZP**, 200 mg, Q2W, SC | 0 | 0 | n/a |  |  |
| COAST-Y**^56^**  *Mixed axSpA*  *week 40* | **IXE**, 80 mg, Q2W | 0 | 0 | n/a |  |  |

RR: Relative risk; CI: confidence interval; $phase 2 trial; AE: Adverse events; SAE: Serious adverse events; EMM: Extra musculoskeletal manifestations; IBD: Inflammatory bowel disease; Q4W: Once every four weeks; Q2W: Once every two weeks; BIW: twice weekly; OW: Once weekly; QM: Once monthly; Q2M: Once every two months; SC: Subcutaneous; n/a: not applicable. Interventional drugs: ADA - Adalimumab; CZP- Certolizumab; ETN- Etanercept; GOL- Golimumab; IXE- Ixekizumab; INX: Infliximab; bs: Bio similar; Risk of bias: Green – Low, Amber – Unclear, Red – High.

### Table 13: Observational studies**^Ř^** – Study characteristics

| **Study ID** | **Country, Setting** (Data sources) | **Study design** | **Exposure** | **N** | **Age**,  years Mean (/*median (SD/**IQR) | Male  n (%) | **Disease duration,** years  Mean (/*median (SD/**IQR) | **Previous TNFi**  n (%) | **Previous DMARD** | **EMM**  **Baseline, %** | | | **RoB ^2^** |
| --- | --- | --- | --- | --- | --- | --- | --- | --- | --- | --- | --- | --- | --- |
|  |  |  |  |  |  |  |  |  |  | IBD | Anterior Uveitis | Psoriasis |  |
| **Aydin 2019**^64^ | Turkey, Healthcare records | Retro-spective cohort | TNFi | 1390 | NR | NR | NR | 0% | NR | NR | NR | NR |  |
|  |  |  | TNFi naïve | 62242 |  |  |  |  |  |  |  |  |  |
| **Chao 2020**^65^ | Taiwan, Healthcare records | Retro-spective cohort | Etanercept | 1118 | NR | NR | NR | 0% | NR | n/a | NR | n/a |  |
|  |  |  | Adalimumab | 1713 |  |  |  | 0% |  | n/a |  | n/a |  |
|  |  |  | Golimumab | 556 |  |  |  | 0% |  | n/a |  | n/a |  |
| **Hellgren 2017**^66^ | Sweden, Denmark,  Disease registry | Retro-spective cohort | TNFi | 3078 | *44  **(35-54) | NR | NR | 0% | NR | NR | NR | NR |  |
|  |  |  | TNFi naïve | 7023 | *49  **(8-59) |  |  |  |  |  |  |  |  |
| **Koo 2021**^67^ | Korea, Healthcare records | Retro-spective cohort | Etanercept | 528 | ≥40 yrs: 48.3% | 81.4% | NR |  | NR | n/a | NR | n/a |  |
|  |  |  | Adalimumab | 914 | ≥40 yrs 40.4% | 82.1% |  |  |  | n/a |  | n/a |  |
|  |  |  | Golimumab | 628 | ≥40 yrs: 43.9% | 81.0% |  |  |  | n/a |  | n/a |  |
|  |  |  | Infliximab | 445 | ≥40 yrs 42.9% | 81.6% |  |  |  | n/a |  | n/a |  |
| **Lie 2017**^68^ | Sweden, Healthcare records | Retro-spective cohort | Adalimumab | 328 | 43.5 (12.0) | 71.4% | *12.9  **(4.8, 23.6) | n/a | 24.4 | 8.1 | 19.2 | 3.2 |  |
|  |  |  | Etanercept | 296 | 44.4 (12.2) | 74.6% | *15.5  **(6.5, 24.7) | n/a | 33.3 | 6.8 | 16.4 | 6.2 |  |
|  |  |  | Infliximab | 503 | 43.6 (12.5) | 72.9% | *14.3  **(7.4, 25.1) | n/a | 31.2 | 10.4 | 16.9 | 5.3 |  |
| **Lim 2018**^69^ | Korea, Healthcare records | Retro-spective cohort | TNFi | 170 | ≥40 yrs: 44.4% | 83.4% | NR | n/a | NR | NR | NR | NR |  |
|  |  |  | cDMARDs | 314 | ≥40 yrs: 41.5% | 69.5% | NR | n/a | NR | NR | NR | NR |  |
|  |  |  | non-DMARDs | 595 | ≥40 yrs: 50% | 60.5% | NR | n/a | 0% | NR | NR | NR |  |
| **Lindstrom 2021**^70^  (Mixed PsA/AS): 39-52% AS | Sweden,  Healthcare records | Retro-spective cohort | Secukinuamb | 456 | 48 (13) | 42.0% | NR | NR | 22% | NR | Previous 14% | 14% |  |
|  |  |  | Etanercept | 1800 | 43 (14) | 51.0% | NR | NR | 23% | NR | 11% | 6% |  |
|  |  |  | Infliximab | 783 | 43 (14) | 52.0% | NR | NR | 36% | NR | 12% | 6% |  |
|  |  |  | Golimumab | 500 | 43 (14) | 58.0% | NR | NR | 24% | NR | 18% | 7% |  |
|  |  |  | Certolizumab | 306 | 44 (14) | 41.0% | NR | NR | 27% | NR | 16% | 11% |  |
|  |  |  | Adalimumab | 1006 | 44 (14) | 50.0% | NR | NR | 25% | NR | 18% | 7% |  |
| **Moon 2019** ^71^ | Korea, Healthcare records | Retro-spective cohort | TNFi | 3899 | 38.3 (12.7) | 74.3% | NR | NR | 33.6 (MTX) 79.8 (SLF) | NR | NR | NR |  |
|  |  |  | no TNFi | 10230 | 43.2 (15.9) | 70.5% | NR | NR | 13.0 (MTX) 54.6 (SLF) | NR | NR | NR |  |
|  |  |  | no AxSpA | 70645 | 41.8 (15.3) | 71.5% | n/a | n/a | n/a | n/a | n/a | n/a |  |
| **Moura 2019**^72^ | Canada, Healthcare records | Retro-spective cohort | TNFi +/- DMARD | 369 | 51.1 (14.6) | 62.4% | NR | n/a | 50.60% | 7.50% | NR | NR |  |
|  |  |  | DMARD | 378 |  |  |  |  |  |  |  |  |  |
|  |  |  | current non-use |  |  |  |  |  |  |  |  |  |  |
| **Rahman 2020**^73^ | Canada, Disease registry | Pro-spective cohort | Infliximab | 389 | 45.6 (11.9) | 62.7% | 8.6 (9.8), *4.0 | 8.7 | 25.4% | 16.4% | 28.3% | 12.1% |  |
|  |  |  | Golimumab | 421 | 45.7 (13.3) | 59.1% | 6.0 (10.1), *1.6 | 7.3 | 22.8% | 8.8% | 14.3% | 15.4% |  |
| **Stovall 2021**^74^ | USA, Healthcare records | nested case control | MI cases | 237 | 63.0 (11.3) | 53.6% | NR | NR | NR | NR | NR | 17.70% |  |
|  |  |  | controls | 894 | 62.8 (11.0) | 54.6% |  |  |  |  |  | 19.40% |  |
| **Wendling 2014**^75^ | USA, healthcare records | Retro-spective cohort | Adalimumab | 717 | 41.6 (12.0) | 59.7% | NR | n/a | 19.7% | n/a | 0% | n/a |  |
|  |  |  | Infliximab | 311 | 43.6 (12.7) | 56.5% |  | n/a | 18.0% |  |  |  |  |
|  |  |  | Etanercept | 1087 | 40.5 (12.5) | 62.4% |  | n/a | 20.0% |  |  |  |  |
| **Kim 2020**^76^ | Korea, Disease registry | Retro-spective cohort | Biosimilar | 124 | 38.6 (13.3) | 76.0% | 4.2 (5.2) | 18% | NR | NR | NR | NR |  |
|  |  |  | Infliximab | 124 | 39.7 (13.5) | 73.0% | 4.3 (5.4) | 24% |  |  |  |  |  |

**^Ř^**real world evidence - representative multisite cohorts in people with Axial Spondylitis, comparing types of drug treatments

^1^ Similar as with RCTs, we have excluded studies that included undifferentiated SpA (PsA/AxSpA) – 3 studies were excluded for this reason only. An exception was made for Lindstrom et al. 2021 – added on request.

^2^ Study limitations mainly concern lack of or insufficient adjustment for confounding, co-interventions, or switching of treatments; Risk of bias: Green – Low, Amber – Unclear, Red – High.

^3^ This was the only study that clearly defined the diagnosis as radiographic AxSpA

### Table 14: Observational studies – Safety outcomes

| **Study ID** | **Safety outcome,** follow-up (years) | **Drug treatment** | **n** (events) | **N / person years (PY)** | **absolute risk / incidence rate (per PY)** | **Comparison** | **Effect estimate** | **Variables Adjusted for** | **Conclusion** |
| --- | --- | --- | --- | --- | --- | --- | --- | --- | --- |
| **Any adverse event (AE) or serious adverse events (SAE)** | | | | | | | | | |
| **Rahman 2020**^73^ | **Any AE**  Up to 16 years (2002-2018) | Infliximab (mean dose 4.78 mg/kg over median 17 infusions) | 1687 | 389 | 136 per 100 PY | Golimumab | IRR: 1.04  (no CI) | No adjustment  IRR calculated from raw data in publication | AE: Very low confidence  One prospective and one retrospective cohort study (total sample size 1,007), both high risk of bias. There are inconsistencies in study design, analysis, and outcomes (downgraded for study limitations, precision, consistency).  Findings appear to show that CT-P13 biosimilar has a similar rate of adverse events as Infliximab, and that infliximab has a similar rate of AEs as golimumab, but with very low precision. SAEs appear higher for infliximab, but numbers are small and confidence intervals cannot be estimated. |
|  |  | Golimumab (50mg starting dose monthly, median 14 injections) | 882 | 421 | 131 per 100 PY | ref |  |  |  |
| **Rahman**  **2020**^73^ | **Any SAE**  up to 16 years (2002-2018) | Infliximab | 130 | 389 | 10.5 per 100 PY | Golimumab | IRR: 1.24 | No adjustment  IRR calculated from raw data in publication |  |
|  |  | Golimumab | 57 | 421 | 8.45 per 100 PY | ref |  |  |  |
| **Kim 2020**^76^ | **Any AE event reason for discontinuing medication**  up to 4 years | CT-P13 (biosimilar | 8 | 105 | 0.076 | Infliximab | RR: 1.41  (no CI) | age, sex, baseline BASDAI (propensity score matching)  RR calculated from raw data in publication |  |
|  |  | infliximab | 5 | 92 | 0.054 | ref |  |  |  |
| **Infections – any serious infection** | | | | | | | | | |
| **Moura 2019**^72^ | **Any serious infection, with hospital admission**  up to 12 years | Adalimumab, Infliximab, Golimumab or Etanercept (new) | 20 | 369 | 21 per 1,000 PY |  | HR: 1.00  0.47, 2.11) | Age, sex, socioeconomic status, previous hospital visits, previous hospitalisation for infection, Charlson Comorbidity Index, IBD, NSAIDs, COXIBs, glucocorticoids | **Hospitalisation due to serious infection or sepsis: Low confidence**  Four retrospective cohort studies (total sample size >7459), two low, two high risk of bias.  Findings appear consistent, although comparator is different. Event rates are low, affecting precision (downgraded for precision and study limitations).  TNFi appear to have a similar risk of serious infection compared to those using DMARDs or none of these drugs.  Within TNFi, the risk of serious infection seems to vary, but confidence intervals are wide (and associations not significant). |
|  |  | DMARDs: (hydroxy)chloroquine, leflunomide, SSZ, MTX) | 27 | 378 | 44 per 1,000 PY |  | HR: 0.96  (0.45, 2.04) |  |  |
|  |  | current non-use | 10 | NR | 26 per ,1000 PY | ref |  |  |  |
| **Koo 2021**^67^ | **Any serious infection, with hospital admission**  up to 4 years | Adalimumab (new) | 58 | 914 | 43.33 per 1,000 PY | etanercept | HR: 0.93  (0.61, 1.42) | Age, sex, Charlson Comorbidity Index, type of TNFi, concomitant medication, previous safety events |  |
|  |  | Golimumab (new) | 34 | 628 | 38.52 per 1,000 PY | etanercept | HR: 0.78  (0.49, 1.26) |  |  |
|  |  | Infliximab (new | 46 | 445 | 62.58 per 1,000 PY | etanercept | HR: 1.36  (0.87, 2.12) |  |  |
|  |  | Etanercept (new) | 35 | 528 | 46.54 per 1,000 PY | ref | ref |  |  |
| **Chao 2020**^65^ | **Sepsis**  up to 19 years | Adalimumab (new) | 31 | 1713 | 45.6 per 1,000 PY | Etanercept | IRR: 0.7  (0.4, 1.3) | Not adjusted |  |
|  |  | Golimumab (new) | 7 | 556 | 55.8 per 1,000 PY | Etanercept | IRR: 0.9  (0.4, 2.1) |  |  |
|  |  | Etanercept (new) | 26 | 1118 | 61.0 per 1,000 PY |  | ref |  |  |
| **Rahman 2020**^73^ | **Any Infection**  up to 16 years (2002-2018) | Infliximab | 408 | 389 | 32.9 per 100 PY | Golimumab | IRR: 0.71 | No adjustment  IRR calculated from raw data in publication |  |
|  |  | Golimumab | 309 | 421 | 45.8 per 100 PY | ref |  |  |  |
| **Tuberculosis** | | | | | | | | | |
| **Aydin 2019**^64^ | **Tuberculosis diagnostic code**  2 years | Adalimumab, certolizumab, etanercept, golimumab, infliximab (new) | 1 | 1390 | 0.000719 | anti-TNF naïve (no prescription during study period) | RR: 1.36  (0.19, 9.92) | Not adjusted.  Risk of TB also described for each drug, but not specific for AxSpA. | **Low confidence**  Two retrospective cohort studies (total sample size: 66,147), one high risk of bias.  Findings appear consistent although comparator is different. Event rates are low, affecting precision of estimates (downgraded for precision, study limitations).  TNFI appear to be associated with an increased risk of tuberculosis, compared to those who have not used TNFi. Within this drug category, the risk of TB appears lower for etanercept, but confidence intervals are very wide (association not significant). |
|  |  |  | 33 | 62242 | 0.000530 | TNFi naive | ref |  |  |
| **Koo 2021**^67^ | **Tuberculosis diagnostic code and use of anti-TB agent**  up to 4 years | Adalimumab (new) | 7 | 914 | 5.05 per 1,000 PY | etanercept | HR: 4.28  (0.52, 34.97) | Age, sex, type of TNFi, Charlson Comorbidity Index, concomitant medication, previous safety events |  |
|  |  | Golimumab (new) | 2 | 628 | 2.18 per 1,000 PY | etanercept | HR: 1.66  0.15, 18.46) |  |  |
|  |  | Infliximab (new | 9 | 445 | 11.48 per 1,000 PY | etanercept | HR: 8.4  (1.06, 66.91) |  |  |
|  |  | Etanercept (new) | 1 | 528 | 1.27 per 1,000 PY | ref | ref |  |  |
| **Infections – Herpes zoster** | | | | | | | | | |
| **Lim 2018**^69^ | **Herpes zoster diagnostic code and use of anti-viral agent**  up to 12 years | Adalimumab, Infliximab, or Eternacept (new) | 8 | 170 | 14.1 per 1,000 PY |  | HR: 3.52  (6.1, 27.8 | Age, sex, baseline corticosteroid use.  Subgroup analyses: incidence of HZ higher in women and those older than 50 years, when using cDMARDs or TNFi compared to non-DMARD use, but interaction not tested | **Low confidence**  Two retrospective cohort studies (total sample size: 3,594), both low risk of bias.  Studies examine different comparisons. Event rates are low, affecting precision of estimates (downgraded for precision, consistency).  Both TNFi and cDMARDs are associated with an increased risk of herpes zoster, compared with those not using DMARDs.  Within TNFi, the risk of HZ appears to be lower for etanercept, but the associations are not very strong and not statistically significant. |
|  |  | cDMARDs (sulfasalazine or methotrexate) | 14 | 314 | 16.7 per 1,000 PY |  | HR: 3.7  (9.1, 28.0) |  |  |
|  |  | no DMARDs | 32 | 595 | 9.1 per 1,000 PY | ref |  |  |  |
| **Koo 2021**^67^ | **Herpes zoster code and use of anti-viral agent**  up to 4 years | Adalimumab (new) | 31 | 914 | 22.89 per 1,000 PY | etanercept | HR: 1.55  (0.79, 3.04) | Age, sex, type of TNFi, Charlson Comorbidity Index, concomitant medication, previous safety events |  |
|  |  | Golimumab (new) | 19 | 628 | 21.00 per 1,000 PY | etanercept | HR: 1.17  (0.56, 2.42) |  |  |
|  |  | Infliximab (new | 16 | 445 | 20.84 per 1,000 PY | etanercept | HR: 1.34  0.63, 2.85) |  |  |
|  |  | Etanercept (new) | 12 | 528 | 15.45 per 1,000 PY | ref |  |  |  |
| **Cancer** | | | | | | | | | |
| **Hellgren 2017**^66^ | **First ever invasive cancer after TNFi prescription**  up to 11 years | Adalimumab, certolizumab, etanercept, golimumab, infliximab | 53 | 3078 | 0.017219 | anti-TNF naïve (no prescription during study period) | RR 0.8  (0.6, 1.1) | Age and sex standardised.  Separate data for different types of cancer, but small numbers, low precision. | **Moderate confidence**  One retrospective cohort study, considered low risk of bias (downgraded for consistency)  TNFi is reported not to be associated with an increased risk of cancer. |
|  |  |  | 310 | 7023 | 0.044141 |  |  |  |  |
| **Cardiovascular events** | | | | | | | | | |
| **Stovall 2021**^74^ | MI  Up to 23 years | TNFi (not further specified) | Exposure to TNFi  11 | 135 | 0.081481 | Cases: MI controls: no MI, exposure to TNFi compared with NSAID only | OR: 0.85  0.39, 1.85) | Age, kidney disease, hypertension, IHD, liver disease, peptic ulcer disease, psoriasis, RA, obesity, statin use, smoking, rheumatology visits, ESR or CRP | **MI: Very low confidence**  One case-control study, considered low risk of bias, and small sample size (in terms of exposure rate), and less valid design (downgraded for precision, consistency, design)  TNFi is reported not to be associated with an increased risk (odds) of MI.  **AF: Low confidence**  One retrospective cohort study, large sample size, but considered high risk of bias (downgraded for study limitations and consistency).  Risk of AF seems higher in people with AxSpA using TNFi, but comparison only with people without AxSpA. |
|  |  |  | 49 | 536 | 0.091418 | NSAID use only | ref |  |  |
| **Moon 2019**^71^ | Atrial fibrilliation  mean 3.5 years | TNFi (not further specified) | 23 | 3899 | 1.65 per 1,000 PY |  | HR: 1.60  (1.02, 2.39) | Age, sex, DM, hypertension, dyslipidaemia, congestive heart failure, PAD, COPD, end stage renal disease, previous MI  No HR presented for the comparison of TNFi vs no TNFI in people with AxSpA |  |
|  |  | no TNFI | 91 | 10230 | 0.99 per 1,000 PY |  | HR: 1.21  (0.95, 1.53) |  |  |
|  |  | Healthy - no AxSpA | 372 | 70645 | 0.69 per 1,000 PY | ref |  |  |  |
| **EMM: Uveitis** | | | | | | | | | |
| **Lie 2017**^68^ | Time to first flare of uveitis in those without AU in previous 2 years  up to 8 years | Etanercept | 37 | 270 | NR | Adalimumab | HR: 3.86  (1.85, 8.06) | Age, sex, start-year TNFi, disease duration, history of IBD, baseline CRP, DMARDs use and other medication | **Moderate confidence**  Three retrospective and one prospective cohort studies (total sample size: 10,190.  Three considered low RoB and one high. Findings from the 3 cohorts are consistent. Events rates are low (downgraded for precision).  In three studies (low RoB) etanercept is associated with an increased risk of uveitis compared with adalimumab. Infliximab is reported to be associated with an increased risk of uveitis compared with adalimumab, but precision is low (low event rate).  Findings from one cohort (low RoB) shows increased risk of uveitis for secukinumab.  One study (high RoB) reports lower risk of uveitis for infliximab compared to Golimumab, but CIs could not be calculated. |
|  |  | Infliximab | 24 | 454 | NR | Adalimumab | HR: 1.94  (0.91, 4.16) |  |  |
|  |  | Adalimumab | 6 | 292 | NR | ref |  |  |  |
| **Lindstrom 2021**^70^ | Occurrence of uveitis flare in those without AU in previous 12 months  up to 4 years | Secukinumab | 13 | 456 | 0.029 | Adalimumab | HR: 2.32  (1.16, 4.63) | Age, sex, previous history of uveitis, patient global assessment |  |
|  |  | Etanercept | 13 | 1800 | 0.007 | Adalimumab | HR: 1.82  (1.13, 2.93) |  |  |
|  |  | Infliximab | 52 | 783 | 0.066 | Adalimumab | HR: 0.99  (0.49, 1.96) |  |  |
|  |  | Golimumab | 22 | 500 | 0.044 | Adalimumab | HR: 1.59  (0.90, 2.80) |  |  |
|  |  | Certolizumab | 6 | 306 | 0.020 | Adalimumab | HR: 1.12  (0.44, 2.83) |  |  |
|  |  | Adalimumab | 25 | 1006 | 0.025 | ref |  |  |  |
| **Wendling 2014**^75^ | Uveitis diagnostic code  12 months | Etanercept | 76 | 2115 | 0.036 | Adalimumab | HR: 1.91  (1.10, 3.31) | Age, sex, medication use |  |
|  |  | Infliximab | 10 | 311 | 0.032 | Adalimumab | HR: 1.35  (0.62, 2.95) |  |  |
|  |  | Adalimumab | 49 | 1087 | 0.045 | ref |  |  |  |
| **Rahman 2020**^73^ | Uveitis diagnostic code  up to 16 years (2002-2018) | Infliximab | 15 | 389 | 1.21 per 100 PY | Golimumab | IRR: 0.63 | No adjustment  IRR calculated from raw data in publication |  |
|  |  | Golimumab | 13 | 421 | 1.93 per 100 PY | ref |  |  |  |

**^Ř^**real world evidence - representative multisite cohorts in people with Axial Spondylitis, comparing types of drug treatments

## References

1. Krabbe S, Ostergaard M, Eshed I, Sorensen IJ, Jensen B, Moller JM, et al. Whole-body Magnetic Resonance Imaging in Axial Spondyloarthritis: Reduction of Sacroiliac, Spinal, and Entheseal Inflammation in a Placebo-controlled Trial of Adalimumab. J Rheumatol. 2018;45(5):621-9.
2. van der Heijde D, Cheng-Chung Wei J, Dougados M, Mease P, Deodhar A, Maksymowych WP, et al. Ixekizumab, an interleukin-17A antagonist in the treatment of ankylosing spondylitis or radiographic axial spondyloarthritis in patients previously untreated with biological disease-modifying anti-rheumatic drugs (COAST-V): 16 week results of a phase 3 ra. Lancet. 2018b;392(10163):2441-51.
3. Pedersen SJ, Poddubnyy D, Sorensen IJ, Loft AG, Hindrup JS, Thamsborg G, et al. Course of Magnetic Resonance Imaging-Detected Inflammation and Structural Lesions in the Sacroiliac Joints of Patients in the Randomized, Double-Blind, Placebo-Controlled Danish Multicenter Study of Adalimumab in Spondyloarthritis, as Assessed by the Berl. Arthritis rheumatol. 2016;68(2):418-29.
4. Deodhar A, Gensler LS, Kay J, Maksymowych WP, Haroon N, Landewe R, et al. A Fifty-Two-Week, Randomized, Placebo-Controlled Trial of Certolizumab Pegol in Nonradiographic Axial Spondyloarthritis. Arthritis rheumatol. 2019;71(7):1101-11.
5. Dougados M, van der Heijde D, Sieper J, Braun J, Maksymowych WP, Citera G, et al. Symptomatic efficacy of etanercept and its effects on objective signs of inflammation in early nonradiographic axial spondyloarthritis: a multicenter, randomized, double-blind, placebo-controlled trial. Arthritis rheumatol. 2014;66(8):2091-102.
6. Tu L, Zhao M, Wang X, Kong Q, Chen Z, Wei Q, et al. Etanercept/celecoxib on improving MRI inflammation of active ankylosing spondylitis: A multicenter, open-label, randomized clinical trial. Front. 2022;13:967658.
7. Rusman T, van der Weijden MAC, Nurmohamed MT, Landewe RBM, de Winter JJH, Boden BJH, et al. Is Treatment in Patients With Suspected Nonradiographic Axial Spondyloarthritis Effective? Six-Month Results of a Placebo-Controlled Trial. Arthritis rheumatol. 2021;73(5):806-15.
8. Dougados M, Wood E, Combe B, Schaeverbeke T, Miceli-Richard C, Berenbaum F, et al. Evaluation of the nonsteroidal anti-inflammatory drug-sparing effect of etanercept in axial spondyloarthritis: results of the multicenter, randomized, double-blind, placebo-controlled SPARSE study. Arthritis Res Ther. 2014;16(6):481.
9. Sieper J, van der Heijde D, Dougados M, Maksymowych WP, Scott BB, Boice JA, et al. A randomized, double-blind, placebo-controlled, sixteen-week study of subcutaneous golimumab in patients with active nonradiographic axial spondyloarthritis. Arthritis rheumatol. 2015;67(10):2702-12.
10. Deodhar A, Reveille JD, Harrison DD, Kim L, Lo KH, Leu JH, et al. Safety and Efficacy of Golimumab Administered Intravenously in Adults with Ankylosing Spondylitis: Results through Week 28 of the GO-ALIVE Study. J Rheumatol. 2018;45(3):341-8.
11. Tam LS, Shang Q, Kun EW, Lee KL, Yip ML, Li M, et al. The effects of golimumab on subclinical atherosclerosis and arterial stiffness in ankylosing spondylitis-a randomized, placebo-controlled pilot trial. Rheumatology (Oxford). 2014;53(6):1065-74.
12. Wei JC, Tsou HK, Leong PY, Chen CY, Huang JX. Head-to-Head Comparison of Etanercept vs. Adalimumab in the Treatment of Ankylosing Spondylitis: An Open-Label Randomized Controlled Crossover Clinical Trial. Front Med (Lausanne). 2020;7:566160.
13. Su J, Li M, He L, Zhao D, Wan W, Liu Y, et al. Comparison of the Efficacy and Safety of Adalimumab (Humira) and the Adalimumab Biosimilar Candidate (HS016) in Chinese Patients with Active Ankylosing Spondylitis: A Multicenter, Randomized, Double-Blind, Parallel, Phase III Clinical Trial. BioDrugs. 2020;34(3):381-93.
14. Li J, Xue Z, Wu Z, Bi L, Liu H, Wu L, et al. Comparison of the efficacy and safety of the adalimumab biosimilar TQ-Z2301 and adalimumab for the treatment of Chinese patients with active ankylosing spondylitis: a multi-center, randomized, double-blind, phase III clinical trial. Clin Rheumatol. 2022;41(10):3005-16.
15. Xu H, Li Z, Wu J, Xing Q, Shi G, Li J, et al. IBI303, a biosimilar to adalimumab, for the treatment of patients with ankylosing spondylitis in China: a randomised, double-blind, phase 3 equivalence trial. The lancet rheumatology. 2019;1(1):e35‐e43.
16. Zhao D, He D, Bi L, Wu H, Liu Y, Wu Z, et al. Safety and Efficacy of Prefilled Liquid Etanercept-Biosimilar Yisaipu for Active Ankylosing Spondylitis: A Multi-Center Phase III Trial. Rheumatol. 2021;8(1):361-74.
17. Baraliakos X, Deodhar A, Dougados M, Gensler LS, Molto A, Ramiro S, et al. Safety and Efficacy of Bimekizumab in Patients With Active Ankylosing Spondylitis: Three-Year Results From a Phase IIb Randomized Controlled Trial and Its Open-Label Extension Study. Arthritis rheumatol. 2022a;74(12):1943-58.
18. van der Heijde D, Deodhar A, Baraliakos X, Brown MA, Dobashi H, Dougados M, et al. Efficacy and safety of bimekizumab in axial spondyloarthritis: results of two parallel phase 3 randomised controlled trials. Ann Rheum Dis. 2023;17:17.
19. Wei JC, Kim TH, Kishimoto M, Ogusu N, Jeong H, Kobayashi S. Efficacy and safety of brodalumab, an anti-IL17RA monoclonal antibody, in patients with axial spondyloarthritis: 16-week results from a randomised, placebo-controlled, phase 3 trial. Ann Rheum Dis. 2021;80(8):1014-21.
20. Deodhar AA, Mease PJ, Rahman P, Navarro-Compan V, Strand V, Hunter T, et al. Ixekizumab improves spinal pain, function, fatigue, stiffness, and sleep in radiographic axial Spondyloarthritis: COAST-V/W 52-week results. BMC Rheumatol. 2021a;5(1):35.
21. Deodhar A, Poddubnyy D, Pacheco-Tena C, Salvarani C, Lespessailles E, Rahman P, et al. Efficacy and Safety of Ixekizumab in the Treatment of Radiographic Axial Spondyloarthritis: Sixteen-Week Results From a Phase III Randomized, Double-Blind, Placebo-Controlled Trial in Patients With Prior Inadequate Response to or Intolerance of Tumor Necr. Arthritis rheumatol. 2019b;71(4):599-611.
22. Deodhar A, van der Heijde D, Gensler LS, Kim TH, Maksymowych WP, Ostergaard M, et al. Ixekizumab for patients with non-radiographic axial spondyloarthritis (COAST-X): a randomised, placebo-controlled trial. Lancet. 2020;395(10217):53-64.
23. Deodhar A, Mease P, Rahman P, Navarro-Compan V, Marzo-Ortega H, Hunter T, et al. Ixekizumab Improves Patient-Reported Outcomes in Non-Radiographic Axial Spondyloarthritis: Results from the Coast-X Trial. Rheumatol. 2021b;8(1):135-50.
24. Erdes S, Nasonov E, Kunder E, Pristrom A, Soroka N, Shesternya P, et al. Primary efficacy of netakimab, a novel interleukin-17 inhibitor, in the treatment of active ankylosing spondylitis in adults. Clinical and experimental rheumatology. 2020;38(1):27‐34.
25. Behrens F, Sewerin P, de Miguel E, Patel Y, Batalov A, Dokoupilova E, et al. Efficacy and safety of secukinumab in patients with spondyloarthritis and enthesitis at the Achilles tendon: results from a phase 3b trial. Rheumatology (Oxford). 2022;61(7):2856-66.
26. Braun J, Baraliakos X, Deodhar A, Baeten D, Sieper J, Emery P, et al. Effect of secukinumab on clinical and radiographic outcomes in ankylosing spondylitis: 2-year results from the randomised phase III MEASURE 1 study. Ann Rheum Dis. 2017a;76(6):1070-7.
27. Braun J, Baraliakos X, Deodhar A, Poddubnyy D, Emery P, Delicha EM, et al. Secukinumab shows sustained efficacy and low structural progression in ankylosing spondylitis: 4-year results from the MEASURE 1 study. Rheumatology (Oxford). 2019;58(5):859-68.
28. Braun J, Buehring B, Baraliakos X, Gensler LS, Porter B, Quebe-Fehling E, et al. Effects of secukinumab on bone mineral density and bone turnover biomarkers in patients with ankylosing spondylitis: 2-year data from a phase 3 study, MEASURE 1. BMC Musculoskelet Disord. 2021;22(1):1037.
29. Deodhar AA, Dougados M, Baeten DL, Cheng-Chung Wei J, Geusens P, Readie A, et al. Effect of Secukinumab on Patient-Reported Outcomes in Patients With Active Ankylosing Spondylitis: A Phase III Randomized Trial (MEASURE 1). Arthritis rheumatol. 2016;68(12):2901-10.
30. Tseng JC, Wei JC, Deodhar A, Martin R, Porter B, McCreddin S, et al. Secukinumab Demonstrates Sustained Efficacy and Safety in a Taiwanese Subpopulation With Active Ankylosing Spondylitis: Four-Year Results From a Phase 3 Study, MEASURE 1. Front. 2020;11:561748.
31. Marzo-Ortega H, Sieper J, Kivitz A, Blanco R, Cohen M, Delicha EM, et al. Secukinumab provides sustained improvements in the signs and symptoms of active ankylosing spondylitis with high retention rate: 3-year results from the phase III trial, MEASURE 2. RMD Open. 2017a;3(2):e000592.
32. Marzo-Ortega H, Sieper J, Kivitz A, Blanco R, Cohen M, Martin R, et al. Secukinumab and Sustained Improvement in Signs and Symptoms of Patients With Active Ankylosing Spondylitis Through Two Years: Results From a Phase III Study. Arthritis Care Res (Hoboken). 2017b;69(7):1020-9.
33. Sieper J, Deodhar A, Marzo-Ortega H, Aelion JA, Blanco R, Jui-Cheng T, et al. Secukinumab efficacy in anti-TNF-naive and anti-TNF-experienced subjects with active ankylosing spondylitis: results from the MEASURE 2 Study. Ann Rheum Dis. 2017;76(3):571-92.
34. Pavelka K, Kivitz A, Dokoupilova E, Blanco R, Maradiaga M, Tahir H, et al. Efficacy, safety, and tolerability of secukinumab in patients with active ankylosing spondylitis: a randomized, double-blind phase 3 study, MEASURE 3. Arthritis Res Ther. 2017;19(1):285.
35. Pavelka K, Kivitz AJ, Dokoupilova E, Blanco R, Maradiaga M, Tahir H, et al. Secukinumab 150/300 mg Provides Sustained Improvements in the Signs and Symptoms of Active Ankylosing Spondylitis: 3-Year Results from the Phase 3 MEASURE 3 Study. ACR Open Rheumatol. 2020;2(2):119-27.
36. Kivitz AJ, Wagner U, Dokoupilova E, Supronik J, Martin R, Talloczy Z, et al. Efficacy and Safety of Secukinumab 150 mg with and Without Loading Regimen in Ankylosing Spondylitis: 104-week Results from MEASURE 4 Study. Rheumatol. 2018;5(2):447-62.
37. Huang F, Sun F, Wan WG, Wu LJ, Dong LL, Zhang X, et al. Secukinumab provided significant and sustained improvement in the signs and symptoms of ankylosing spondylitis: results from the 52-week, Phase III China-centric study, MEASURE 5. Chin Med J. 2020;133(21):2521-31.
38. Deodhar A, Blanco R, Dokoupilova E, Hall S, Kameda H, Kivitz AJ, et al. Improvement of Signs and Symptoms of Nonradiographic Axial Spondyloarthritis in Patients Treated With Secukinumab: Primary Results of a Randomized, Placebo-Controlled Phase III Study. Arthritis rheumatol. 2021e;73(1):110-20.
39. Sieper J, Braun J, Kay J, Badalamenti S, Radin AR, Jiao L, et al. Sarilumab for the treatment of ankylosing spondylitis: results of a Phase II, randomised, double-blind, placebo-controlled study (ALIGN). Ann Rheum Dis. 2015;74(6):1051-7.
40. Baeten D, Ostergaard M, Wei JC, Sieper J, Jarvinen P, Tam LS, et al. Risankizumab, an IL-23 inhibitor, for ankylosing spondylitis: results of a randomised, double-blind, placebo-controlled, proof-of-concept, dose-finding phase 2 study. Ann Rheum Dis. 2018;77(9):1295-302.
41. van der Heijde D, Baraliakos X, Gensler LS, Maksymowych WP, Tseluyko V, Nadashkevich O, et al. Efficacy and safety of filgotinib, a selective Janus kinase 1 inhibitor, in patients with active ankylosing spondylitis (TORTUGA): results from a randomised, placebo-controlled, phase 2 trial. Lancet. 2018;392(10162):2378-87.
42. Maksymowych WP, Ostergaard M, Landewe R, Barchuk W, Liu K, Gilles L, et al. Filgotinib decreases both vertebral body and posterolateral spine inflammation in ankylosing spondylitis: results from the TORTUGA trial. Rheumatology (Oxford). 2022;61(6):2388-97.
43. van der Heijde D, Deodhar A, Wei JC, Drescher E, Fleishaker D, Hendrikx T, et al. Tofacitinib in patients with ankylosing spondylitis: a phase II, 16-week, randomised, placebo-controlled, dose-ranging study. Ann Rheum Dis. 2017;76(8):1340-7.
44. Deodhar A, Sliwinska-Stanczyk P, Xu H, Baraliakos X, Gensler LS, Fleishaker D, et al. Tofacitinib for the treatment of ankylosing spondylitis: a phase III, randomised, double-blind, placebo-controlled study. Ann Rheum Dis. 2021;80(8):1004-13.
45. Navarro-Compan V, Wei JC, Van den Bosch F, Magrey M, Wang L, Fleishaker D, et al. Effect of tofacitinib on pain, fatigue, health-related quality of life and work productivity in patients with active ankylosing spondylitis: results from a phase III, randomised, double-blind, placebo-controlled trial. RMD Open. 2022;8(2):06.
46. van der Heijde D, Song IH, Pangan AL, Deodhar A, van den Bosch F, Maksymowych WP, et al. Efficacy and safety of upadacitinib in patients with active ankylosing spondylitis (SELECT-AXIS 1): a multicentre, randomised, double-blind, placebo-controlled, phase 2/3 trial. Lancet. 2019;394(10214):2108-17.
47. van der Heijde D, Deodhar A, Maksymowych WP, Sieper J, Van den Bosch F, Kim TH, et al. Upadacitinib in active ankylosing spondylitis: results of the 2-year, double-blind, placebo-controlled SELECT-AXIS 1 study and open-label extension. RMD Open. 2022;8(2):07.
48. Deodhar A, Van den Bosch F, Poddubnyy D, Maksymowych WP, van der Heijde D, Kim TH, et al. Upadacitinib for the treatment of active non-radiographic axial spondyloarthritis (SELECT-AXIS 2): a randomised, double-blind, placebo-controlled, phase 3 trial. Lancet. 2022;400(10349):369-79.
49. van der Heijde D, Baraliakos X, Sieper J, Deodhar A, Inman RD, Kameda H, et al. Efficacy and safety of upadacitinib for active ankylosing spondylitis refractory to biological therapy: a double-blind, randomised, placebo-controlled phase 3 trial. Ann Rheum Dis. 2022;81(11):1515-23.
50. Taylor PC, van der Heijde D, Landewe R, McCue S, Cheng S, Boonen A. A Phase III Randomized Study of Apremilast, an Oral Phosphodiesterase 4 Inhibitor, for Active Ankylosing Spondylitis. J Rheumatol. 2021;48(8):1259-67.
51. Michielsens CA, den Broeder N, van den Hoogen FH, Mahler EA, Teerenstra S, van der Heijde D, et al. Treat-to-target dose reduction and withdrawal strategy of TNF inhibitors in psoriatic arthritis and axial spondyloarthritis: a randomised controlled non-inferiority trial. Ann Rheum Dis. 2022;81(10):1392-9.
52. Syversen SW, Goll GL, Jorgensen KK, Sandanger O, Sexton J, Olsen IC, et al. Effect of Therapeutic Drug Monitoring vs Standard Therapy During Infliximab Induction on Disease Remission in Patients With Chronic Immune-Mediated Inflammatory Diseases: A Randomized Clinical Trial. Jama. 2021;325(17):1744-54.
53. Molto A, Lopez-Medina C, Van den Bosch FE, Boonen A, Webers C, Dernis E, et al. Efficacy of a tight-control and treat-to-target strategy in axial spondyloarthritis: results of the open-label, pragmatic, cluster-randomised TICOSPA trial. Ann Rheum Dis. 2021;80(11):1436-44.
54. Yates M, Hamilton LE, Elender F, Dean L, Doll H, MacGregor AJ, et al. Is Etanercept 25 mg Once Weekly as Effective as 50 mg at Maintaining Response in Patients with Ankylosing Spondylitis? A Randomized Control Trial. J Rheumatol. 2015;42(7):1177-85.
55. Landewe RB, Gensler LS, Poddubnyy D, Rahman P, Hojnik M, Li X, et al. Continuing versus withdrawing ixekizumab treatment in patients with axial spondyloarthritis who achieved remission: efficacy and safety results from a placebo-controlled, randomised withdrawal study (COAST-Y). Ann Rheum Dis. 2021;80(8):1022-30.
56. Landewe RB, van der Heijde D, Dougados M, Baraliakos X, Van den Bosch FE, Gaffney K, et al. Maintenance of clinical remission in early axial spondyloarthritis following certolizumab pegol dose reduction. Ann Rheum Dis. 2020;79(7):920-8.
57. Weinstein CLJ, Sliwinska-Stanczyk P, Hala T, Stanislav M, Tzontcheva A, Yao R, et al. Efficacy and safety of golimumab in patients with non-radiographic axial spondyloarthritis: a withdrawal and retreatment study (GO-BACK). Rheumatology (Oxford). 2023;15:15.
58. Ruwaard J, Mj LA, Kneepkens EL, Krieckaert C, Nurmohamed MT, Hooijberg F, et al. Interval prolongation of etanercept in rheumatoid arthritis, ankylosing spondylitis, and psoriatic arthritis: a randomized controlled trial. Scand J Rheumatol. 2023;52(2):129-36.
59. Gratacos J, Pontes C, Juanola X, Sanz J, Torres F, Avendano C, et al. Non-inferiority of dose reduction versus standard dosing of TNF-inhibitors in axial spondyloarthritis. Arthritis Res Ther. 2019;21(1):11.
60. Zhang T, Zhu J, He D, Chen X, Wang H, Zhang Y, et al. Disease activity guided stepwise tapering or discontinuation of rhTNFR:Fc, an etanercept biosimilar, in patients with ankylosing spondylitis: a prospective, randomized, open-label, multicentric study. Ther. 2020;12:1759720X20929441.
61. Landewe R, Sieper J, Mease P, Inman RD, Lambert RG, Deodhar A, et al. Efficacy and safety of continuing versus withdrawing adalimumab therapy in maintaining remission in patients with non-radiographic axial spondyloarthritis (ABILITY-3): a multicentre, randomised, double-blind study. Lancet. 2018;392(10142):134-44.
62. Jorgensen KK, Olsen IC, Goll GL, Lorentzen M, Bolstad N, Haavardsholm EA, et al. Switching from originator infliximab to biosimilar CT-P13 compared with maintained treatment with originator infliximab (NOR-SWITCH): a 52-week, randomised, double-blind, non-inferiority trial. Lancet. 2017;389(10086):2304-16.
63. Mathkhor AJ, Altaqi A, Abdullah A, Khudhairy A. Patients with Ankylosing Spondylitis Can Maintain Clinical and Functional Improvement after Switching from Infliximab Reference Product to Infliximab Biosimilar (REMSIMA): 12 Months Comparative Open-Label Study. Journal of Clinical Rheumatology and Immunology. 2022;22(2):67-72.
64. Aydin V et al. Relative risk of tuberculosis in patients with rheumatic diseases managed with anti-tumour necrosis factor-alpha therapy: A nationwide cohort study. Journal of Clinical Pharmacy & Therapeutics 2019;44:553-560.
65. Chao WC et al. Factors associated with sepsis risk in immune-mediated inflammatory diseases receiving tumor necrosis factor inhibitors: a nationwide study. Therapeutic Advances in Musculoskeletal Disease 2020;12:1-10.
66. Hellgren K et al. Cancer risk in patients with spondyloarthritis treated with TNF inhibitors: a collaborative study from the ARTIS and DANBIO registers. Annals of the Rheumatic Diseases 2017;76:105-111.
67. Koo BS et al. The risk factors and incidence of major infectious diseases in patients with ankylosing spondylitis receiving tumor necrosis factor inhibitors. Modern Rheumatology 2021;13(6):1192-1201.
68. Lie E et al. Tumour necrosis factor inhibitor treatment and occurrence of anterior uveitis in ankylosing spondylitis: results from the Swedish biologics register. Ann Rheum Dis 2017;76:1515-1521
69. Lim DH et al. The risk of herpes zoster in patients with ankylosing spondylitis: Analysis of the Korean National Health Insurance Service - Sample cohort database. Modern Rheumatology 2018;28:168-173.
70. Lindstrom U et al. Anterior uveitis in patients with spondyloarthritis treated with secukinumab or tumour necrosis factor inhibitors in routine care: Does the choice of biological therapy matter? Ann Rheum Dis 2021;80:1445-1452.
71. Moon I et al. Ankylosing spondylitis: A novel risk factor for atrial fibrillation - A nationwide population-based study. International Journal of Cardiology 2019;275:77-82.
72. Moura CS et al. Use of disease-modifying anti-rheumatic or anti-tumour necrosis factor drugs and risk of hospitalized infection in ankylosing spondylitis. Scand J Rheumatol 2019;48:121-127.
73. Rahman P et al. Long-term effectiveness and safety of infliximab and golimumab in ankylosing spondylitis patients from a Canadian prospective observational registry. BMC Rheumatol 2020;4:56.
74. Stovall R et al. Relation of therapies for ankylosing spondylitis and psoriatic arthritis to risk of myocardial infarction: a nested case control study. BMC Rheumatology 2021;5:36.
75. Wendling D et al. Comparing the risk of developing uveitis in patients initiating anti-tumor necrosis factor therapy for ankylosing spondylitis: an analysis of a large US claims database. Curr Med Res Opin 2014;30(12):2515-21.
76. Kim HA et al. Retention Rate and Efficacy of the Biosimilar CT-P13 Versus Reference Infliximab in Patients with Ankylosing Spondylitis: A Propensity Score-Matched Analysis from the Korean College of Rheumatology Biologics Registry. BioDrugs 2020; 34:529-539.
