# Supplementary material for: The 2025 British Society for Rheumatology guideline for the treatment of axial spondyloarthritis with biologic and targeted synthetic DMARDs
Source: Rheumatology (Oxford). 2025 Apr 9;64(6):3242–54. doi: 10.1093/rheumatology/keaf089 (PMC12107049; doi:10.1093/rheumatology/keaf089)
Supplement: keaf089_Supplementary_Data [file keaf089_supplementary_data.zip › keaf089_Supplementary_Data/rhe-24-2017-File003.docx]

**The 2024 BSR guideline for the treatment of axial spondyloarthritis with biologic and targeted synthetic DMARDs**

**Supplementary Data S2. Search strategies**

The following table is an explanation of the symbols used in the search strategies below.

| **Symbols** | **Explanation** | **Platform** |
| --- | --- | --- |
| / | indicates an index term (MeSH) | Ovid |
| exp | before an index term indicates that all subheadings were selected | Ovid |
| ad, ae, ai, de, tu, to | after an index term indicates term qualified by ≥1 of following subheadings Administration & Dosage, Adverse Effects, Antagonists & Inhibitors, Drug effects, Therapeutic Use, Toxicity | Ovid |
| ae, ct, ad, cb, cm, it, dt, to, im, iv, po, pv, sc, tm | after an index term indicates term qualified by ≥1 of following subheadings Adverse Drug Reaction, Clinical Trial, Drug Administration, Drug Combination, Drug Comparison, Drug Interaction, Drug Therapy, Drug Toxicity, Intramuscular Drug Administration, Intravenous Drug Administration, Oral Drug Administration, Special Situation for Pharmacovigilance, Subcutaneous Drug Administration, Unexpected Outcome of Drug Treatment | Ovid |
| .ab. | indicates a search for a term in the abstract | Ovid |
| .pt. | indicates a search for term in publication type | Ovid |
| .sh. | indicates a search for a term in subject heading | Ovid |
| .ti. | indicates a search for a term in the title | Ovid |
| .ti,ab,kf | indicates a search for a term in the title/abstract/word(s) in keyword | Ovid |
| :ti,ab,kw | indicates a search for a term in the title/abstract/word(s) in keyword | Cochrane |
| * | at the end of a term indicates that this term has been truncated. | Ovid, Cochrane |
| # | mandated wild card character stands for one character within a word or at the end of a word. | Ovid |
| ? | optional wild card character stands for zero or one character within a word or at the end of a word. | Ovid |
| adj | indicates a search for two terms where they appear adjacent to each another | Ovid |
| adj*n* | indicates a search for two terms where they appear within *n* words of each another | Ovid |
| NEXT | indicates a search for two terms where they appear adjacent to each another | Cochrane |
| NEAR/n | indicates a search for two terms where they appear within *n* words of each another | Cochrane |

# Search filters (study design)

RCT search filters (see https://training.cochrane.org/handbook/version-6/chapter-4-tech-suppl)

- MEDLINE - Cochrane RCT Filter: sensitivity- and precision-maximizing version (2008 revision)
- EMBASE - Cochrane Highly Sensitive Search Strategy for identifying controlled trials in Embase:

(2018 revision); Ovid format (Glanville et al 2019b)

Controlled non-randomised studies – sensitive

- Waffenschmidt S,Navarro-Ruan T, Hobson N, Hausner E,Sauerland S, Haynes RB. Development and validation of study filters for identifying controllednon-randomized studies in PubMed and OvidMEDLINE.Res Syn Meth. 2020;11:617–626. https://doi.org/10.1002/jrsm.1425626

# Medline (OvidSP)

| 1 | exp Axial Spondyloarthritis/ |
| --- | --- |
| 2 | Spondylarthropathies/ |
| 3 | Spondylarthritis/ |
| 4 | Spondylitis/ |
| 5 | Sacroiliitis/ |
| 6 | ankylosi*.ti,ab,kf. [pick up only ankylosing or ankylosis, not ankylosed] |
| 7 | spondyl?arthr*.ti,ab,kf. |
| 8 | spondylitis.ti,ab,kf. |
| 9 | sacroili?tis.ti,ab,kf. |
| 10 | spondylodiscitis.ti,ab,kf. |
| 11 | (bekhtere* or bechtere*).ti,ab,kf. |
| 12 | axSpA.ti,ab,kf. [will pick up nr axSpA, nr-axSpA] |
| 13 | nraxSpA.ti,ab,kf. |
| 14 | (axial adj3 SpA).ti,ab,kf. |
| 15 | AS.ti,ab,kf. |
| 16 | marie stru*.ti,ab,kf. |
| 17 | marie* disease*.ti,ab,kf. |
| 18 | bamboo spine.ti,ab,kf. |
| 19 | (spin* adj3 (arthrit* or arthropath*)).ti,ab,kf. |
| 20 | or/1-19 |
| 21 | Biological products/ |
| 22 | Biosimilar Pharmaceuticals/ |
| 23 | antirheumatic agents/ |
| 24 | biosimilar*.ti,ab,kf. |
| 25 | (targeted adj3 (DMARD* or disease modify* or antirheumatic* or anti rheumatic*)).ti,ab,kf. |
| 26 | tsDMARD*.ti,ab,kf. |
| 27 | targeted synthetic*.ti,ab,kf. [Consider focusing more?] |
| 28 | bDMARD*.ti,ab,kf. |
| 29 | (biologic* adj3 (DMARD* or disease modify* or antirheumatic* or anti rheumatic*)).ti,ab,kf. |
| 30 | (biologic* adj3 (drug* or medication* or medicine* or pharmaceut*)).ti,ab,kf. |
| 31 | Phosphodiesterase Inhibitors/ |
| 32 | exp Phosphodiesterase 4 Inhibitors/ |
| 33 | ((phosphodiesterase 4 or phosphodiesterase IV) adj3 (inhibitor* or antagonist* or blocker*)).ti,ab,kf. |
| 34 | (PDE4i or PDE 4i or ((PDE4 or PDE 4) adj3 (inhibitor* or antagonist* or blocker*))).ti,ab,kf. |
| 35 | ((PDEiv or PDE iv) adj3 (inhibitor* or antagonist* or blocker*)).ti,ab,kf. |
| 36 | apremilast.ti,ab,kf. |
| 37 | Otelza*.ti,ab,kf. |
| 38 | exp Janus Kinases/ad, ae, ai, de, tu, to [Administration & Dosage, Adverse Effects, Antagonists & Inhibitors, Drug effects, Therapeutic Use, Toxicity] |
| 39 | Janus Kinase Inhibitors/ |
| 40 | ((JAK or janus kinase or tyrosine kinase or TYK) adj3 (inhibitor* or antagonist* or blocker*)).ti,ab,kf. |
| 41 | (anti adj (JAK* or janus kinase or tyrosine kinase or TYK)).ti,ab,kf. |
| 42 | ((JAK or JAK1 or JAK2 or JAK3 or TYK2) adj3 (blocker* or inhibitor* or antagonist*)).ti,ab,kf. |
| 43 | (JAKi* or JAK1i* or JAK2i* or JAK3i* or TYK2i*).ti,ab,kf. |
| 44 | (antiJAK* or antiTYK*).ti,ab,kf. |
| 45 | tofacitinib.ti,ab,kf. |
| 46 | Xeljanz*.ti,ab,kf. |
| 47 | upadacitinib.ti,ab,kf. |
| 48 | Rinvoq*.ti,ab,kf. |
| 49 | filgotinib.ti,ab,kf. |
| 50 | Jyseleca*.ti,ab,kf. |
| 51 | baricitinib.ti,ab,kf. |
| 52 | Olumiant*.ti,ab,kf. |
| 53 | NF-kappa B/ad, ae, ai, de, tu, to |
| 54 | ((NF kB or kappa B) adj3 (inhibitor* or antagonist* or blocker*)).ti,ab,kf. |
| 55 | (anti adj (NF kB or kappa B)).ti,ab,kf. |
| 56 | Iguratimod.ti,ab,kf. |
| 57 | exp Monokines/ad, ae, ai, de, tu, to |
| 58 | exp Antibodies, Monoclonal/ |
| 59 | exp Tumor Necrosis Factor Inhibitors/ |
| 60 | Tumor Necrosis Factor-alpha/ad, ae, ai, de, tu, to |
| 61 | exp Receptors, Tumor Necrosis Factor/ad, ae, ai, de, tu, to |
| 62 | ((tumo?r necrosis or TNF*) adj3 (inhibitor* or antagonist* or blocker*)).ti,ab,kf. |
| 63 | anti tumo?r necrosis.ti,ab,kf. |
| 64 | (TNF?i or anti TNF* or antiTNF*).ti,ab,kf. |
| 65 | adalimumab/ |
| 66 | Adalimumab.ti,ab,kf. |
| 67 | Humira*.ti,ab,kf. |
| 68 | Abrilada*.ti,ab,kf. |
| 69 | Amgevita*.ti,ab,kf. |
| 70 | Amsparity*.ti,ab,kf. |
| 71 | Cyltezo*.ti,ab,kf. |
| 72 | Hadlima*.ti,ab,kf. |
| 73 | Halimatoz*.ti,ab,kf. |
| 74 | Hefiya*.ti,ab,kf. |
| 75 | Hulio*.ti,ab,kf. |
| 76 | Hyrimoz*.ti,ab,kf. |
| 77 | Idacio*.ti,ab,kf. |
| 78 | Imraldi*.ti,ab,kf. |
| 79 | Kromeya*.ti,ab,kf. |
| 80 | Solymbic*.ti,ab,kf. |
| 81 | Trudexa*.ti,ab,kf. |
| 82 | Yuflyma*.ti,ab,kf. |
| 83 | certolizumab pegol/ |
| 84 | Certolizumab.ti,ab,kf. |
| 85 | Cimzia*.ti,ab,kf. |
| 86 | Etanercept/ |
| 87 | Etanercept.ti,ab,kf. |
| 88 | Enbrel*.ti,ab,kf. |
| 89 | Altebrel*.ti,ab,kf. |
| 90 | Avent*.ti,ab,kf. |
| 91 | Benepali*.ti,ab,kf. |
| 92 | Brenzys*.ti,ab,kf. |
| 93 | Davictrel*.ti,ab,kf. |
| 94 | Erelzi*.ti,ab,kf. |
| 95 | Etacept*.ti,ab,kf. |
| 96 | Etanar*.ti,ab,kf. |
| 97 | Eticovo*.ti,ab,kf. |
| 98 | Eucept*.ti,ab,kf. |
| 99 | Infinitam*.ti,ab,kf. |
| 100 | Nanercept*.ti,ab,kf. |
| 101 | Nepexto*.ti,ab,kf. |
| 102 | Qiangke*.ti,ab,kf. |
| 103 | Yisaipu*.ti,ab,kf. |
| 104 | Golimumab.ti,ab,kf. |
| 105 | Simponi*.ti,ab,kf. |
| 106 | Infliximab/ |
| 107 | Infliximab.ti,ab,kf. |
| 108 | Remicade*.ti,ab,kf. |
| 109 | Avsola*.ti,ab,kf. |
| 110 | Baimaibo*.ti,ab,kf. |
| 111 | Flixabi*.ti,ab,kf. |
| 112 | Flammegis*.ti,ab,kf. |
| 113 | Inflectra*.ti,ab,kf. |
| 114 | Infimab*.ti,ab,kf. |
| 115 | Ixifi*.ti,ab,kf. |
| 116 | Remsima*.ti,ab,kf. |
| 117 | Renflexis*.ti,ab,kf. |
| 118 | Zessly*.ti,ab,kf. |
| 119 | exp interleukin/ad, ae, ai, de, tu, to |
| 120 | exp receptors, interleukin/ad, ae, ai, de, tu, to |
| 121 | Interleukin 1 Receptor Antagonist Protein/ |
| 122 | ((interleukin 1 or IL 1 or IL1) adj3 (inhibitor* or antagonist* or blocker*)).ti,ab,kf. |
| 123 | anakinra.ti,ab,kf. |
| 124 | kineret*.ti,ab,kf. |
| 125 | ((interleukin 6* or IL6* or IL 6*) adj3 (inhibitor* or antagonist* or blocker*)).ti,ab,kf. |
| 126 | tocilizumab.ti,ab,kf. |
| 127 | actemra*.ti,ab,kf. |
| 128 | roActemra.ti,ab,kf. |
| 129 | Sarilumab.ti,ab,kf. |
| 130 | Kevzara*.ti,ab,kf. |
| 131 | ((Interleukin 17* or IL 17* or IL17*) adj3 (inhibitor* or antagonist* or blocker*)).ti,ab,kf. |
| 132 | Ixekizumab.ti,ab,kf. |
| 133 | Taltz*.ti,ab,kf. |
| 134 | Secukinumab.ti,ab,kf. |
| 135 | Cosentyx*.ti,ab,kf. |
| 136 | Bimekizumab.ti,ab,kf. |
| 137 | Bimzelx*.ti,ab,kf. |
| 138 | Brodalumab.ti,ab,kf. |
| 139 | Kyntheum*.ti,ab,kf. |
| 140 | Siliq*.ti,ab,kf. |
| 141 | Netakimab.ti,ab,kf. |
| 142 | ((Interleukin 12* or IL 12* or IL12*) adj3 (inhibitor* or antagonist* or blocker*)).ti,ab,kf. |
| 143 | Ustekinumab.ti,ab,kf. |
| 144 | Neulara*.ti,ab,kf. |
| 145 | Stelara*.ti,ab,kf. |
| 146 | Briakinumab.ti,ab,kf. |
| 147 | ((Interleukin 23* or IL 23* or IL23*) adj3 (inhibitor* or antagonist* or blocker*)).ti,ab,kf. |
| 148 | Risankizumab.ti,ab,kf. |
| 149 | Skyrizi*.ti,ab,kf. |
| 150 | Guselkumab.ti,ab,kf. |
| 151 | Tremfya*.ti,ab,kf. |
| 152 | Tildrakizumab.ti,ab,kf. |
| 153 | ilumetri*.ti,ab,kf. |
| 154 | ilumya*.ti,ab,kf. |
| 155 | (anti CD20 adj2 antibod*).ti,ab,kf. |
| 156 | Rituximab.ti,ab,kf. |
| 157 | Rituxan*.ti,ab,kf. |
| 158 | AcellBia*.ti,ab,kf. |
| 159 | Blitzima*.ti,ab,kf. |
| 160 | Halpriza*.ti,ab,kf. |
| 161 | Maball*.ti,ab,kf. |
| 162 | Mabion*.ti,ab,kf. |
| 163 | MabTas*.ti,ab,kf. |
| 164 | Novex*.ti,ab,kf. |
| 165 | Reditux*.ti,ab,kf. |
| 166 | Riabni*.ti,ab,kf. |
| 167 | Ritemvia*.ti,ab,kf. |
| 168 | Ritucad*.ti,ab,kf. |
| 169 | RituxiRel*.ti,ab,kf. |
| 170 | Rituzena*.ti,ab,kf. |
| 171 | Rixathon*.ti,ab,kf. |
| 172 | Ruxience*.ti,ab,kf. |
| 173 | Truxima*.ti,ab,kf. |
| 174 | Usmal*.ti,ab,kf. |
| 175 | Zytux*.ti,ab,kf. |
| 176 | Mabthera*.ti,ab,kf. |
| 177 | exp antigens, cd/ad, ae, ai, de, tu, to |
| 178 | immunoconjugates/ or abatacept/ |
| 179 | ((co stimulation or costimulation) adj modulator*).ti,ab,kf. |
| 180 | abatacept.ti,ab,kf. |
| 181 | Orencia*.ti,ab,kf. |
| 182 | Granulocyte-Macrophage Colony-Stimulating Factor/ad, ae, ai, de, tu, to |
| 183 | (granulocyte macrophage adj5 (inhibitor* or antagonist* or blocker*)).ti,ab,kf. |
| 184 | anti granulocyte macrophage.ti,ab,kf. |
| 185 | ((GM CSF or GMCSF) adj3 (inhibitor* or antagonist* or blocker*)).ti,ab,kf. |
| 186 | (anti adj (GM CSF or GMCSF)).ti,ab,kf. |
| 187 | namilumab.ti,ab,kf. |
| 188 | or/21-187 |
| 189 | 20 and 188 |
| 190 | exp animals/ not humans/ |
| 191 | 189 not 190 |
| 192 | limit 191 to ed=20140630-20230417 [create date: date added to pubmed] |
| 193 | limit 191 to ed=20140630-20230417 [entry date: when processing of record ends] |
| 194 | limit 191 to ez=20140630-20230417 [entrez date: date citation added to pubmed] |
| 195 | or/192-194 |
| 196 | randomized controlled trial.pt. [Cochrane Highly Sensitive Search Strategy: sensitivity- and precision-maximizing version (2008 revision) - modified] |
| 197 | controlled clinical trial.pt. |
| 198 | randomi#ed.ab. [modified to pick up 's' and 'z' variants] |
| 199 | placebo.ab. |
| 200 | clinical trials as topic.sh. |
| 201 | randomly.ab. |
| 202 | trial.ti. |
| 203 | or/196-202 |
| 204 | exp cohort studies/ or exp epidemiologic studies/ or exp clinical trial/ or exp evaluation studies as topic/ or exp statistics as topic/ [Waffenscchmidt 2020 Filter for controlled NRS - best sensitivity] |
| 205 | ((control and (group* or study)) or (time and factors) or program or survey* or ci or cohort or comparative stud* or evaluation studies or follow-up*).mp. |
| 206 | 204 or 205 |
| 207 | comment/ or editorial/ or exp review/ or meta analysis/ or consensus/ or exp guideline/ |
| 208 | hi.fs. or case report.mp. |
| 209 | 207 or 208 |
| 210 | 206 not 209 |
| 211 | 203 or 210 |
| 212 | 195 and 211 [RCT + cNRS best sensitivity filters] |

# EMBASE (OvidSP)

| 1 | exp axial spondyloarthritis/ |
| --- | --- |
| 2 | spondyloarthropathy/ |
| 3 | spondylarthritis/ |
| 4 | spondylitis/ |
| 5 | sacroiliitis/ |
| 6 | ankylosi*.ti,ab,kf. |
| 7 | spondyl?arthr*.ti,ab,kf. |
| 8 | spondylitis.ti,ab,kf. |
| 9 | sacroili?tis.ti,ab,kf. |
| 10 | spondylodiscitis.ti,ab,kf. |
| 11 | (bekhtere* or bechtere*).ti,ab,kf. |
| 12 | axSpA.ti,ab,kf. |
| 13 | nraxSpA.ti,ab,kf. |
| 14 | (axial adj3 SpA).ti,ab,kf. |
| 15 | AS.ti,ab,kf. |
| 16 | marie stru*.ti,ab,kf. |
| 17 | marie* disease*.ti,ab,kf. |
| 18 | bamboo spine.ti,ab,kf. |
| 19 | (spin* adj3 (arthrit* or arthropath*)).ti,ab,kf. |
| 20 | or/1-19 |
| 21 | biological product/ |
| 22 | biosimilar agent/ |
| 23 | antirheumatic agent/ |
| 24 | biosimilar*.ti,ab,kf. |
| 25 | (targeted adj3 (DMARD* or disease modify* or antirheumatic* or anti rheumatic*)).ti,ab,kf. |
| 26 | tsDMARD*.ti,ab,kf. |
| 27 | bDMARD*.ti,ab,kf. |
| 28 | (biologic* adj3 (DMARD* or disease modify* or antirheumatic* or anti rheumatic*)).ti,ab,kf. |
| 29 | (biologic* adj3 (drug* or medication* or medicine* or pharmaceut*)).ti,ab,kf. |
| 30 | phosphodiesterase inhibitor/ |
| 31 | exp phosphodiesterase IV inhibitor/ |
| 32 | phosphodiesterase IV/ae, ct, ad, cb, cm, it, dt, to, im, iv, po, pv, sc, tm |
| 33 | phosphodiesterase/ae, ct, ad, cb, cm, it, dt, to, im, iv, po, pv, sc, tm |
| 34 | ((phosphodiesterase 4 or phosphodiesterase IV) adj3 (inhibitor* or antagonist* or blocker*)).ti,ab,kf. |
| 35 | (PDE4i or PDE 4i or ((PDE4 or PDE 4) adj3 (inhibitor* or antagonist* or blocker*))).ti,ab,kf. |
| 36 | ((PDEiv or PDE iv) adj3 (inhibitor* or antagonist* or blocker*)).ti,ab,kf. |
| 37 | apremilast.ti,ab,kf. |
| 38 | Otelza*.ti,ab,kf. |
| 39 | Janus kinase/ae, ct, ad, cb, cm, it, dt, to, im, iv, po, pv, sc, tm |
| 40 | exp Janus kinase inhibitor/ |
| 41 | ((JAK or janus kinase or tyrosine kinase or TYK) adj3 (inhibitor* or antagonist* or blocker*)).ti,ab,kf. |
| 42 | (anti adj (JAK* or janus kinase or tyrosine kinase or TYK)).ti,ab,kf. |
| 43 | ((JAK or JAK1 or JAK2 or JAK3 or TYK2) adj3 (blocker* or inhibitor* or antagonist*)).ti,ab,kf. |
| 44 | (JAKi* or JAK1i* or JAK2i* or JAK3i* or TYK2i*).ti,ab,kf. |
| 45 | (antiJAK* or antiTYK*).ti,ab,kf. |
| 46 | tofacitinib.ti,ab,kf. |
| 47 | Xeljanz*.ti,ab,kf. |
| 48 | upadacitinib.ti,ab,kf. |
| 49 | Rinvoq*.ti,ab,kf. |
| 50 | filgotinib.ti,ab,kf. |
| 51 | Jyseleca*.ti,ab,kf. |
| 52 | baricitinib.ti,ab,kf. |
| 53 | Olumiant*.ti,ab,kf. |
| 54 | immunoglobulin enhancer binding protein/ae, ct, ad, cb, cm, it, dt, to, im, iv, po, pv, sc, tm |
| 55 | I kappa B/ [NK kappa B inhibitor] |
| 56 | ((NF kB or kappa B) adj3 (inhibitor* or antagonist* or blocker*)).ti,ab,kf. |
| 57 | Iguratimod.ti,ab,kf. |
| 58 | monokine/ae, ct, ad, cb, cm, it, dt, to, im, iv, po, pv, sc, tm |
| 59 | exp monoclonal antibody/ |
| 60 | exp tumor necrosis factor inhibitor/ |
| 61 | tumor necrosis factor receptor/ae, ct, ad, cb, cm, it, dt, to, im, iv, po, pv, sc, tm |
| 62 | ((tumo?r necrosis or TNF*) adj3 (inhibitor* or antagonist* or blocker*)).ti,ab,kf. |
| 63 | anti tumo?r necrosis.ti,ab,kf. |
| 64 | (TNF?i or anti TNF* or antiTNF*).ti,ab,kf. |
| 65 | Adalimumab.ti,ab,kf. |
| 66 | Humira*.ti,ab,kf. |
| 67 | Abrilada*.ti,ab,kf. |
| 68 | Amgevita*.ti,ab,kf. |
| 69 | Amsparity*.ti,ab,kf. |
| 70 | Cyltezo*.ti,ab,kf. |
| 71 | Hadlima*.ti,ab,kf. |
| 72 | Halimatoz*.ti,ab,kf. |
| 73 | Hefiya*.ti,ab,kf. |
| 74 | Hulio*.ti,ab,kf. |
| 75 | Hyrimoz*.ti,ab,kf. |
| 76 | Idacio*.ti,ab,kf. |
| 77 | Imraldi*.ti,ab,kf. |
| 78 | Kromeya*.ti,ab,kf. |
| 79 | Solymbic*.ti,ab,kf. |
| 80 | Trudexa*.ti,ab,kf. |
| 81 | Yuflyma*.ti,ab,kf. |
| 82 | Certolizumab.ti,ab,kf. |
| 83 | Cimzia*.ti,ab,kf. |
| 84 | Etanercept.ti,ab,kf. |
| 85 | Enbrel*.ti,ab,kf. |
| 86 | Altebrel*.ti,ab,kf. |
| 87 | Avent*.ti,ab,kf. |
| 88 | Benepali*.ti,ab,kf. |
| 89 | Brenzys*.ti,ab,kf. |
| 90 | Davictrel*.ti,ab,kf. |
| 91 | Erelzi*.ti,ab,kf. |
| 92 | Etacept*.ti,ab,kf. |
| 93 | Etanar*.ti,ab,kf. |
| 94 | Eticovo*.ti,ab,kf. |
| 95 | Eucept*.ti,ab,kf. |
| 96 | Infinitam*.ti,ab,kf. |
| 97 | Nanercept*.ti,ab,kf. |
| 98 | Nepexto*.ti,ab,kf. |
| 99 | Qiangke*.ti,ab,kf. |
| 100 | Yisaipu*.ti,ab,kf. |
| 101 | Golimumab.ti,ab,kf. |
| 102 | Simponi*.ti,ab,kf. |
| 103 | Infliximab.ti,ab,kf. |
| 104 | Remicade*.ti,ab,kf. |
| 105 | Avsola*.ti,ab,kf. |
| 106 | Baimaibo*.ti,ab,kf. |
| 107 | Flixabi*.ti,ab,kf. |
| 108 | Flammegis*.ti,ab,kf. |
| 109 | Inflectra*.ti,ab,kf. |
| 110 | Infimab*.ti,ab,kf. |
| 111 | Ixifi*.ti,ab,kf. |
| 112 | Remsima*.ti,ab,kf. |
| 113 | Renflexis*.ti,ab,kf. |
| 114 | Zessly*.ti,ab,kf. |
| 115 | exp interleukin receptor/ae, ct, ad, cb, cm, it, dt, to, im, iv, po, pv, sc, tm |
| 116 | exp interleukin 1 receptor blocking agent/ |
| 117 | recombinant interleukin 1 receptor antagonist/ |
| 118 | ((interleukin 1 or IL 1 or IL1) adj3 (inhibitor* or antagonist* or blocker*)).ti,ab,kf. |
| 119 | anakinra.ti,ab,kf. |
| 120 | kineret*.ti,ab,kf. |
| 121 | ((interleukin 6* or IL6* or IL 6*) adj3 (inhibitor* or antagonist* or blocker*)).ti,ab,kf. |
| 122 | tocilizumab.ti,ab,kf. |
| 123 | actemra*.ti,ab,kf. |
| 124 | roActemra.ti,ab,kf. |
| 125 | Kevzara*.ti,ab,kf. |
| 126 | ((Interleukin 17* or IL 17* or IL17*) adj3 (inhibitor* or antagonist* or blocker*)).ti,ab,kf. |
| 127 | Ixekizumab.ti,ab,kf. |
| 128 | Taltz*.ti,ab,kf. |
| 129 | Secukinumab.ti,ab,kf. |
| 130 | Cosentyx*.ti,ab,kf. |
| 131 | Bimekizumab.ti,ab,kf. |
| 132 | Bimzelx*.ti,ab,kf. |
| 133 | Brodalumab.ti,ab,kf. |
| 134 | Kyntheum*.ti,ab,kf. |
| 135 | Siliq*.ti,ab,kf. |
| 136 | Netakimab.ti,ab,kf. |
| 137 | ((Interleukin 12* or IL 12* or IL12*) adj3 (inhibitor* or antagonist* or blocker*)).ti,ab,kf. |
| 138 | Ustekinumab.ti,ab,kf. |
| 139 | Neulara*.ti,ab,kf. |
| 140 | Stelara*.ti,ab,kf. |
| 141 | Briakinumab.ti,ab,kf. [CHECK re inclusion for axSpA] |
| 142 | ((Interleukin 23* or IL 23* or IL23*) adj3 (inhibitor* or antagonist* or blocker*)).ti,ab,kf. |
| 143 | Risankizumab.ti,ab,kf. |
| 144 | Skyrizi*.ti,ab,kf. |
| 145 | Guselkumab.ti,ab,kf. |
| 146 | Tremfya*.ti,ab,kf. |
| 147 | Tildrakizumab.ti,ab,kf. |
| 148 | ilumetri*.ti,ab,kf. |
| 149 | ilumya*.ti,ab,kf. |
| 150 | CD20 antibody/ae, ct, ad, cb, cm, it, dt, to, im, iv, po, pv, sc, tm |
| 151 | (anti CD20 adj2 antibod*).ti,ab,kf. |
| 152 | rituximab/ |
| 153 | Rituximab.ti,ab,kf. |
| 154 | Rituxan*.ti,ab,kf. |
| 155 | AcellBia*.ti,ab,kf. |
| 156 | Blitzima*.ti,ab,kf. |
| 157 | Halpriza*.ti,ab,kf. |
| 158 | Maball*.ti,ab,kf. |
| 159 | Mabion*.ti,ab,kf. |
| 160 | MabTas*.ti,ab,kf. |
| 161 | Novex*.ti,ab,kf. |
| 162 | Reditux*.ti,ab,kf. |
| 163 | Riabni*.ti,ab,kf. |
| 164 | Ritemvia*.ti,ab,kf. |
| 165 | Ritucad*.ti,ab,kf. |
| 166 | RituxiRel*.ti,ab,kf. |
| 167 | Rituzena*.ti,ab,kf. |
| 168 | Rixathon*.ti,ab,kf. |
| 169 | Ruxience*.ti,ab,kf. |
| 170 | Truxima*.ti,ab,kf. |
| 171 | Usmal*.ti,ab,kf. |
| 172 | Zytux*.ti,ab,kf. |
| 173 | Mabthera*.ti,ab,kf. |
| 174 | exp leukocyte antigen/ae, ct, ad, cb, cm, it, dt, to, im, iv, po, pv, sc, tm |
| 175 | antibody conjugate/ae, ct, ad, cb, cm, it, dt, to, im, iv, po, pv, sc, tm |
| 176 | antibody drug conjugate/ |
| 177 | abatacept/ |
| 178 | ((co stimulation or costimulation) adj modulator*).ti,ab,kf. |
| 179 | abatacept.ti,ab,kf. |
| 180 | Orencia*.ti,ab,kf. |
| 181 | granulocyte macrophage colony stimulating factor/ae, ct, ad, cb, cm, it, dt, to, im, iv, po, pv, sc, tm |
| 182 | (granulocyte macrophage adj5 (inhibitor* or antagonist* or blocker*)).ti,ab,kf. |
| 183 | anti granulocyte macrophage.ti,ab,kf. |
| 184 | ((GM CSF or GMCSF) adj3 (inhibitor* or antagonist* or blocker*)).ti,ab,kf. |
| 185 | (anti adj (GM CSF or GMCSF)).ti,ab,kf. |
| 186 | namilumab.ti,ab,kf. |
| 187 | or/21-186 |
| 188 | randomized controlled trial/ [Cochrane Highly Sensitive Search Strategy for identifying controlled trials] |
| 189 | controlled clinical trial/ |
| 190 | random*.ti,ab. |
| 191 | randomization/ |
| 192 | intermethod comparison/ |
| 193 | placebo.ti,ab. |
| 194 | (compare or compared or comparison).ti. |
| 195 | ((evaluated or evaluate or evaluating or assessed or assess) and (compare or compared or comparing or comparison)).ab. |
| 196 | open label.ti,ab. |
| 197 | ((double or single or doubly or singly) adj (blind or blindedor blindly)).ti,ab. |
| 198 | double blind procedure/ |
| 199 | parallel group*1.ti,ab. |
| 200 | (crossover or cross over).ti,ab. |
| 201 | ((assign* or match or matched or allocation) adj5 (alternate or group*1 or intervention*1 or patient*1 or subject*1 or participant*1)).ti,ab. |
| 202 | (assigned or allocated).ti,ab. |
| 203 | (controlled adj7 (study or design or trial)).ti,ab. |
| 204 | (volunteer or volunteers).ti,ab. |
| 205 | human experiment/ |
| 206 | trial.ti. |
| 207 | or/188-206 |
| 208 | (random* adj sampl* adj7 (cross section* or questionnaire*1 or survey* or database*1)).ti,ab. not (comparative study/ or controlled study/ or randomi?ed controlled.ti,ab. or randomly assigned.ti,ab.) |
| 209 | Cross-sectional study/ not (randomized controlled trial/ or controlled clinical study/ or controlled study/ or randomi?ed controlled.ti,ab. or control group*1.ti,ab.) |
| 210 | (((case adj control*) and random*) not randomi?ed controlled).ti,ab. |
| 211 | (Systematic review not (trial or study)).ti. |
| 212 | (nonrandom$ not random$).ti,ab. |
| 213 | random field*.ti,ab. |
| 214 | (random cluster adj3 sampl*).ti,ab. |
| 215 | (review.ab. and review.pt.) not trial.ti. |
| 216 | we searched.ab. and (review.ti. or review.pt.) |
| 217 | (databases adj4 searched).ab. |
| 218 | (rat or rats or mouse or mice or swine or porcine or murine or sheep or lambs or pigs or piglets or rabbit or rabbits or cat or cats or dog or dogs or cattle or bovine or monkey or monkeys or trout or marmoset*1).ti. and animal experiment/ |
| 219 | Animal experiment/ not (human experiment/ or human/) |
| 220 | or/208-219 |
| 221 | 207 not 220 |
| 222 | cohort analysis/ or exp epidemiology/ or exp clinical trial/ or exp evaluation study/ or exp statistics/ [based on Waffenscchmidt 2020 MEDLINE filter for controlled NRS - best sensitivity] |
| 223 | ((control and (group* or study)) or (time and factors) or program or survey* or ci or cohort or comparative stud* or evaluation studies or follow-up*).mp. |
| 224 | 222 or 223 |
| 225 | editorial/ or exp "review"/ or exp meta analysis/ or consensus/ or exp practice guideline/ or case report.mp. |
| 226 | exp animal/ not human/ |
| 227 | 225 or 226 |
| 228 | 224 not 227 |
| 229 | 221 or 228 |
| 230 | 20 and 187 and 229 |
| 231 | limit 230 to embase |
| 232 | limit 231 to dc=20140630-20230417 [date created - date of last activity on citation before delivered to ovid i.e. original file created] |
| 233 | limit 231 to dd=20140630-20230417 [date created for delivery state ="new" original info delivered to ovid] |
| 234 | 232 or 233 |

# Cochrane CENTRAL

| #1 | MeSH descriptor: [Axial Spondyloarthritis] explode all trees |
| --- | --- |
| #2 | MeSH descriptor: [Spondylarthropathies] this term only |
| #3 | MeSH descriptor: [Spondylarthritis] this term only |
| #4 | MeSH descriptor: [Spondylitis] this term only |
| #5 | MeSH descriptor: [Sacroiliitis] this term only |
| #6 | ankylos*:ti,ab,kw |
| #7 | spondylarthr* OR spondyloarthr*:ti,ab,kw |
| #8 | spondylitis:ti,ab,kw |
| #9 | sacroiliitis:ti,ab,kw |
| #10 | spondylodiscitis:ti,ab,kw |
| #11 | bekhtere* or bechtere*:ti,ab,kw |
| #12 | axSpA:ti,ab,kw |
| #13 | nraxSpA:ti,ab,kw |
| #14 | SpA:ti,ab,kw |
| #15 | marie NEXT stru*:ti,ab,kw |
| #16 | marie NEXT disease*:ti,ab,kw |
| #17 | "bamboo spine":ti,ab,kw |
| #18 | spin* NEAR/3 (arthrit* OR arthropath*):ti,ab,kw |
| #19 | {OR #1-#18} |
| #20 | MeSH descriptor: [Biological Products] this term only |
| #21 | MeSH descriptor: [Biosimilar Pharmaceuticals] this term only |
| #22 | MeSH descriptor: [Antirheumatic Agents] this term only |
| #23 | biosimilar*:ti,ab,kw |
| #24 | targeted NEAR/3 (DMARD* or (disease NEXT modify*) or antirheumatic* or (anti NEXT rheumatic*)):ti,ab,kw |
| #25 | tsDMARD*:ti,ab,kw |
| #26 | targeted NEXT synthetic*:ti,ab,kw |
| #27 | bDMARD*:ti,ab,kw |
| #28 | biologic* NEAR/3 (DMARD* or (disease NEXT modify*) or antirheumatic* or (anti NEXT rheumatic*)):ti,ab,kw |
| #29 | biologic* NEAR/3 (drug* or medication* or medicine* or pharmaceut*):ti,ab,kw |
| #30 | MeSH descriptor: [Phosphodiesterase Inhibitors] this term only |
| #31 | MeSH descriptor: [Phosphodiesterase 4 Inhibitors] this term only |
| #32 | ("phosphodiesterase 4" or "phosphodiesterase IV") NEAR/3 (inhibitor* or antagonist* or blocker*):ti,ab,kw |
| #33 | PDE4i or PDE 4i or ((PDE4 or PDE 4) NEAR/3 (inhibitor* or antagonist* or blocker*)):ti,ab,kw |
| #34 | (PDEiv or "PDE iv") NEAR/3 (inhibitor* or antagonist* or blocker*):ti,ab,kw |
| #35 | apremilast:ti,ab,kw |
| #36 | Otelza*:ti,ab,kw |
| #37 | MeSH descriptor: [Janus Kinases] explode all trees |
| #38 | MeSH descriptor: [Janus Kinase Inhibitors] this term only |
| #39 | (JAK or "janus kinase" or "tyrosine kinase" or TYK) NEAR/3 (inhibitor* or antagonist* or blocker*):ti,ab,kw |
| #40 | anti NEXT (JAK* or "janus kinase" or "tyrosine kinase" or TYK):ti,ab,kw |
| #41 | ((JAK or JAK1 or JAK2 or JAK3 or TYK2) NEAR/3 (blocker* or inhibitor* or antagonist*)):ti,ab,kw |
| #42 | (JAKi* or JAK1i* or JAK2i* or JAK3i* or TYK2i*):ti,ab,kw |
| #43 | (antiJAK* or antiTYK*):ti,ab,kw |
| #44 | tofacitinib:ti,ab,kw |
| #45 | Xeljanz*:ti,ab,kw |
| #46 | upadacitinib:ti,ab,kw |
| #47 | Rinvoq*:ti,ab,kw |
| #48 | filgotinib:ti,ab,kw |
| #49 | Jyseleca*:ti,ab,kw |
| #50 | baricitinib:ti,ab,kw |
| #51 | Olumiant*:ti,ab,kw |
| #52 | MeSH descriptor: [NF-kappa B] explode all trees |
| #53 | (("NF kB" or "kappa B") adj3 (inhibitor* or antagonist* or blocker*)):ti,ab,kw |
| #54 | (anti NEXT ("NF kB" or "kappa B")):ti,ab,kw |
| #55 | Iguratimod:ti,ab,kw |
| #56 | MeSH descriptor: [Monokines] explode all trees |
| #57 | MeSH descriptor: [Antibodies, Monoclonal] explode all trees |
| #58 | MeSH descriptor: [Tumor Necrosis Factor Inhibitors] this term only |
| #59 | MeSH descriptor: [Tumor Necrosis Factor-alpha] this term only |
| #60 | MeSH descriptor: [Receptors, Tumor Necrosis Factor] explode all trees |
| #61 | (("tumor necrosis" or "tumour necrosis" or TNF*) NEAR/3 (inhibitor* or antagonist* or blocker*)):ti,ab,kw |
| #62 | ("anti tumor necrosis" or "anti tumour necrosis"):ti,ab,kw |
| #63 | (anti NEXT TNF*) or antiTNF* or TNFi*:ti,ab,kw |
| #64 | MeSH descriptor: [Adalimumab] this term only |
| #65 | Humira*:ti,ab,kw |
| #66 | Abrilada*:ti,ab,kw |
| #67 | Amgevita*:ti,ab,kw |
| #68 | Amsparity*:ti,ab,kw |
| #69 | Cyltezo*:ti,ab,kw |
| #70 | Hadlima*:ti,ab,kw |
| #71 | Halimatoz*:ti,ab,kw |
| #72 | Hefiya*:ti,ab,kw |
| #73 | Hulio*:ti,ab,kw |
| #74 | Hyrimoz*:ti,ab,kw |
| #75 | Idacio*:ti,ab,kw |
| #76 | Imraldi*:ti,ab,kw |
| #77 | Kromeya*:ti,ab,kw |
| #78 | Solymbic*:ti,ab,kw |
| #79 | Trudexa*:ti,ab,kw |
| #80 | Yuflyma*:ti,ab,kw |
| #81 | MeSH descriptor: [Certolizumab Pegol] this term only |
| #82 | Certolizumab:ti,ab,kw |
| #83 | Cimzia*:ti,ab,kw |
| #84 | MeSH descriptor: [Etanercept] this term only |
| #85 | Etanercept:ti,ab,kw |
| #86 | Enbrel*:ti,ab,kw |
| #87 | Altebrel*:ti,ab,kw |
| #88 | Avent*:ti,ab,kw |
| #89 | Benepali*:ti,ab,kw |
| #90 | Brenzys*:ti,ab,kw |
| #91 | Davictrel*:ti,ab,kw |
| #92 | Erelzi*:ti,ab,kw |
| #93 | Etacept*:ti,ab,kw |
| #94 | Etanar*:ti,ab,kw |
| #95 | Eticovo*:ti,ab,kw |
| #96 | Eucept*:ti,ab,kw |
| #97 | Infinitam*:ti,ab,kw |
| #98 | Nanercept*:ti,ab,kw |
| #99 | Nepexto*:ti,ab,kw |
| #100 | Qiangke*:ti,ab,kw |
| #101 | Yisaipu*:ti,ab,kw |
| #102 | Golimumab:ti,ab,kw |
| #103 | Simponi*:ti,ab,kw |
| #104 | MeSH descriptor: [Infliximab] this term only |
| #105 | Infliximab:ti,ab,kw |
| #106 | Remicade*:ti,ab,kw |
| #107 | Avsola*:ti,ab,kw |
| #108 | Baimaibo*:ti,ab,kw |
| #109 | Flixabi*:ti,ab,kw |
| #110 | Flammegis*:ti,ab,kw |
| #111 | Inflectra*:ti,ab,kw |
| #112 | Infimab*:ti,ab,kw |
| #113 | Ixifi*:ti,ab,kw |
| #114 | Remsima*:ti,ab,kw |
| #115 | Renflexis*:ti,ab,kw |
| #116 | Zessly*:ti,ab,kw |
| #117 | MeSH descriptor: [Interleukins] explode all trees |
| #118 | MeSH descriptor: [Receptors, Interleukin] explode all trees |
| #119 | MeSH descriptor: [Interleukin 1 Receptor Antagonist Protein] this term only |
| #120 | (("interleukin 1" or "IL 1" or IL1) NEAR/3 (inhibitor* or antagonist* or blocker*)):ti,ab,kw |
| #121 | anakinra:ti,ab,kw |
| #122 | kineret*:ti,ab,kw |
| #123 | (((interleukin NEXT 6*) or IL6* or (IL NEXT 6*)) NEAR/3 (inhibitor* or antagonist* or blocker*)):ti,ab,kw |
| #124 | tocilizumab:ti,ab,kw |
| #125 | actemra*:ti,ab,kw |
| #126 | roActemra:ti,ab,kw |
| #127 | Sarilumab:ti,ab,kw |
| #128 | Kevzara*:ti,ab,kw |
| #129 | (((interleukin NEXT 17*) or IL17* or (IL NEXT 17*)) NEAR/3 (inhibitor* or antagonist* or blocker*)):ti,ab,kw |
| #130 | Ixekizumab:ti,ab,kw |
| #131 | Taltz*:ti,ab,kw |
| #132 | Secukinumab:ti,ab,kw |
| #133 | Cosentyx*:ti,ab,kw |
| #134 | Bimekizumab:ti,ab,kw |
| #135 | Bimzelx*:ti,ab,kw |
| #136 | Brodalumab:ti,ab,kw |
| #137 | Kyntheum*:ti,ab,kw |
| #138 | Siliq*:ti,ab,kw |
| #139 | Netakimab:ti,ab,kw |
| #140 | (((interleukin NEXT 12*) or IL12* or (IL NEXT 12*)) NEAR/3 (inhibitor* or antagonist* or blocker*)):ti,ab,kw |
| #141 | Ustekinumab:ti,ab,kw |
| #142 | Neulara*:ti,ab,kw |
| #143 | Stelara*:ti,ab,kw |
| #144 | Briakinumab:ti,ab,kw |
| #145 | (((interleukin NEXT 23*) or IL23* or (IL NEXT 23*)) NEAR/3 (inhibitor* or antagonist* or blocker*)):ti,ab,kw |
| #146 | Risankizumab:ti,ab,kw |
| #147 | Skyrizi*:ti,ab,kw |
| #148 | Guselkumab:ti,ab,kw |
| #149 | Tremfya*:ti,ab,kw |
| #150 | Tildrakizumab:ti,ab,kw |
| #151 | ilumetri*:ti,ab,kw |
| #152 | ilumya*:ti,ab,kw |
| #153 | ("anti CD20" NEAR/2 antibod*):ti,ab,kw |
| #154 | Rituximab:ti,ab,kw |
| #155 | Rituxan*:ti,ab,kw |
| #156 | AcellBia*:ti,ab,kw |
| #157 | Blitzima*:ti,ab,kw |
| #158 | Halpriza*:ti,ab,kw |
| #159 | Maball*:ti,ab,kw |
| #160 | Mabion*:ti,ab,kw |
| #161 | MabTas*:ti,ab,kw |
| #162 | Novex*:ti,ab,kw |
| #163 | Reditux*:ti,ab,kw |
| #164 | Riabni*:ti,ab,kw |
| #165 | Ritemvia*:ti,ab,kw |
| #166 | Ritucad*:ti,ab,kw |
| #167 | RituxiRel*:ti,ab,kw |
| #168 | Rituzena*:ti,ab,kw |
| #169 | Rixathon*:ti,ab,kw |
| #170 | Ruxience*:ti,ab,kw |
| #171 | Truxima*:ti,ab,kw |
| #172 | Usmal*:ti,ab,kw |
| #173 | Zytux*:ti,ab,kw |
| #174 | Mabthera*:ti,ab,kw |
| #175 | MeSH descriptor: [Antigens, CD] explode all trees |
| #176 | MeSH descriptor: [Immunoconjugates] this term only |
| #177 | MeSH descriptor: [Abatacept] this term only |
| #178 | (("co stimulation" or costimulation) NEXT modulator*):ti,ab,kw |
| #179 | abatacept:ti,ab,kw |
| #180 | Orencia*:ti,ab,kw |
| #181 | MeSH descriptor: [Granulocyte-Macrophage Colony-Stimulating Factor] this term only |
| #182 | ("granulocyte macrophage" NEAR/5 (inhibitor* or antagonist* or blocker*)):ti,ab,kw |
| #183 | "anti granulocyte macrophage":ti,ab,kw |
| #184 | (("GM CSF" or GMCSF) NEAR/3 (inhibitor* or antagonist* or blocker*)):ti,ab,kw |
| #185 | (anti NEXT ("GM CSF" or GMCSF)):ti,ab,kw |
| #186 | namilumab:ti,ab,kw |
| #187 | {OR #20-#186} |
| #188 | #19 AND #187 |
| #189 | #19 AND #187 with Cochrane Library publication date Between Jun 2014 and Mar 2023 |
