# Supplementary material for: The 2025 British Society for Rheumatology guideline for the treatment of axial spondyloarthritis with biologic and targeted synthetic DMARDs
Source: Rheumatology (Oxford). 2025 Apr 9;64(6):3242–54. doi: 10.1093/rheumatology/keaf089 (PMC12107049; doi:10.1093/rheumatology/keaf089)
Supplement: keaf089_Supplementary_Data [file keaf089_supplementary_data.zip › keaf089_Supplementary_Data/rhe-24-2017-File004.docx]

**The 2024 BSR guideline for the treatment of axial spondyloarthritis**

**with biologic and targeted synthetic DMARDs**

**Supplementary Data S3. Selection Criteria – clinical trials**

|  | Inclusion criteria – research studies should have the following characteristics: | Exclusion criteria |
| --- | --- | --- |
| Study design: | - Primary research i.e.:   - Randomised controlled trials (RCTs) of any design (individual or cluster randomisation, step-wedge design). **[Efficacy/effectiveness/safety]**   - Controlled clinical trials (CCTs) **[Efficacy/Effectiveness/safety]**   - Secondary, post-hoc, and sub-group analyses of individual RCTs & CCTs **[Efficacy/Effectiveness/safety]**   - Large observational (‘real world’) cohort studies using electronic health records or register data and including a control group not receiving the intervention of interest **[Effectiveness/safety]**   - cohort studies where there is a comparator group or a population-based standardised incidence rate is reported, and include ≥50 participants per group **[Safety; Efficacy/Effectiveness re EMMs only].**   - Open label/long-term extension studies of RCTs (OLE/LTEs) **[Safety; Efficacy/Effectiveness re EMMs only]** | - Secondary evidence i.e.   - Systematic reviews (SRs)^1^   - Meta-analyses (not associated with SRs), umbrella reviews (*i.e.* overviews of systematic reviews), ‘narrative’ reviews   - Guidelines/Recommendations   - evidence-based synopses - Pooled analysis of RCTs^2^ & CCTs (not associated with SRs) - Case-control studies - Case series - Case studies - Qualitative studies - Editorials, commentaries, trial protocols, letters, etc. - Conference abstracts - Trials registry records - Study protocol   ^1^ defined as a review which carried out a systematic search of ≥1 electronic database, included critical appraisal and synthesised results. **If** all other selection criteria are met, please **TAG** as **‘Relevant SR’** [bibliography to be checked]  ^2^ please **TAG** as ‘**Pooled analysis of RCTs** ' [bibliography to be checked] |
| Participants and conditions of interest | - Adults with axSpA, including ankylosing spondylitis (or radiographic axSpA) and non-radiographic axSpA. | - Axial manifestations of psoriatic arthritis - Enthesitis-related Juvenile Idiopathic Arthritis - Spondylitis-related Juvenile Idiopathic Arthritis - Other rheumatic and musculoskeletal conditions. |
| Interventions or exposures | - Pharmacological treatment of people with axial spondyloarthritis using b/tsDMARDs including biosimilars, including TNFi, IL-17i, JAKi, in any formulation and duration. - Treatment strategies including switching, tapering, withdrawal and treat-to-target approaches. | - Treatment of enthesitis- or spondylitis-related Juvenile Idiopathic Arthritis and axial disease in psoriatic arthritis. - NSAIDs, glucocorticoids and conventional synthetic DMARDs. - Non-pharmacological management (a brief summary from related guidelines will be included). |
| Comparisons or control groups | - the same b/tsDMARD in a different dose/regimen - or, another b/tsDMARD, - or, any non-b/tsDMARD drug treatment, - or, combination of b/tsDMARD with non-b/tsDMARD treatment - or, placebo.   OR   - no control group for safety outcomes only, if population-based incidence rates are reported. | n/a |
| Outcomes of interest | **Efficacy/Effectiveness**:   - ASAS response criteria (ASAS20, ASAS40, ASAS5/6 and ASAS partial remission) - Disease activity   - Ankylosing Spondylitis Disease Activity Score [ASDAS]: absolute change, response criteria (clinically important improvement (Δ≥1.1), major improvement (Δ≥2.0)), states (inactive disease (<1.3), low disease activity (<2.1));   - Bath Ankylosing Spondylitis Disease Activity Index (BASDAI): absolute change, response (≥50% improvement);   - patient global assessment of disease activity   - day/night spinal pain - Spinal mobility   - Bath Ankylosing Spondylitis Metrology Index (BASMI);   - individual spinal mobility measures - Physical function (Bath Ankylosing Spondylitis Functional Index; BASFI), - Peripheral manifestations (enthesitis scores; swollen/tender joint count), - functioning and health (ASAS Health Index (ASAS HI)), - radiographic damage (modified Stoke AS Spine Score); - inflammation on MRI (active sacroiliitis according to ASAS/Outcome Measures in Rheumatology (OMERACT) definition; - Spondyloarthritis Research Consortium of Canada (SPARCC) for sacroiliac joints and spine, work disability and productivity. - Severity of Extra-musculoskeletal manifestations (EMMs), (psoriasis, acute anterior uveitis (AAU), inflammatory bowel disease) [also included under safety]   **Safety:**   - serious adverse events (AEs) *i.e.* those that result in death, require either inpatient hospitalization or the prolongation of hospitalization, are life-threatening, result in a persistent or significant disability/incapacity or result in a congenital anomaly/birth defect - withdrawals due to AEs, including but not limited to, deaths, infections, malignancies, congestive heart failure, cardiovascular disease, infusion/injection-site reactions, lipid levels, renal function, hepatic effects, haematological abnormalities, gastrointestinal effects, demyelinating disease. - Extra-musculoskeletal manifestations (EMMs) *i.e.*  incidence of:   - psoriasis,   - acute anterior uveitis (AAU),   - inflammatory bowel disease (IBD), [also included as efficacy/effectiveness outcomes] |  |
| Setting | - Secondary/tertiary care rheumatology (targeted therapies are restricted to specialist use). |  |
| Other | - English language papers, or non-English language papers with an English abstract | - Non-English language papers if there is no English-language abstract |

**Key issues and Questions**

| **Treatment strategy:** | | |
| --- | --- | --- |
|  | **Q1** | • In adults with active axSpA, what is the clinical effectiveness and safety of targeted therapies, compared to each other or placebo, on   - - Axial symptoms;   - Peripheral musculoskeletal manifestations, namely, arthritis, dactylitis and enthesitis;   - Extra-musculoskeletal manifestations, namely, acute anterior uveitis, psoriasis and inflammatory bowel disease;   - Comorbidities and risk factors (including the impact of comorbidities or risk factors on choice of targeted therapy and effect of therapy on common comorbidities)? |
|  | **Q2** | - In adults with active axSpA who do not respond adequately to or tolerate one or more targeted therapies, what is the clinical effectiveness and safety of switching   - to biosimilars,   - to targeted therapies with different mechanisms of action,   - after multiple targeted therapies. |
|  | **Q3** | • In adults with active axSpA, what is the clinical effectiveness and safety of combining targeted therapies (including those licensed for extra-musculoskeletal manifestations)? |
|  | **Q4** | • In adults with active axSpA, what is the evidence for a treat-to-target strategy compared to usual care? |
|  | **Q5** | • In adults with axSpA who have achieved clinical remission or low disease activity, what is the evidence, compared to usual care, for   - - tapering or dose reduction of targeted therapies,   - withdrawing targeted therapies,   - switching to biosimilars? |

**Selection Criteria – Real World Evidence (observational) studies**

**Note:** Considering the higher risk of bias and confounding in observational cohort studies compared to RCTs, evidence from ‘real-world’ studies will be downgraded for non-optimal design, and additional concerns related to study limitations (risk of bias, confounding) will be assessed as part of the GRADE approach.

|  | Inclusion criteria – research studies should have the following characteristics: | Exclusion criteria |
| --- | --- | --- |
| Study design: | - Primary research i.e.:   - Representative observational (‘real world’) cohort studies **including a control group** not receiving the intervention of interest   - Prospective, bespoke cohort design or retrospective analysis of data from an existing cohort | - Single centre cohorts reporting results from a single site (e.g. rheumatology department) - Clinical cohorts without a control group - Editorials, commentaries, letters, etc. - Conference abstracts - Study protocols |
| Participants and conditions of interest | - Adults with axSpA, including ankylosing spondylitis (or radiographic axSpA) and non-radiographic axSpA - For mixed samples of people with inflammatory arthritis, outcome data need to be presented separately for people with AxSpA - **Representative study sample**, likely to reflect target population of people with AxSpA, e.g. samples from population based studies, primary care records, disease registries, multiple sites | - Mixed populations including various types of inflammatory arthritis - Small or non-representative cohorts, selected from single sites, or using eligibility criteria that reduces representativeness - Axial manifestations of psoriatic arthritis - Enthesitis-related Juvenile Idiopathic Arthritis - Spondylitis-related Juvenile Idiopathic Arthritis - Other rheumatic and musculoskeletal conditions |
| Interventions or exposures | - Pharmacological treatment of people with axial spondyloarthritis using b/tsDMARDs including biosimilars, including TNFi, IL-17i, JAKi, in any formulation and duration. - Treatment strategies including switching, tapering, withdrawal and treat-to-target approaches. | - Treatment of enthesitis- or spondylitis-related Juvenile Idiopathic Arthritis and axial disease in psoriatic arthritis. - NSAIDs, glucocorticoids and conventional synthetic DMARDs. - Non-pharmacological management |
| Comparisons or control groups | - Head-to-head comparison with - the same b/tsDMARD in a different dose/regimen - another b/tsDMARD - any non-b/tsDMARD drug treatment - Comparison with non-pharmacological management - Comparison with standard/usual care only | n/a |
| Outcomes of interest | **Safety,** *i.e.* incidence of:   - - any AE, any SAE   - infections – any, or specifically: TB, candidiasis, herpes (shingles)   - CVD: VTE (PE/DVT), MACE (major adverse cardiovascular events – typically fatal and non-fatal MI/stroke   - events of special interest: cancer, MS   **Extra-musculoskeletal manifestations (EMMs)** *i.e.*  prevalence / incidence of:   - - psoriasis   - uveitis   - inflammatory bowel disease (IBD) | Effectiveness outcomes will not be extracted, so studies only reporting effectiveness data will be excluded. |
| Other | - English language papers, or non-English language papers with an English abstract | - Non-English language papers if there is no English-language abstract |
