# Supplementary material for: The 2025 British Society for Rheumatology guideline for the treatment of axial spondyloarthritis with biologic and targeted synthetic DMARDs
Source: Rheumatology (Oxford). 2025 Apr 9;64(6):3242–54. doi: 10.1093/rheumatology/keaf089 (PMC12107049; doi:10.1093/rheumatology/keaf089)
Supplement: keaf089_Supplementary_Data [file keaf089_supplementary_data.zip › keaf089_Supplementary_Data/rhe-24-2017-File005.docx]

**The 2024 BSR guideline for the treatment of axial spondyloarthritis**

**with biologic and targeted synthetic DMARDs**

**Supplementary Data S4. PRISMA flow diagram**

Records identified from Databases

Medline – 2072

EMBASE – 2861

Cochrane Library – 1667

Total (n = 6600)

Records removed *before screening*:

Duplicate records removed (n = 1804)

1. Records screened (Title)

(n = 4817)

1. Records screened (Abstracts)

(n= 1057)

Records excluded (n = 728)

Reports sought for retrieval

(n =329)

Reports not retrieved (n = 1)

Reports assessed for eligibility

(n =328)

Reports excluded (n=205):

Included in previous BSR guideline (n=36)

Methodological issues (n = 5)

Mixed population, no subgroup analysis by disease type (n=14)

No comparator group (n = 6)

Wrong study design (n = 48)

**Outside review’s scope (n= 96)

New studies included in review

(n =66)

Reports of new included studies

(n = 123)

**Identification of new studies via databases**

**Identification**

**Screening**

**Included**

*Reports identified from relevant systematic review (n = 21)

*Webers C, Ortolan A, Sepriano A, Falzon L, Baraliakos X, Landewé RBM, Ramiro S, van der Heijde D, Nikiphorou E. Efficacy and safety of biological DMARDs: a systematic literature review informing the 2022 update of the ASAS-EULAR recommendations for the management of axial spondyloarthritis. Ann Rheum Dis. 2023 Jan;82(1):130-141. doi: 10.1136/ard-2022-223298. Epub 2022 Oct 21. PMID: 36270657.

**Outside review’s scope: reasons for exclusion included - non English reports, primary study, protocol, wrong population group.

*From:*  Page MJ, McKenzie JE, Bossuyt PM, Boutron I, Hoffmann TC, Mulrow CD, et al. The PRISMA 2020 statement: an updated guideline for reporting systematic reviews. BMJ 2021;372:n71. doi: 10.1136/bmj.n71
