# Supplementary material for: The 2025 British Society for Rheumatology guideline for the treatment of axial spondyloarthritis with biologic and targeted synthetic DMARDs
Source: Rheumatology (Oxford). 2025 Apr 9;64(6):3242–54. doi: 10.1093/rheumatology/keaf089 (PMC12107049; doi:10.1093/rheumatology/keaf089)
Supplement: keaf089_Supplementary_Data [file keaf089_supplementary_data.zip › keaf089_Supplementary_Data/rhe-24-2017-File006.docx]

**The 2024 BSR guideline for the treatment of axial spondyloarthritis with biologic and targeted synthetic DMARDs**

**Supplementary Data S5. Recommendations voting results**

|  | OAPs | | | Recommendations | | | | | | | | | | | | | | |
| --- | --- | --- | --- | --- | --- | --- | --- | --- | --- | --- | --- | --- | --- | --- | --- | --- | --- | --- |
|  | 1 | 2 | 3 | i | ii | iii | iv | v | vi | vii | viii | ix | x | xi | xii | xiii | xiv | xv |
| Anonymised votes from 18 guideline working group members | 100 | 100 | 100 | 100 | 100 | 100 | 100 | 100 | 100 | 100 | 100 | 100 | 100 | 100 | 100 | 100 | 100 | 100 |
|  | 100 | 100 | 100 | 100 | 100 | 100 | 100 | 100 | 100 | 100 | 100 | 100 | 100 | 100 | 100 | 100 | 100 | 100 |
|  | 100 | 100 | 100 | 80 | 80 | 90 | abs | 100 | abs | abs | abs | abs | abs | abs | abs | 100 | 95 | abs |
|  | 100 | 100 | 80 | 100 | 100 | 100 | 100 | 100 | 100 | 100 | 100 | abs | 100 | 100 | 100 | 100 | abs | abs |
|  | 100 | 100 | 100 | 100 | 100 | 100 | 100 | 100 | 100 | 100 | 95 | 95 | 95 | 100 | 100 | 100 | 100 | 100 |
|  | 100 | 100 | 100 | 100 | 100 | 100 | 100 | 100 | 100 | 100 | 95 | 95 | 100 | 100 | 100 | 100 | 100 | 100 |
|  | 100 | 100 | 100 | 100 | 100 | 100 | 100 | 100 | 100 | 100 | 100 | 100 | 100 | 100 | 100 | 100 | 100 | 100 |
|  | 100 | 95 | 100 | 100 | 95 | 90 | 100 | 100 | 95 | 95 | 95 | 95 | 90 | 100 | 90 | 95 | 100 | 100 |
|  | 100 | 100 | 100 | 100 | 100 | abs | abs | 100 | abs | abs | 100 | 100 | 100 | 100 | abs | 100 | 90 | 100 |
|  | 100 | 100 | 100 | 98 | 95 | 95 | 98 | 100 | 95 | 95 | 95 | 95 | 90 | 100 | 98 | 100 | 90 | 99 |
|  | 100 | 100 | 100 | 100 | 90 | 90 | 100 | 100 | 100 | 90 | 90 | 90 | 90 | 100 | 90 | 95 | 95 | 100 |
|  | 100 | 100 | 100 | 100 | 98 | 100 | 100 | 90 | 90 | 90 | 100 | 100 | 90 | 90 | 90 | 100 | 100 | 100 |
|  | 100 | 100 | 100 | 90 | 100 | 100 | 100 | 100 | 100 | 90 | 100 | 100 | 100 | 100 | 90 | 100 | 100 | 100 |
|  | 100 | 100 | 100 | 100 | 100 | 100 | 100 | 100 | 100 | 100 | 100 | 100 | 100 | 100 | 100 | 100 | 100 | 90 |
|  | 100 | 100 | 100 | abs | 100 | 100 | 100 | 100 | abs | abs | 100 | abs | 100 | abs | abs | 100 | 100 | abs |
|  | 100 | 100 | 100 | 100 | 100 | 95 | 100 | 100 | 100 | 100 | 100 | 100 | 100 | 100 | 100 | 100 | 100 | 100 |
|  | 90 | 88 | 95 | 95 | 90 | 95 | 95 | 95 | 95 | 90 | 95 | 85 | 90 | 90 | 95 | 90 | 95 | 90 |
|  | 100 | 100 | 90 | 90 | 100 | 100 | 100 | 100 | 100 | 100 | 90 | 90 | 100 | 100 | 100 | 100 | 100 | abs |
| **Mean** | **99** | **99** | **98** | **97** | **97** | **97** | **100** | **99** | **98** | **97** | **97** | **96** | **97** | **99** | **97** | **99** | **98** | **99** |
| **Minimum** | **90** | **88** | **80** | **80** | **80** | **90** | **95** | **90** | **90** | **90** | **90** | **85** | **90** | **90** | **90** | **90** | **90** | **90** |
| OAP, overarching principles; abs, abstain. | | | | | | | | | | | | | | | | | | |
