# Supplementary material for: The 2025 British Society for Rheumatology guideline for the treatment of axial spondyloarthritis with biologic and targeted synthetic DMARDs
Source: Rheumatology (Oxford). 2025 Apr 9;64(6):3242–54. doi: 10.1093/rheumatology/keaf089 (PMC12107049; doi:10.1093/rheumatology/keaf089)
Supplement: keaf089_Supplementary_Data [file keaf089_supplementary_data.zip › keaf089_Supplementary_Data/rhe-24-2017-File007.docx]

**The 2024 BSR guideline for the treatment of axial spondyloarthritis with biologic and targeted synthetic DMARDs**

**Supplementary Data S6. Audit tool**

|  | **Yes** | **No** | **N/A** | **Comments** |
| --- | --- | --- | --- | --- |
| Has there been appropriate use of non-pharmacological management options^a^ before initiating targeted therapies? |  |  |  |  |
| Have non-pharmacological management options^a^ been discussed and offered alongside targeted therapies? |  |  |  |  |
| Has the risk of radiographic progression^b^ been assessed and discussed with the person with axSpA, including smoking cessation where applicable? |  |  |  |  |
| Has the diagnosis^c^ of axSpA been made or verified by a consultant rheumatologist? |  |  |  |  |
| Has the desired therapeutic target^d^ been discussed and agreed upon with the person with axSpA? |  |  |  |  |
| Has BASDAI, spinal pain and ASDAS^e^ been recorded at baseline and at each follow-up assessment? |  |  |  |  |
| In the absence of response to targeted therapies, has the diagnosis and/or extent of inflammatory disease activity been reviewed, ideally with supporting imaging evidence^f^? |  |  |  |  |
| Has the history, including severity and frequency, of uveitis been reviewed and documented prior to initiating/switching targeted therapies? |  |  |  |  |
| If new uveitis develops despite axSpA being well-controlled, has the decisions to change treatment been discussed with an ophthalmologist? |  |  |  |  |
| Has the extent of psoriasis involvement been assessed and documented (e.g., percentage of body surface area^g^)? |  |  |  |  |
| Has there been review and documentation of gastrointestinal symptoms suggestive of IBD before initiating/switching targeted therapies? |  |  |  |  |
| Has the history, including activity and severity, of IBD been reviewed and documented before initiating/switching targeted therapies? |  |  |  |  |
| Have comorbidities been reviewed and documented prior to starting/switching targeted therapy? |  |  |  |  |
| For individuals in sustained remission, has tapering of targeted therapy been discussed^h^? |  |  |  |  |

1. Non-pharmacological options include but are not limited to physiotherapy, hydrotherapy, and supported self-management.
2. Factors associated with higher risk of radiographic progression include but are not limited to male sex, cigarette smoking, obesity, elevated CRP/disease activity, baseline syndesmophytes, bone marrow oedema +/- fatty lesions on MRI
3. Diagnosis must not be made solely based on the ASAS classification criteria. Classification criteria are intended to be applied in the context of an already established diagnosis.
4. The desired therapeutic target should be developed in partnership with the individual living with axSpA based on their needs and priorities, within the available resources, and not solely based on disease indices.
5. ASDAS is a composite outcome using questions 2, 3 and 6 from BASDAI, patient global, and CRP/ESR. If patient global is unavailable, replacing it with the total BASDAI score is an acceptable last resort.
6. Non-pathological inflammatory and/or structural changes can be common in the general population, particularly postpartum and in physically active individuals.
7. Body surface area can be estimated using the palm method, where the patient’s palm covers approximately 1% of their body surface area
8. Tapering is typically done by extending dosing intervals, which (with the exception of certolizumab pegol) is an off-licence use of therapy with implications for the prescriber.
